# Nickel/photoredox-catalyzed three-component silylacylation of acrylates *via* chlorine photoelimination†

**DOI:** 10.1039/d4sc02164a

**Published:** 2024-04-24

**Authors:** Yejin Koo, Sungwoo Hong

**Affiliations:** a Department of Chemistry, Korea Advanced Institute of Science and Technology (KAIST) Daejeon 34141 Korea hongorg@kaist.ac.kr; b Center for Catalytic Hydrocarbon Functionalizations, Institute for Basic Science (IBS) Daejeon 34141 Korea

## Abstract

The extensive utility of organosilicon compounds across a wide range of disciplines has sparked significant interest in their efficient synthesis. Although catalytic 1,2-silyldifunctionalization of alkenes provides a promising method for the assembly of intricate organosilicon frameworks with atom and step economy, its advancement is hindered by the requirement of an external hydrogen atom transfer (HAT) agent in photoredox catalysis. Herein, we disclose an efficient three-component silylacylation of α,β-unsaturated carbonyl compounds, leveraging a synergistic nickel/photoredox catalysis with various hydrosilanes and aroyl chlorides. This method enables the direct conversion of acrylates into valuable building blocks that contain both carbonyl and silicon functionalities through a single, redox-neutral process. Key to this reaction is the precise activation of the Si–H bond, achieved through chlorine radical-induced HAT, enabled by the photoelimination of a Ni–Cl bond. Acyl chlorides serve a dual role, functioning as both acylating agents and chloride donors. Our methodology is distinguished by its mild conditions and extensive substrate adaptability, significantly enhancing the late-stage functionalization of pharmaceuticals.

## Abstract 2

By leveraging the synergistic interplay between nickel and photoredox catalysis, we have developed a three-component silylacylation strategy for various acrylates, yielding valuable building blocks with carbonyl and silicon functionalities.

## Graphical abstract

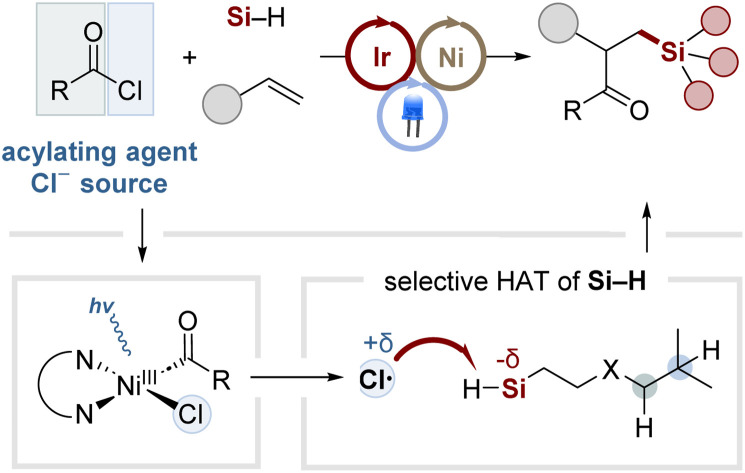

## Introduction

**Scheme 1:**
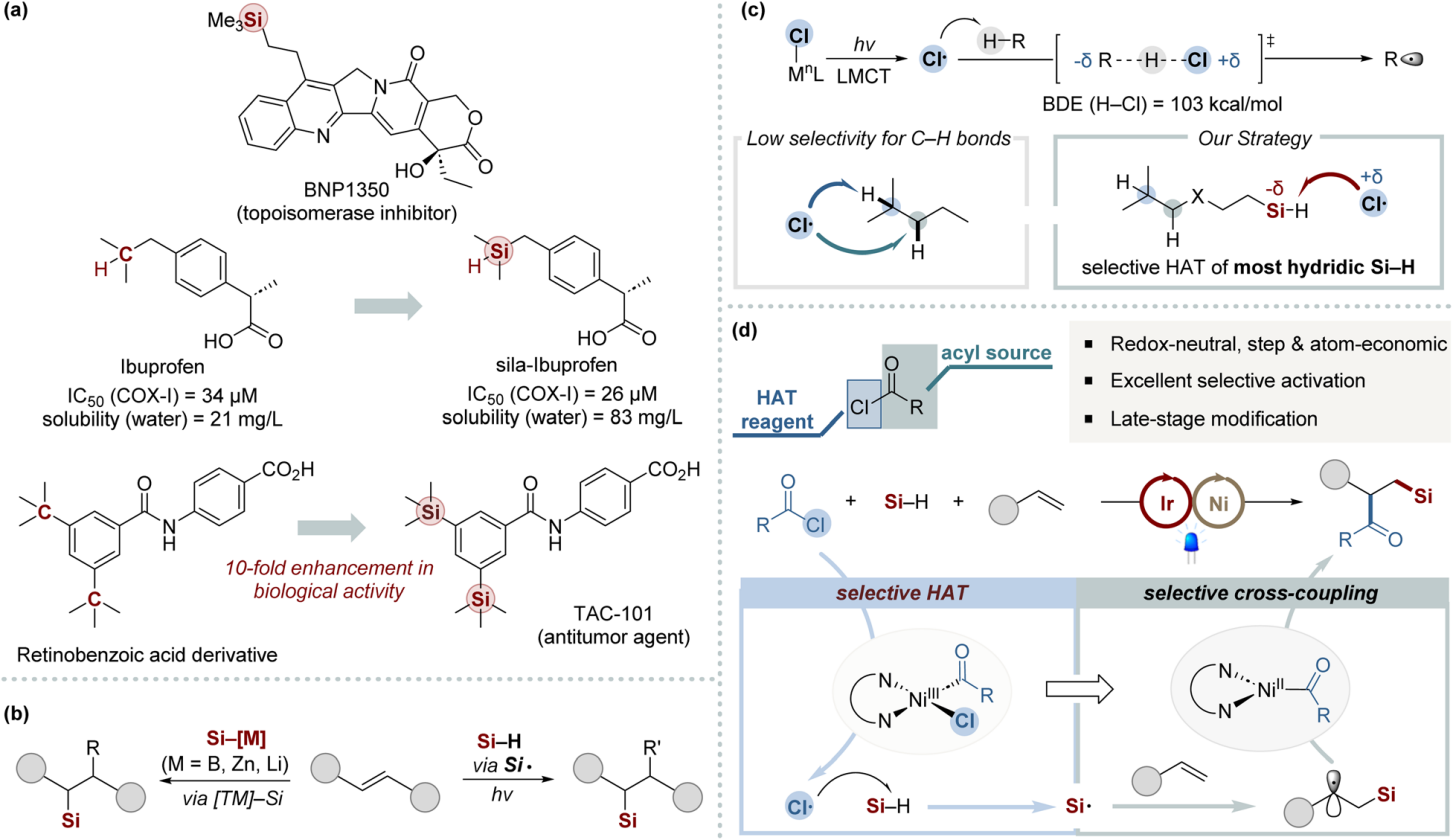
(a) Sila-substitution in bioactive substrates. (b) Previous works for 1,2-silylfunctionalization of alkenes. (c) The state-of-the-art application of chlorine photoelimination. (d) Three-component silylacylation *via* chlorine photoelimination (this work).

Organosilicon compounds have received significant interest over recent decades owing to their distinctive properties and broad applications across material science, pharmaceuticals, and organic synthesis (Scheme 1a).^1^ Consequently, it is imperative to develop efficient synthesis methods for organosilicon compounds that provide a wide array of steric and electronic environments around the silyl group. Among the various methods for C–Si bond formation, the silylfunctionalization of alkenes stands out as a particularly straightforward and atom-economic approach, converting common alkene feedstocks into valuable organosilicon derivatives.^2–9^ The simultaneous introduction of a silicon unit and an additional functional group is invaluable for the rapid assembly of complex organosilicon frameworks. However, the development of efficient and practical methods for the 1,2-silyldifunctionalization of alkenes remains a significant challenge. This primarily arises from the difficulty in avoiding side reactions, notably hydrosilylation and dehydrogenative silylation.

Recent advancements in multicomponent silyl functionalization methods, especially those developed by the Engle,^5^ Yin,^6^ and Brown groups,^7^ represent notable progress in the field (Scheme 1b, left). However, these methods face limitations due to the required preactivation of Si–M reagents^8^ and their limited scope regarding silyl substituents.^2,9^ This confines their utility primarily to simple silanes, reducing their effectiveness with bulkier silyl groups. On the other hand, radical-mediated silyl functionalization offers a viable alternative by directly activating hydrosilanes to form silyl radicals using external HAT regents, such as peroxides, or through oxidative pathways.^2*a*,*b*^ Such strategies have been significantly advanced by recent innovations in photoredox chemistry (Scheme 1b, right).^10^ These advancements provide a mild and efficient strategy for generating silyl radicals. Nonetheless, the field continues to face notable obstacles in realizing efficient 1,2-silyldifunctionalization with complex silyl groups, primarily due to the requirement for an external HAT reagent and the necessity for its meticulous integration with photoredox catalysis.

The chlorine radical is widely recognized as an effective electrophilic hydrogen atom abstractor, particularly in photoredox catalysis.^11^ By integrating chlorine radical within the HAT catalytic cycle and employing readily available chloride sources, this strategy offers high atom economy and obviates the need for external hydrogen atom abstractors. Particularly, the ligand-to-metal charge transfer (LMCT) within Ni(iii)–chloride complexes presents an elegant method for catalytically generating chlorine radicals under mild conditions (Scheme 1c).^11–14^ Building upon foundational work by the Doyle group,^12^ notable advancements have been achieved by combining Ni-catalyzed cross-coupling with chlorine radical-mediated C–H bond activation through HAT. Nevertheless, the inherent electrophilicity of chlorine radicals, coupled with the subtle electronic distinctions among neighboring C–H bonds, often results in low site-selectivity.^12*e*,15^ Hence, the efficient application of this strategy in multicomponent cross-coupling remains underexplored, with few examples utilizing photoeliminated chlorine atoms to generate heteroatom-centered radicals.^16^ This underscores the significant challenge in controlling chlorine radical reactivity within intricate catalytic networks in multicomponent reactions. In this context, we speculated that employing *in situ* generated chlorine radicals as highly selective HAT agents for the most hydridic Si–H bonds could effectively generate silyl radicals (Scheme 1d). The resulting silyl radicals are subsequently poised to readily react with alkenes. Additionally, we anticipate that acyl chlorides can fulfill a dual function: acting as acylating agents and chloride donors within nickel-catalyzed systems. Herein, we report the development of an efficient 1,2-silylcarbofunctionalization of alkenes, leveraging synergistic nickel/photoredox catalysis founded on a chlorine photoelimination strategy. This method demonstrates broad utility across various silanes, maintains high functional group tolerance, and is suitable for the late-stage functionalization of an extensive range of pharmaceuticals.

## Results and discussion

We initiated our investigation by employing 4-*t*-butyl benzoyl chloride 1a, ethyl acrylate 2a, and tris(trimethylsilyl)silane (TTMSS) 3a as model substrates to establish the optimal conditions for our reaction (Table 1). During this optimization, we encountered the significant formation of a hydrosilylation byproduct,^4^ highlighting the challenge in attaining selectivity in such multicomponent couplings. Through a comprehensive evaluation of various parameters, we found that the use of Ni(cod)_2_, 4,4′-di-*tert*-butyl-2,2′-bipyridine (dtbbpy) as the ligand, along with Ir[(dFCF_3_ppy)_2_(dtbbpy)][PF_6_] [Ir-I] as the co-catalyst under blue light irradiation, effectively facilitated the synthesis of the desired silylacylated product 4a, with a yield of 78% (entry 1). While the use of Ni(ii) precatalysts led to lower efficiency (entries 2 and 3), reducing the amount of catalyst and ligand had only a minor impact on the reaction's success (entry 4). No product formation occurred with the high-reduction-potential photocatalyst Ir(ppy)_3_, suggesting that the generation of an acyl radical through reduction of the acyl chloride is unlikely (entry 5).^17^ Employing ethyl acetate as the solvent resulted in reduced reaction efficiency (entry 6), while an increased ratio of alkene to hydrosilane slightly decreased the yield (entry 7). The use of 2,6-lutidine as a base afforded a comparable yield (entry 8), and further screening revealed the beneficial role of base in enhancing reaction efficiency. Control experiments further confirmed that nickel, the photocatalyst, and light are essential components for this synthesis to proceed (entries 9 and 10).

**Table 1:** Optimization for the reaction conditions^a^

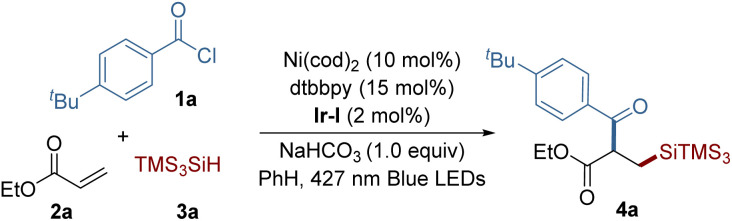		
Entry	Deviation from standard condition	Yield^b^ (%)
1	None	78 (76)^c^
2	Using NiCl_2_·glyme instead of Ni(cod)_2_	23
3	Using NiBr_2_·glyme instead of Ni(cod)_2_	11
4	5 mol% of Ni(cod)_2_, 6 mol% of dtbbpy	66
5	Ir(ppy)_3_ instead of Ir-I	NR
6	EtOAc instead of PhH	51
7	Using 2a (2.0 equiv.) and 3a (1.5 equiv.)	66
8	2,6-Lutidine instead of NaHCO_3_	72
9	Without base	32
10	Without Ni catalyst, photocatalyst, or light	NR

a Reaction conditions: 1a (0.05 mmol), 2a (0.075 mmol), 3a (0.10 mmol), NaHCO_3_ (0.05 mmol) and [Ir(dFCF_3_ppy)_2_dtbbpy]PF_6_ (2 mol%) in solvent (0.5 mL) under irradiation using blue LEDs (427 nm) at 33 °C for 22 h under argon atmosphere.

b Yields were determined by ^1^H NMR spectroscopy, and caffeine was used as an internal standard.

c Isolated yield.

With the established reaction condition in hand, we set off to investigate the generality of this method. As shown in Table 2, we explored the applicability of our protocol to a diverse range of alkyl and aryl hydrosilanes, including those with bulky silyl substituents (4b–4h). These substrates are known for their problematic nature in reactions with Si-based organometallics, which highlights the robustness of our photoredox/nickel-catalyzed strategy. It is worth noting that the method demonstrated a preferential activation of hydridic Si–H bonds over numerous benzylic C–H bonds, which possess similar bond strengths, culminating in the synthesis of targeted products 4e and 4g. The method exhibited excellent tolerance toward a variety of functional groups, including methyl (4i), methoxy (4j and 4k), *tert*-butyl (4l), trimethylsilyl (4m), trifluoromethyl (4n), cyano (4o), and halogen (4p and 4q) groups at different positions of the aromatic rings, which facilitated the production of the respective compounds under standard conditions and laid the groundwork for further structural elaboration. Furthermore, alkynyl silane was also a viable substrate for this method to afford the desired product 4r. To further demonstrate the versatility of our protocol, it was applied in the late-stage functionalization of biologically relevant molecules. This was exemplified by the successful modification of an ibuprofen derivative, yielding the desired product 4s. Furthermore, compounds like menthol (4t) and borneol (4u) derivatives, despite their sensitive α-oxy C–H bonds, were effectively modified *via* selective Si–H bond activation, demonstrating the method's broad scope across diverse functional molecules.

**Table 2:** Silane scope of three-component silylacylation^a^

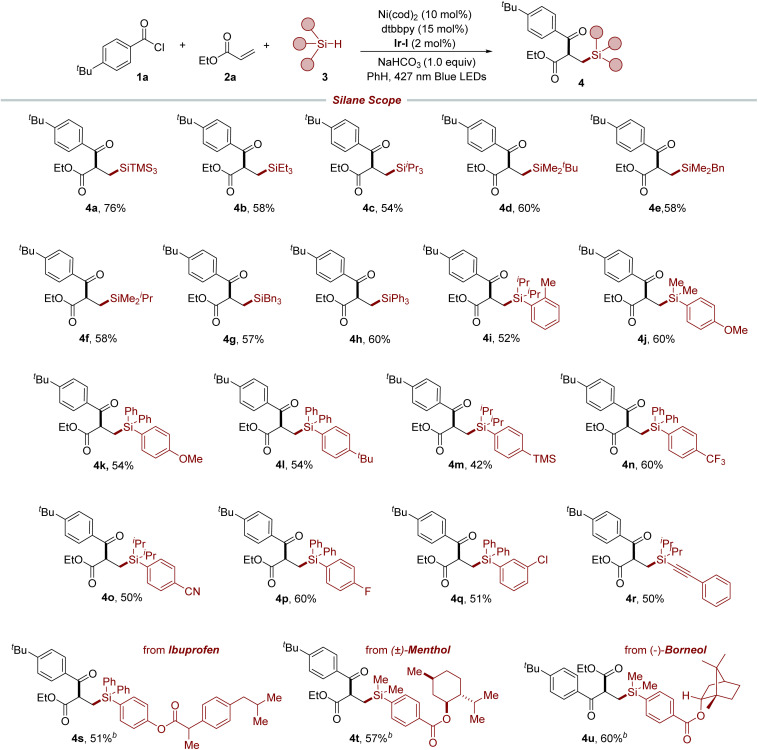

a Reaction conditions: 1a (0.05 mmol), 2a (0.075 mmol), 3 (0.10 mmol), Ni(cod)_2_ (10 mol%), dtbbpy (15 mol%), NaHCO_3_ (0.05 mmol) and [Ir(dFCF_3_ppy)_2_dtbbpy]PF_6_ (2 mol%) in PhH (0.5 mL) using blue LEDs (427 nm) at 33 °C for 22 h under Ar atmosphere.

b 48 h. Isolated yields.

Subsequently, we expanded our investigations to a range of alkenes, implementing the silylacylation protocol (Table 3). This exploration included electron-deficient acrylates that featured pivotal functional groups such as phenoxy (4ad), alkoxy (4ae and 4af), Boc-protected amine (4ag), ester (4ah), free hydroxy (4ai), and ether (4aj) – all of which were proficiently incorporated under our standard conditions to afford the corresponding difunctionalized products. Additionally, our method proved compatible with vinyl amide, generating adducts 4ak. We further extended our methodology to intricate alkenes, including those derived from menthol (4al), cholesterol (4am), estrone (4an), and eugenol (4ao), demonstrating the method's adaptability to complex molecular frameworks. Subsequently, we explored a series of acyl chlorides characterized by electron-donating and electron-withdrawing substituents positioned at the *para* and *meta* positions on the aryl ring. These variants underwent effective transformations to produce compounds featuring diverse functional groups, such as methyl (4aq), methoxy (4ar and 4au), phenyl (4av), trifluoromethoxy (4as), fluoro (4at and 4aw), trifluoromethyl (4ax) and ester (4ay) groups respectively. In addition, *ortho*-substituted aroyl chlorides (4az and 4ba) were also suitable substrates for this method, yielding the desired products with marginally reduced efficiencies.

**Table 3:** Alkene and aroyl chloride scope of three-component silylacylation^a^

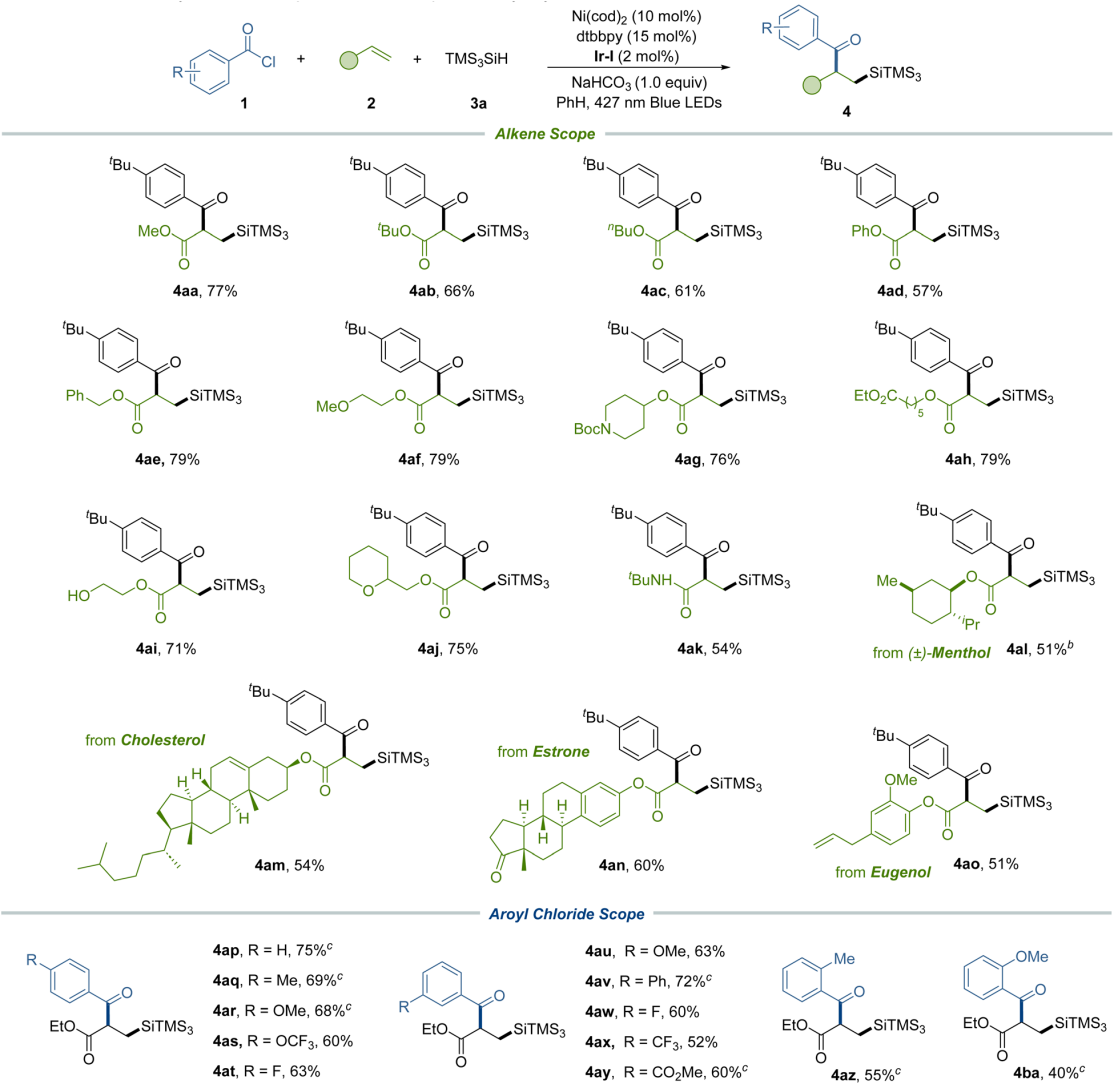

a Reaction conditions: 1 (0.05 mmol), 2 (0.075 mmol), 3a (0.10 mmol), Ni(cod)_2_ (10 mol%), dtbbpy (15 mol%), NaHCO_3_ (0.05 mmol) and [Ir(dFCF_3_ppy)_2_dtbbpy]PF_6_ (2 mol%) in PhH (0.5 mL) using blue LEDs (427 nm) at 33 °C for 22 h under Ar atmosphere.

b Ethyl acetate : PhH = 1 : 1 was used as a solvent.

c Using tetrabutylammonium bromide (25 mol%) as an additive. Isolated yields.

**Scheme 2:**
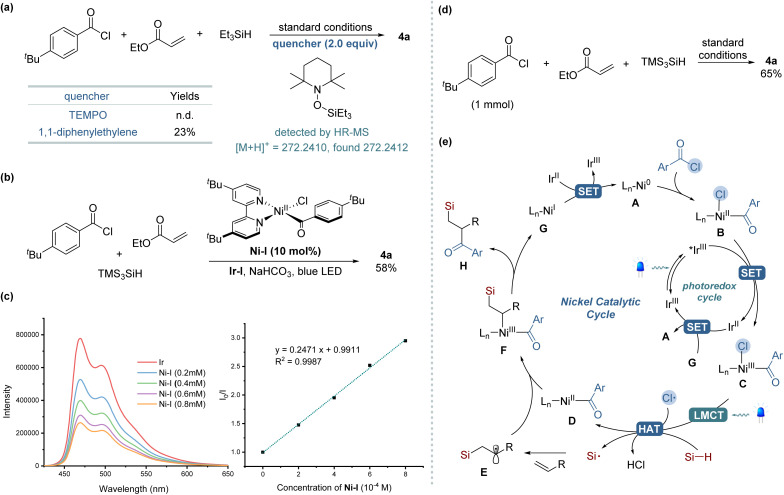
Experimental mechanistic studies and proposed reaction mechanism. (a) Radical inhibitor experiments. (b) Catalytic reactions with Ni(ii) complex. (c) Stern–Volmer quenching studies. (d) Large-scale (1 mmol) reaction. (e) Proposed mechanism.

To elucidate the mechanism underlying our silylacylation method, a series of control experiments were conducted, as shown in Scheme 2. The addition of a radical scavenger TEMPO (2.0 equiv.) under the standard reaction conditions completely inhibited the formation of the desired product, while TEMPO adducts of silyl radical was detected by HR-MS (Scheme 2a). Similar inhibitory effect was observed when using diphenylethylene, another radical trapping agent, which significantly reduced the reaction efficiency. These observations collectively suggest the involvement of radical species in the reaction pathway. In further explorations, we replaced benzoyl chloride with *N*-acylsuccinimide, a substrate known for its straightforward oxidative addition with Ni(0) species,^14^ but the desired product was not detected. However, the addition of LiCl as an external chloride source led to the formation of the product 4a, albeit with low yield (4%) (see the ESI†). These results underscore the essential role of chloride in this transformation, suggesting its pivotal role as a source of chlorine radicals for initiating the reaction process. When we employed an isolated Ni-complex [Ni(dtbbpy)(^*t*^BuPhCO)Cl] (Ni-I) in a catalytic quantity instead of the Ni(cod)_2_ precatalyst, the desired silylacylated product was obtained in a 58% yield (Scheme 2b). Conversely, employing a stoichiometric amount of this Ni-I significantly reduced the yield to 4%, indicating that an excess of Ni catalyst likely promotes unfavorable side reactions. Further investigations, particularly Stern–Volmer luminescence quenching experiments, demonstrated exclusive luminescence quenching by Ni-I complex (Scheme 2c), not by TTMSS despite its appropriate reduction potential,^10*c*,18^ which excluded direct TTMSS–photocatalyst interactions (see the ESI† for more detail). Scaling up the reaction twentyfold (1 mmol) while maintaining identical conditions successfully produced compound 4a, demonstrating the protocol's scalability and practical applicability (Scheme 2d). Based on the mechanistic investigations and corroborating literature, the proposed mechanism for the three-component silylacylation protocol is delineated in Scheme 2e.^12,13,19^ The reaction is initiated by the oxidative addition of acyl chloride to Ni(0) A to afford Ni(ii) complex B. This complex undergoes single-electron oxidation by the excited photocatalyst *Ir(iii) (*E_1/2_ = +1.21 V *vs.* SCE in MeCN),^20^ leading to the formation of a Ni(iii) species C. Under visible-light irradiation, the Ni(iii) complex engages in LMCT, resulting in the photoelimination of a chlorine radical and the regeneration of a Ni(ii) complex D. This chlorine radical mediates HAT from the hydrosilane, generating a silyl radical that combines with an alkene to yield an alkyl radical E. The capture of the alkyl radical by the Ni(ii) complex D leads to the formation of a Ni(iii) species F. This species then undergoes reductive elimination to produce the silylacylated product H and a Ni(i) complex G. The catalytic cycle completes with SET between the reduced Ir(ii) (*E*_1/2_ = −1.37 V *vs.* SCE in MeCN) and Ni(i) (*E*_p_ = −1.17 V *vs.* SCE in THF),^20^ regenerating the ground-state Ir(iii) and Ni(0).

## Conclusions

In summary, our study has successfully established a redox-neutral, three-component 1,2-silylacylation method for α,β-unsaturated alkenes, employing widely available hydrosilanes and aroyl chlorides *via* synergistic Ni/photoredox catalysis under mild conditions. This efficient and atom-economical approach leverages the unique ability of photoeliminated chlorine radicals for hydrogen abstraction, enabling selective Si–H bond activation in various hydrosilanes. Consequently, it enables the efficient and regioselective assembly of a variety of organosilicon compounds, accomplishing sequential C–C and C–Si bond formations in a single operational step. Demonstrating extensive substrate compatibility, this methodology has been further corroborated through reaction upscaling and its successful integration into the late-stage functionalization of complex structures. Given the significant utility of organosilicon entities across various fields, our findings are set to markedly contribute to the advancement of synthetic strategies for silicon-bearing molecules.

## Data availability

Detailed synthetic procedures, supporting experimental results, and complete characterization data for all new compounds can be found in the ESI.†

## Author contributions

Y. K. performed the experiments, analyzed the data, and wrote the manuscript. S. H. directed the project and wrote the manuscript.

## Conflicts of interest

There are no conflicts to declare.

## Supplementary Material

SC-015-D4SC02164A-s001
