# Supplementary material for: Nickel/photoredox-catalyzed three-component silylacylation of acrylates via chlorine photoelimination
Source: Chem Sci. 2024 Apr 24;15(20):7707–13. doi: 10.1039/d4sc02164a (PMC11110154; doi:10.1039/d4sc02164a)
Supplement: SC-015-D4SC02164A-s001 [file SC-015-D4SC02164A-s001.pdf]

Electronic Supplementary Material (ESI) for Chemical Science.
This journal is © The Royal Society of Chemistry 2024

Supporting Information

Nickel/Photoredox-Catalyzed Three-Component Silylacylation of Acrylates via Chlorine Photoelimination

*Yejin Koo[a,b], Sungwoo Hong[b,a]**

Email : hongorg@kaist.ac.kr

[a]Department of Chemistry, Korea Advanced Institute of Science and Technology (KAIST), Daejeon, 34141, Korea

[b]Center for Catalytic Hydrocarbon Functionalizations, Institute for Basic Science (IBS), Daejeon, 34141, Korea

I. General Methods and Materials S2

II. Experimental Procedure S3

III. Optimization Table S6

IV. Control Experiments S8

V. Mechanistic investigation S12

VI. Compound Characterizations S17

Appendix I

Spectral Copies of ^{1}H-, ^{13}C- and ^{19}F-NMR Data S41

I. General Methods and Materials

Unless stated otherwise, reactions were performed in flame-dried glassware. Analytical thin layer chromatography (TLC) was performed on precoated silica gel 60 F^{254} plates and silica gel 60 RP-18 F^{254}s, and visualization on TLC was achieved by UV light (254 and 365 nm). Flash column chromatography was performed on silica gel (400-630 mesh) or a Combi*Flash*® R_f^+ system with Redi*Sep*® R_f silica columns (230-400 mesh) using a proper eluent. ^{1}H NMR was recorded on Brucker Avance 400 MHz, Brucker Avance 500 MHz or Agilent Technologies DD2 600 MHz. Chemical shifts were quoted in parts per million (ppm) referenced to the appropriate solvent peak or 0.0 ppm for tetramethylsilane. The following abbreviations were used to describe peak splitting patterns when appropriate: br = broad, s = singlet, d = doublet, t = triplet, q = quartet, m = multiplet, dd = doublet of doublet, td = triplet of doublet, ddd = doublet of doublet of doublet. Coupling constants, *J*, were reported in hertz unit (Hz). ^{13}C NMR was recorded on Brucker Avance 125 MHz, or Agilent Technologies DD2 150 MHz and was fully decoupled by broad band proton decoupling. Chemical shifts were reported in ppm referenced to the centerline of a pentet at 53.8 ppm of CD_2Cl_2. ^{19}F NMR was recorded on Brucker Advance 376 MHz. High resolution mass spectroscopy was conducted on a Bruker Daltonik micrOTOF-QII and obtained by using ESI from Korea Basic Science Institute (Ochang). The Absorption spectra were measured by a spectrophotometer (V-530 UV/Vis Spectrophotometer, Jasco, Inc.). Commercial grade reagents and solvents were used without further purification except as indicated below.

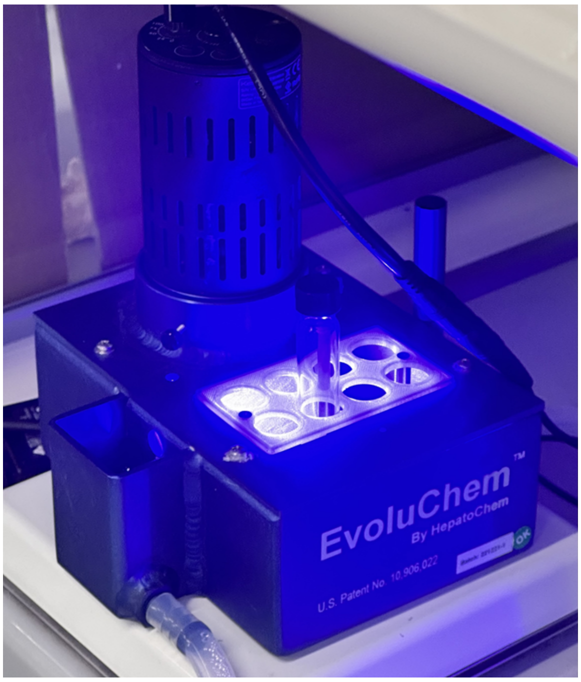

Figure S1. Set-up for the visible light induced reaction (25% Kessil 427 nm in hepatochem reactor)

II. Experimental Procedure

Trialkylhydrosilanes **3a-3h** and acrylates **2aa-2af, 2ai, and 2ak** were purchased from commercial suppliers (Tokyo Chemical Industry Co. or Sigma-Aldrich Co.) and used as received unless otherwise noted. ^{1}H and ^{13}C NMR spectra were in agreement with those in the literature. [1-4]

Scheme S1.

General procedure 1 for the preparation of hydrosilanes 3i-3l and 3p-3r (GP1)[1]

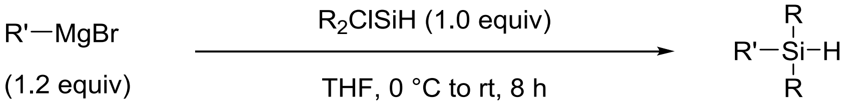

In a 100 mL round-bottom flask, R_2ClSiH (1.11 mL, 10.0 mmol) in THF (6 mL) was cooled to 0 °C. A solution of phenylmagnesium bromide (24 mL, 0.5 M in THF) was added dropwise slowly over 15 min. Then reaction was allowed to warm to room temperature and stirred for 8 hours. The reaction mixture was quenched with NH_4Cl (5 mL, sat. aq.) and the product was extracted with Et_2O (15 mL × 3). The organic layer was washed with water (20 mL), brine (20 mL), dried over $MgSO_4$, and then concentrated under reduced pressure. The residue was purified by flash silica gel column chromatography to afford the hydrosilane.

Scheme S2.

General procedure 2 for the preparation of hydrosilanes 3m-3o and 3s-3u (GP2)[2]

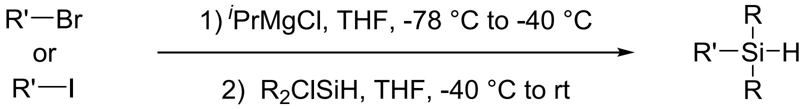

In a 100 mL round-bottom flask, aryl bromide or aryl iodide (5.0 mmol, 1.0 equiv) in THF (10 mL) was cooled to –78 °C. ^{i}PrMgCl (3 mL, 2.0 M in THF, 6.0 mmol, 1.2 equiv) was added dropwise slowly over 15 min. The resulting mixture was allowed to warm to -40 °C in 2 h and maintained at -40 °C for another 2 h before the dropwise addition of R_2ClSiH (6.0 mmol, 1.2 equiv). The reaction was allowed to warm to room temperature and stirred for 8 h. The reaction mixture was quenched with NH_4Cl (10 mL, sat. aq.) and the mixture was extracted with CH_2Cl_2 (15 mL × 3). The combined organic layer was washed with water (20 mL), brine (20 mL), dried over anhydrous Na_2SO_4, and then concentrated under reduced pressure. The residue was purified by flash silica gel column chromatography to afford the hydrosilane.

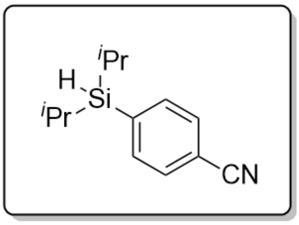

4-(diisopropylsilyl)benzonitrile (3o). Colorless oil. ^{1}H NMR (600 MHz, CD_2Cl_2) δ 7.64 (m, 4H), 3.97 (m, 1H), 1.32 – 1.22 (m, 2H), 1.07 (d, J = 7.3 Hz, 6H), 0.98 (d, J = 7.4 Hz, 6H). ^{13}C NMR (125 MHz, CD_2Cl_2) δ 141.7, 136.3, 131.2, 119.3, 113.2, 18.6, 18.5, 10.9. HRMS (ESI$^+$) m/z calcd. $C_{13}H_{20}NSi^+$ $[M+H]^+$: 218.1365, found: 218.1365.

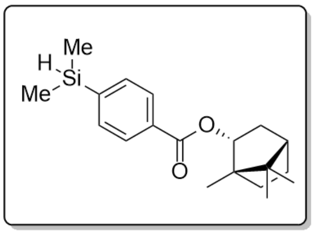

(1*S*,2*R*,4*S*)-1,7,7-trimethylbicyclo[2.2.1]heptan-2-yl 4-(dimethylsilyl)benzoate (3u). Colorless oil. ^{1}H NMR (600 MHz, CD_2Cl_2) δ 8.03 (d, J = 8.1 Hz, 2H), 7.66 (d, J = 8.1 Hz, 2H), 5.12 (m, 1H), 4.46 (m, 1H), 2.60 – 2.41 (m, 1H), 2.16 (m, 1H), 1.83 (m, 1H), 1.75 (m, 1H), 1.46 – 1.38 (m, 1H), 1.32 (m, 1H), 1.13 (m, 1H), 0.99 (s, 3H), 0.93 (m, 6H), 0.38 (d, J = 3.8 Hz, 6H). ^{13}C NMR (125 MHz, CD_2Cl_2) δ 167.0, 144.0, 134.4, 132.0, 128.8, 80.9, 49.5, 48.3, 45.5, 37.3, 28.4, 27.8, 19.9, 19.1, 13.8, -3.8. HRMS (ESI$^+$) m/z calcd. $C_{19}H_{28}O_2NaSi^+$ $[M+Na]^+$: 339.1756, found: 339.1757.

Scheme S3.

General procedure 3 for the preparation of acrylates 2ag, 2ah, 2aj, and 2al-2ao (GP3)[5]

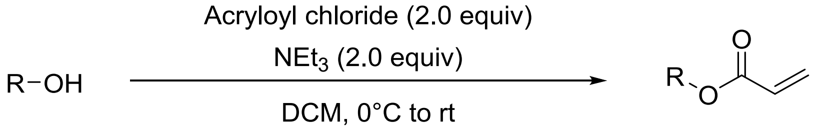

To a solution of R–OH and Et_3N (20 mmol, 2.0 equiv) in CH_2Cl_2 (25 mL) at 0 °C acryloyl chloride (20 mmol, 2.0 equiv) was added dropwise. The reaction mixture was warmed to rt and stirred for 12 h. The reaction mixture was quenched with water (15 mL) and the mixture was extracted with CH_2Cl_2 (15 mL × 3). The combined organic layer was dried over $MgSO_4$, and then concentrated under reduced pressure. The residue was purified by flash silica gel column chromatography to afford the acrylate.

Scheme S4.

General procedure 4 for three-component silylacylation (GP4)

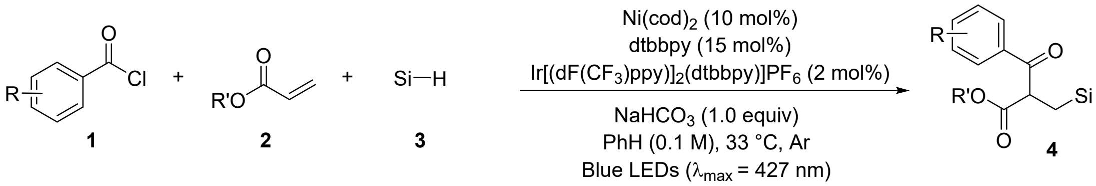

In an argon-filled glovebox, to a flame-dried 12 mL test tube equipped with a magnetic bar were added with $NaHCO_3$ (1.0 equiv, 0.05 mmol) and Ir[(dF(CF_3)ppy)]$_2$(dtbbpy)]PF_6 (0.02 equiv, 1 μmol). To the reaction vial was added 100 μL of a dark purple solution of Ni(cod)$_2$ (0.1 equiv, 0.005 mmol) and 4,4′-di-tert-butyl-2,2′-bipyridine (0.15 equiv, 0.0075 mmol) in benzene which had been stirred for 15 minutes prior to use. This was followed successively by 100 μL of a stock solution of hydrosilane (2.0 equiv, 0.1 mmol), 100 μL of a stock solution of alkene (1.5 equiv, 0.075 mmol), and 200 μL of a stock solution of aroyl chloride (1.0 equiv, 0.05 mmol) in benzene, turning the solution a deep red color. The tube was sealed with a screw cap, removed from the glove box, irradiated with Kessil PR160-427 nm blue LED with 25% intensity, and stirred for 22 hours in a water bath set to 33°C. After reaction completion, the reaction mixture was diluted with distilled water and was extracted with EtOAc three times. The combined organic layer was dried over anhydrous Na_2SO_4, filtered through a Celite pad, and then concentrated under reduced pressure. After removal of solvent, the residue was purified by flash column chromatography on silica gel (eluent: ethyl acetate/*n*-hexane = 1:15 – 1:20).

1 mmol scale procedure

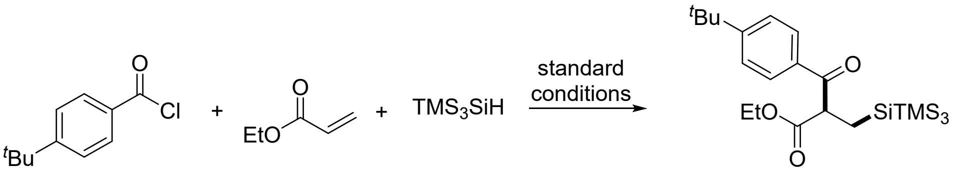

4a, 65%

In an argon-filled glovebox, to a flame-dried 50 ml round-bottom flask equipped with a magnetic bar were added with $NaHCO_3$ (1.0 equiv, 1 mmol) and Ir[(dF(CF_3)ppy)]$_2$(dtbbpy)]PF_6 (0.02 equiv, 0.02 mmol). To the reaction vial was added 2 mL of a dark purple solution of Ni(cod)$_2$ (0.1 equiv, 0.1 mmol) and 4,4′-di-tert-butyl-2,2′-bipyridine (0.15 equiv, 0.15 mmol) in benzene which had been stirred for 15 minutes prior to use. This was followed successively by hydrosilane (2.0 equiv, 2 mmol), alkene (1.5 equiv, 1.5 mmol), aroyl chloride (1.0 equiv, 1 mmol), and dry benzene (8 mL). The tube was sealed with a rubber septa, removed from the glove box, irradiated with Kessil PR160-427 nm blue LED with 25% intensity, and stirred for 22 hours in a water bath set to 33°C. After reaction completion, the reaction mixture was diluted with distilled water and was extracted with EtOAc three times. The combined organic layer was dried over anhydrous Na_2SO_4, filtered through a Celite pad, and then concentrated under reduced pressure. After removal of solvent, the residue was purified by flash column

chromatography on silica gel.

III. Optimization Table

Table S1. Effect of ligand [a]

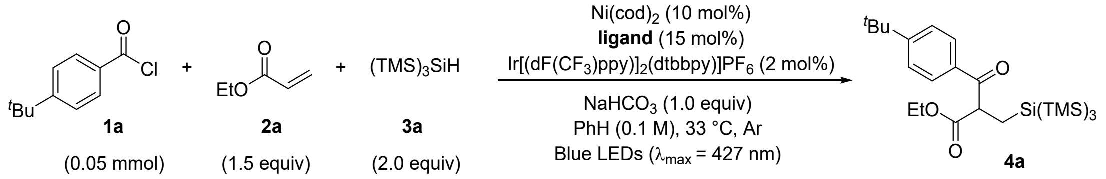

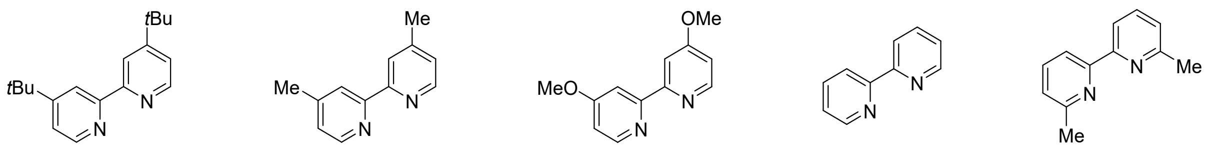

L1, 78% **L2**, 32% **L3**, 20% **L4**, 22% **L5**, 7%

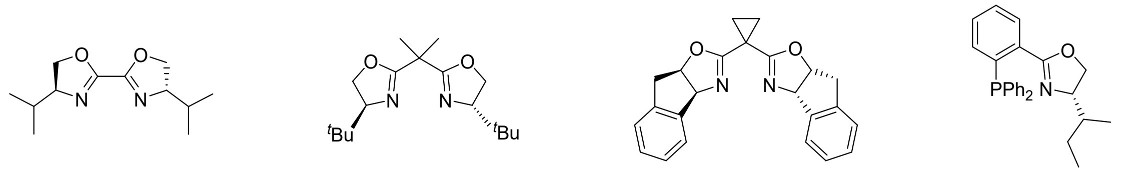

L15, 22% **L6,** N.R. **L7,** N.R. **L8,** N.R.

[a] Yields were determined by ^{1}H NMR with caffeine as internal standard.

Table S2. Effect of base [a]

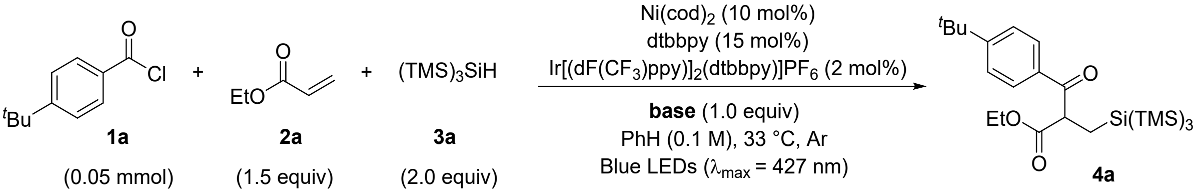

Entry	Base	Yield (%)
1	$NaHCO_3$	78
2	none	32
3	K_3PO_4	28
4	K_2HPO_4	32
5	KH_2PO_4	23
6	Na_3PO_4	24

7	Na_2HPO_4	25
8	NaH_2PO_4	20
9	$KHCO_3$	23
10	Na_2CO_3	26
11	K_2CO_3	25
12	Cs_2CO_3	24
13	$K_3PO_4\,H_2O$	23
14	NaTFA	7
15	NaOAc	33
16	NaO^{t}Bu	17
17	KO^{t}Bu	16

[a] Yields were determined by ^{1}H NMR with caffeine as internal standard.

Table S3. Effect of solvent [a]

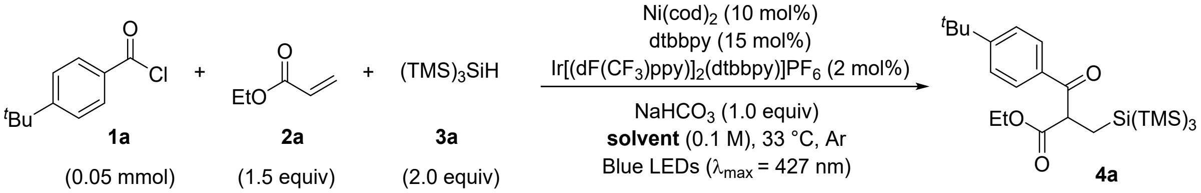

Entry	Solvent	Yield (%)
1	PhH	78
2	1,4-dioxane	51
3	MeCN	12
4	toluene	70
5	DMSO	trace
6	DMF	NR
7	DMA	NR
8	1,2-DCE	17
9	DCM	trace
10	HFIP	NR
11	**EA**	51
12	iPrOAc	60

[a] Yields were determined by ^{1}H NMR with caffeine as internal standard.

Table S4. Effect of photocatalyst [a]

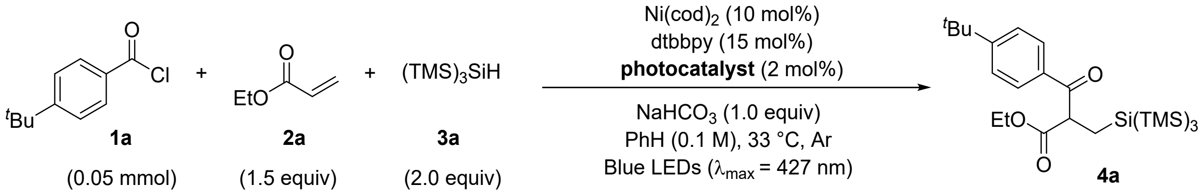

Entry	Photocatalyst	Yield (%)
1	$[Ir(dFCF_3ppy)_2(dtbbpy)]PF_6$	78
2	$[Ir(dFCF_3ppy)_2(bpy)]PF_6$	16
3	$Ir(dFppy)_3$	10
4	$[Ir(dFppy)_2(dtbbpy)]PF_6$	11
5	$[Ir(dFCF_3ppy)_2(5,5'\text{-}dCF_3bpy)]PF_6$	26
6	$[Ir(ppy)_2 (bpy)]PF_6$	trace
7	*fac*-$[Ir(ppy)_3]$	NR
8	$[Ir(ppy)_2 (dtbbpy)]PF_6$	11
9	$[Mes\text{-}Acr]^+ClO_4^-$	NR
10	Without photocatalyst	NR

[a] Yields were determined by ^{1}H NMR with caffeine as internal standard.

IV. Control Experiments

Radical inhibitor experiment with TEMPO

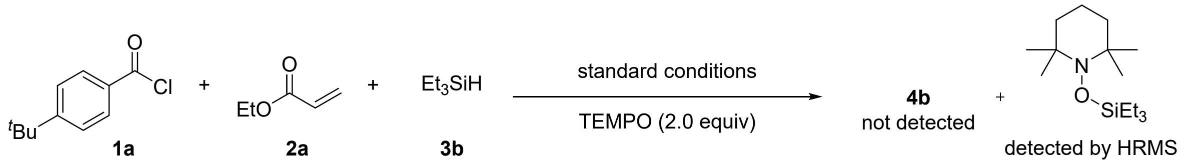

In an argon-filled glovebox, to a flame-dried 12 mL test tube equipped with a magnetic bar were added with $NaHCO_3$ (1.0 equiv, 0.05 mmol), $Ir[(dF(CF_3)ppy)]_2(dtbbpy)]PF_6$ (0.02 equiv, 1 μmol), and TEMPO (2,2,6,6-tetramethylpiperidine-1-oxyl, 2.0 equiv, 0.1 mmol). To the reaction vial was added 100 μL of a dark purple solution of $Ni(cod)_2$ (0.1 equiv, 0.005 mmol) and 4,4′-di-tert-butyl-2,2′-bipyridine (0.15 equiv, 0.0075 mmol) in benzene which had been stirred for 15 minutes prior to use. This was followed successively by 100 μL of a stock solution of hydrosilane (2.0 equiv, 0.1 mmol), 100 μL of a stock solution of alkene (1.5 equiv, 0.075 mmol), and 200 μL of a stock solution of aroyl chloride (1.0 equiv, 0.05 mmol) in benzene. The tube was sealed with a screw cap, removed from the glove box, irradiated with Kessil PR160-427 nm blue LED with 25% intensity, and stirred for 22 hours in a water bath set to 33°C. The crude reaction mixture was analyzed directly by ^{1}H-NMR and HRMS (ESI). As a result, compound **4b** was not detected, suggesting that the reaction proceeds via a radical

pathway.

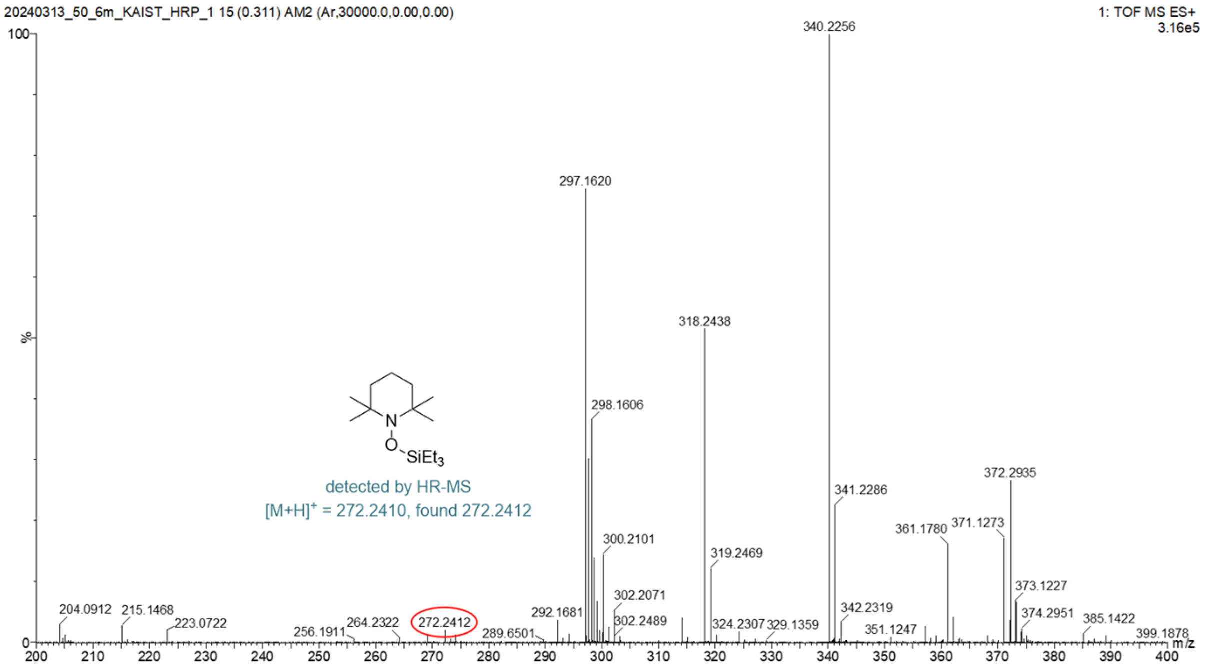

Figure S2. HRMS (ESI) spectra for radical inhibitor experiment with TEMPO

Radical inhibitor experiment with 1,1-diphenylethylene

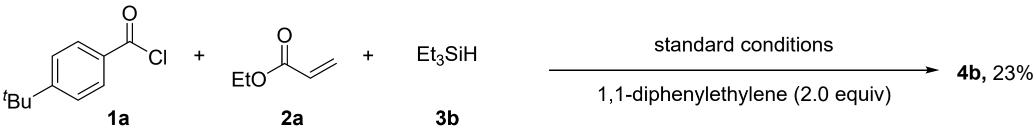

In an argon-filled glovebox, to a flame-dried 12 mL test tube equipped with a magnetic bar were added with $NaHCO_3$ (1.0 equiv, 0.05 mmol), $Ir[(dF(CF_3)ppy)]_2(dtbbpy)]PF_6$ (0.02 equiv, 1 μmol), and 1,1-diphenylethylene (2.0 equiv, 0.1 mmol). To the reaction vial was added 100 μL of a dark purple solution of $Ni(cod)_2$ (0.1 equiv, 0.005 mmol) and 4,4′-di-tert-butyl-2,2′-bipyridine (0.15 equiv, 0.0075 mmol) in benzene which had been stirred for 15 minutes prior to use. This was followed successively by 100 μL of a stock solution of hydrosilane (2.0 equiv, 0.1 mmol), 100 μL of a stock solution of alkene (1.5 equiv, 0.075 mmol), and 200 μL of a stock solution of aroyl chloride (1.0 equiv, 0.05 mmol) in benzene. The tube was sealed with a screw cap, removed from the glove box, irradiated with Kessil PR160-427 nm blue LED with 25% intensity, and stirred for 22 hours in a water bath set to 33°C. The crude reaction mixture was analyzed directly by ^1H-NMR using caffeine as an internal standard.

Stoichiometric experiment with complex Ni-I

The oxidative addition complex **Ni-I** was prepared following literature procedures.[6]

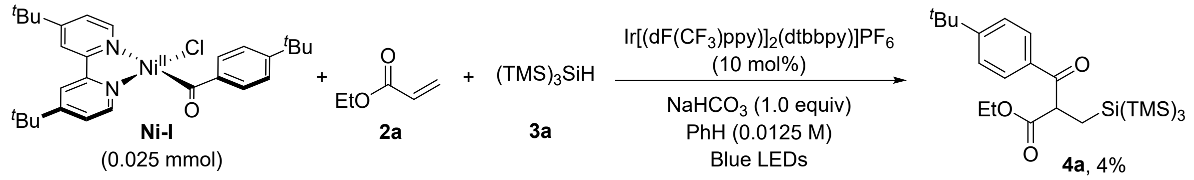

In an argon-filled glovebox, to a flame-dried 12 mL test tube equipped with a magnetic bar were added with complex **Ni-I** (1.0 equiv, 0.025 mmol) $NaHCO_3$ (1.0 equiv, 0.025 mmol) and $Ir[(dF(CF_3)ppy)]_2(dtbbpy)]PF_6$ (0.1 equiv, 2.5 μmol). To the reaction vial was added 100 μL of a stock solution of hydrosilane (2.0 equiv, 0.05 mmol), 100 μL of a stock solution of alkene (1.5 equiv, 0.0375 mmol), and dry benzene (1.8 mL). The tube was sealed with a screw cap, removed from the glove box, irradiated with Kessil PR160-427 nm blue LED with 25% intensity, and stirred for 22 hours in a water bath set to 33°C. The crude reaction mixture was analyzed directly by ^{1}H-NMR using caffeine as an internal standard.

Catalytic experiment with complex Ni-I

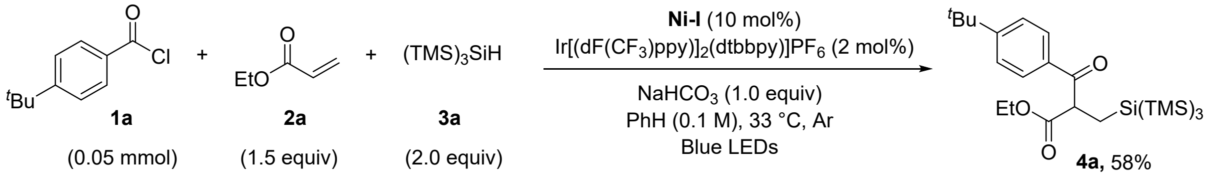

In an argon-filled glovebox, to a flame-dried 12 mL test tube equipped with a magnetic bar were added with **Ni-I** (0.1 equiv, 0.005 mmol), $NaHCO_3$ (1.0 equiv, 0.05 mmol) and $Ir[(dF(CF_3)ppy)]_2(dtbbpy)]PF_6$ (0.02 equiv, 1 μmol). To the reaction vial was added successively 100 μL of a stock solution of hydrosilane (2.0 equiv, 0.1 mmol), 100 μL of a stock solution of alkene (1.5 equiv, 0.075 mmol), and 200 μL of a stock solution of aroyl chloride (1.0 equiv, 0.05 mmol) in benzene and dry benzene (0.3 mL). The tube was sealed with a screw cap, removed from the glove box, irradiated with Kessil PR160-427 nm blue LED with 25% intensity, and stirred for 22 hours in a water bath set to 33°C. The crude reaction mixture was analyzed directly by ^{1}H-NMR using caffeine as an internal standard.

Control experiment with *N*-acylSuccinimide

The *N*-acylsuccinimide **1bb** was prepared following literature procedures.[7]

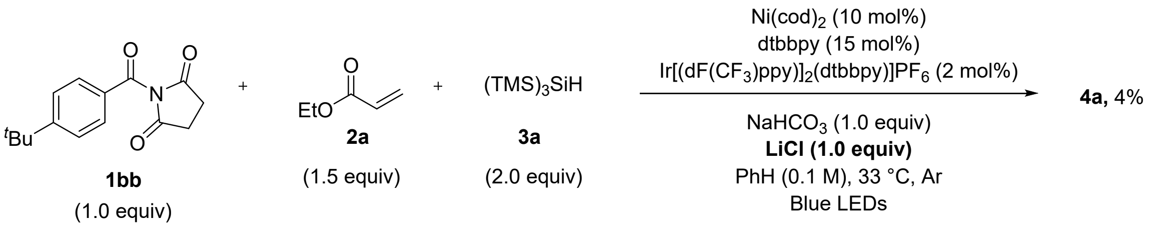

In an argon-filled glovebox, to a flame-dried 12 mL test tube equipped with a magnetic bar were added with *N*-acylsuccinimide (1.0 equiv, 0.05 mmol), $NaHCO_3$ (1.0 equiv, 0.05 mmol), Ir[(dF(CF_3)ppy)]$_2$(dtbbpy)]PF_6 (0.02 equiv, 1 μmol), and LiCl (1.0 equiv, 0.05 mmol). To the reaction vial was added 100 μL of a dark purple solution of Ni(cod)$_2$ (0.1 equiv, 0.005 mmol) and 4,4′-di-tert-butyl-2,2′-bipyridine (0.15 equiv, 0.0075 mmol) in benzene which had been stirred for 15 minutes prior to use. This was followed successively by 100 μL of a stock solution of hydrosilane (2.0 equiv, 0.1 mmol),100 μL of a stock solution of alkene (1.5 equiv, 0.075 mmol) and dry benzene (0.2 mL). The tube was sealed with a screw cap, removed from the glove box, irradiated with Kessil PR160-427 nm blue LED with 25% intensity, and stirred for 22 hours in a water bath set to 33°C. The crude reaction mixture was analyzed directly by ^{1}H-NMR using caffeine as an internal standard.

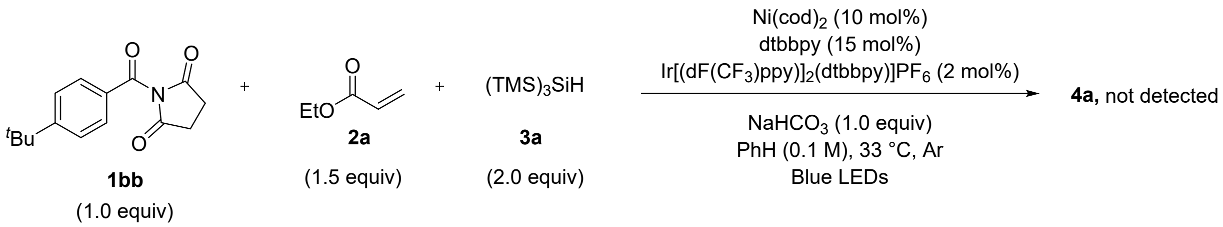

In an argon-filled glovebox, to a flame-dried 12 mL test tube equipped with a magnetic bar were added with *N*-acylsuccinimide (1.0 equiv, 0.05 mmol), $NaHCO_3$ (1.0 equiv, 0.05 mmol), and Ir[(dF(CF_3)ppy)]$_2$(dtbbpy)]PF_6 (0.02 equiv, 1 μmol). To the reaction vial was added 100 μL of a dark purple solution of Ni(cod)$_2$ (0.1 equiv, 0.005 mmol) and 4,4′-di-tert-butyl-2,2′-bipyridine (0.15 equiv, 0.0075 mmol) in benzene which had been stirred for 15 minutes prior to use. This was followed successively by 100 μL of a stock solution of hydrosilane (2.0 equiv, 0.1 mmol),100 μL of a stock solution of alkene (1.5 equiv, 0.075 mmol) and dry benzene (0.2 mL). The tube was sealed with a screw cap, removed from the glove box, irradiated with Kessil PR160-427 nm blue LED with 25% intensity, and stirred for 22 hours in a water bath set to 33°C. The crude reaction mixture was analyzed directly by LC-MS and ^{1}H-NMR using caffeine as an internal standard. The desired product **4a** was not observed without LiCl additive, underscoring the essential role of chloride in this transformation.

V. Mechanistic investigation

Stern-Volmer Quenching Experiments

Procedure: Following stock solutions and samples were prepared in an argon-filled glovebox, and analyzed immediately.

Photocatalyst (0.1 mM): Ir[(dF(CF_3)ppy)]$_2$(dtbbpy)]PF_6 (0.90 mg, 0.8 μmol) in 8.0 mL 1,2-dichloroethane

4-*tert*butyl benzoyl chloride (0.02 M): benzoyl chloride (15.6 μL, 80 μmol) in 4.0 mL 1,2-dichloroethane

Ethyl acrylate (0.02 M): Ethyl acrylate (8.5 μL, 80 μmol) in 4.0 mL 1,2-dichloroethane

TMS_3SiH (0.02 M): TTMSS (24.7 μL, 80 μmol) in 4.0 mL 1,2-dichloroethane

Ni(dtbbpy)(*t*BuPhCO)Cl (Ni-I) solution was prepared by mixing 0.04 mmol Ni(cod)$_2$ with 0.04 mmol dtbbpy in 10.0 mL 1,2-dichloroethane. After 30 min, 0.04 mmol 4-*tert*butybenzoyl chloride was then added. After 30 minutes, 1.0 mL of the mixture was transferred to a volumetric flask of 4.0 mL, and diluted to give a solution of (1.0 mM).

With the above solutions, the samples for the analysis were prepared as following.

4-*tert*butyl benzoyl chloride (1a) : The process is the same as the above. The final concentrations of photocatalyst was 0.01 mM, and the concentrations of benzoyl chloride were 0.004 M, 0.008 M, 0.012 M, 0.016 M.

Ethyl acrylate (2a) : The process is the same as the above. The final concentrations of photocatalyst was 0.01 mM, and the concentrations of ethyl acrylate were 0.004 M, 0.008 M, 0.012 M, 0.016 M.

TMS_3SiH (3a) : To four volumetric flasks of 4.0 mL were added 0.4 mL, 0.8 mL, 1.2 mL and 1.6 mL TMS_3SiH stock solution, followed by the addition of 0.2 mL of photocatalyst stock solution (0.1 mM). Then, the solutions were diluted to give the final concentration of 0.01 mM for photocatalyst, and of 0.004 M, 0.008 M, 0.012 M, 0.016 M for TMS_3SiH.

Ni(dtbbpy)(*t*BuPhCO)Cl (Ni-I) solution: The process is the same as the above. The final concentrations of photocatalyst was 0.01 mM, and the concentrations of Ni(dtbbpy)(*t*BuPhCO)Cl were 0.02 mM, 0.04 mM, 0.06 mM, 0.08 mM.

All solutions were excited at 405 nm and the fluorescence spectra was measured over the range of 420 – 700 nm at low speed, among which the emission intensity was recorded at 469 nm.

It was clearly found that the Ni(dtbbpy)(*t*BuPhCO)Cl is the only reagent which could quench the emission of Ir photocatalyst. Other reagents such as TMS_3SiH could not quench the emission of the photocatalyst.

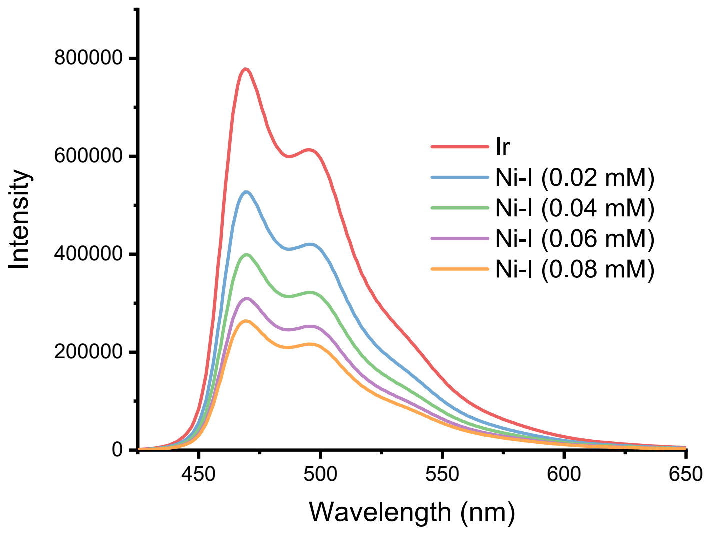

Figure S3. Quenching of the Ir[(dF(CF_3)ppy)]$_2$(dtbbpy)]PF_6 emission in the presence of increasing amount of **Ni-I**

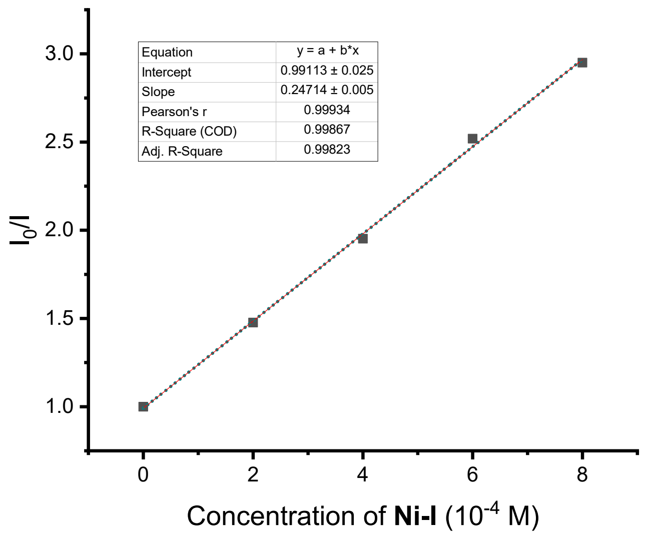

Figure S4. Stern-Volmer quenching plot with **Ni-I**

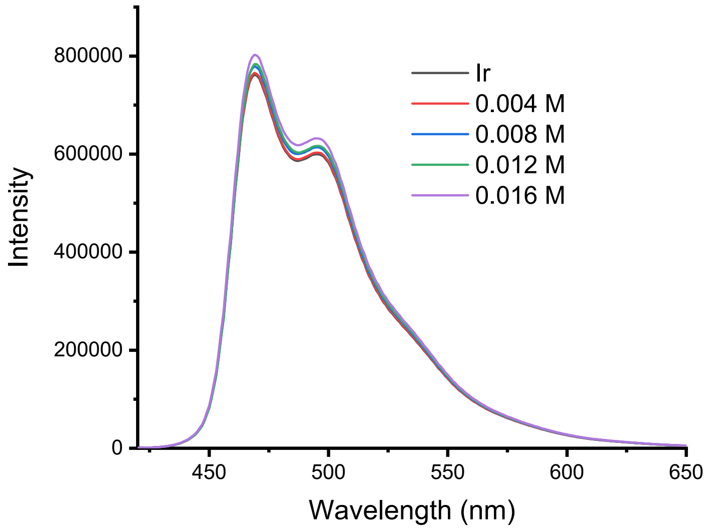

Figure S5. Quenching of the Ir[(dF(CF_3)ppy)]$_2$(dtbbpy)]PF_6 emission in the presence of increasing amount of **1a**

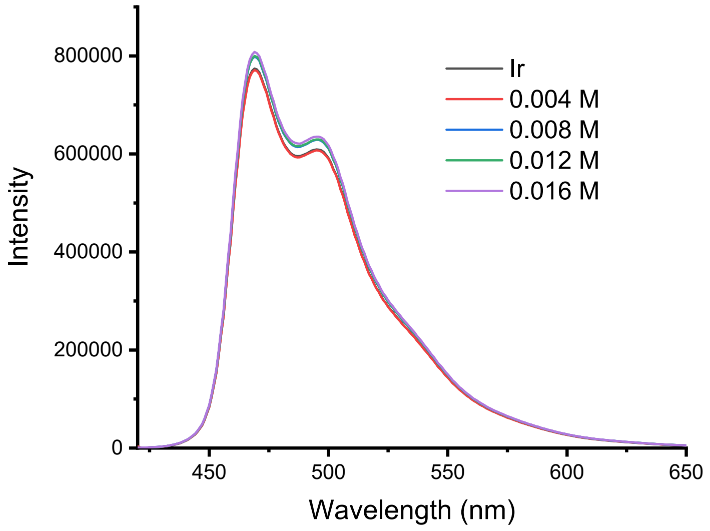

Figure S6. Quenching of the Ir[(dF(CF_3)ppy)]$_2$(dtbbpy)]PF_6 emission in the presence of increasing amount of **2a**

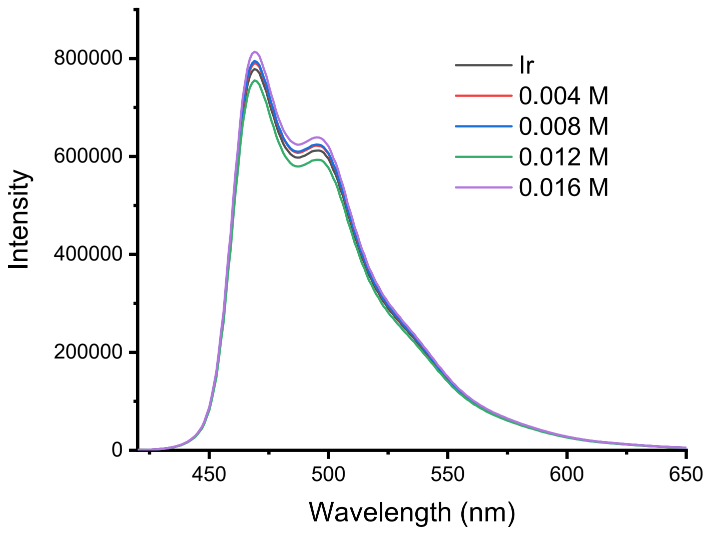

Figure S7. Quenching of the Ir[(dF(CF_3)ppy)]$_2$(dtbbpy)]PF_6 emission in the presence of increasing amount of **3a**

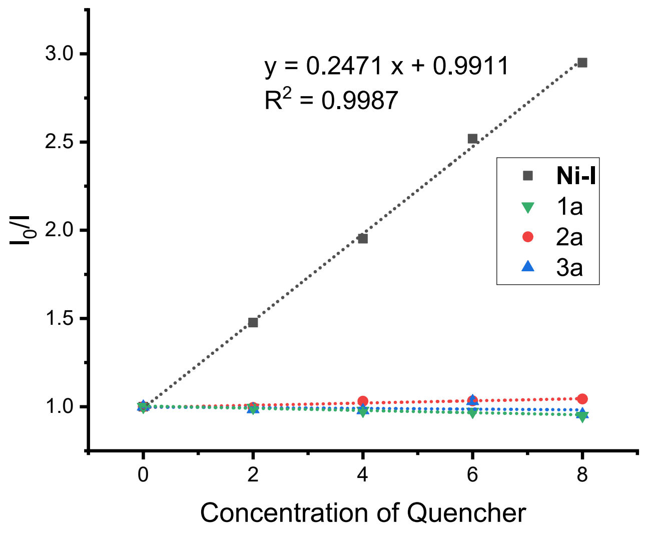

Figure S8. Stern-Volmer quenching plot with ^{t}BuPhCOCl (**1a**), ethyl acrylate (**2a**), TTMSS (**3a**), and **Ni-I**.

References

(1) Kan, S. B. J.; Lewis, R. D.; Chen, K.; Arnold, F. H. Directed Evolution of Cytochrome c for Carbon-Silicon Bond Formation: Bringing Silicon to Life. *Science* **2016**, *354*, 1048–1051.

(2) Liang, H.; Wang, L.-J.; Ji, Y.-X.; Wang, H.; Zhang, B. Selective Electrochemical Hydrolysis of Hydrosilanes to Silanols via Anodically Generated Silyl Cations. *Angew. Chem. Int. Ed.* **2021**, *60*, 1839–1844.

(3) He, P.; Zhang, F.; Si, X.; Jiang, W.; Shen, Q.; Li, Z.; Zhu, Z.; Tang, S.; Gui, Q.-W. Visible-Light-Induced Aerobic Oxidation of Tertiary Silanes to Silanols Using Molecular Oxygen as an Oxidant. *Synthesis* **2023**, *55*, 765–772.

(4) Li, S.; Li, H.; Tung, C.-H.; Liu, L. Practical and Selective Bio-Inspired Iron-Catalyzed Oxidation of Si–H Bonds to Diversely Functionalized Organosilanols. *ACS Catal.* **2022**, *12*, 9143–9152.

(5) García-Domínguez, A.; Mondal, R.; Nevado, C. Dual Photoredox/Nickel-Catalyzed Three-Component Carbofunctionalization of Alkenes. *Angew. Chem. Int. Ed.* **2019**, *58*, 12286–12290.

(6) Sun, Z.; Kumagai, N.; Shibasaki, M. Photocatalytic α-Acylation of Ethers. *Org. Lett.* **2017**, *19*, 3727–3730.

(7) Lee, G. S.; Won, J.; Choi, S.; Baik, M.-H.; Hong, S. H. Synergistic Activation of Amides and Hydrocarbons for Direct C(sp^3)–H Acylation Enabled by Metallaphotoredox Catalysis. *Angew. Chem. Int. Ed.* **2020**, *59*, 16933–16942.

VI. Compound Characterizations

Characterization of silylacylated products obtained in this study

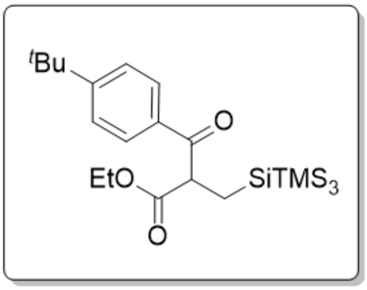

ethyl 3-(4-(*tert*-butyl)phenyl)-2-((1,1,1,3,3,3-hexamethyl-2-(trimethylsilyl)trisilan-2-yl)methyl)-3-oxopropanoate (4a). Yield 76%. 19.3 mg. Colorless oil. ^{1}H NMR (600 MHz, CD_2Cl_2) δ 7.89 (d, *J* = 8.6 Hz, 2H), 7.50 (d, *J* = 8.6 Hz, 2H), 4.32 (t, *J* = 6.7 Hz, 1H), 4.14 – 4.07 (m, 2H), 1.58 (dd, *J* = 14.7, 6.6 Hz, 1H), 1.39 (dd, *J* = 14.7, 6.8 Hz, 1H), 1.34 (s, 9H), 1.15 (t, *J* = 7.1 Hz, 3H), 0.18 (s, 27H). ^{13}C NMR (125 MHz, CD_2Cl_2) δ 194.9, 171.3, 157.7, 133.7, 128.9, 126.2, 61.7, 53.6, 35.5, 31.3, 14.2, 7.4, 1.3. HRMS (ESI$^+$) m/z calcd. $C_{25}H_{48}NaO_3Si_4^+$ [M+Na]$^+$: 531.2578, found: 531.2578.

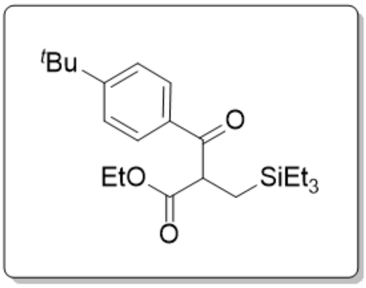

ethyl 3-(4-(*tert*-butyl)phenyl)-3-oxo-2-((triethylsilyl)methyl)propanoate (4b). Yield 58%. 10.9 mg. Yellow oil. ^{1}H NMR (600 MHz, CD_2Cl_2) δ 8.03 – 7.78 (m, 2H), 7.58 – 7.43 (m, 2H), 4.32 (t, *J* = 7.3 Hz, 1H), 4.10 (m, 2H), 1.37 – 1.30 (m, 9H), 1.24 (d, *J* = 7.2 Hz, 2H), 1.22 – 1.12 (m, 3H), 0.99 – 0.86 (m, 9H), 0.53 (m, 6H). ^{13}C NMR (125 MHz, CD_2Cl_2) δ 195.8, 171.6, 157.6, 133.7, 128.9, 126.1, 61.7, 50.1, 35.4, 31.2, 14.2, 11.6, 7.5, 3.8. HRMS (ESI$^+$) m/z calcd. $C_{22}H_{37}O_3Si^+$ [M+H]$^+$: 377.2512, found: 377.2513.

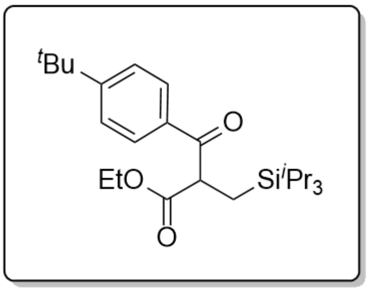

ethyl 3-(4-(*tert*-butyl)phenyl)-3-oxo-2-((triisopropylsilyl)methyl)propanoate (4c). Yield 54%. 11.3 mg. Yellow oil. ^{1}H NMR (600 MHz, CD_2Cl_2) δ 7.91 (d, *J* = 8.4 Hz, 2H), 7.50 (d, *J* = 8.2 Hz, 2H), 4.43 (t, *J* = 6.7 Hz, 1H), 4.18 – 4.02 (m, 2H), 1.42 (dd, *J* = 15.3, 6.3 Hz, 1H), 1.34 (s, 9H), 1.28 (dd, *J* = 15.4, 7.0 Hz, 1H), 1.15 (t, *J* = 7.1 Hz, 3H), 1.04 (m, 21H). ^{13}C NMR (125 MHz, CD_2Cl_2) δ 194.9, 171.3, 157.7, 133.7, 128.9, 126.2, 61.7, 53.6, 35.5, 31.3, 14.2, 7.4, 1.3. HRMS (ESI$^+$) m/z calcd. $C_{25}H_{43}O_3Si^+$ $[M+H]^+$: 419.2981, found: 419.2982.

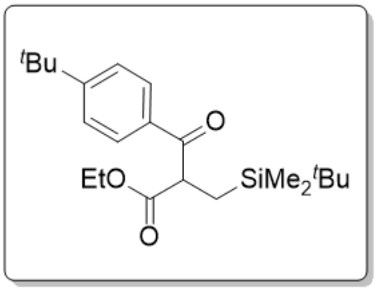

ethyl 3-(4-(*tert*-butyl)phenyl)-2-((*tert*-butyldimethylsilyl)methyl)-3-oxopropanoate (4d). Yield 60%. 11.4 mg. Yellow oil. ^{1}H NMR (600 MHz, CD_2Cl_2) δ 7.90 (d, *J* = 8.5 Hz, 2H), 7.51 (d, *J* = 8.5 Hz, 2H), 4.32 (m, 1H), 4.11 (m, 2H), 1.34 (s, 9H), 1.24 (m, 2H), 1.18 (t, *J* = 7.1 Hz, 3H), 0.89 (s, 9H), -0.04 (d, *J* = 15.2 Hz, 6H). ^{13}C NMR (125 MHz, CD_2Cl_2) δ 195.9, 171.5, 157.7, 133.7, 128.9, 126.1, 61.7, 50.3, 35.5, 31.2, 26.5, 16.9, 14.2, 12.32, -5.8, -5.9. HRMS (ESI$^+$) m/z calcd. $C_{22}H_{37}O_3Si^+$ $[M+H]^+$: 377.2512, found: 377.2513.

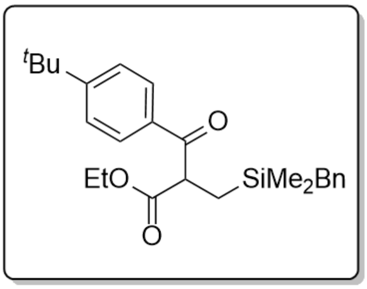

ethyl 2-((benzyldimethylsilyl)methyl)-3-(4-(*tert*-butyl)phenyl)-3-oxopropanoate (4e). Yield 58%. 11.9 mg. Colorless oil. ^{1}H NMR (600 MHz, CD_2Cl_2) δ 7.84 (d, J = 8.5 Hz, 2H), 7.49 (d, J = 8.6 Hz, 2H), 7.20 (t, J = 7.7 Hz, 2H), 7.10 – 7.04 (m, 1H), 7.04 – 6.97 (m, 2H), 4.26 (m, 1H), 4.10 (m, 2H), 2.12 (s, 2Hf), 1.35 (s, 9H), 1.24 (m, 2H), 1.17 (t, J = 7.1 Hz, 3H), -0.02 (m, 6H). ^{13}C NMR (125 MHz, CD_2Cl_2) δ 195.8, 171.4, 157.7, 140.2, 133.6, 128.9, 128.6, 128.5, 126.1, 124.5, 61.7, 50.0, 35.4, 31.18, 26.0, 14.8, 14.2, -3.0, -3.1. HRMS (ESI$^+$) m/z calcd. $C_{25}H_{35}O_3Si^+$ [M+H]$^+$: 411.2355, found: 411.2355.

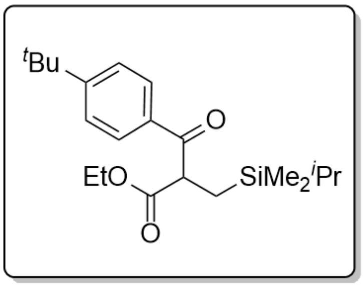

ethyl 3-(4-(*tert*-butyl)phenyl)-2-((isopropyldimethylsilyl)methyl)-3-oxopropanoate (4f). Yield 58%. 10.5 mg. Colorless oil. ^{1}H NMR (500 MHz, CD_2Cl_2) δ 7.90 (d, J = 8.2 Hz, 2H), 7.51 (d, J = 8.1 Hz, 2H), 4.31 (m, 1H), 4.10 (m, 2H), 1.34 (s, 9H), 1.22 (m, 2H), 1.17 (t, J = 7.1 Hz, 3H), 0.95 (m, 6H), 0.78 (m, 1H), -0.05 (m, 6H). ^{13}C NMR (125 MHz, CD_2Cl_2) δ 195.9, 171.5, 157.6, 133.7, 128.9, 126.1, 61.7, 50.2, 35.4, 31.2, 17.6, 14.2, 13.8, 13.4, -5.1, -5.2. HRMS (ESI$^+$) m/z calcd. $C_{21}H_{35}O_3Si^+$ [M+H]$^+$: 363.2355, found: 363.2356.

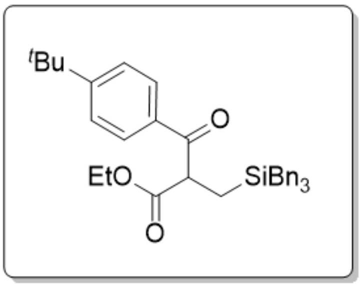

ethyl 3-(4-(*tert*-butyl)phenyl)-3-oxo-2-((tribenzylsilyl)methyl)propanoate (4g). Yield 57%. 16.0 mg. Colorless oil. ^{1}H NMR (600 MHz, CD_2Cl_2) δ 7.67 (d, *J* = 8.5 Hz, 2H), 7.45 (d, *J* = 8.6 Hz, 2H), 7.20 (t, *J* = 7.6 Hz, 6H), 7.10 (t, *J* = 7.5 Hz, 3H), 7.02 – 6.95 (m, 6H), 4.05 (m, 3H), 2.19 – 1.99 (m, 6H), 1.35 (s, 9H), 1.33 – 1.28 (m, 1H), 1.20 (m, 1H), 1.14 (t, *J* = 7.1 Hz, 3H). ^{13}C NMR (125 MHz, CD_2Cl_2) δ 195.5, 171.3, 157.7, 139.3, 133.6, 129.0, 128.9, 128.8, 126.0, 124.9, 61.8, 49.3, 35.4, 31.2, 22.2, 14.1, 11.8. HRMS (ESI$^+$) m/z calcd. $C_{37}H_{43}O_3Si^+$ [M+H]$^+$: 563.2981, found: 563.2984.

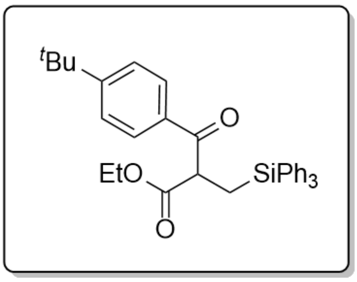

ethyl 3-(4-(*tert*-butyl)phenyl)-3-oxo-2-((triphenylsilyl)methyl)propanoate (4h). Yield 60%. 15.6 mg. Colorless oil. ^{1}H NMR (600 MHz, CD_2Cl_2) δ 7.64 (d, *J* = 8.1 Hz, 2H), 7.55 (m, 6H), 7.37 (m, 11H), 4.43 (m, 1H), 3.90 – 3.65 (m, 2H), 2.17 (dd, *J* = 15.3, 8.3 Hz, 1H), 2.07 (dd, *J* = 15.4, 5.9 Hz, 1H), 1.32 (s, 9H), 1.01 (t, *J* = 7.1 Hz, 3H). ^{13}C NMR (125 MHz, CD_2Cl_2) δ 195.4, 170.8, 157.5, 136.2, 134.4, 133.5, 130.1, 128.9, 128.3, 125.8, 61.7, 49.9, 35.4, 31.2, 13.9, 12.8. HRMS (ESI$^+$) m/z calcd. $C_{34}H_{36}O_3$ $NaSi^+$ [M+Na]$^+$: 543.2331, found: 543.2331.

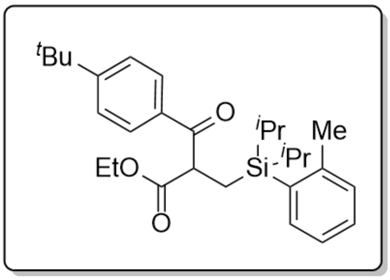

ethyl 3-(4-(*tert*-butyl)phenyl)-2-((diisopropyl(*o*-tolyl)silyl)methyl)-3-oxopropanoate (4i). Yield 52%. 12.1 mg. Colorless oil. ^{1}H NMR (600 MHz, CD_2Cl_2) δ 7.69 (d, *J* = 8.1 Hz, 2H), 7.40 (d, *J* = 8.2 Hz, 2H), 7.37 (d, *J* = 7.4 Hz, 1H), 7.20 (t, *J* = 7.5 Hz, 1H), 7.13 (d, *J* = 7.6 Hz, 1H), 7.03 (t, *J* = 7.4 Hz, 1H), 4.28 (t, *J* = 6.9 Hz, 1H), 3.90 (m, 2H), 2.40 (s, 3H), 1.68 (dd, *J* = 15.5, 6.7 Hz, 1Hf), 1.62 (dd, *J* = 15.4, 6.9 Hz, 1H), 1.41 (m, 2H), 1.32 (s, 9H), 1.12 – 1.01 (m, 15H). ^{13}C NMR (125 MHz, CD_2Cl_2) δ 195.5, 171.3, 157.4, 144.7, 136.5, 133.9, 133.6, 130.7, 129.5, 128.8, 125.9, 125.1, 61.7, 50.0, 35.4, 31.2, 23.9, 18.9, 18.8, 14.0, 12.9, 12.7, 10.8. HRMS (ESI$^+$) m/z calcd. $C_{29}H_{42}O_3NaSi^+$ [M+Na]$^+$: 489.2801, found: 489.2801.

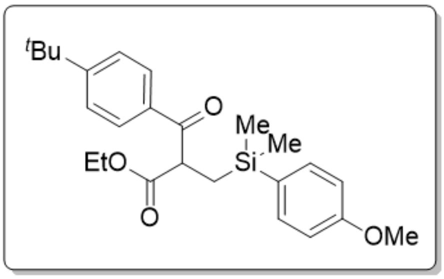

ethyl 3-(4-(*tert*-butyl)phenyl)-2-(((4-methoxyphenyl)dimethylsilyl)methyl)-3-oxopropanoate (4j). Yield 60%. 12.8 mg. Colorless oil. ^{1}H NMR (500 MHz, CD_2Cl_2) δ 7.84 – 7.72 (m, 2H), 7.50 – 7.34 (m, 4H), 6.93 – 6.83 (m, 2H), 4.23 (m, 1H), 4.01 (q, *J* = 7.1 Hz, 2H), 3.80 (s, 3H), 1.46 (dd, *J* = 14.8, 8.5 Hz, 1H), 1.33 (m, 10H), 1.13 (t, *J* = 7.1 Hz, 3H), 0.27 (m, 6H). ^{13}C NMR (125 MHz, CD_2Cl_2) δ 195.9, 171.3, 161.1, 157.5, 135.6, 133.5, 129.0, 128.9, 126.0, 113.9, 61.6, 55.4, 50.1, 35.4, 31.2, 16.2, 14.1, -2.5, -2.5. HRMS (ESI$^+$) m/z calcd. $C_{25}H_{34}O_4NaSi^+$ [M+Na]$^+$: 449.2124, found: 449.2124.

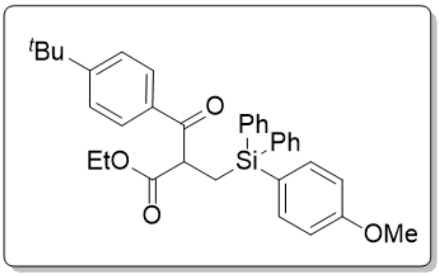

ethyl 3-(4-(*tert*-butyl)phenyl)-2-(((4-methoxyphenyl)diphenylsilyl)methyl)-3-oxopropanoate (4k). Yield 54%. 14.8 mg. Colorless oil. ^{1}H NMR (500 MHz, CD_2Cl_2) δ 7.64 (d, *J* = 8.2 Hz, 2H), 7.55 (d, *J* = 7.2 Hz, 4H), 7.46 (d, *J* = 8.1 Hz, 2H), 7.42 – 7.26 (m, 8H), 6.89 (d, *J* = 8.1 Hz, 2H), 4.43 (dd, *J* = 8.1, 6.0 Hz, 1H), 3.93 – 3.68 (m, 5H), 2.15 (dd, *J* = 15.2, 8.1 Hz, 1H), 2.06 (dd, *J* = 15.2, 6.0 Hz, 1H), 1.33 (s, 9H), 1.03 (t, *J* = 7.1 Hz, 3H). ^{13}C NMR (125 MHz, CD_2Cl_2) δ 195.4, 170.9, 161.5, 157.5, 137.7, 136.1, 134.9, 133.5, 130.0, 128.9, 128.3, 125.8, 124.8, 114.1, 61.7, 55.4, 49.9, 35.4, 31.2, 14.0, 13.1. HRMS (ESI$^+$) m/z calcd. $C_{35}H_{38}O_4NaSi^+$ $[M+Na]^+$: 573.2437, found: 573.2437.

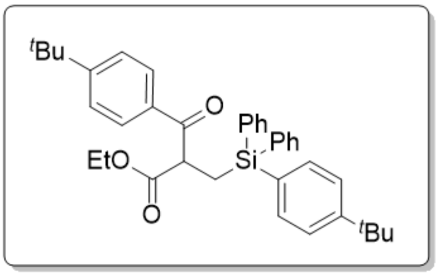

ethyl 3-(4-(*tert*-butyl)phenyl)-2-(((4-(*tert*-butyl)phenyl)diphenylsilyl)methyl)-3-oxopropanoate (4l). Yield 54%. 15.6 mg. Colorless oil. ^{1}H NMR (600 MHz, CD_2Cl_2) δ 7.62 (d, *J* = 8.3 Hz, 2H), 7.58 – 7.54 (m, 4H), 7.48 (d, *J* = 7.9 Hz, 2H), 7.38 (m, 10H), 4.43 (dd, *J* = 8.1, 6.0 Hz, 1H), 3.82 (m, 1H), 3.78 – 3.71 (m, 1H), 2.15 (dd, *J* = 15.3, 8.1 Hz, 1H), 2.07 (dd, *J* = 15.3, 6.0 Hz, 1H), 1.40 – 1.19 (m, 18H), 1.01 (t, *J* = 7.1 Hz, 3H). ^{13}C NMR (125 MHz, CD_2Cl_2) δ 195.4, 170.9, 157.4, 153.2, 136.1, 136.1, 134.7, 133.5, 130.6, 130.0, 128.9, 128.3, 125.8, 125.3, 61.7, 49.9, 35.4, 35.0, 31.3, 31.2, 14.0, 12.9. HRMS (ESI$^+$) m/z calcd. $C_{38}H_{44}O_3NaSi^+$ $[M+Na]^+$: 599.2957, found: 599.2956.

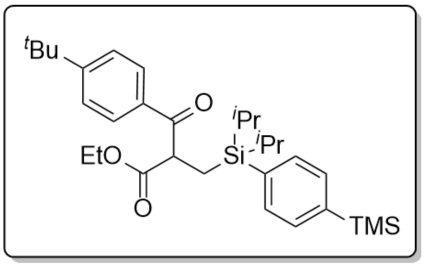

ethyl 3-(4-(*tert*-butyl)phenyl)-2-((diisopropyl(4-(trimethylsilyl)phenyl)silyl)methyl)-3-oxopropanoate (4m). Yield 42%. 11.0 mg. Colorless oil. ^{1}H NMR (500 MHz, CD_2Cl_2) δ 7.80 – 7.68 (m, 2H), 7.52 – 7.38 (m, 6H), 4.35 (t, J = 6.8 Hz, 1H), 3.96 (q, J = 7.1 Hz, 2H), 1.64 – 1.53 (m, 2H), 1.33 (m, 11H), 1.15 – 0.97 (m, 15H), 0.26 (s, 9H). ^{13}C NMR (125 MHz, CD_2Cl_2) δ 195.5, 171.4, 157.6, 141.6, 135.2, 134.7, 133.7, 132.9, 128.9, 126.0, 61.7, 50.0, 35.4, 31.2, 18.4, 18.3, 14.1, 11.7, 11.6, 9.8, -1.1. HRMS (ESI$^+$) m/z calcd. $C_{31}H_{48}O_3NaSi_2^+$ [M+Na]$^+$: 547.3040, found: 547.3040.

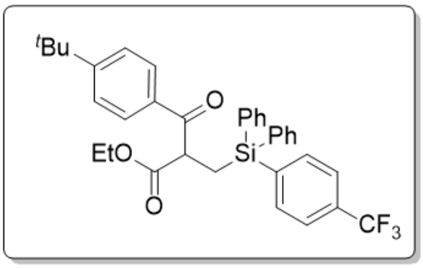

ethyl 3-(4-(*tert*-butyl)phenyl)-2-((diphenyl(4-(trifluoromethyl)phenyl)silyl)methyl)-3-oxopropanoate (4n). Yield 60%. 17.6 mg. Colorless oil. ^{1}H NMR (600 MHz, CD_2Cl_2) δ 7.68 (d, J = 7.7 Hz, 2H), 7.62 (d, J = 8.2 Hz, 2H), 7.58 – 7.52 (m, 6H), 7.46 – 7.34 (m, 8H), 4.43 (t, J = 7.1 Hz, 1H), 3.85 (m, 1H), 3.77 (m, 1H), 2.22 – 2.11 (m, 2H), 1.31 (s, 9H), 1.02 (t, J = 7.1 Hz, 3H). ^{13}C NMR (125 MHz, CD_2Cl_2) δ 195.1, 170.8, 157.7, 139.9, 136.6, 136.1, 133.5, 133.5, 130.4, 128.8, 128.5, 128.5, 125.9, 124.76 – 124.55 (m), 61.8, 49.7, 35.4, 31.1, 13.9, 12.6. ^{19}F NMR (376 MHz, CD_2Cl_2) δ -63.3. HRMS (ESI$^+$) m/z calcd. $C_{35}H_{35}O_3F_3NaSi^+$ [M+Na]$^+$: 611.2205, found: 611.2203.

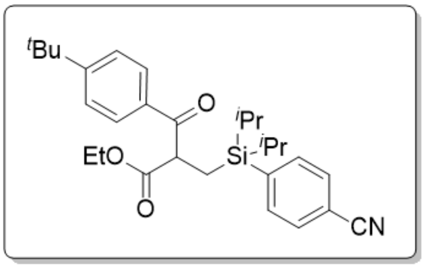

ethyl 3-(4-(*tert*-butyl)phenyl)-2-(((4-cyanophenyl)diisopropylsilyl)methyl)-3-oxopropanoate (4o). Yield 50%. 12.1 mg. Colorless oil. ^{1}H NMR (500 MHz, CD_2Cl_2) δ 7.76 (d, *J* = 8.1 Hz, 2H), 7.56 (m, 4H), 7.45 (d, *J* = 8.1 Hz, 2H), 4.33 (t, *J* = 6.9 Hz, 1H), 3.98 (q, *J* = 7.1 Hz, 2H), 1.69 (dd, *J* = 15.3, 7.0 Hz, 1H), 1.59 (dd, *J* = 15.4, 7.1 Hz, 1H), 1.41 – 1.25 (m, 11H), 1.15 – 0.96 (m, 15H). ^{13}C NMR (125 MHz, CD_2Cl_2) δ 195.0, 171.2, 157.8, 142.1, 135.9, 133.6, 131.1, 128.8, 126.0, 119.2, 113.0, 61.9, 49.7, 35.4, 31.2, 18.2, 18.2, 14.0, 11.5, 11.5, 9.4. HRMS (ESI$^+$) m/z calcd. $C_{29}H_{39}NO_3NaSi^+$ [M+Na]$^+$: 500.2597, found: 500.2597.

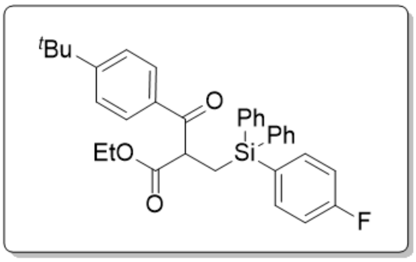

ethyl 3-(4-(*tert*-butyl)phenyl)-2-(((4-fluorophenyl)diphenylsilyl)methyl)-3-oxopropanoate (4p). Yield 60%. 16.2 mg. Colorless oil. ^{1}H NMR (600 MHz, CD_2Cl_2) δ 7.64 (d, *J* = 8.4 Hz, 2H), 7.56 – 7.49 (m, 6H), 7.44 – 7.32 (m, 8H), 7.07 – 7.00 (m, 2H), 4.43 (dd, *J* = 7.9, 6.3 Hz, 1H), 3.89 – 3.73 (m, 2H), 2.16 (dd, *J* = 15.3, 7.9 Hz, 1H), 2.09 (dd, *J* = 15.3, 6.3 Hz, 1H), 1.32 (s, 9H), 1.02 (t, *J* = 7.1 Hz, 3H). ^{13}C NMR (125 MHz, CD_2Cl_2) δ 195.3, 170.8, 164.5 (d, *J* = 248.9 Hz), 157.6, 138.4, 138.3, 136.1, 134.2 (d, *J* = 4.8 Hz), 133.5, 130.2, 130.0 (d, *J* = 3.7 Hz), 128.9, 128.4, 125.8, 115.4 (d, *J* = 19.8 Hz), 61.8, 49.8, 35.4, 31.2, 14.0, 12.9. ^{19}F NMR (471 MHz, CD_2Cl_2) δ -111.6. HRMS (ESI$^+$) m/z calcd. $C_{34}H_{35}O_3F$ $NaSi^+$ [M+Na]$^+$: 561.2237, found: 561.2236.

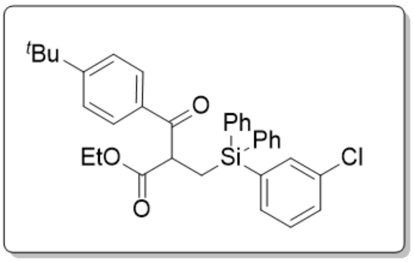

ethyl 3-(4-(*tert*-butyl)phenyl)-2-(((3-chlorophenyl)diphenylsilyl)methyl)-3-oxopropanoate (4q). Yield 51%. 14.2 mg. Colorless oil. ^{1}H NMR (600 MHz, CD_2Cl_2) δ 7.67 – 7.62 (m, 2H), 7.54 (m, 4H), 7.49 (m, 1H), 7.45 – 7.33 (m, 10H), 7.29 (m, 1H), 4.43 (dd, J = 7.9, 6.2 Hz, 1H), 3.90 – 3.74 (m, 2H), 2.17 (dd, J = 15.5, 8.0 Hz, 1H), 2.10 (dd, J = 15.3, 6.1 Hz, 1H), 1.32 (s, 9H), 1.03 (m, 3H). ^{13}C NMR (125 MHz, CD_2Cl_2) δ 195.2, 170.8, 157.6, 137.5, 136.1, 135.6, 134.7, 134.3, 133.6, 133.5, 130.3, 130.1, 129.8, 128.9, 128.4, 125.9, 61.8, 49.7, 35.4, 31.2, 14.0, 12.7. HRMS (ESI$^+$) m/z calcd. $C_{34}H_{35}O_3NaSi$ Cl^+ $[M+Na]^+$: 577.1942, found: 577.1942.

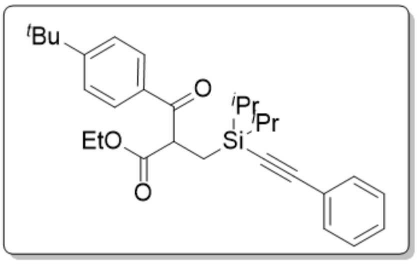

ethyl 3-(4-(*tert*-butyl)phenyl)-2-((diisopropyl(phenylethynyl)silyl)methyl)-3-oxopropanoate (4r). Yield 50%. 11.8 mg. Colorless oil. ^{1}H NMR (500 MHz, CD_2Cl_2) δ 7.95 (d, J = 8.1 Hz, 2H), 7.43 (d, J = 8.1 Hz, 2H), 7.32 – 7.11 (m, 5H), 4.64 (t, J = 7.1 Hz, 1H), 4.09 (m, 2H), 1.45 (dd, J = 15.0, 7.3 Hz, 1H), 1.31 (m, 10H), 1.12 (m, 17H). ^{13}C NMR (125 MHz, CD_2Cl_2) δ 195.5, 171.4, 157.5, 134.7, 132.9, 132.2, 129.1, 128.5, 126.0, 123.2, 108.9, 89.8, 61.7, 50.5, 35.4, 31.2, 18.2, 18.1, 14.1, 12.7, 9.4, -1.1. HRMS (ESI$^+$) m/z calcd. $C_{30}H_{40}O_3NaSi^+$ $[M+Na]^+$: 499.2644, found: 499.2645.

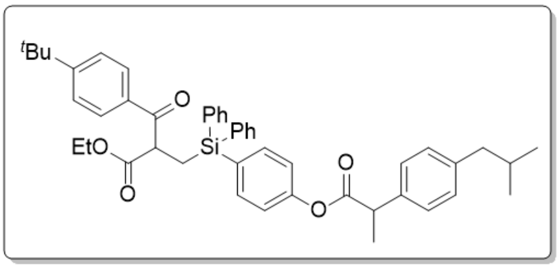

ethyl 3-(4-(*tert*-butyl)phenyl)-2-(((4-((2-(4-isobutylphenyl)propanoyl)oxy)phenyl)diphenylsilyl) methyl)-3-oxopropanoate (4s). Yield 51%. 18.5 mg. Colorless oil. ^{1}H NMR (600 MHz, CD_2Cl_2) δ 7.64 – 7.58 (m, 2H), 7.57 – 7.49 (m, 6H), 7.42 – 7.32 (m, 8H), 7.30 (d, *J* = 7.9 Hz, 2H), 7.16 (d, *J* = 7.9 Hz, 2H), 6.99 (d, *J* = 8.0 Hz, 2H), 4.42 (dd, *J* = 8.2, 5.9 Hz, 1H), 3.95 (q, *J* = 7.1 Hz, 1H), 3.82 (m, 1H), 3.78 – 3.70 (m, 1H), 2.48 (d, *J* = 7.2 Hz, 2H), 2.16 (dd, *J* = 15.3, 8.3 Hz, 1H), 2.06 (dd, *J* = 15.3, 5.9 Hz, 1H), 1.87 (m, 1H), 1.59 (d, *J* = 7.2 Hz, 3H), 1.32 (s, 9H), 1.01 (t, *J* = 7.1 Hz, 3H), 0.91 (d, *J* = 6.6 Hz, 6H). ^{13}C NMR (125 MHz, CD_2Cl_2) δ 195.4, 173.4, 170.8, 157.5, 152.8, 141.3, 137.8, 137.4, 136.1, 134.2, 133.5, 131.9, 130.2, 129.9, 128.9, 128.4, 127.6, 125.9, 121.5, 61.7, 49.8, 45.6, 45.3, 35.4, 31.2, 30.6, 22.5, 18.8, 13.9, 12.9. HRMS (ESI$^+$) m/z calcd. $C_{47}H_{52}O_5NaSi^+$ $[M+Na]^+$: 747.3482, found: 747.3483.

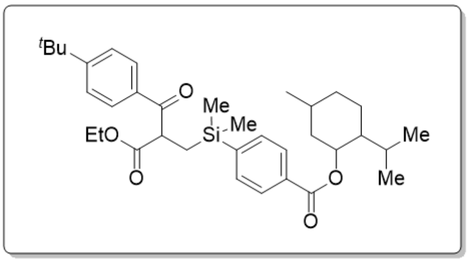

2-isopropyl-5-methylcyclohexyl 4-((2-(4-(*tert*-butyl)benzoyl)-3-ethoxy-3-oxopropyl)dimethylsilyl) benzoate (4t). Yield 57%. 16.5 mg. Colorless oil. ^{1}H NMR (600 MHz, CD_2Cl_2) δ 7.99 – 7.94 (m, 2H), 7.73 (t, *J* = 8.2 Hz, 2H), 7.58 (d, *J* = 7.7 Hz, 2H), 7.43 (dd, *J* = 8.5, 2.5 Hz, 2H), 4.92 (td, *J* = 10.9, 4.4 Hz, 1H), 4.24 (m, 1H), 4.05 – 3.93 (m, 2H), 2.11 (m, 1H), 2.03 – 1.90 (m, 1H), 1.78 – 1.71 (m, 2H), 1.62 – 1.53 (m, 2H), 1.51 (m, 1H), 1.43 (m, 1H), 1.33 (s, 9H), 1.20 – 1.11 (m, 5H), 0.99 – 0.90 (m, 7H), 0.79 (m, 3H), 0.35 – 0.30 (m, 6H). ^{13}C NMR (125 MHz, CD_2Cl_2) δ 195.7, 171.2, 166.3, 157.7, 144.5, 134.1, 133.5, 131.9, 128.8, 128.8, 126.0, 75.2, 61.7, 50.0, 47.7, 41.4, 35.4, 34.7, 31.9, 31.2, 27.0, 24.1,

22.2, 20.9, 16.7, 15.7, 14.1, -2.7. HRMS (ESI$^+$) m/z calcd. $C_{35}H_{50}O_5NaSi^+$ [M+Na]$^+$: 601.3325, found: 601.3325.

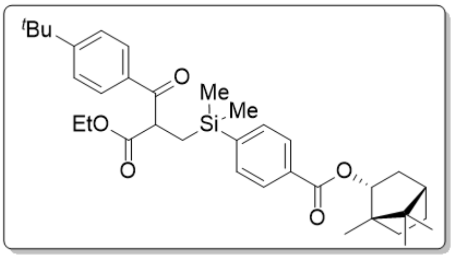

(1*S*,2*R*,4*S*)-1,7,7-trimethylbicyclo[2.2.1]heptan-2-yl 4-((2-(4-(*tert*-butyl)benzoyl)-3-ethoxy-3-oxopropyl)dimethylsilyl)benzoate (4u). Yield 60%. 17.2 mg. Colorless oil. ^{1}H NMR (500 MHz, CD_2Cl_2) δ 7.99 (d, *J* = 7.6 Hz, 2H), 7.74 (d, *J* = 8.1 Hz, 2H), 7.59 (d, *J* = 7.6 Hz, 2H), 7.44 (d, *J* = 8.1 Hz, 2H), 5.21 – 5.01 (m, 1H), 4.25 (t, *J* = 7.4 Hz, 1H), 4.00 (q, *J* = 7.1 Hz, 2H), 2.46 (m, 1H), 2.15 (m, 1H), 1.82 (m, 1H), 1.74 (m, 1H), 1.55 – 1.22 (m, 14H), 1.15 – 1.09 (m, 3H), 0.98 (s, 3H), 0.92 (m, 6H), 0.33 (m, 6H). ^{13}C NMR (125 MHz, CD_2Cl_2) δ 195.7, 171.2, 167.0, 157.7, 144.6, 134.1, 133.5, 131.9, 128.9, 128.8, 126.0, 80.9, 61.7, 50.0, 49.4, 48.2, 45.5, 37.3, 35.4, 31.2, 28.4, 27.7, 19.9, 19.1, 15.7, 14.1, 13.8, -2.7. HRMS (ESI$^+$) m/z calcd. $C_{35}H_{49}O_5Si^+$ [M+H]$^+$: 577.3349, found: 577.3348.

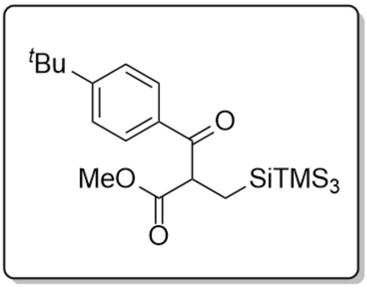

methyl 3-(4-(*tert*-butyl)phenyl)-2-((1,1,1,3,3,3-hexamethyl-2-(trimethylsilyl)trisilan-2-yl)methyl)-3-oxopropanoate (4aa). Yield 77%. 19.1 mg. Colorless oil. ^{1}H NMR (600 MHz, CD_2Cl_2) δ 7.94 – 7.81 (m, 2H), 7.58 – 7.44 (m, 2H), 4.42 – 4.28 (m, 1H), 3.65 (s, 3H), 1.59 – 1.52 (m, 1H), 1.42 (m, 1H), 1.34 (s, 9H), 0.17 (s, 27H). ^{13}C NMR (125 MHz, CD_2Cl_2) δ 194.8, 171.7, 157.8, 133.5, 128.9, 126.2, 53.3, 52.8, 35.5, 31.2, 7.6, 1.3. HRMS (ESI$^+$) m/z calcd. $C_{24}H_{46}O_3NaSi_4^+$ [M+Na]$^+$: 517.2422, found: 517.2422.

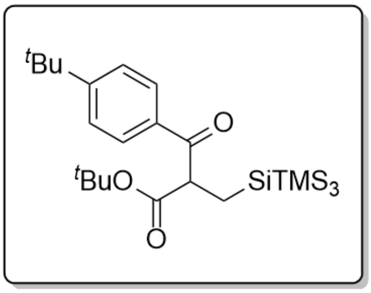

***tert*-butyl 3-(4-(*tert*-butyl)phenyl)-2-((1,1,1,3,3,3-hexamethyl-2-(trimethylsilyl)trisilan-2-yl) methyl)-3-oxopropanoate (4ab).** Yield 66%. 17.7 mg. Colorless oil. ^{1}H NMR (600 MHz, CD_2Cl_2) δ 7.87 (m, 2H), 7.49 (m, 2H), 4.19 (m, 1H), 1.61 (m, 1H), 1.34 (m, 9H), 1.32 (m, 9H), 1.31 – 1.28 (m, 1H), 0.17 (m, 27H). ^{13}C NMR (125 MHz, CD_2Cl_2) δ 195.0, 170.7, 157.5, 134.1, 128.8, 126.0, 81.9, 54.6, 35.4, 31.2, 27.9, 6.6, 1.2. HRMS (ESI$^+$) m/z calcd. $C_{27}H_{52}O_3NaSi_4^+$ [M+Na]$^+$: 559.2891, found: 559.2891.

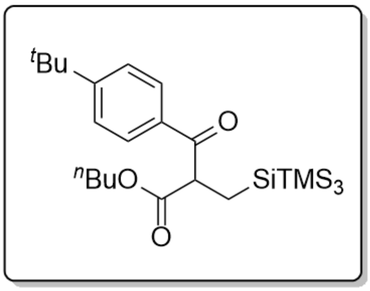

butyl 3-(4-(*tert*-butyl)phenyl)-2-((1,1,1,3,3,3-hexamethyl-2-(trimethylsilyl)trisilan-2-yl)methyl)-3-oxopropanoate (4ac). Yield 61%. 16.3 mg. Colorless oil. ^{1}H NMR (600 MHz, CD_2Cl_2) δ 7.89 (m, 2H), 7.50 (m, 2H), 4.32 (m, 1H), 4.04 (m, 2H), 1.60 (m, 1H), 1.53 – 1.45 (m, 2H), 1.39 (m, 1H), 1.34 (s, 9H), 1.25 – 1.16 (m, 2H), 0.82 (m, 3H), 0.18 (s, 27H). ^{13}C NMR (125 MHz, CD_2Cl_2) δ 194.8, 171.4, 157.7, 133.8, 128.9, 126.1, 65.5, 53.6, 35.5, 31.2, 30.9, 19.4, 13.8, 7.2, 1.3. HRMS (ESI$^+$) m/z calcd. $C_{27}H_{52}O_3NaSi_4^+$ [M+Na]$^+$: 559.2891, found: 559.2890.

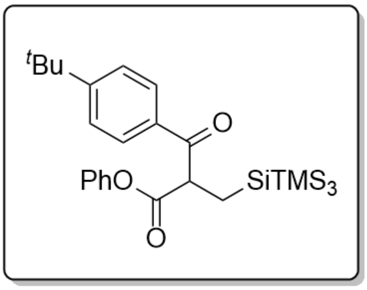

phenyl 3-(4-(*tert*-butyl)phenyl)-2-((1,1,1,3,3,3-hexamethyl-2-(trimethylsilyl)trisilan-2-yl)methyl)-3-oxopropanoate (4ad). Yield 57%. 15.9 mg. Colorless oil. ^{1}H NMR (600 MHz, CD_2Cl_2) δ 7.97 (d, *J* = 8.2 Hz, 2H), 7.56 (d, *J* = 8.3 Hz, 2H), 7.35 (t, *J* = 7.8 Hz, 2H), 7.25 – 7.19 (m, 1H), 6.96 (d, *J* = 8.7 Hz, 2H), 4.57 – 4.54 (m, 1H), 1.72 – 1.61 (m, 1H), 1.56 – 1.49 (m, 1H), 1.36 (s, 9H), 0.22 (s, 27H). ^{13}C NMR (125 MHz, CD_2Cl_2) δ 194.8, 170.2, 158.1, 151.2, 133.4, 129.8, 129.0, 126.4, 126.3, 121.7, 53.6, 35.5, 31.2, 7.5, 1.3. HRMS (ESI$^+$) m/z calcd. $C_{29}H_{48}O_3NaSi_4^+$ [M+Na]$^+$: 579.2578, found: 579.2579.

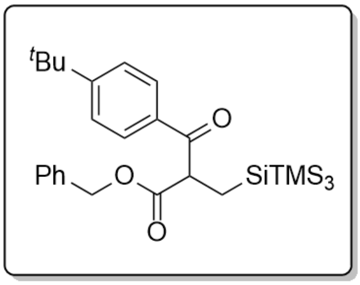

benzyl 3-(4-(*tert*-butyl)phenyl)-2-((1,1,1,3,3,3-hexamethyl-2-(trimethylsilyl)trisilan-2-yl)methyl)-3-oxopropanoate (4ae). Yield 79%. 22.5 mg. Colorless oil. ^{1}H NMR (600 MHz, CD_2Cl_2) δ 7.85 (d, *J* = 8.5 Hz, 2H), 7.45 (d, *J* = 8.5 Hz, 2H), 7.27 (m, 3H), 7.17 (m, 2H), 5.08 (d, *J* = 4.4 Hz, 2H), 4.36 (t, *J* = 6.7 Hz, 1H), 1.62 (dd, *J* = 14.7, 6.9 Hz, 1H), 1.40 (dd, *J* = 14.7, 6.4 Hz, 1H), 1.34 (s, 9H), 0.17 (s, 27H). ^{13}C NMR (125 MHz, CD_2Cl_2) δ 194.5, 171.1, 157.8, 136.0, 133.6, 128.9, 128.8, 128.5, 128.4, 126.1, 67.3, 53.5, 35.4, 31.2, 7.3, 1.3. HRMS (ESI$^+$) m/z calcd. $C_{30}H_{50}O_3NaSi_4^+$ [M+Na]$^+$: 593.2735, found: 593.2736.

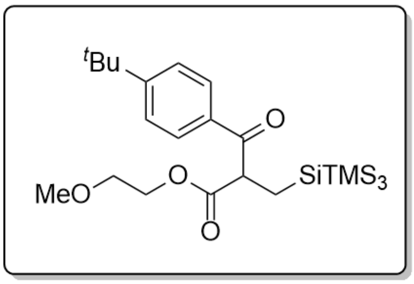

2-methoxyethyl 3-(4-(*tert*-butyl)phenyl)-2-((1,1,1,3,3,3-hexamethyl-2-(trimethylsilyl)trisilan-2-yl) methyl)-3-oxopropanoate (4af). Yield 79%. 21.2 mg. Colorless oil. ^{1}H NMR (600 MHz, CD_2Cl_2) δ 7.90 (d, *J* = 8.6 Hz, 2H), 7.50 (d, *J* = 8.6 Hz, 2H), 4.35 (t, *J* = 6.6 Hz, 1H), 4.19 (m, 2H), 3.49 – 3.41 (m, 2H), 3.21 (s, 3H), 1.61 (dd, *J* = 14.7, 6.9 Hz, 1H), 1.40 (dd, *J* = 14.7, 6.4 Hz, 1H), 1.34 (s, 9H), 0.18 (s, 27H). ^{13}C NMR (125 MHz, CD_2Cl_2) δ 194.5, 171.4, 157.7, 133.6, 128.9, 126.1, 70.5, 64.7, 59.0, 53.5, 35.5, 31.2, 7.3, 1.3. HRMS (ESI$^+$) m/z calcd. $C_{26}H_{50}O_4NaSi_4^+$ [M+Na]$^+$: 561.2684, found: 561.2683.

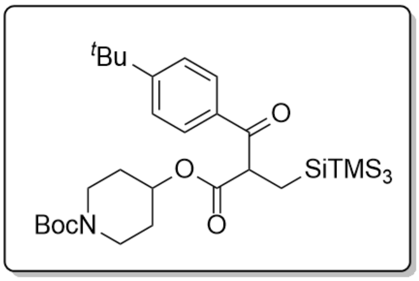

***tert*-butyl 4-((3-(4-(*tert*-butyl)phenyl)-2-((1,1,1,3,3,3-hexamethyl-2-(trimethylsilyl)trisilan-2-yl) methyl)-3-oxopropanoyl)oxy)piperidine-1-carboxylate (4ag).** Yield 76%. 25.2 mg. Colorless oil. ^{1}H NMR (600 MHz, CD_2Cl_2) δ 7.87 (d, *J* = 8.5 Hz, 2H), 7.50 (d, *J* = 8.5 Hz, 2H), 4.90 (m, 1H), 4.32 (t, *J* = 6.6 Hz, 1H), 3.37 – 3.25 (m, 2H), 3.25 – 3.14 (m, 2H), 1.68 (m, 2H), 1.58 (m, 2H), 1.52 – 1.42 (m, 2H), 1.40 (s, 9H), 1.34 (s, 9H), 0.18 (s, 27H). ^{13}C NMR (125 MHz, CD_2Cl_2) δ 194.8, 170.7, 157.8, 154.8, 133.8, 128.9, 126.2, 79.6, 75.5, 70.9, 35.5, 31.2, 30.6, 30.4, 28.5, 7.0, 1.3. HRMS (ESI$^+$) m/z calcd. $C_{33}H_{61}NO_5NaSi_4^+$ [M+Na]$^+$: 686.3524, found: 686.3525.

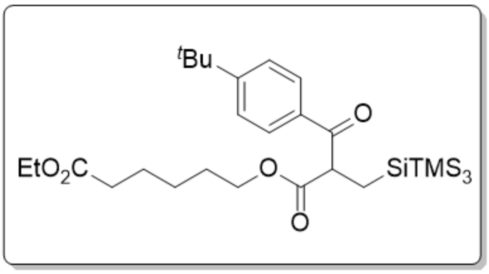

ethyl 6-((3-(4-(*tert*-butyl)phenyl)-2-((1,1,1,3,3,3-hexamethyl-2-(trimethylsilyl)trisilan-2-yl)methyl)-3-oxopropanoyl)oxy)hexanoate (4ah). Yield 79%. 24.6 mg. Colorless oil. ^1H NMR (600 MHz, CD_2Cl_2) δ 7.89 (d, *J* = 8.5 Hz, 2H), 7.50 (d, *J* = 8.6 Hz, 2H), 4.32 (t, *J* = 6.7 Hz, 1H), 4.16 – 3.99 (m, 4H), 2.18 (t, *J* = 7.6 Hz, 2H), 1.62 – 1.55 (m, 1H), 1.52 (m, 4H), 1.39 (m, 1H), 1.34 (s, 9H), 1.22 (m, 5H), 0.17 (s, 27H). ^{13}C NMR (125 MHz, CD_2Cl_2) δ 194.7, 173.6, 171.3, 157.7, 133.7, 128.9, 126.1, 65.5, 60.5, 53.5, 35.5, 34.4, 31.2, 28.5, 25.7, 24.8, 14.5, 7.3, 1.3. HRMS (ESI^+) m/z calcd. $C_{31}H_{58}O_5NaSi_4^+$ $[M+Na]^+$: 645.3259, found: 645.3259.

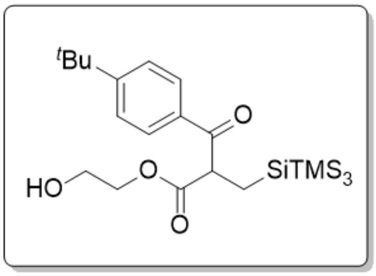

2-hydroxyethyl 3-(4-(*tert*-butyl)phenyl)-2-((1,1,1,3,3,3-hexamethyl-2-(trimethylsilyl)trisilan-2-yl)methyl)-3-oxopropanoate (4ai). Yield 71%. 18.6 mg. Colorless oil. ^1H NMR (500 MHz, CD_2Cl_2) δ 7.89 (d, *J* = 8.5 Hz, 2H), 7.52 (d, *J* = 8.4 Hz, 2H), 4.37 (dd, *J* = 7.9, 5.8 Hz, 1H), 4.25 (m, 1H), 4.14 (m, 1H), 3.85 – 3.44 (m, 2H), 1.91 (s, 1H), 1.52 (dd, *J* = 14.7, 5.8 Hz, 1H), 1.45 (dd, *J* = 14.6, 7.8 Hz, 1H), 1.34 (s, 9H), 0.19 (s, 27H). ^{13}C NMR (125 MHz, CD_2Cl_2) δ 195.8, 171.4, 158.1, 133.3, 129.0, 126.3, 67.2, 61.2, 53.7, 35.5, 31.2, 7.6, 1.3. HRMS (ESI^+) m/z calcd. $C_{25}H_{48}O_4NaSi_4^+$ $[M+Na]^+$: 547.2527, found: 547.2527.

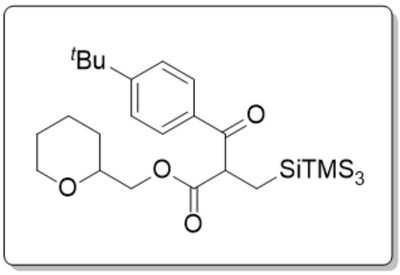

(tetrahydro-2*H*-pyran-2-yl)methyl 3-(4-(*tert*-butyl)phenyl)-2-((1,1,1,3,3,3-hexamethyl-2-(trimethylsilyl)trisilan-2-yl)methyl)-3-oxopropanoate (4aj). Yield 75%. 21.7 mg. Colorless oil. ^{1}H NMR (600 MHz, CD_2Cl_2) δ 7.95 – 7.84 (m, 2H), 7.55 – 7.45 (m, 2H), 4.48 – 4.24 (m, 1H), 4.01 (m, 2H), 3.93 – 3.72 (m, 1H), 3.34 (m, 1H), 3.25 (m, 1H), 1.78 – 1.72 (m, 1H), 1.63 (m, 1H), 1.44 – 1.35 (m, 5H), 1.34 (s, 9H), 1.15 (m, 1H), 0.17 (s, 27H). ^{13}C NMR (125 MHz, CD_2Cl_2) δ 194.5, 171.3, 157.7, 133.7, 129.0, 126.1, 75.6, 68.5, 68.2, 53.5, 35.4, 31.2, 28.2, 26.2, 23.3, 7.2, 1.3. HRMS (ESI$^+$) m/z calcd. $C_{29}H_{55}O_4Si_4^+$ $[M+H]^+$: 579.3177, found: 579.3177.

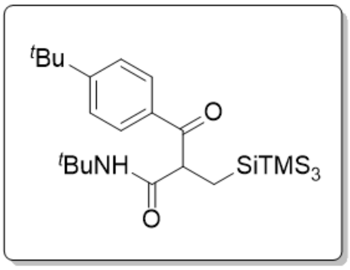

***N*-(*tert*-butyl)-3-(4-(*tert*-butyl)phenyl)-2-((1,1,1,3,3,3-hexamethyl-2-(trimethylsilyl)trisilan-2-yl)methyl)-3-oxopropanamide (4ak).** Yield 54%. 14.5 mg. Colorless oil. ^{1}H NMR (600 MHz, CD_2Cl_2) δ 8.14 – 7.81 (m, 2H), 7.64 – 7.32 (m, 2H), 5.35 (s, 1H), 4.14 (dd, J = 8.8, 4.1 Hz, 1H), 1.69 (dd, J = 14.4, 8.8 Hz, 1H), 1.34 (s, 9H), 1.33 – 1.29 (m, 1H), 1.20 (s, 9H), 0.15 (s, 27H). ^{13}C NMR (125 MHz, CD_2Cl_2) δ 197.9, 169.5, 157.7, 134.1, 129.1, 126.0, 58.0, 51.6, 35.5, 31.2, 28.6, 8.5, 1.2. HRMS (ESI$^+$) m/z calcd. $C_{27}H_{54}NO_2Si_4{}^+$ $[M+H]^+$: 536.3232, found: 536.3233.

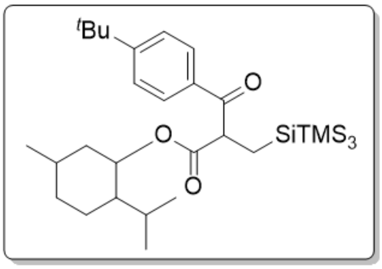

2-isopropyl-5-methylcyclohexyl 3-(4-(*tert*-butyl)phenyl)-2-((1,1,1,3,3,3-hexamethyl-2-(trimethyl silyl)trisilan-2-yl)methyl)-3-oxopropanoate (4al). Yield 51%. 15.8 mg. ^{1}H NMR (600 MHz, CD_2Cl_2) δ 7.89 – 7.82 (m, 2H), 7.53 – 7.41 (m, 2H), 4.55 (td, *J* = 10.7, 4.2 Hz, 1H), 4.32 – 4.21 (m, 1H), 1.83 (m, 1H), 1.80 – 1.70 (m, 1H), 1.66 – 1.57 (m, 3H), 1.50 – 1.39 (m, 2H), 1.34 (s, 9H), 1.24 (m, 1H), 1.03 – 0.77 (m, 6H), 0.75 – 0.69 (m, 3H), 0.51 (m, 3H), 0.17 (s, 27H). ^{13}C NMR (125 MHz, CD_2Cl_2) δ 193.6, 170.7, 157.6, 134.0, 128.7, 126.1, 74.9, 53.9, 47.3, 40.5, 34.5, 31.7, 31.2, 25.9, 23.2, 22.1, 20.9, 15.8, 6.7, 1.2.HRMS (ESI^{+}) m/z calcd. $C_{33}H_{62}O_3NaSi_4^+$ $[M+Na]^+$: 641.3674, found: 641.3675.

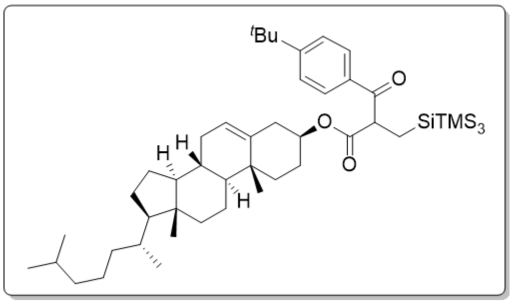

10,13-dimethyl-17-(6-methylheptan-2-yl)-2,3,4,7,8,9,10,11,12,13,14,15,16,17-tetradecahydro-1*H*-cyclopenta[a]phenanthren-3-yl 3-(4-(*tert*-butyl)phenyl)-2-((1,1,1,3,3,3-hexamethyl-2-(trimethyl silyl)trisilan-2-yl)methyl)-3-oxopropanoate (4am). Yield 54%. 22.9 mg. White solid. ^{1}H NMR (500 MHz, CD_2Cl_2) δ 7.87 (d, *J* = 8.1 Hz, 2H), 7.50 (d, *J* = 8.1 Hz, 2H), 5.33 (m, 1H), 4.54 (m, 1H), 4.27 (t, *J* = 6.5 Hz, 1H), 2.19 (m, 2H), 2.05 – 1.89 (m, 2H), 1.89 – 1.75 (m, 2H), 1.75 – 1.65 (m, 1H), 1.65 – 1.40 (m, 9H), 1.34 (m, 15H), 1.13 (m, 10H), 0.91 (m, 4H), 0.86 (m, 6H), 0.67z (s, 3H), 0.17 (s, 27H). ^{13}C NMR (125 MHz, CD_2Cl_2) δ 194.5, 170.4, 157.2, 139.6, 139.5, 133.4, 128.5, 125.7, 122.6, 74.9, 56.7, 56.1, 50.0, 42.3, 39.7, 39.5, 37.8, 37.6, 36.9, 36.5, 36.2, 35.8, 35.0, 31.9, 30.8, 28.2, 28.0, 27.5, 27.3, 24.2, 23.8, 22.5, 22.3, 21.0, 19.0, 18.5, 11.6, 6.6, 0.9. HRMS (ESI^{+}) m/z calcd. $C_{50}H_{88}O_3NaSi_4^+$ $[M+Na]^+$: 871.5708, found: 871.5707.

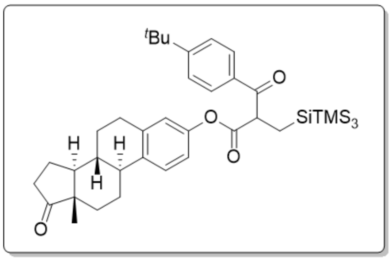

13-methyl-17-oxo-7,8,9,11,12,13,14,15,16,17-decahydro-6*H*-cyclopenta[*a*]phenanthren-3-yl 3-(4-(*tert*-butyl)phenyl)-2-((1,1,1,3,3,3-hexamethyl-2-(trimethylsilyl)trisilan-2-yl)methyl)-3-oxopropanoate (4an). Yield 60%. 21.9 mg. Colorless oil. ^{1}H NMR (500 MHz, CD_2Cl_2) δ 7.96 (d, *J* = 8.1 Hz, 2H), 7.55 (d, *J* = 8.1 Hz, 2H), 7.25 (d, *J* = 8.5 Hz, 1H), 6.72 (dd, *J* = 8.5, 2.5 Hz, 1H), 6.65 (d, *J* = 2.6 Hz, 1H), 4.52 (t, *J* = 6.7 Hz, 1H), 2.86 (m, 2H), 2.45 (m, 1H), 2.37 (m, 1H), 2.27 (m, 1H), 2.16 – 1.95 (m, 3H), 1.90 (m, 1H), 1.71 – 1.39 (m, 8H), 1.36 (s, 9H), 0.88 (s, 3H), 0.21 (s, 27H). ^{13}C NMR (125 MHz, CD_2Cl_2) δ 194.8, 170.4, 158.1, 149.0, 138.7, 138.3, 133.4, 129.0, 126.7, 126.3, 121.6, 121.6, 118.7, 53.6, 50.8, 48.2, 44.6, 38.4, 36.2, 35.5, 32.0, 31.2, 29.8, 26.7, 26.2, 21.9, 14.1, 7.4, 1.3. HRMS (ESI$^+$) m/z calcd. $C_{41}H_{64}O_4NaSi_4^+$ $[M+Na]^+$: 755.3779, found: 755.3779.

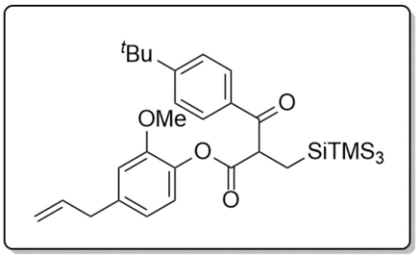

4-allyl-2-methoxyphenyl 3-(4-(*tert*-butyl)phenyl)-2-((1,1,1,3,3,3-hexamethyl-2-(trimethylsilyl)trisilan-2-yl)methyl)-3-oxopropanoate (4ao). Yield 51%. 16.0 mg. Colorless oil. ^{1}H NMR (600 MHz, CD_2Cl_2) δ 8.12 – 7.93 (m, 2H), 7.54 (d, *J* = 8.5 Hz, 2H), 6.82 (d, *J* = 8.0 Hz, 1H), 6.76 – 6.67 (m, 2H), 5.95 (ddt, *J* = 16.8, 10.0, 6.7 Hz, 1H), 5.17 – 4.98 (m, 2H), 4.55 (dd, *J* = 7.9, 5.0 Hz, 1H), 3.57 (s, 3H), 3.35 (d, *J* = 6.8 Hz, 2H), 1.82 (dd, *J* = 14.7, 7.9 Hz, 1H), 1.54 (dd, *J* = 14.7, 4.9 Hz, 1H), 1.36 (s, 9H), 0.20 (s, 27H). ^{13}C NMR (125 MHz, CD_2Cl_2) δ 193.7, 169.9, 157.8, 151.3, 139.9, 138.2, 137.5, 133.8, 129.1, 126.1, 122.5, 120.7, 116.2, 113.0, 55.7, 53.3, 40.4, 35.5, 31.2, 7.2, 1.2. HRMS (ESI$^+$) m/z calcd. $C_{33}H_{54}O_4NaSi_4^+$ $[M+Na]^+$: 649.2997, found: 649.2995.

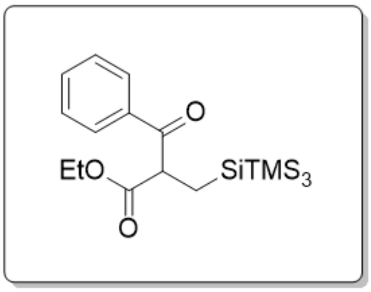

ethyl 2-((1,1,1,3,3,3-hexamethyl-2-(trimethylsilyl)trisilan-2-yl)methyl)-3-oxo-3-phenyl propanoate (4ap). Yield 75%. 16.9 mg. Colorless oil. ^{1}H NMR (500 MHz, CD_2Cl_2) δ 7.95 (d, *J* = 7.8 Hz, 2H), 7.60 (t, *J* = 7.4 Hz, 1H), 7.49 (t, *J* = 7.3 Hz, 2H), 4.33 (m, 1H), 4.10 (q, *J* = 7.2 Hz, 2H), 1.59 (m, 1H), 1.45 – 1.36 (m, 1H), 1.14 (t, *J* = 7.2, 3H), 0.17 (s, 27H). ^{13}C NMR (125 MHz, CD_2Cl_2) δ 195.2, 171.2, 136.3, 133.8, 129.1, 129.0, 61.8, 53.6, 14.1, 7.3, 1.2. HRMS (ESI$^+$) m/z calcd. $C_{21}H_{40}O_3NaSi_4^+$ $[M+Na]^+$: 475.1952, found: 475.1954.

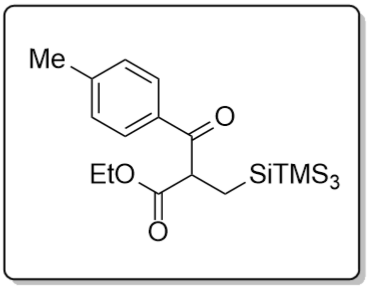

ethyl 2-((1,1,1,3,3,3-hexamethyl-2-(trimethylsilyl)trisilan-2-yl)methyl)-3-oxo-3-(*p*-tolyl) propanoate (4aq). Yield 69%. 16.1 mg. Colorless oil. ^{1}H NMR (600 MHz, CD_2Cl_2) δ 7.86 (d, *J* = 8.3 Hz, 2H), 7.30 (d, *J* = 8.0 Hz, 2H), 4.31 (t, *J* = 6.7 Hz, 1H), 4.11 (m, 2H), 2.42 (s, 3H), 1.60 (dd, *J* = 14.7, 6.8 Hz, 1H), 1.39 (dd, *J* = 14.7, 6.6 Hz, 1H), 1.15 (t, *J* = 7.1 Hz, 3H), 0.18 (s, 27H). ^{13}C NMR (150 MHz, CD_2Cl_2) δ 194.7, 171.3, 144.9, 133.8, 129.9, 129.1, 61.7, 53.5, 21.8, 14.2, 7.4, 1.3. HRMS (ESI$^+$) m/z calcd. $C_{22}H_{43}O_3Si_4^+$ $[M+H]^+$: 467.2289, found: 467.2290.

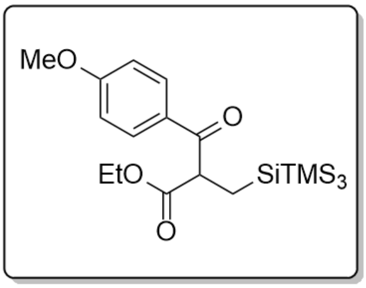

ethyl 2-((1,1,1,3,3,3-hexamethyl-2-(trimethylsilyl)trisilan-2-yl)methyl)-3-(4-methoxyphenyl)-3-oxopropanoate (4ar). Yield 68%. 16.5 mg. Colorless oil. ^{1}H NMR (600 MHz, CD_2Cl_2) δ 7.98 – 7.92 (m, 2H), 6.99 – 6.94 (m, 2H), 4.29 (t, J = 6.7 Hz, 1H), 4.10 (m, 2H), 3.87 (s, 3H), 1.59 (dd, J = 14.7, 6.8 Hz, 1H), 1.39 (dd, J = 14.7, 6.5 Hz, 1H), 1.16 (t, J = 7.1 Hz, 3H), 0.17 (s, 27H). ^{13}C NMR (125 MHz, CD_2Cl_2) δ 193.6, 171.4, 164.3, 131.3, 129.3, 114.3, 61.7, 56.0, 53.3, 14.2, 7.4, 1.3. HRMS (ESI$^+$) m/z calcd. $C_{22}H_{43}O_4Si_4^+$ [M+H]$^+$: 483.2238, found: 483.2240.

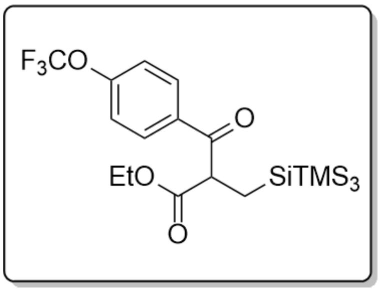

ethyl 2-((1,1,1,3,3,3-hexamethyl-2-(trimethylsilyl)trisilan-2-yl)methyl)-3-oxo-3-(4-(trifluoro meth oxy)phenyl)propanoate (4as). Yield 60%. 16.1 mg. Colorless oil. ^{1}H NMR (500 MHz, CD_2Cl_2) δ 8.24 – 7.73 (m, 2H), 7.32 (m, 2H), 4.29 (m, 1H), 4.19 – 3.99 (m, 2H), 1.60 (m, 1H), 1.41 (m, 1H), 1.14 (t, J = 7.2 Hz, 3H), 0.18 (s, 27H). ^{13}C NMR (125 MHz, CD_2Cl_2) δ 193.7, 170.9, 153.2 (d, J = 3.1 Hz), 134.7, 131.1, 120.9, 62.0, 53.9, 14.1, 7.2, 1.3. ^{19}F NMR (471 MHz, CD_2Cl_2) δ -58.0. HRMS (ESI$^+$) m/z calcd. $C_{22}H_{39}O_4F_3NaSi_4^+$ [M+Na]$^+$: 559.1775, found: 559.1774.

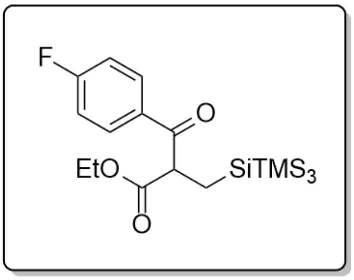

ethyl 3-(4-fluorophenyl)-2-((1,1,1,3,3,3-hexamethyl-2-(trimethylsilyl)trisilan-2-yl)methyl)-3-oxo propanoate (4at). Yield 63%. 14.8 mg. Colorless oil. ^{1}H NMR (500 MHz, CD_2Cl_2) δ 8.00 (m, 2H), 7.17 (m, 2H), 4.28 (t, J = 6.7 Hz, 1H), 4.10 (m, 2H), 1.59 (dd, J = 14.7, 6.9 Hz, 1H), 1.39 (dd, J = 14.7, 6.3 Hz, 1H), 1.14 (t, J = 7.1 Hz, 3H), 0.17 (s, 27H). ^{13}C NMR (125 MHz, CD_2Cl_2) δ 193.6, 171.0, 166.3 (d, J = 254.6 Hz), 132.8 (d, J = 3.2 Hz), 131.7 (d, J = 9.3 Hz), 116.2 (d, J = 22.0 Hz), 61.9, 53.7, 14.1, 7.3, 1.2. ^{19}F NMR (376 MHz, CD_2Cl_2) δ -105.5. HRMS (ESI^{+}) m/z calcd. $C_{21}H_{39}O_3FNaSi_4^{+}$ [M+Na]$^{+}$: 493.1858, found: 493.1858.

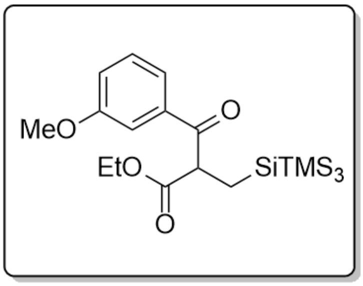

ethyl 2-((1,1,1,3,3,3-hexamethyl-2-(trimethylsilyl)trisilan-2-yl)methyl)-3-(3-methoxyphenyl)-3-oxopropanoate (4au). Yield 63%. 15.2 mg. Colorless oil. ^{1}H NMR (500 MHz, CD_2Cl_2) δ 7.52 (d, J = 7.8 Hz, 1H), 7.49 (s, 1H), 7.39 (t, J = 7.9 Hz, 1H), 7.14 (m, 1H), 4.31 (t, J = 6.6 Hz, 1H), 4.11 (q, J = 7.1 Hz, 2H), 3.85 (s, 3H), 1.59 (dd, J = 14.6, 6.8 Hz, 1H), 1.38 (dd, J = 14.7, 6.5 Hz, 1H), 1.15 (t, J = 7.1 Hz, 3H), 0.17 (s, 27H). ^{13}C NMR (125 MHz, CD_2Cl_2) δ 195.0, 171.2, 160.5, 137.7, 130.1, 121.4, 120.2, 113.3, 61.8, 55.8, 53.7, 14.1, 7.3, 1.2. HRMS (ESI^{+}) m/z calcd. $C_{22}H_{42}O_4NaSi_4^{+}$ [M+Na]$^{+}$: 505.2058, found: 505.2059.

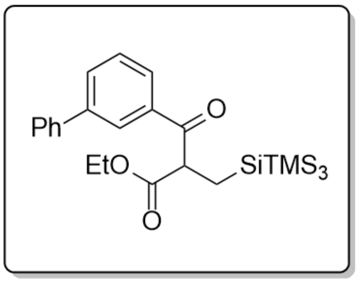

ethyl 3-([1,1'-biphenyl]-3-yl)-2-((1,1,1,3,3,3-hexamethyl-2-(trimethylsilyl)trisilan-2-yl)methyl)-3-oxopropanoate (4av). Yield 72%. 19.1 mg. Colorless oil. ^{1}H NMR (600 MHz, CD_2Cl_2) δ 8.22 (s, 1H), 7.94 (dd, *J* = 7.9, 1.7 Hz, 1H), 7.85 (dd, *J* = 7.7, 1.8 Hz, 1H), 7.67 – 7.62 (m, 2H), 7.58 (t, *J* = 7.7 Hz, 1H), 7.49 (t, *J* = 7.6 Hz, 2H), 7.43 – 7.37 (m, 1H), 4.41 (t, *J* = 6.7 Hz, 1H), 4.20 – 4.05 (m, 2H), 1.64 (dd, *J* = 14.7, 6.7 Hz, 1H), 1.44 (dd, *J* = 14.7, 6.6 Hz, 1H), 1.16 (t, *J* = 7.1 Hz, 3H), 0.19 (s, 27H). ^{13}C NMR (125 MHz, CD_2Cl_2) δ 195.2, 171.2, 142.3, 140.4, 136.9, 132.4, 129.6, 129.4, 128.3, 127.8, 127.6, 127.5, 61.9, 53.8, 14.2, 7.4, 1.3. HRMS (ESI$^+$) m/z calcd. $C_{27}H_{44}O_3NaSi_4^+$ [M+Na]$^+$: 551.2265, found: 551.2266.

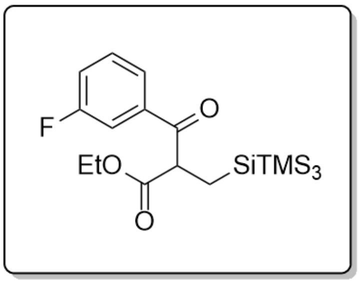

ethyl 3-(3-fluorophenyl)-2-((1,1,1,3,3,3-hexamethyl-2-(trimethylsilyl)trisilan-2-yl)methyl)-3-oxo propanoate (4aw). Yield 60%. 14.1 mg. Colorless oil. ^{1}H NMR (600 MHz, CD_2Cl_2) δ 7.76 (m, 1H), 7.66 (m, 1H), 7.49 (m, 1H), 7.31 (m, 1H), 4.28 (t, *J* = 6.6 Hz, 1H), 4.12 (m, 2H), 1.60 (dd, *J* = 14.7, 6.9 Hz, 1H), 1.40 (dd, *J* = 14.7, 6.4 Hz, 1H), 1.15 (t, *J* = 7.2 Hz, 3H), 0.18 (s, 27H). ^{13}C NMR (125 MHz, CD_2Cl_2) δ 194.0, 170.9, 163.4 (d, *J* = 247.7 Hz), 138.5 (d, *J* = 6.3 Hz), 131.0 (d, *J* = 7.7 Hz), 124.8 (d, *J* = 2.9 Hz), 120.8 (d, *J* = 21.6 Hz), 115.6 (d, *J* = 22.6 Hz), 62.0, 53.9, 14.1, 7.3, 1.3. ^{19}F NMR (376 MHz, CD_2Cl_2) δ -112.2. HRMS (ESI$^+$) m/z calcd. $C_{21}H_{39}O_3FNaSi_4^+$ [M+Na]$^+$: 493.1858, found: 493.1859.

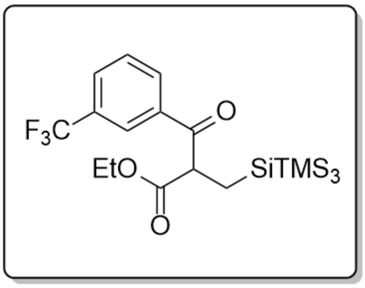

ethyl 2-((1,1,1,3,3,3-hexamethyl-2-(trimethylsilyl)trisilan-2-yl)methyl)-3-oxo-3-(3-(trifluoromethyl)phenyl)propanoate (4ax). Yield 52%. 13.5 mg. Colorless oil. ^{1}H NMR (500 MHz, CD_2Cl_2) δ 8.23 (s, 1H), 8.15z (d, J = 7.9 Hz, 1H), 7.86 (d, J = 7.8 Hz, 1H), 7.66 (t, J = 7.9 Hz, 1H), 4.36 – 4.29 (m, 1H), 4.11 (q, J = 7.2 Hz, 2H), 1.63 – 1.55 (m, 1H), 1.47 – 1.37 (m, 1H), 1.14 (t, J = 7.2 Hz, 3H), 0.17 (s, 27H). ^{13}C NMR (125 MHz, CD_2Cl_2) δ 194.1, 170.6, 136.9, 132.2, 130.2, 130.2, 130.0, 125.8, 125.8, 62.1, 14.1, 7.2, 1.2. ^{19}F NMR (471 MHz, CD_2Cl_2) δ -63.2. HRMS (ESI$^+$) m/z calcd. $C_{22}H_{39}O_3F_3NaSi_4^+$ [M+Na]$^+$: 543.1826, found: 543.1827.

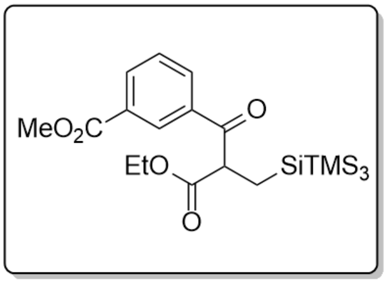

methyl 3-(3-ethoxy-2-((1,1,1,3,3,3-hexamethyl-2-(trimethylsilyl)trisilan-2-yl)methyl)-3-oxopropanoyl)benzoate (4ay). Yield 60%. 15.3 mg. White solid. ^{1}H NMR (600 MHz, CD_2Cl_2) δ 8.59 (s, 1H), 8.25 (d, J = 7.7 Hz, 1H), 8.15 (d, J = 7.8 Hz, 1H), 7.60 (t, J = 7.8 Hz, 1H), 4.37 (t, J = 6.7 Hz, 1H), 4.12 (q, J = 7.2 Hz, 2H), 3.93 (s, 3H), 1.57 (dd, J = 14.7, 6.4 Hz, 1H), 1.43 (dd, J = 14.6, 6.9 Hz, 1H), 1.15 (t, J = 7.1 Hz, 3H), 0.18 (s, 27H). ^{13}C NMR (125 MHz, CD_2Cl_2) δ 194.7, 170.8, 166.3, 136.6, 134.5, 133.0, 131.5, 129.9, 129.5, 61.9, 53.7, 52.7, 14.1, 7.3, 1.3. HRMS (ESI$^+$) m/z calcd. $C_{23}H_{42}O_5NaSi_4^+$ [M+Na]$^+$: 533.2007, found: 533.2006.

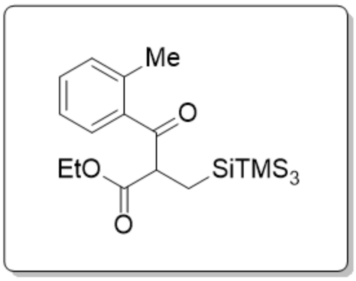

ethyl 2-((1,1,1,3,3,3-hexamethyl-2-(trimethylsilyl)trisilan-2-yl)methyl)-3-oxo-3-(*o*-tolyl)propanoate (4az). Yield 55%. 12.8 mg. White solid. ^{1}H NMR (600 MHz, CD_2Cl_2) δ 7.59 (d, *J* = 7.8 Hz, 1H), 7.39 (t, *J* = 7.4 Hz, 1H), 7.28 (m, 2H), 4.18 (m, 1H), 4.07 (q, *J* = 7.1 Hz, 2H), 2.45 (s, 3H), 1.42 (m, 2H), 1.11 (t, *J* = 7.1 Hz, 3H), 0.17 (s, 27H). ^{13}C NMR (125 MHz, CD_2Cl_2) δ 199.3, 170.9, 139.0, 137.5, 132.3, 131.8, 128.4, 126.0, 61.6, 56.2, 21.0, 14.1, 6.7, 1.2, 1.2. HRMS (ESI$^+$) m/z calcd. $C_{22}H_{42}O_3NaSi_4^+$ [M+Na]$^+$: 489.2109, found: 489.2107.

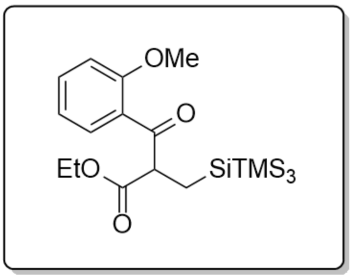

ethyl 2-((1,1,1,3,3,3-hexamethyl-2-(trimethylsilyl)trisilan-2-yl)methyl)-3-(2-methoxyphenyl)-3-oxopropanoate (4ba). Yield 40%. 9.7 mg. White solid. ^{1}H NMR (600 MHz, CD_2Cl_2) δ 7.72 (m, 1H), 7.49 (m, 1H), 7.11 – 6.87 (m, 2H), 4.41 (m, 1H), 4.14 – 3.99 (m, 2H), 3.87 (s, 3H), 1.75 – 1.53 (m, 1H), 1.25 (m, 1H), 1.22 – 1.03 (m, 3H), 0.16 (s, 27H). ^{13}C NMR (125 MHz, CD_2Cl_2) δ 196.5, 171.9, 158.8, 134.3, 131.4, 127.2, 121.1, 112.0, 61.2, 57.5, 55.6, 14.2, 6.3, 1.2. HRMS (ESI$^+$) m/z calcd. $C_{22}H_{42}O_4NaSi_4^+$ [M+Na]$^+$: 505.2058, found: 505.2058.

Appendix I

Spectral Copies of ^{1}H, ^{13}C, and ^{19}F NMR Data

Obtained in this study

4-(diisopropylsilyl)benzonitrile (3o).

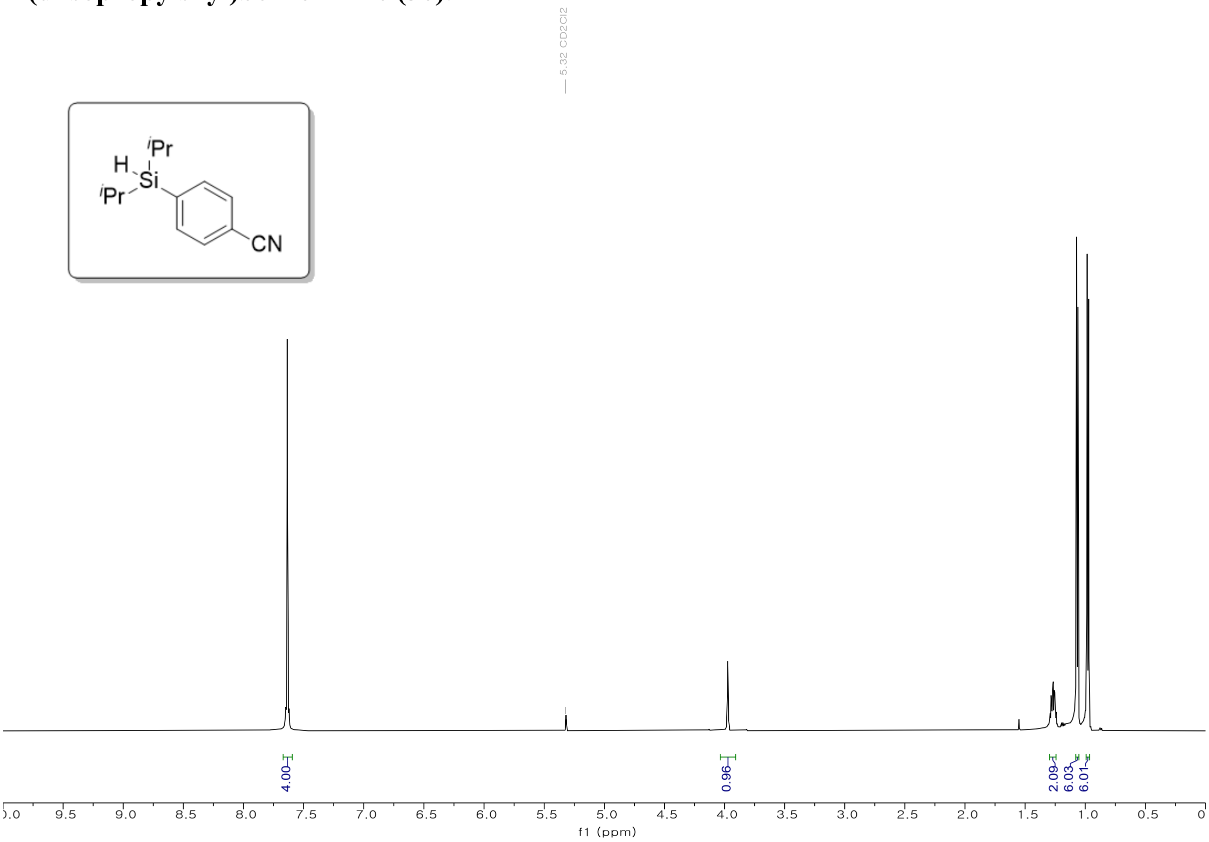

600 MHz, ^{1}H NMR in CD_2Cl_2

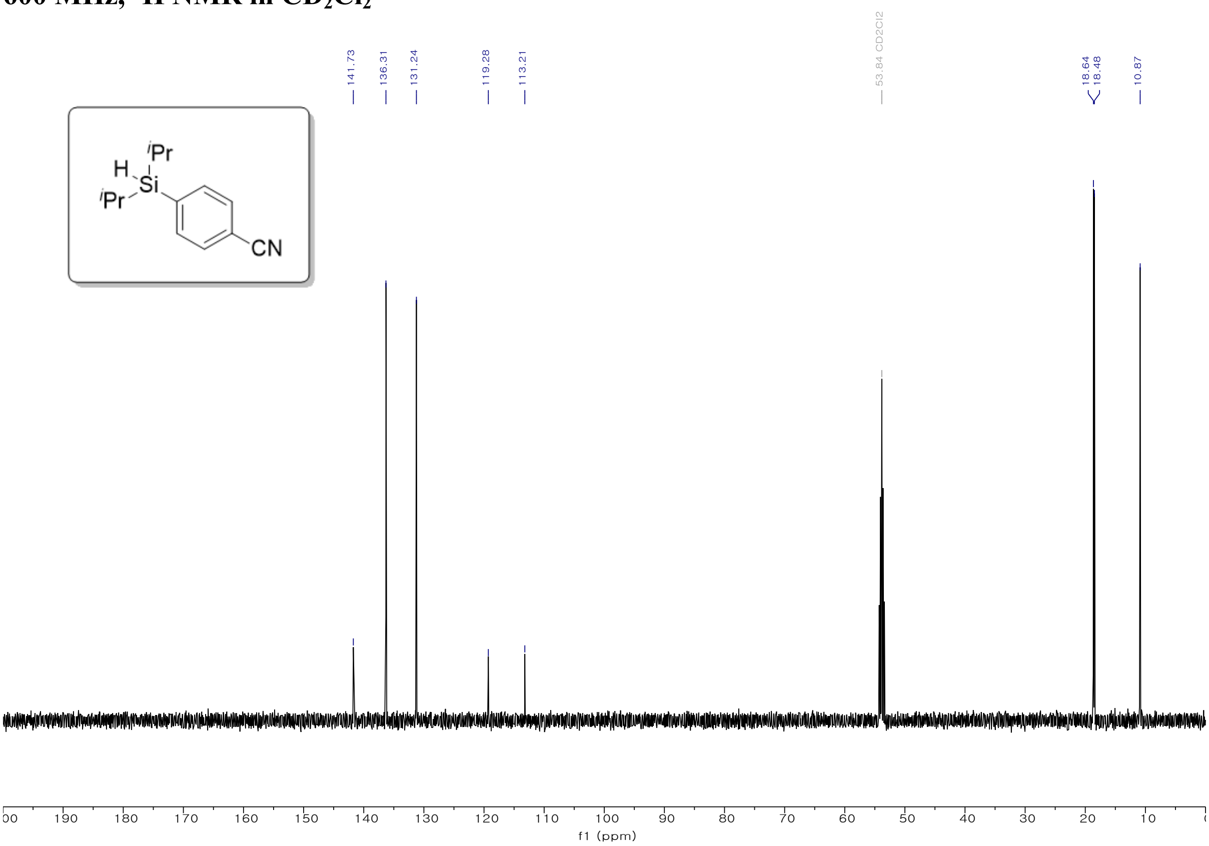

125 MHz, ^{13}C NMR in CD_2Cl_2

(1*S*,2*R*,4*S*)-1,7,7-trimethylbicyclo[2.2.1]heptan-2-yl 4-(dimethylsilyl)benzoate (3u).

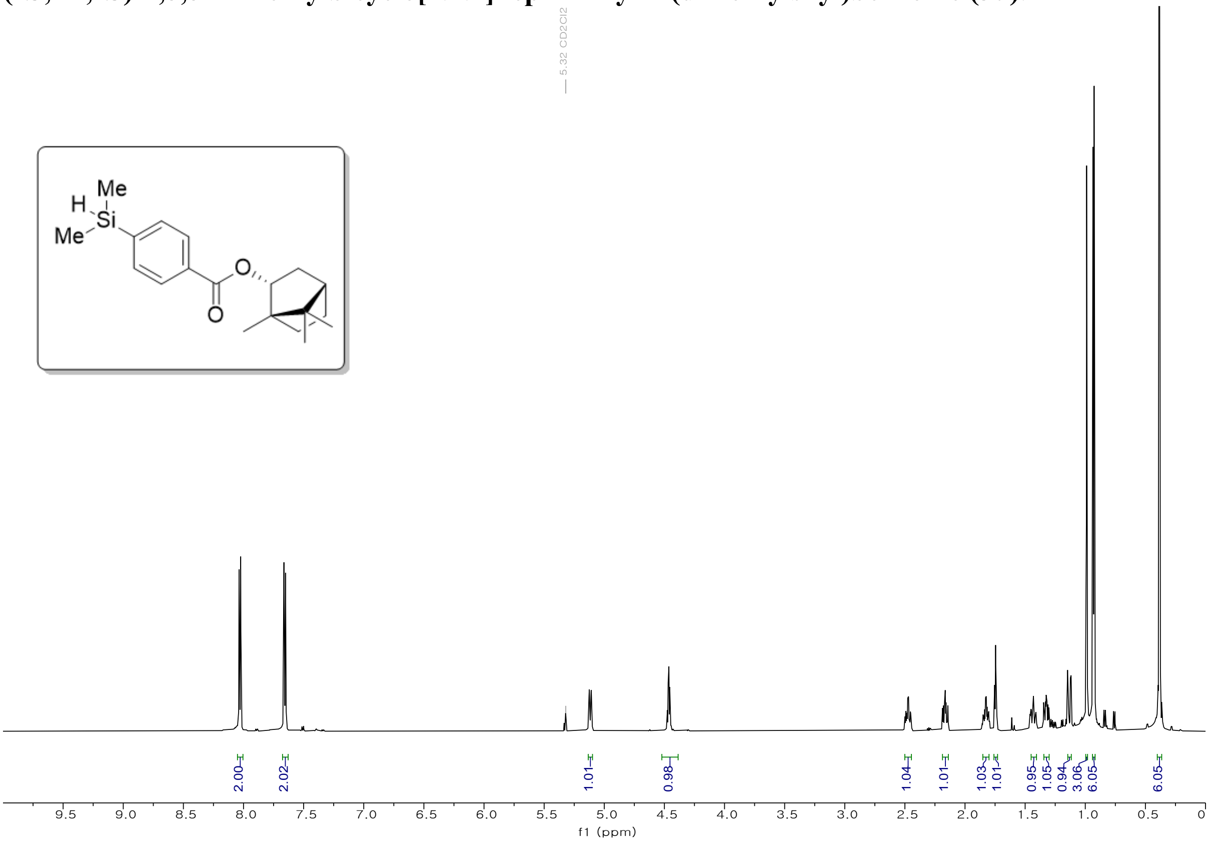

600 MHz, ^{1}H NMR in CD_2Cl_2

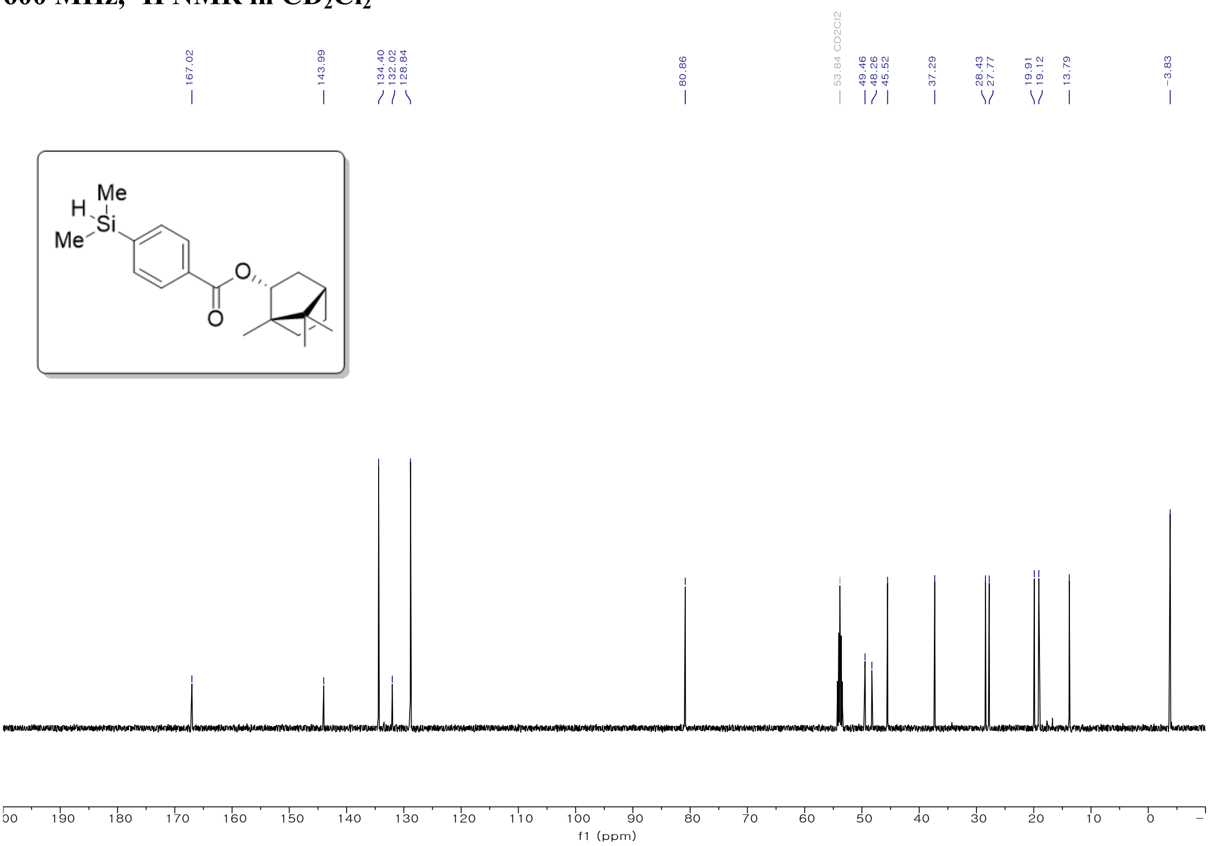

125 MHz, ^{13}C NMR in CD_2Cl_2

ethyl 3-(4-(*tert*-butyl)phenyl)-2-((1,1,1,3,3,3-hexamethyl-2-(trimethylsilyl)trisilan-2-yl)methyl)-3-oxopropanoate (4a).

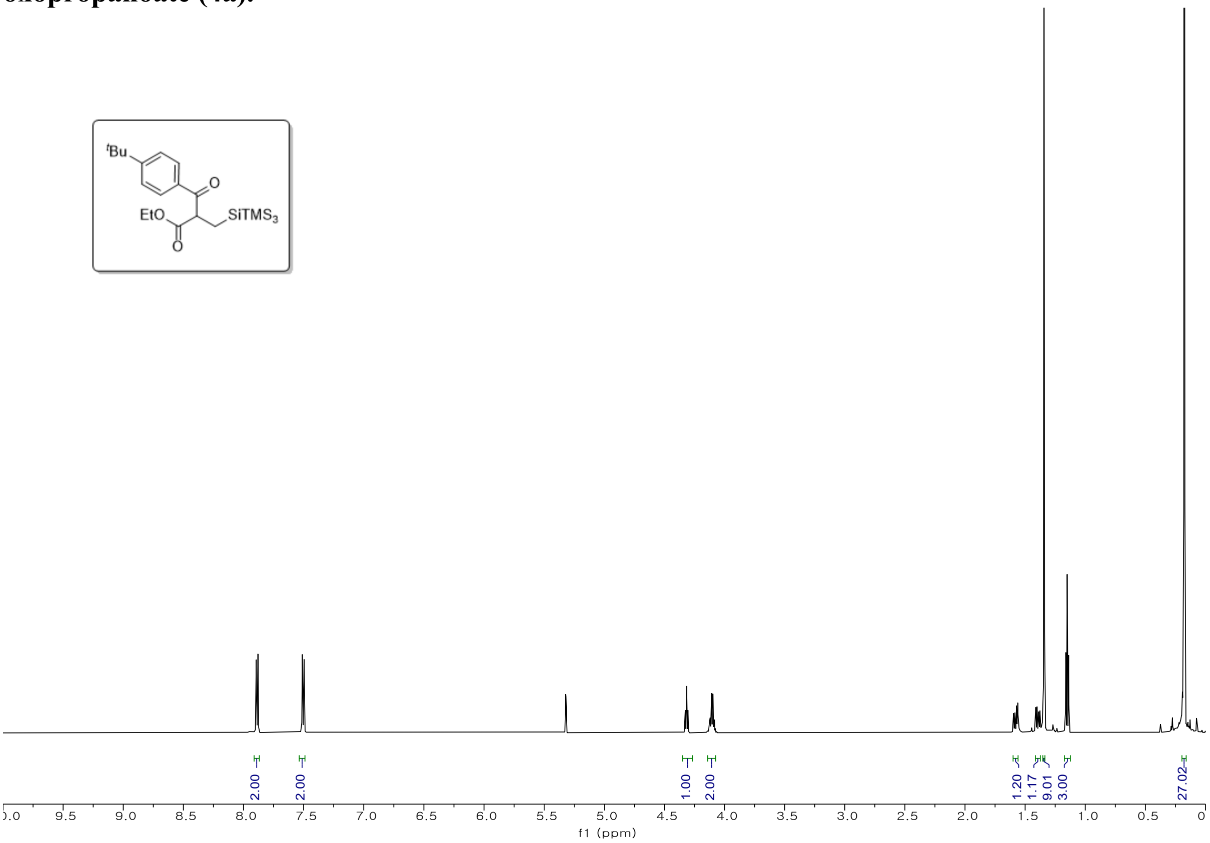

600 MHz, ^{1}H NMR in CD_2Cl_2

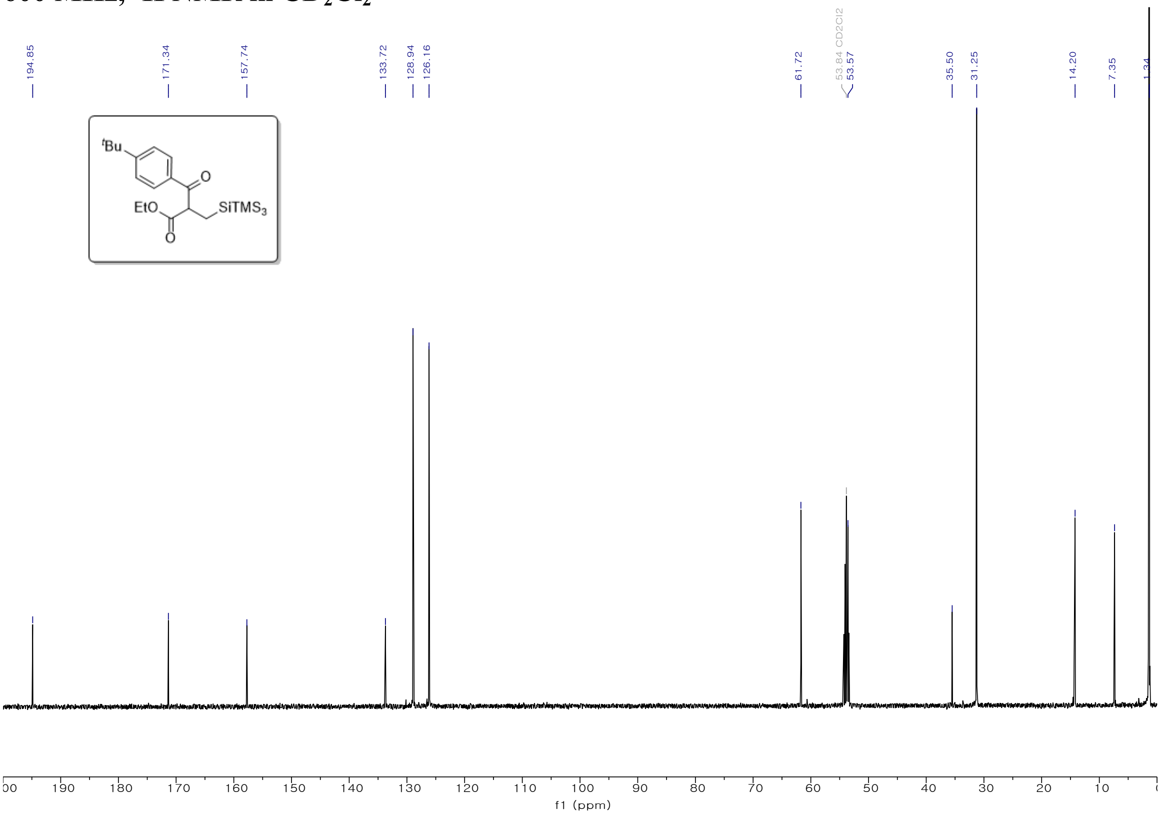

125 MHz, ^{13}C NMR in CD_2Cl_2

ethyl 3-(4-(*tert*-butyl)phenyl)-3-oxo-2-((triethylsilyl)methyl)propanoate (4b).

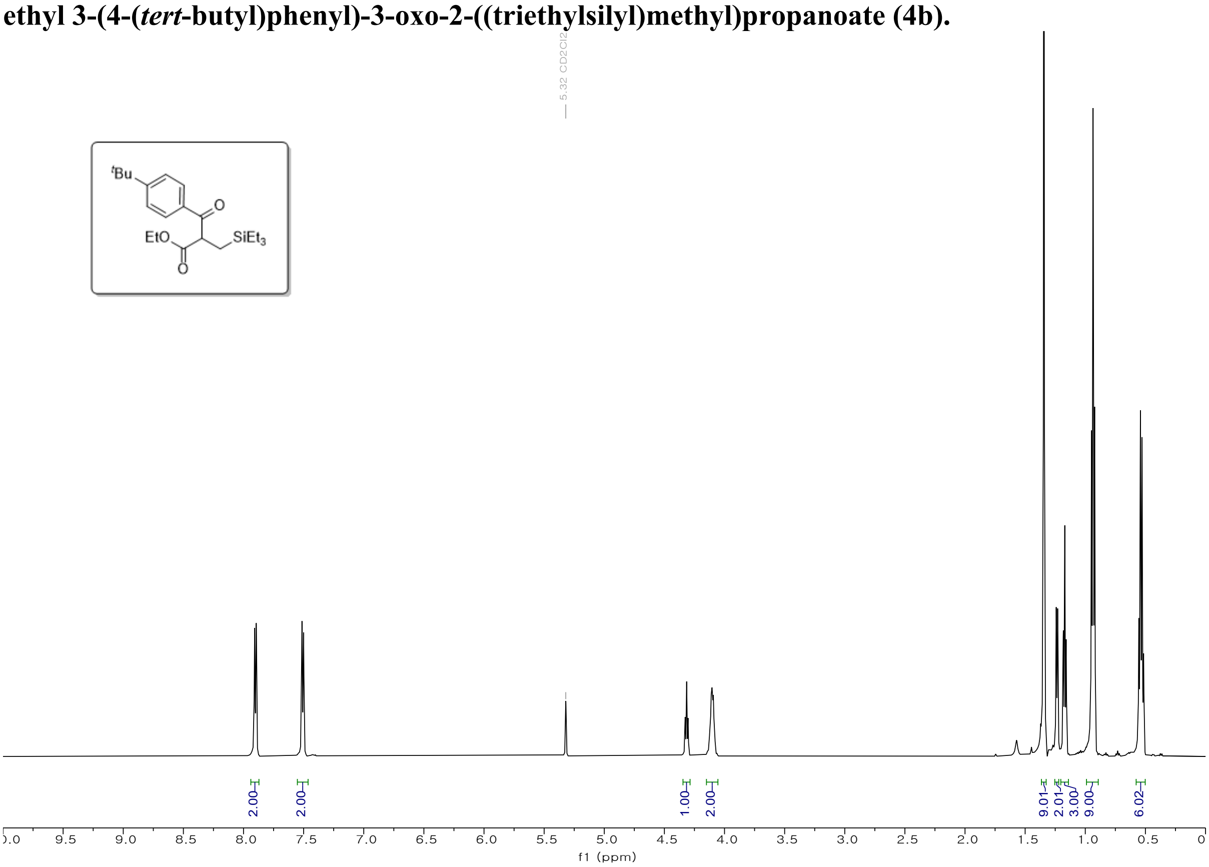

600 MHz, ^{1}H NMR in CD_2Cl_2

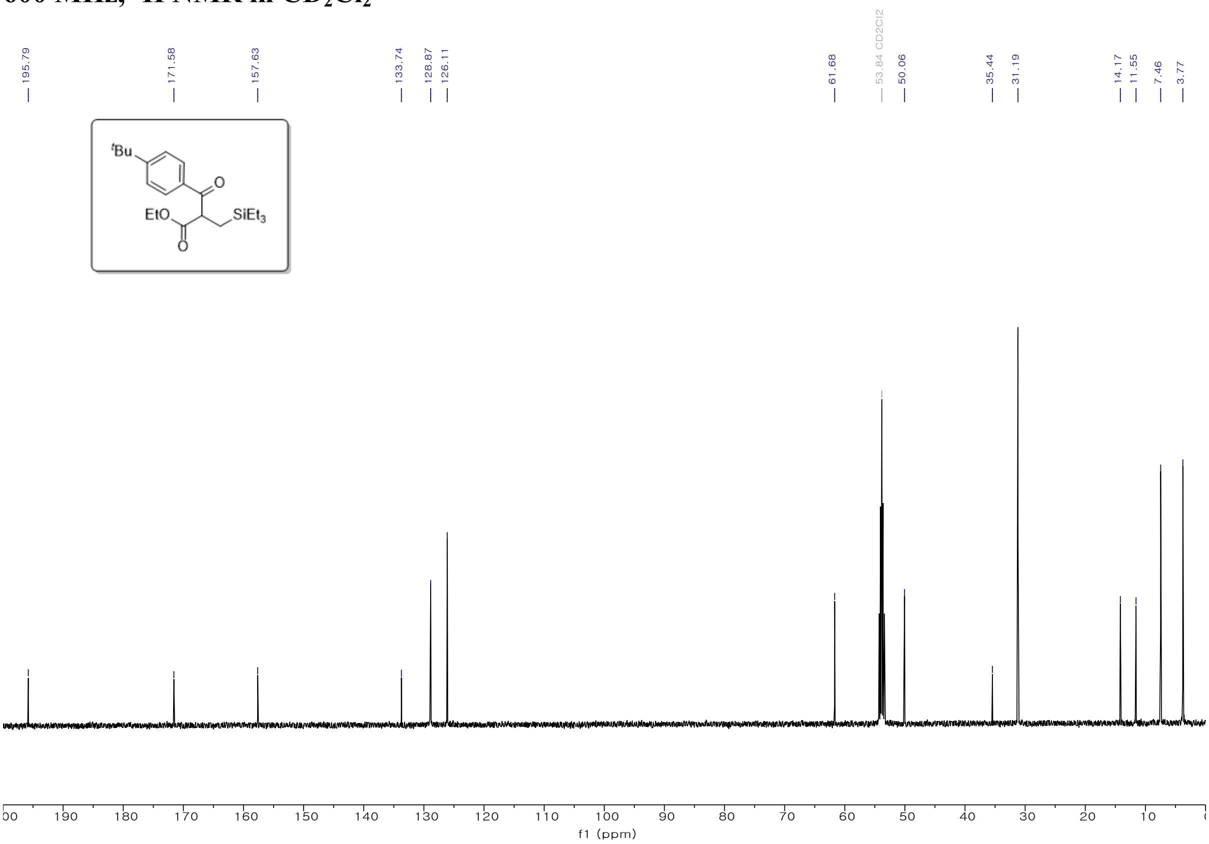

125 MHz, ^{13}C NMR in CD_2Cl_2

ethyl 3-(4-(*tert*-butyl)phenyl)-3-oxo-2-((triisopropylsilyl)methyl)propanoate (4c).

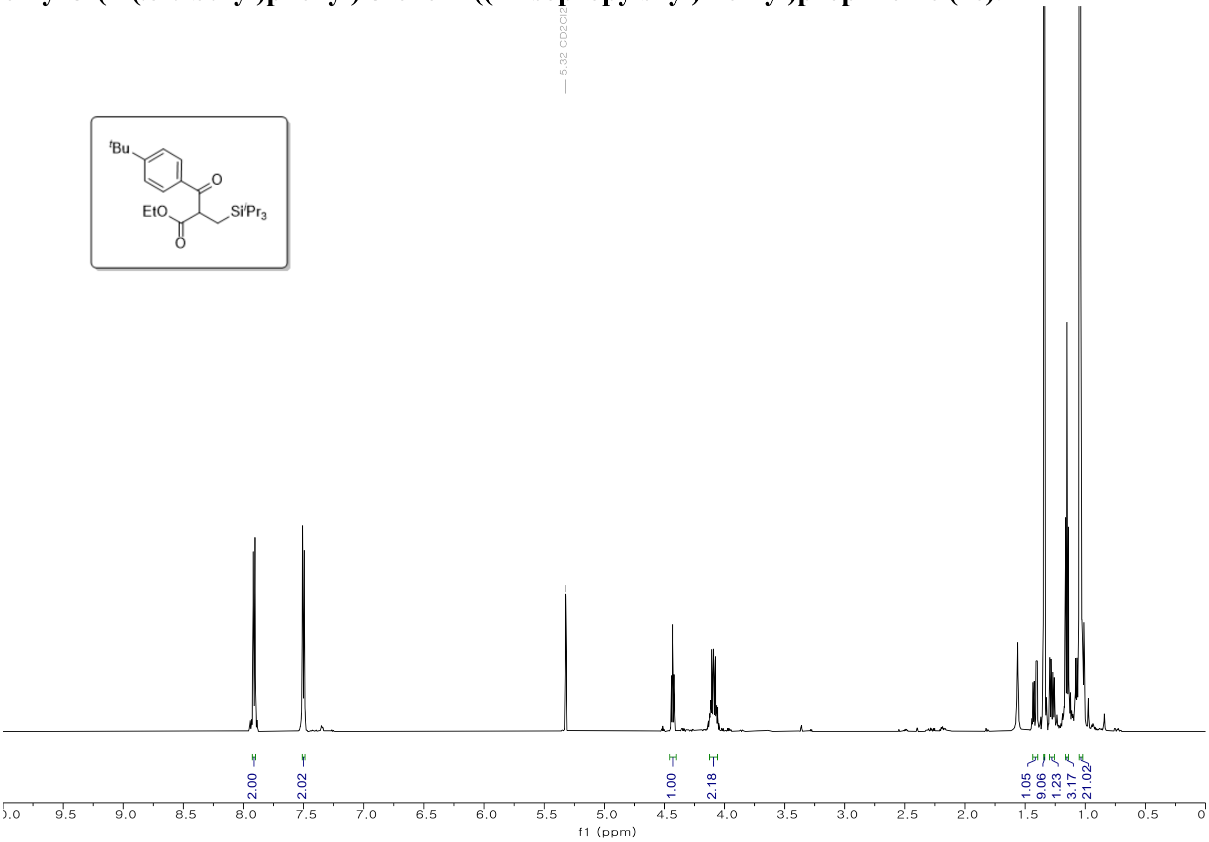

600 MHz, ^{1}H NMR in CD_2Cl_2

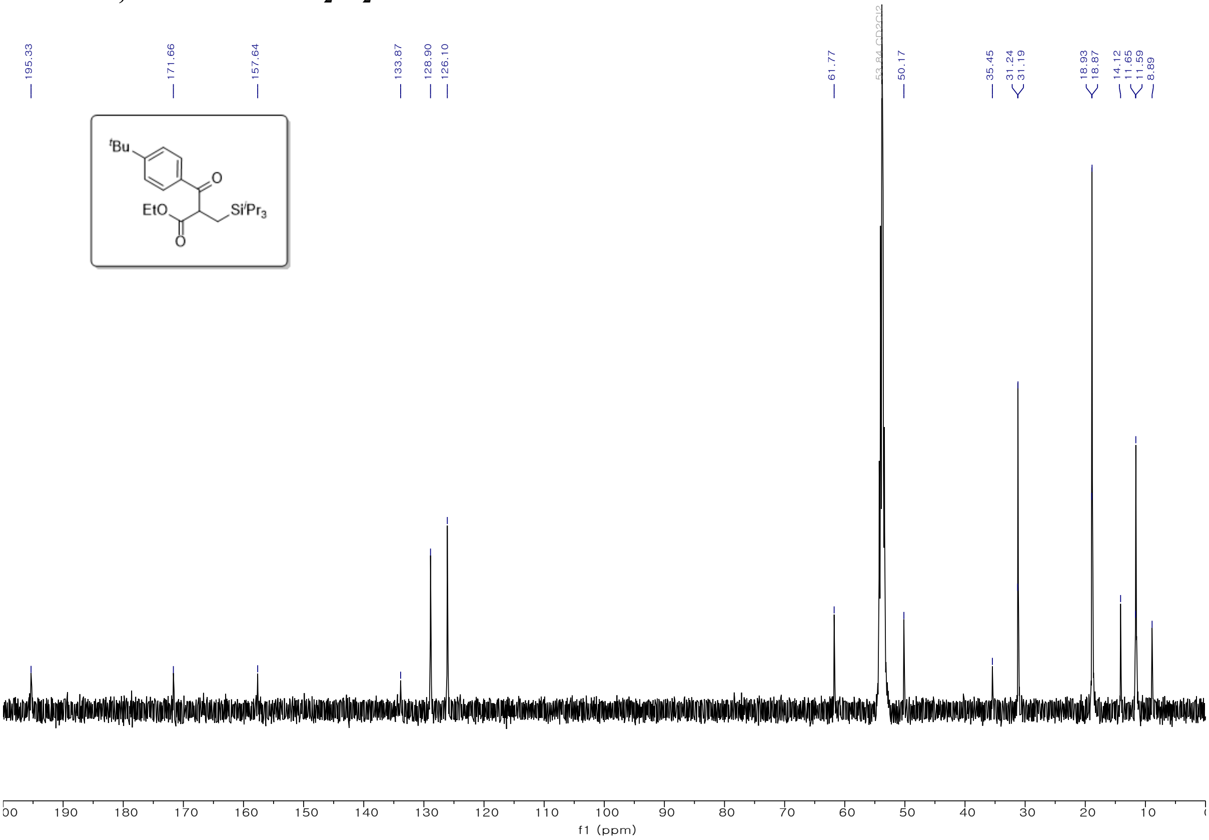

125 MHz, ^{13}C NMR in CD_2Cl_2

ethyl 3-(4-(*tert*-butyl)phenyl)-2-((*tert*-butyldimethylsilyl)methyl)-3-oxopropanoate (4d).

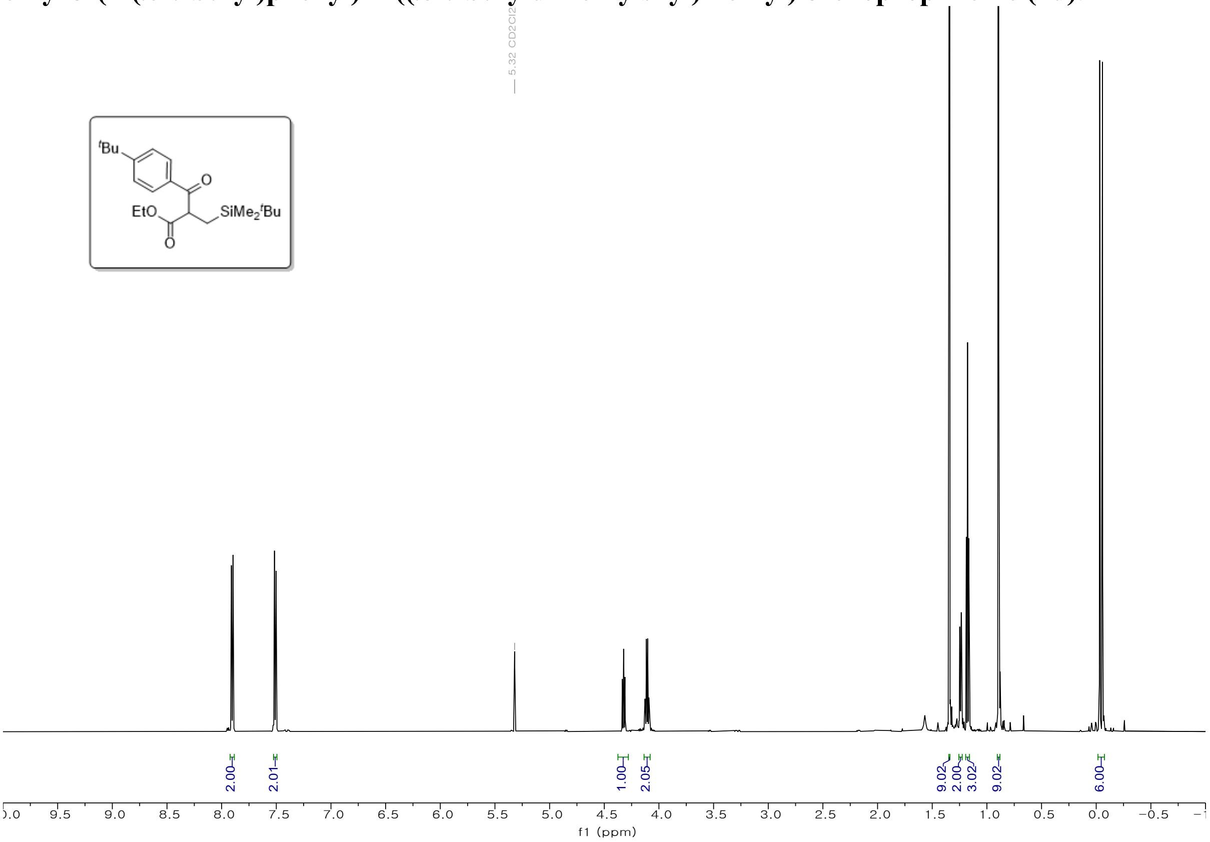

600 MHz, ^{1}H NMR in CD_2Cl_2

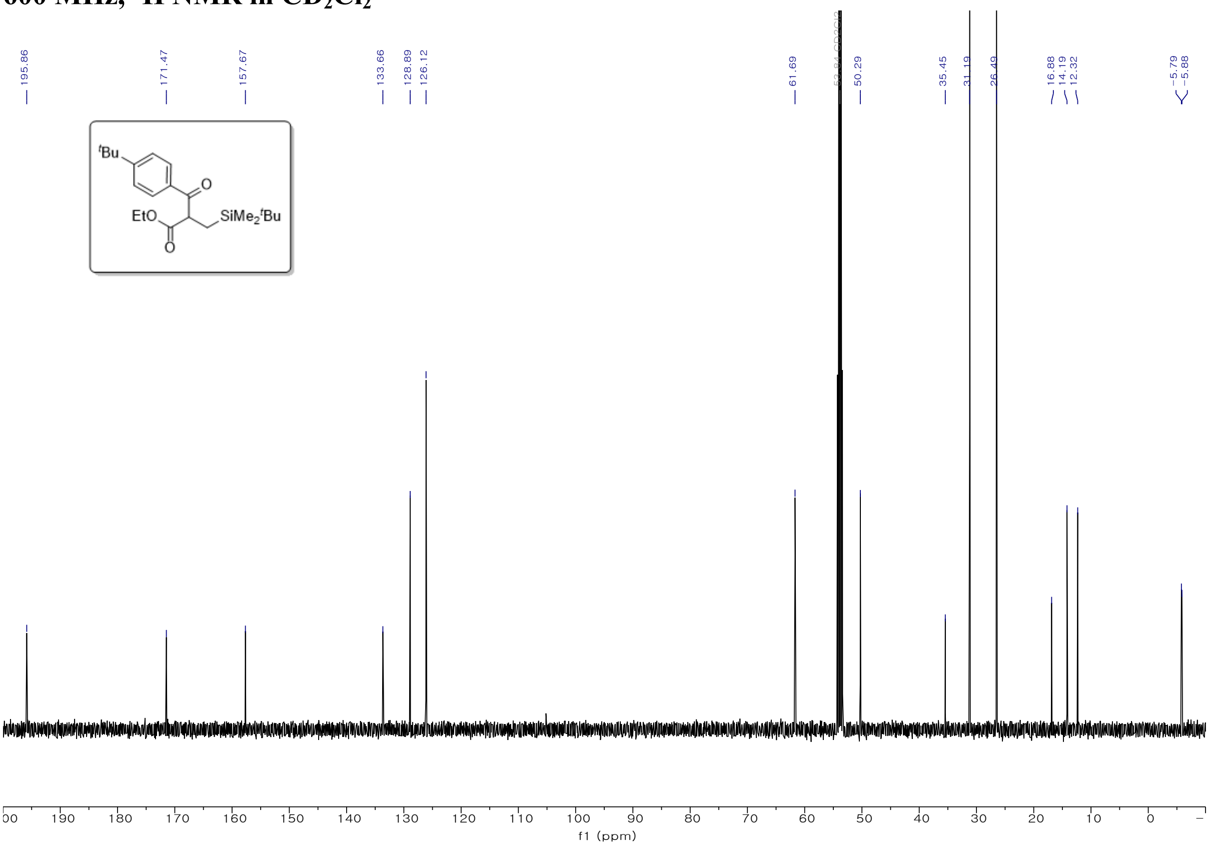

125 MHz, ^{13}C NMR in CD_2Cl_2

ethyl 2-((benzyldimethylsilyl)methyl)-3-(4-(*tert*-butyl)phenyl)-3-oxopropanoate (4e).

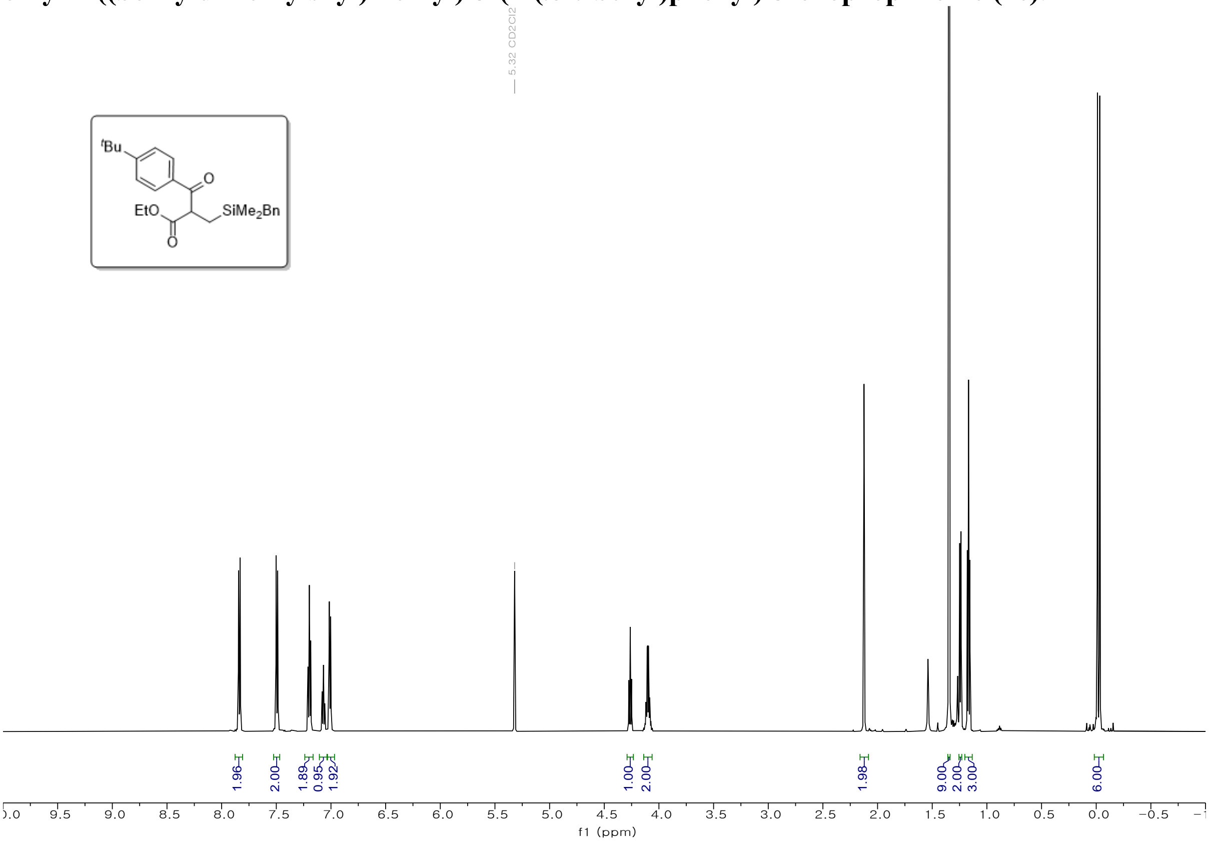

600 MHz, ^{1}H NMR in CD_2Cl_2

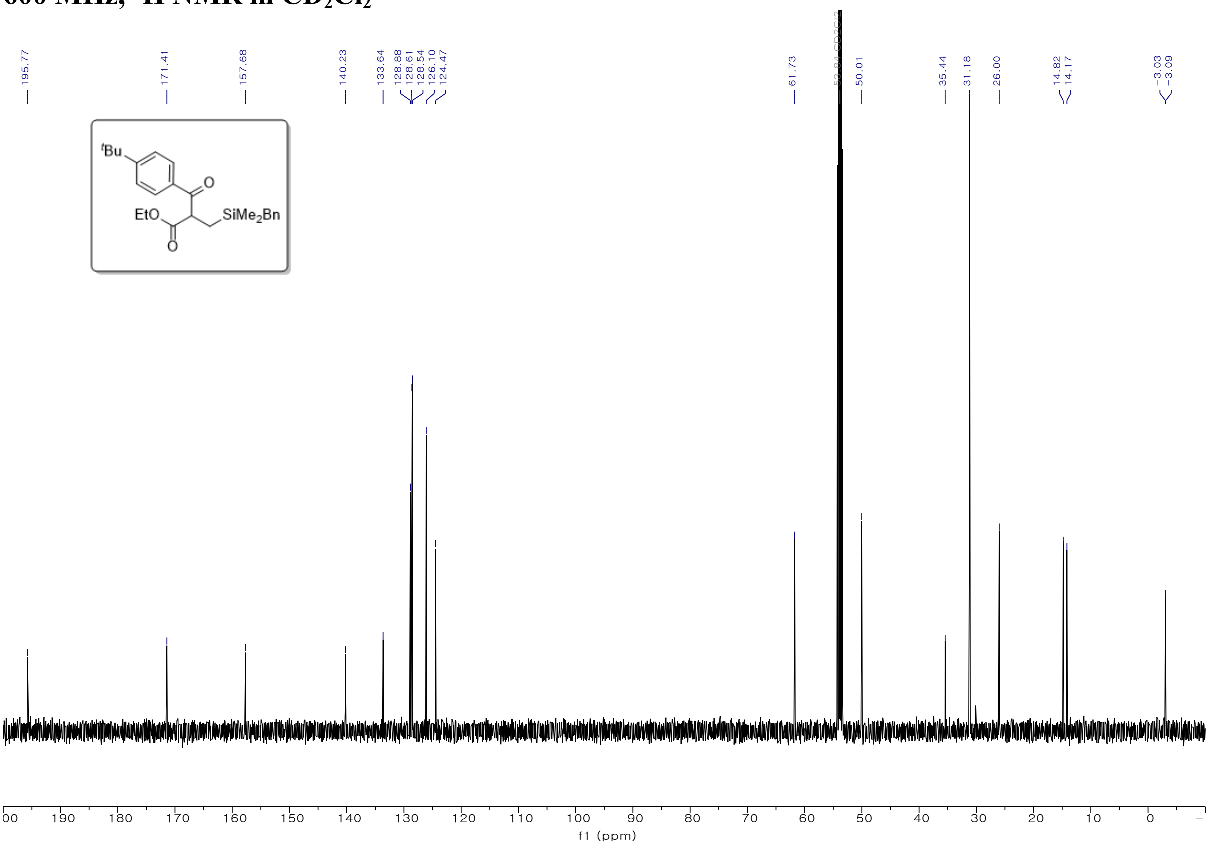

125 MHz, ^{13}C NMR in CD_2Cl_2

ethyl 3-(4-(*tert*-butyl)phenyl)-2-((isopropyldimethylsilyl)methyl)-3-oxopropanoate (4f).

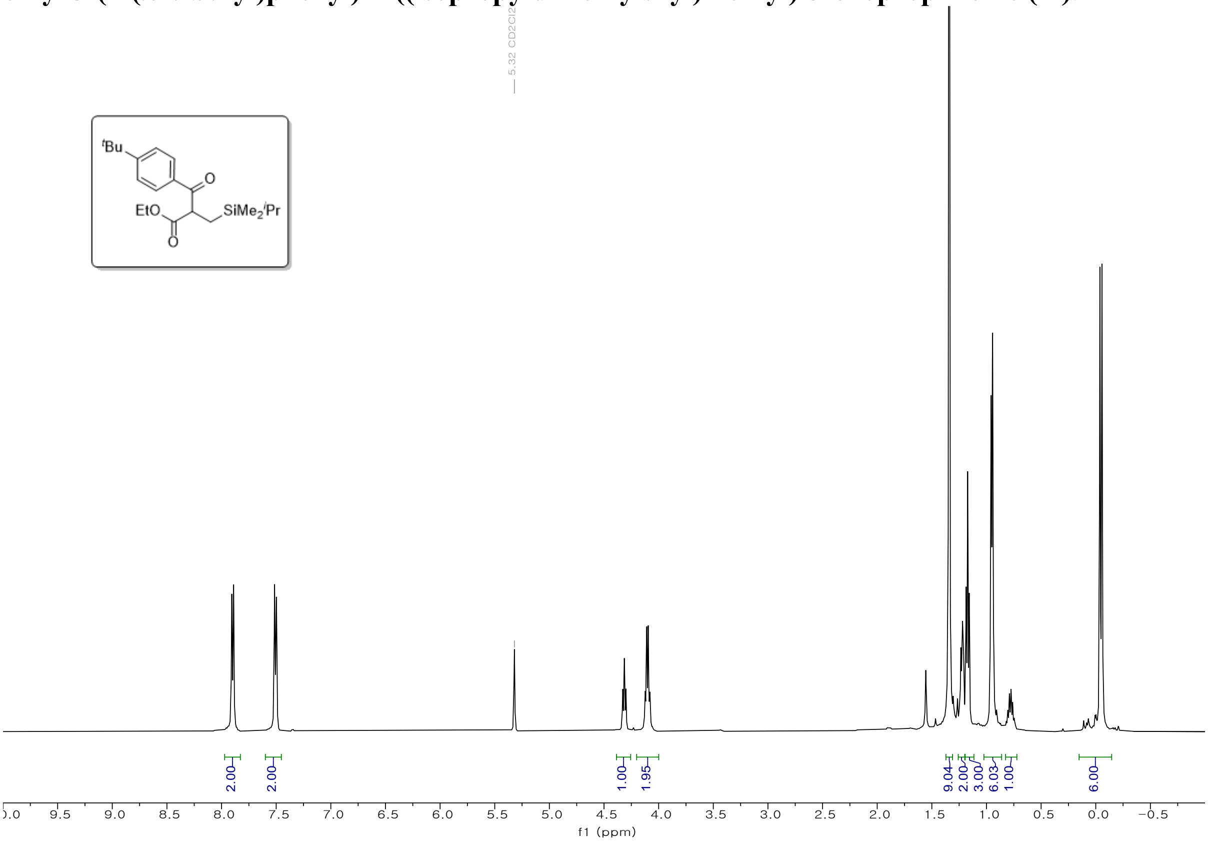

500 MHz, ^{1}H NMR in CD_2Cl_2

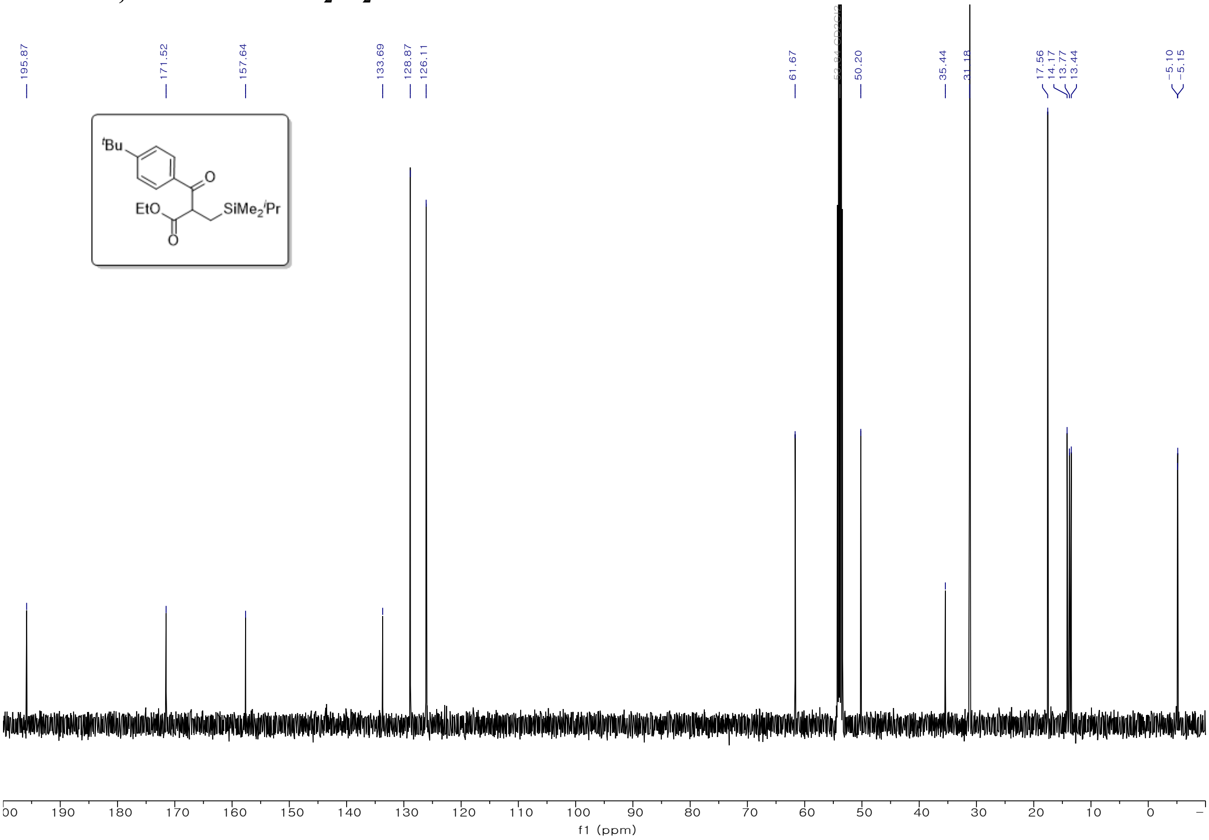

125 MHz, ^{13}C NMR in CD_2Cl_2

ethyl 3-(4-(*tert*-butyl)phenyl)-3-oxo-2-((tribenzylsilyl)methyl)propanoate (4g).

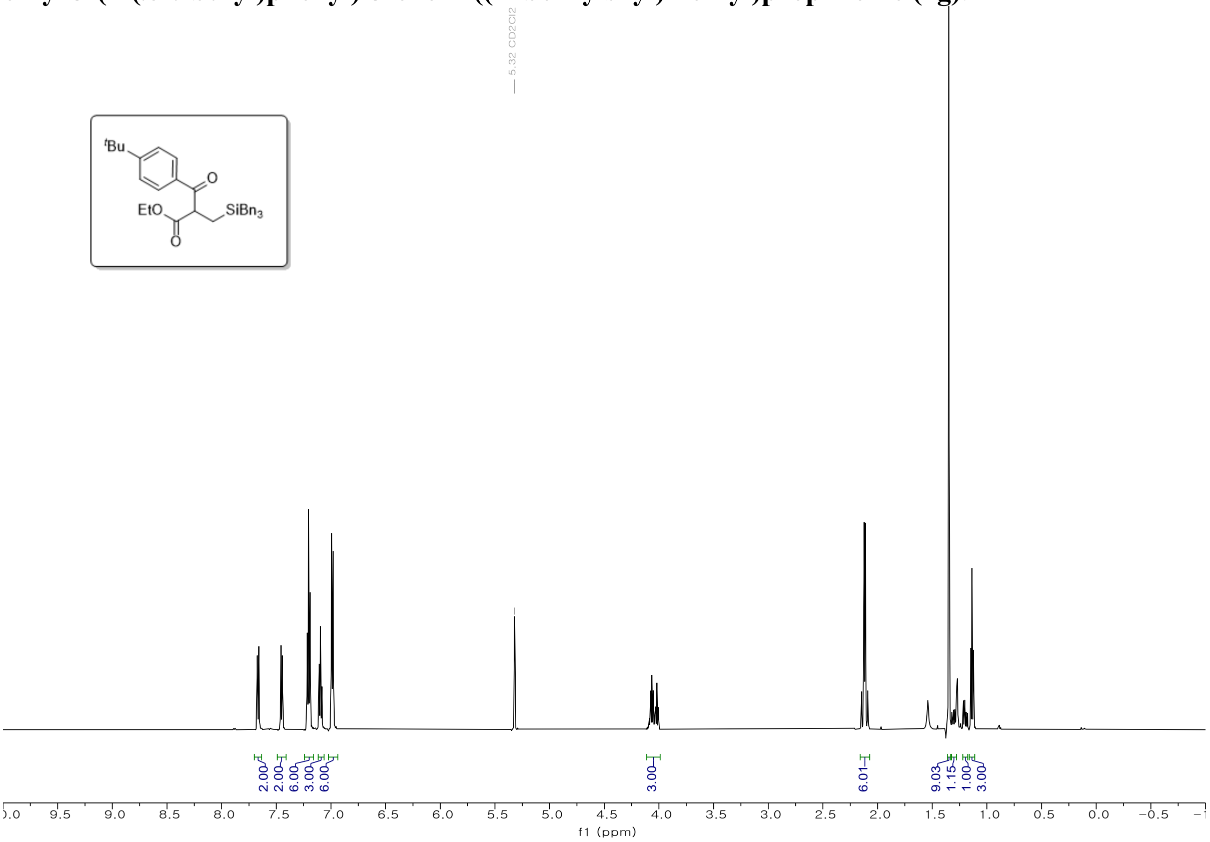

600 MHz, ^{1}H NMR in CD_2Cl_2

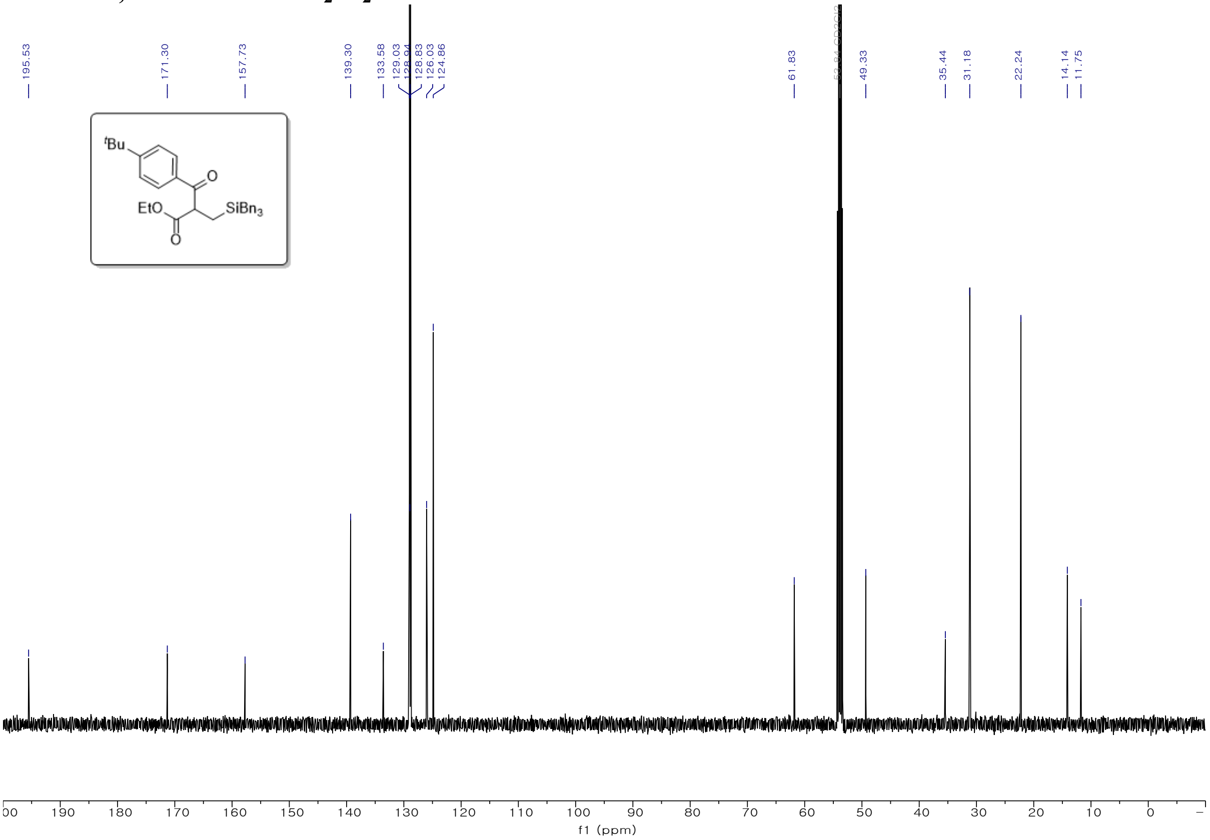

125 MHz, ^{13}C NMR in CD_2Cl_2

ethyl 3-(4-(*tert*-butyl)phenyl)-3-oxo-2-((triphenylsilyl)methyl)propanoate (4h).

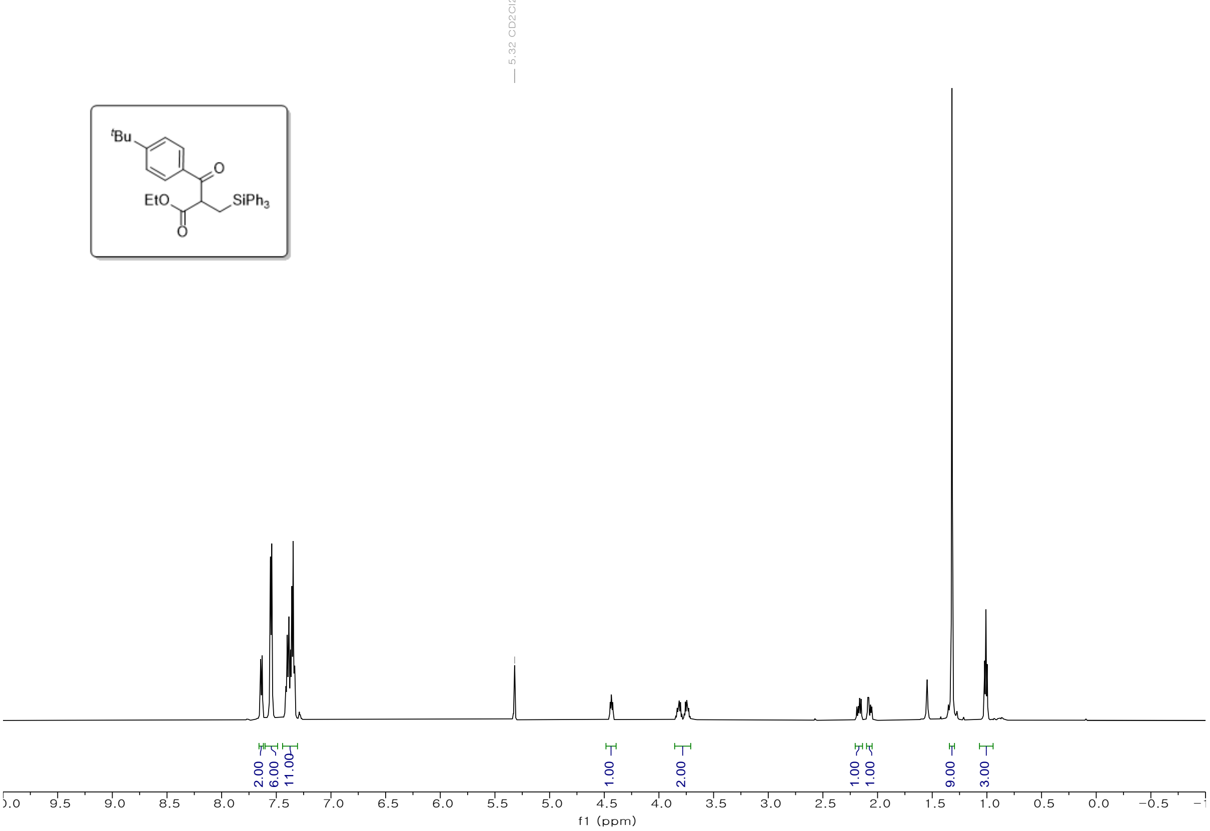

600 MHz, ^{1}H NMR in CD_2Cl_2

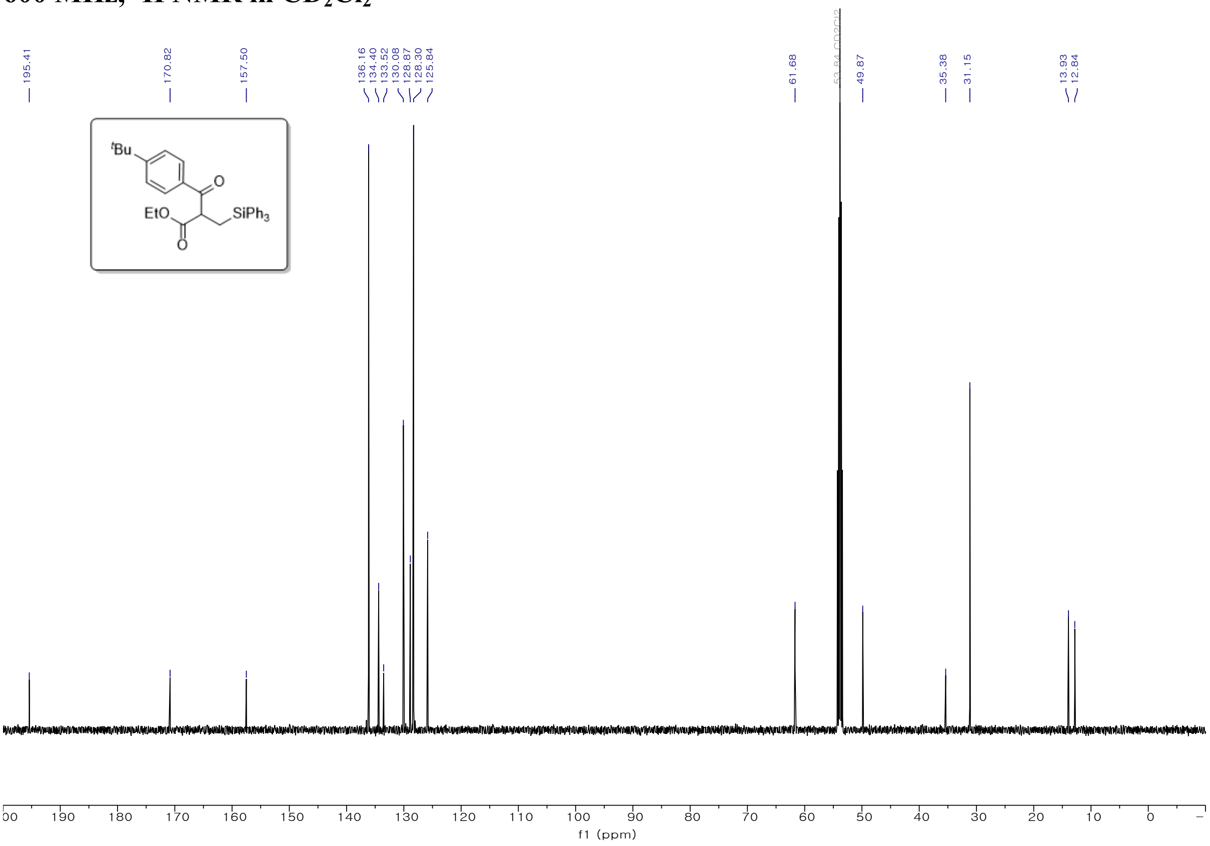

125 MHz, ^{13}C NMR in CD_2Cl_2

ethyl 3-(4-(*tert*-butyl)phenyl)-2-((diisopropyl(*o*-tolyl)silyl)methyl)-3-oxopropanoate (4i).

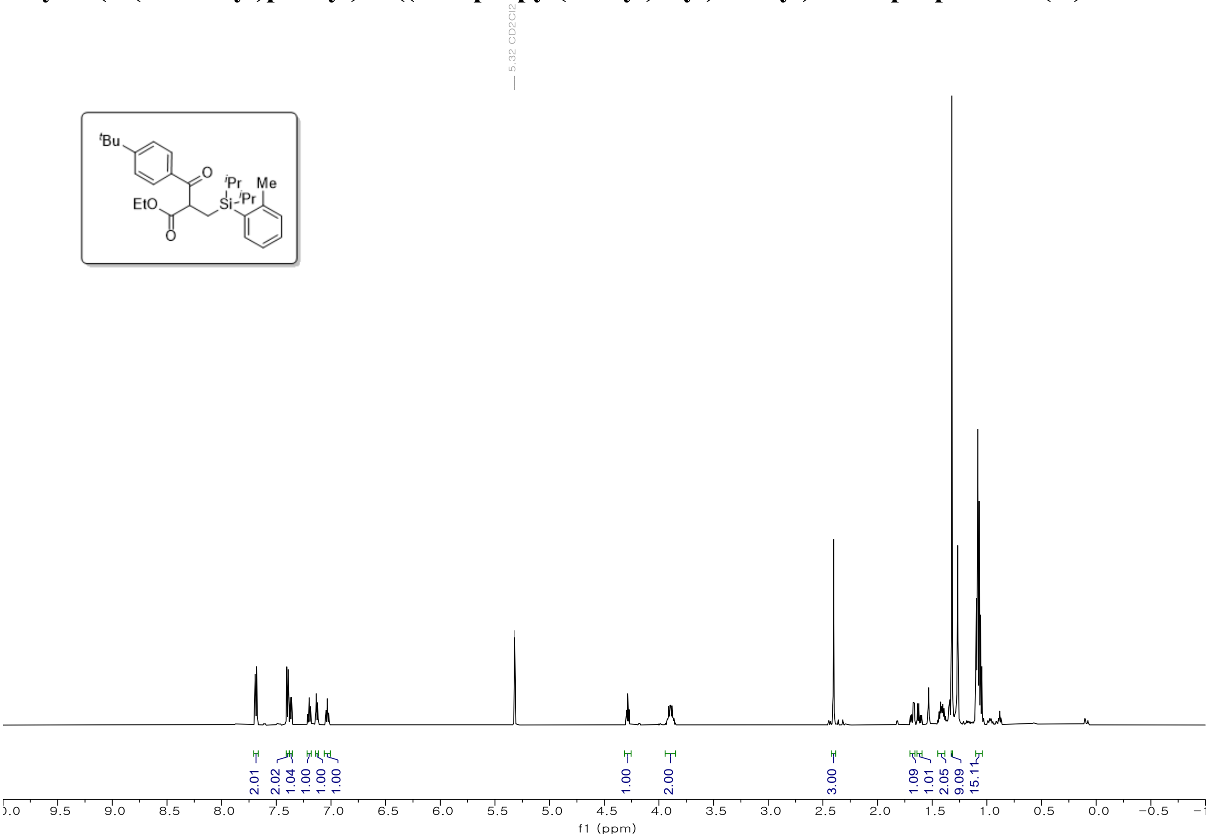

600 MHz, ^{1}H NMR in CD_2Cl_2

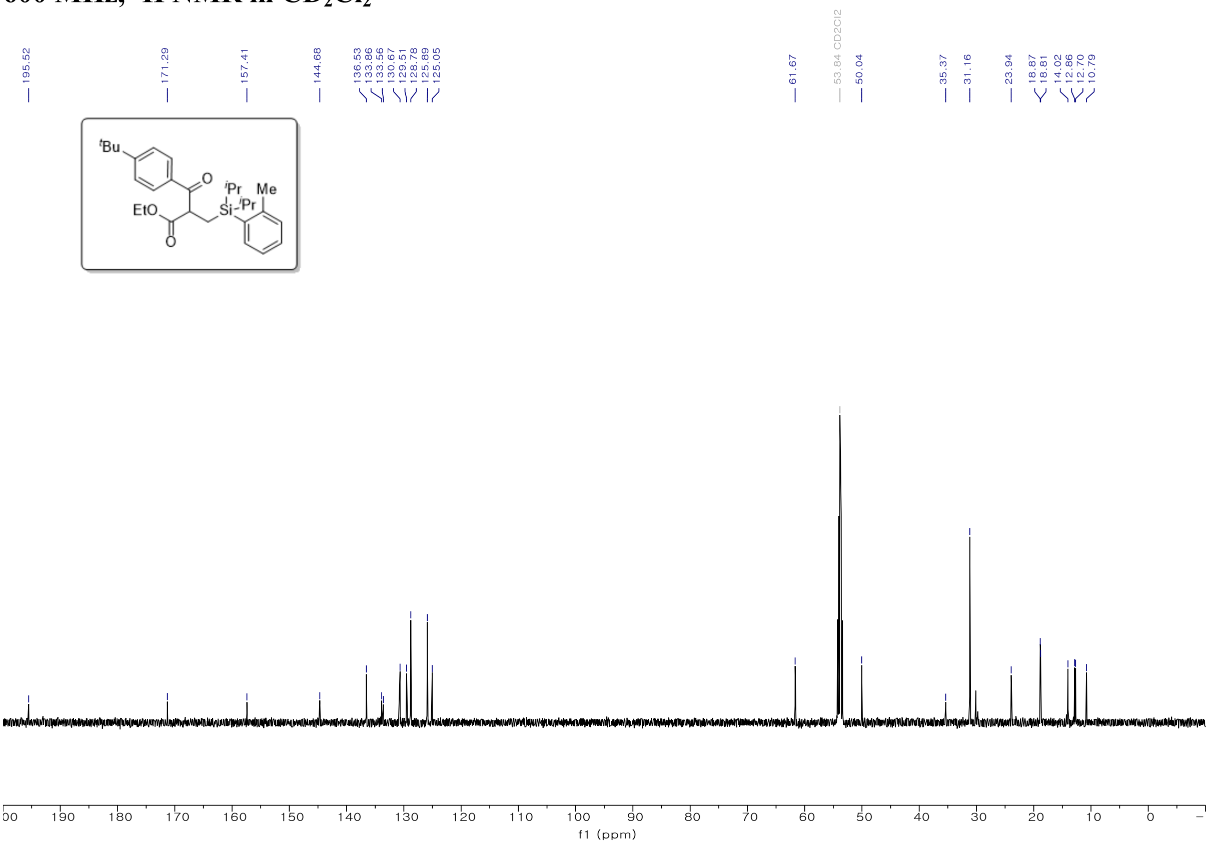

125 MHz, ^{13}C NMR in CD_2Cl_2

ethyl 3-(4-(*tert*-butyl)phenyl)-2-(((4-methoxyphenyl)dimethylsilyl)methyl)-3-oxopropanoate (4j).

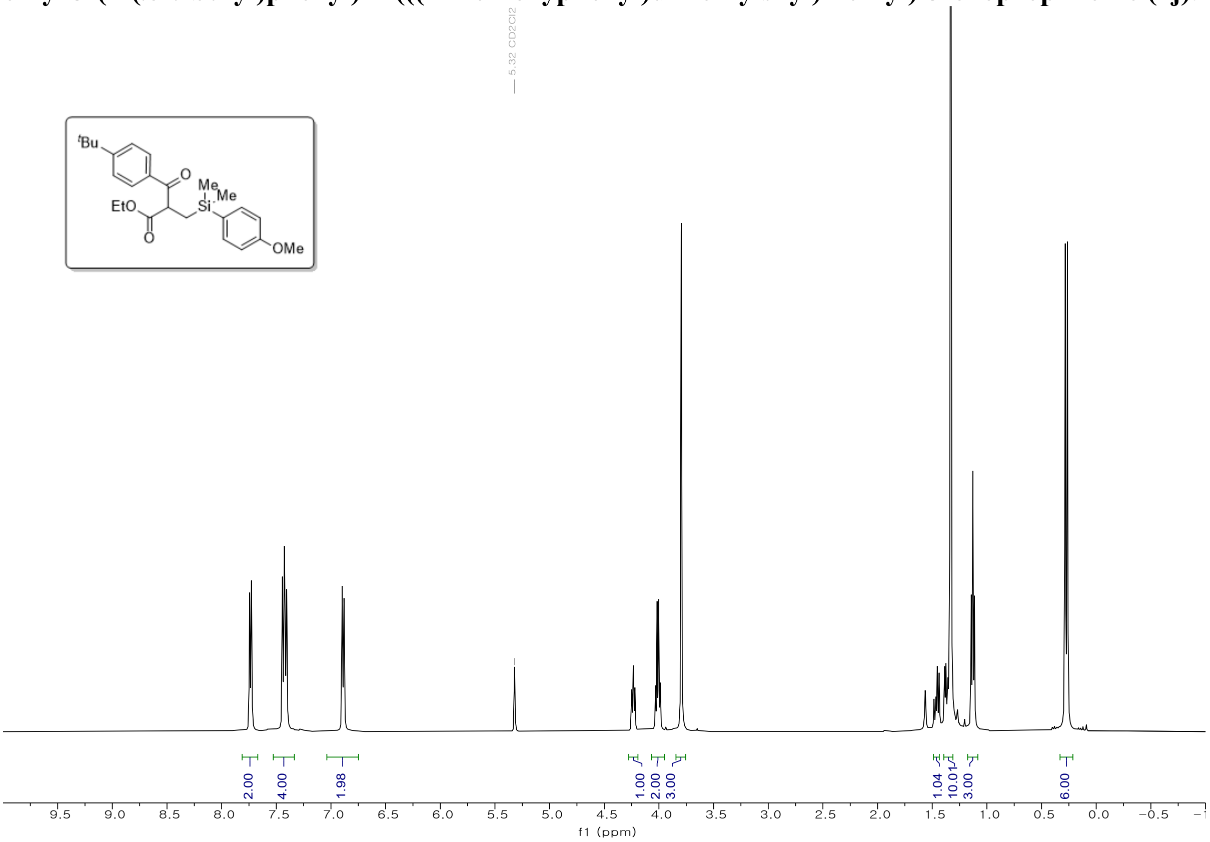

500 MHz, ^{1}H NMR in CD_2Cl_2

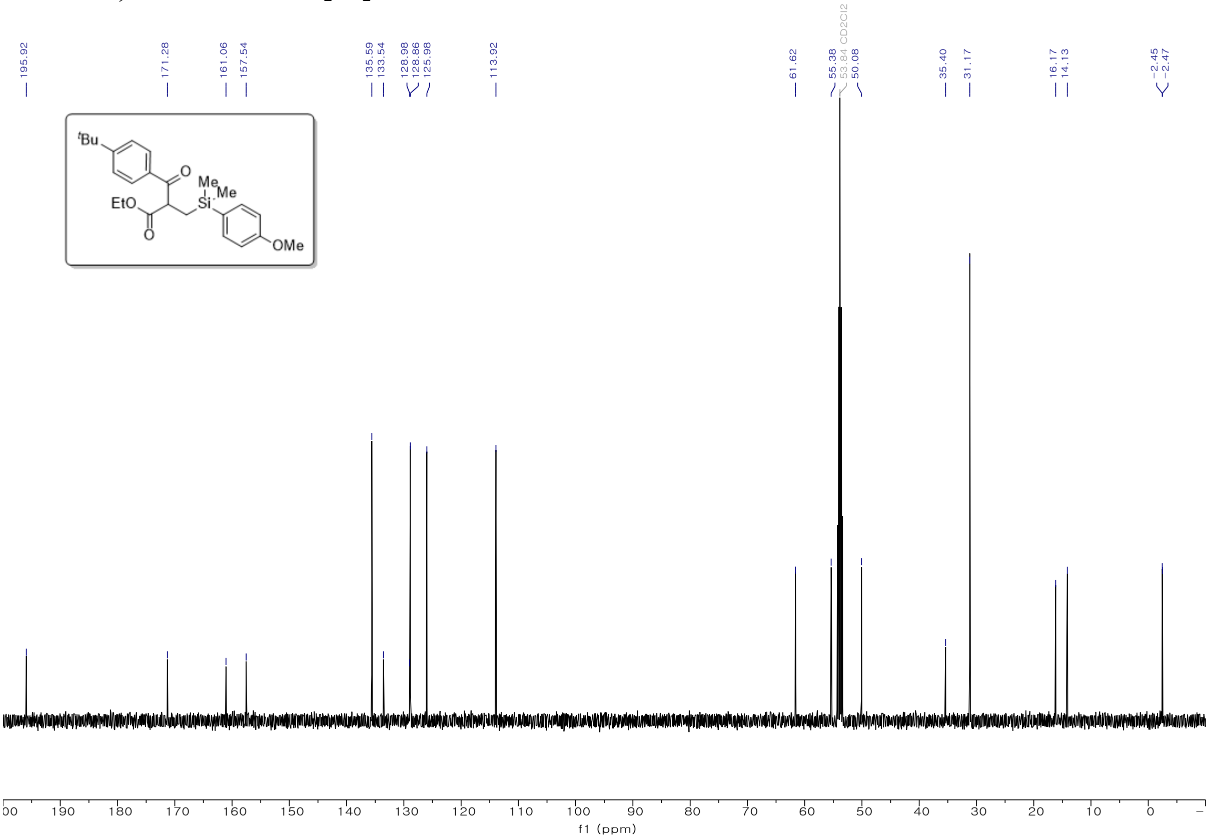

125 MHz, ^{13}C NMR in CD_2Cl_2

ethyl 3-(4-(*tert*-butyl)phenyl)-2-(((4-methoxyphenyl)diphenylsilyl)methyl)-3-oxopropanoate (4k).

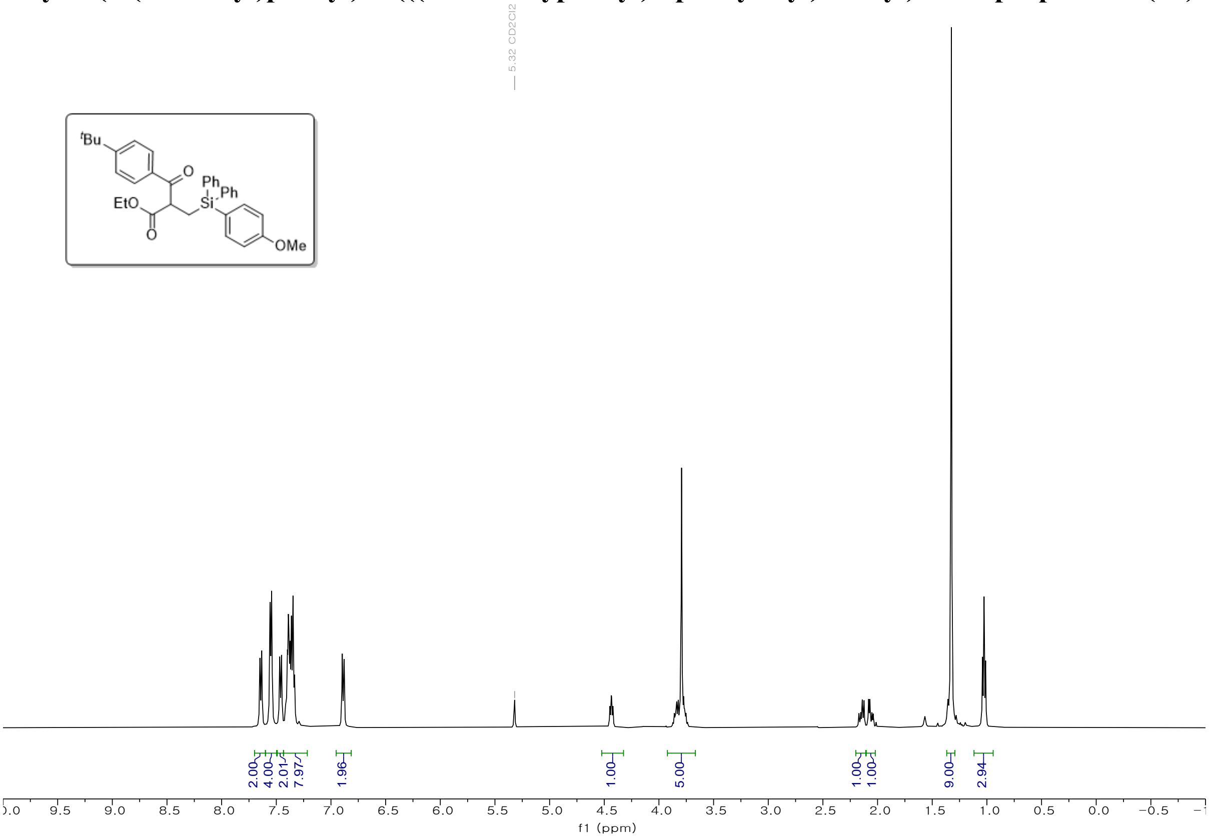

500 MHz, ^{1}H NMR in CD_2Cl_2

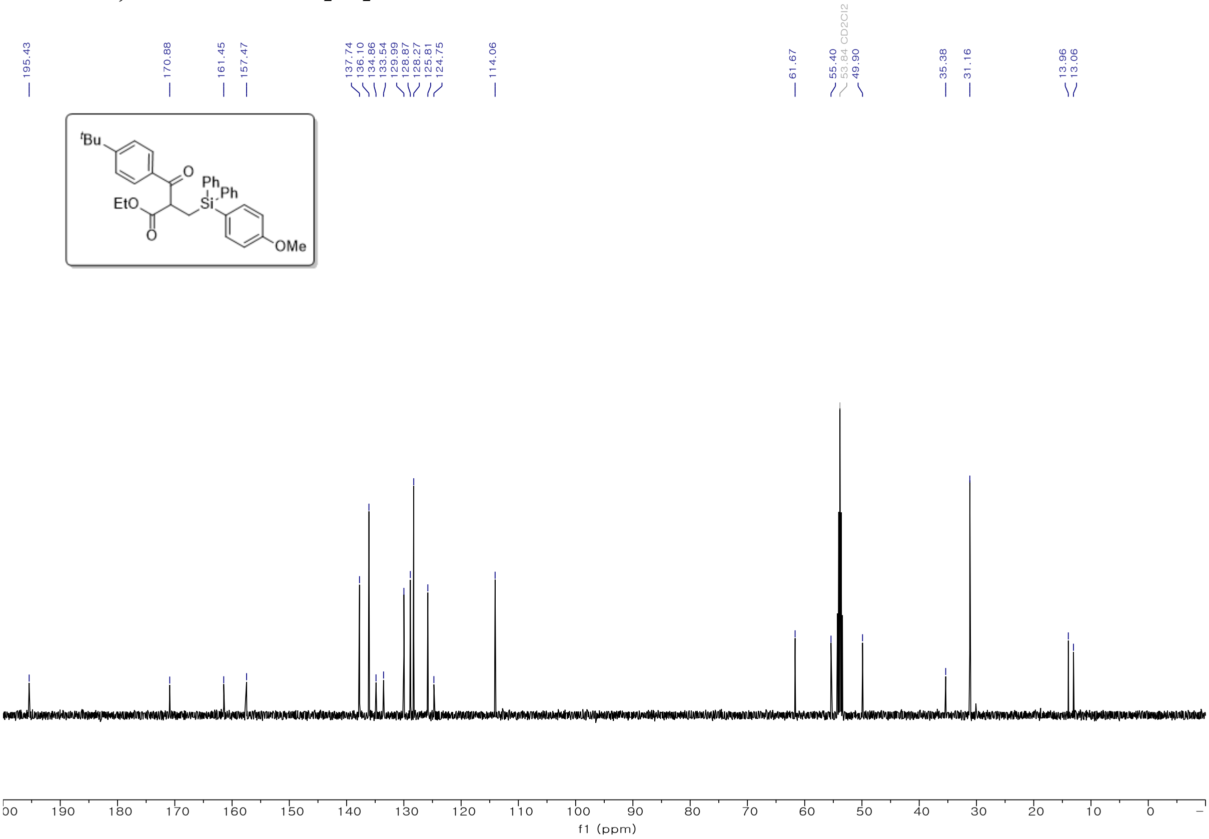

125 MHz, ^{13}C NMR in CD_2Cl_2

ethyl 3-(4-(*tert*-butyl)phenyl)-2-(((4-(*tert*-butyl)phenyl)diphenylsilyl)methyl)-3-oxopropanoate (4l).

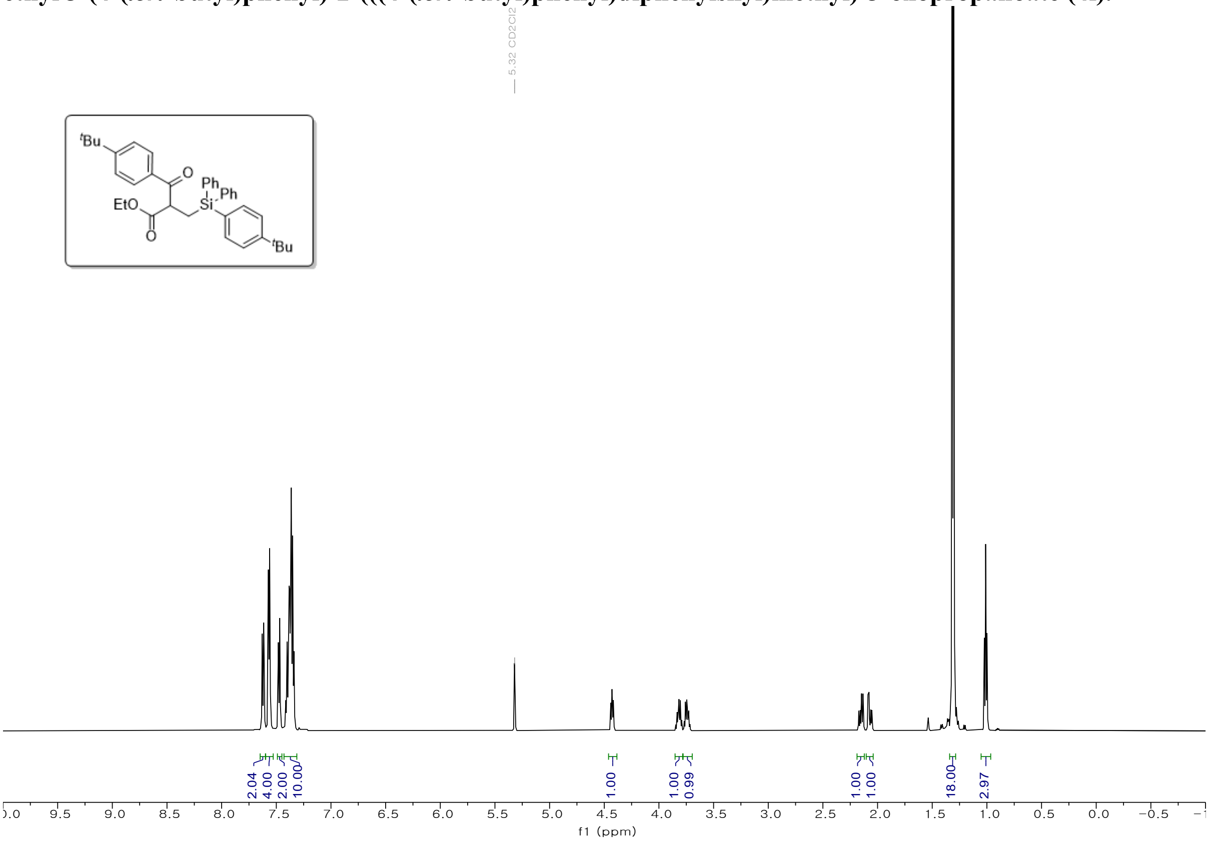

600 MHz, ^{1}H NMR in CD_2Cl_2

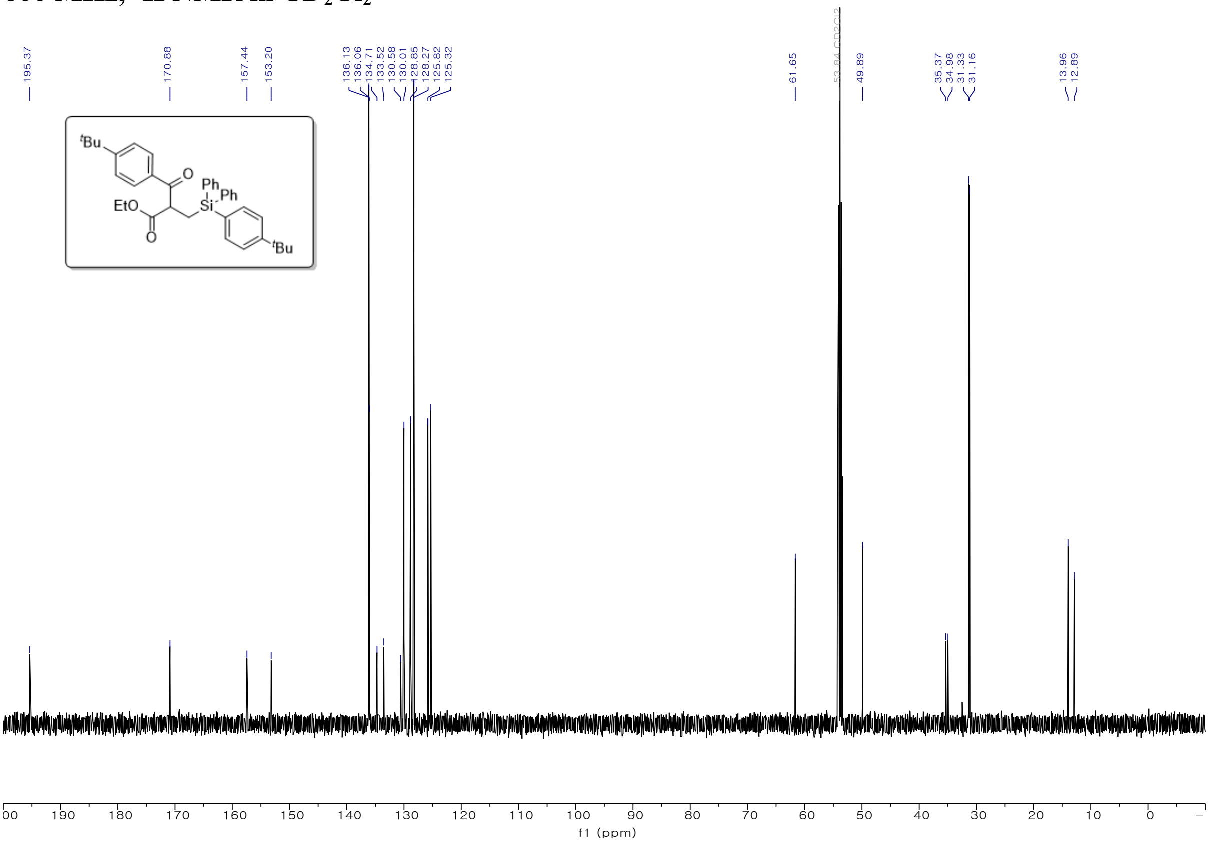

125 MHz, ^{13}C NMR in CD_2Cl_2

ethyl 3-(4-(*tert*-butyl)phenyl)-2-((diisopropyl(4-(trimethylsilyl)phenyl)silyl)methyl)-3-oxopropanoate (4m).

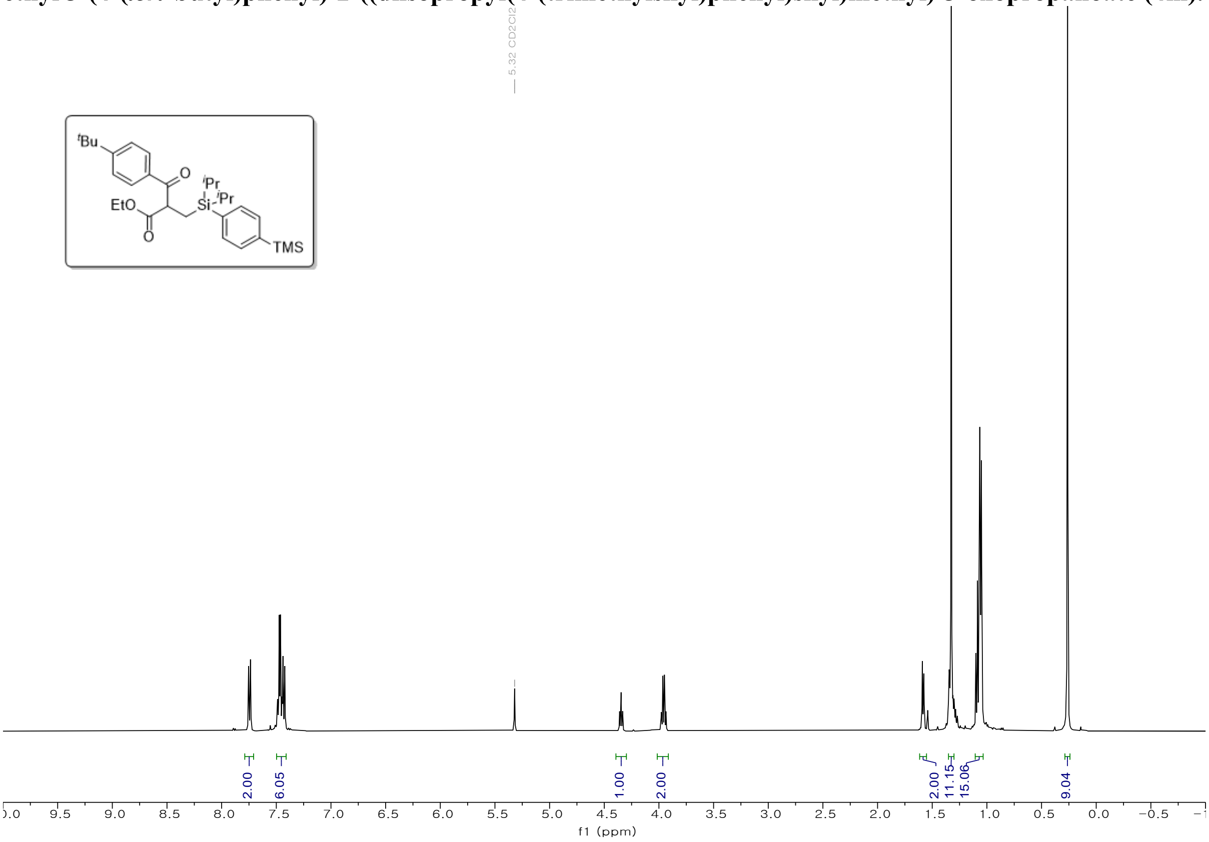

500 MHz, ^{1}H NMR in CD_2Cl_2

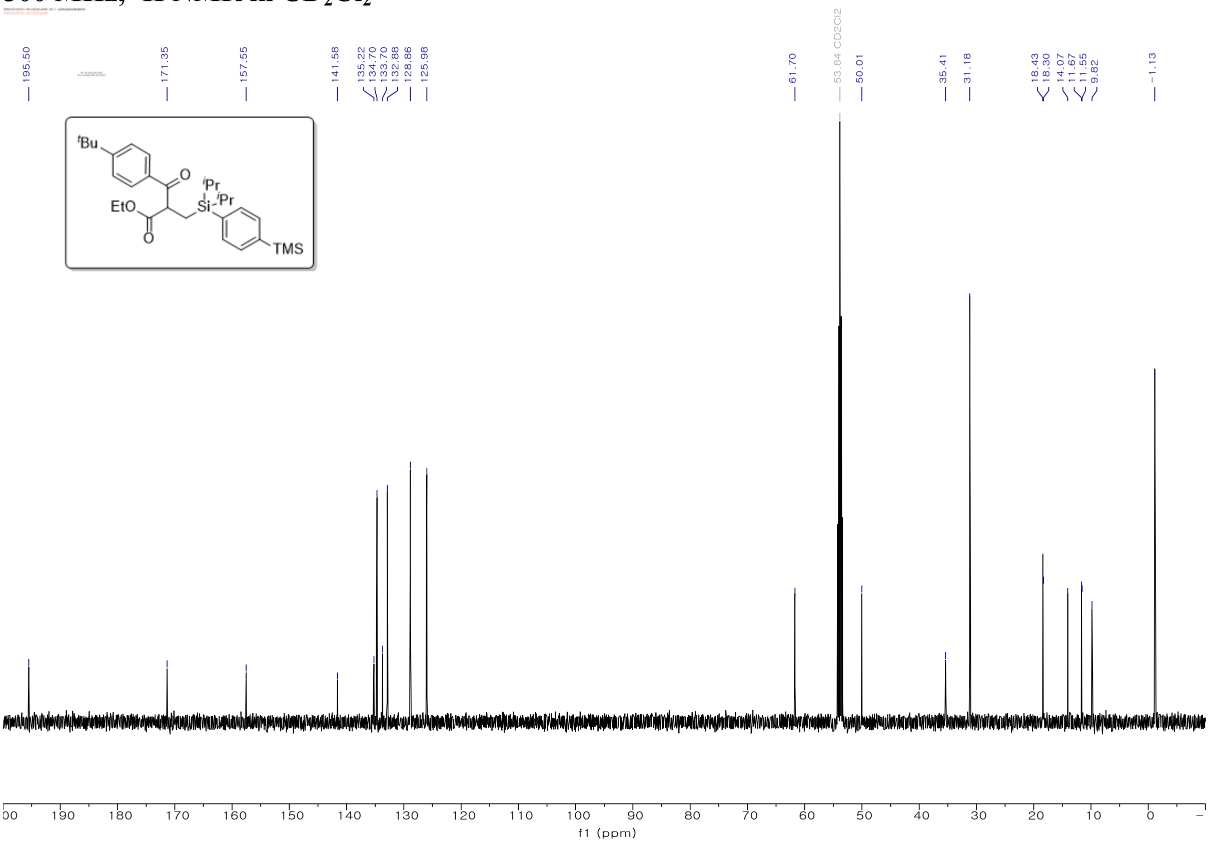

125 MHz, ^{13}C NMR in CD_2Cl_2

ethyl 3-(4-(*tert*-butyl)phenyl)-2-((diphenyl(4-(trifluoromethyl)phenyl)silyl)methyl)-3-oxopropanoate (4n).

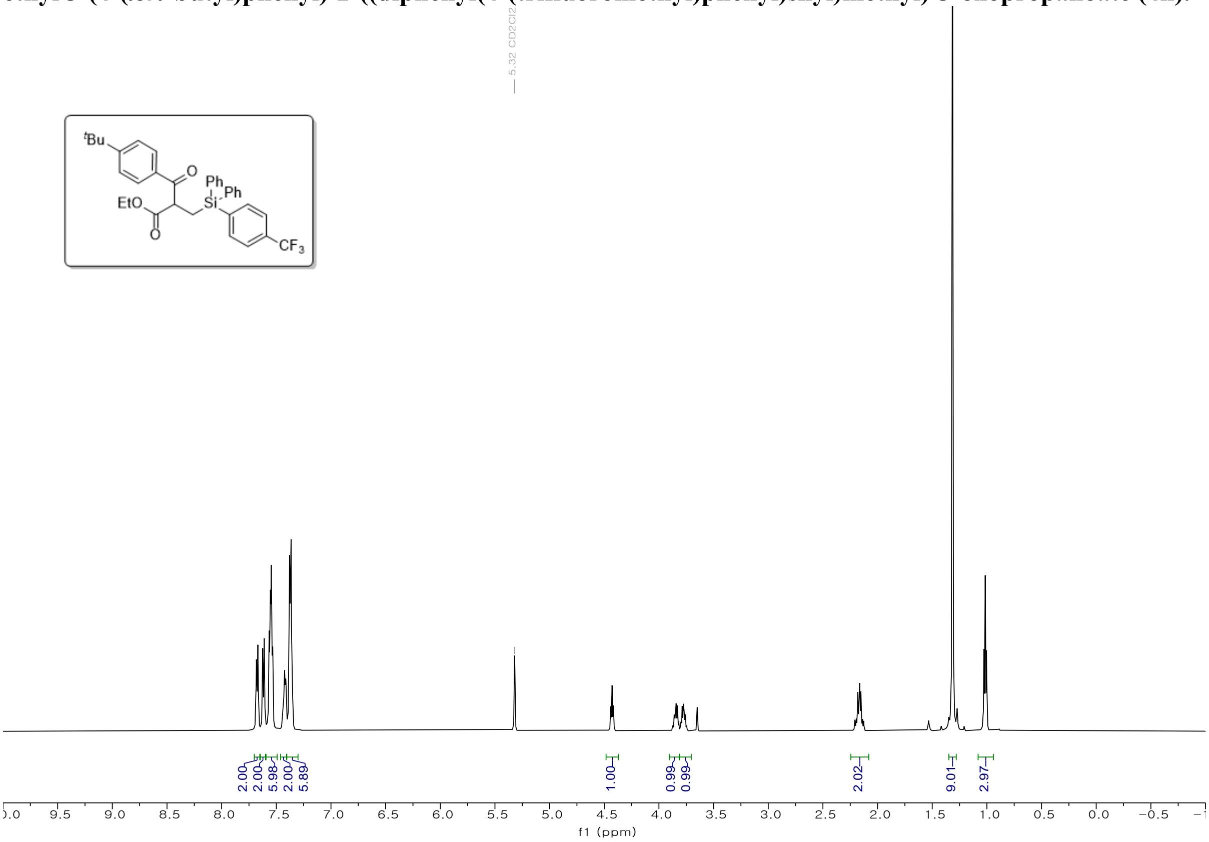

600 MHz, ^{1}H NMR in CD_2Cl_2

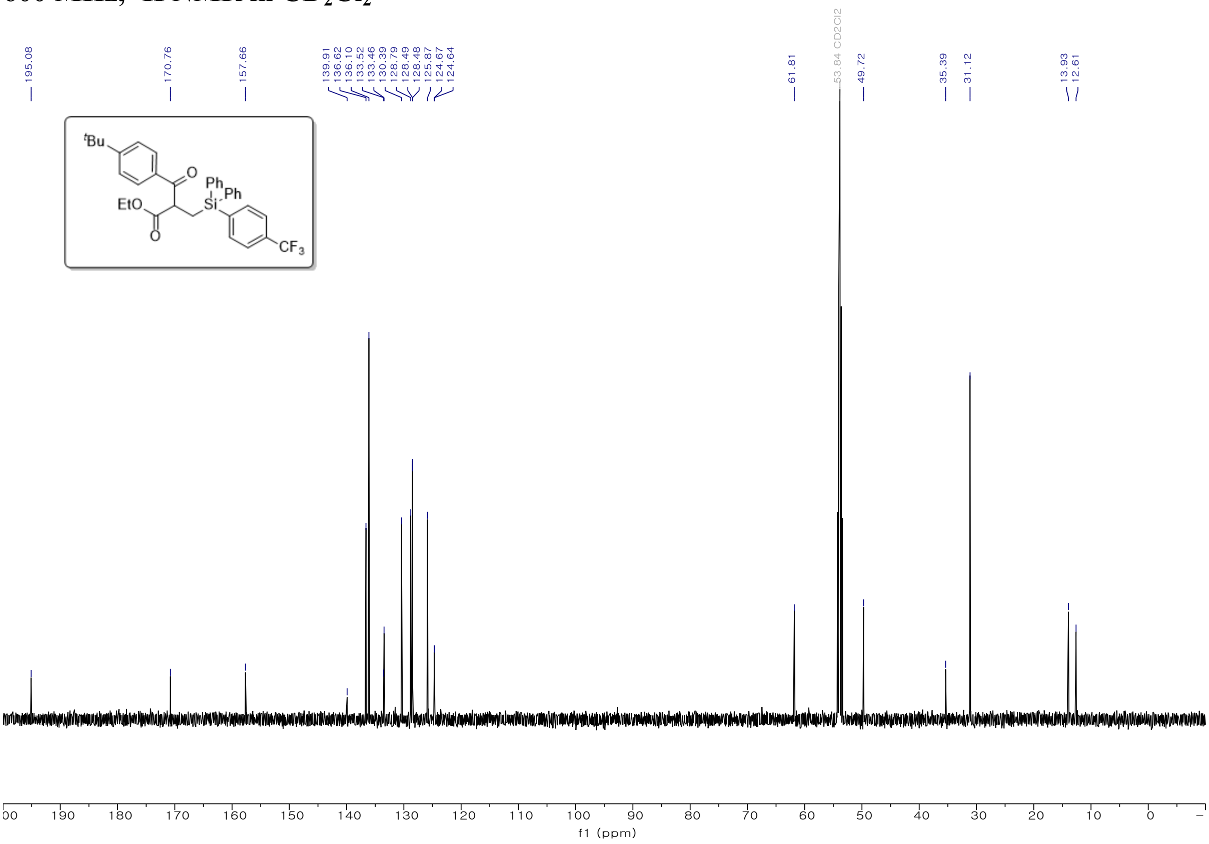

125 MHz, ^{13}C NMR in CD_2Cl_2

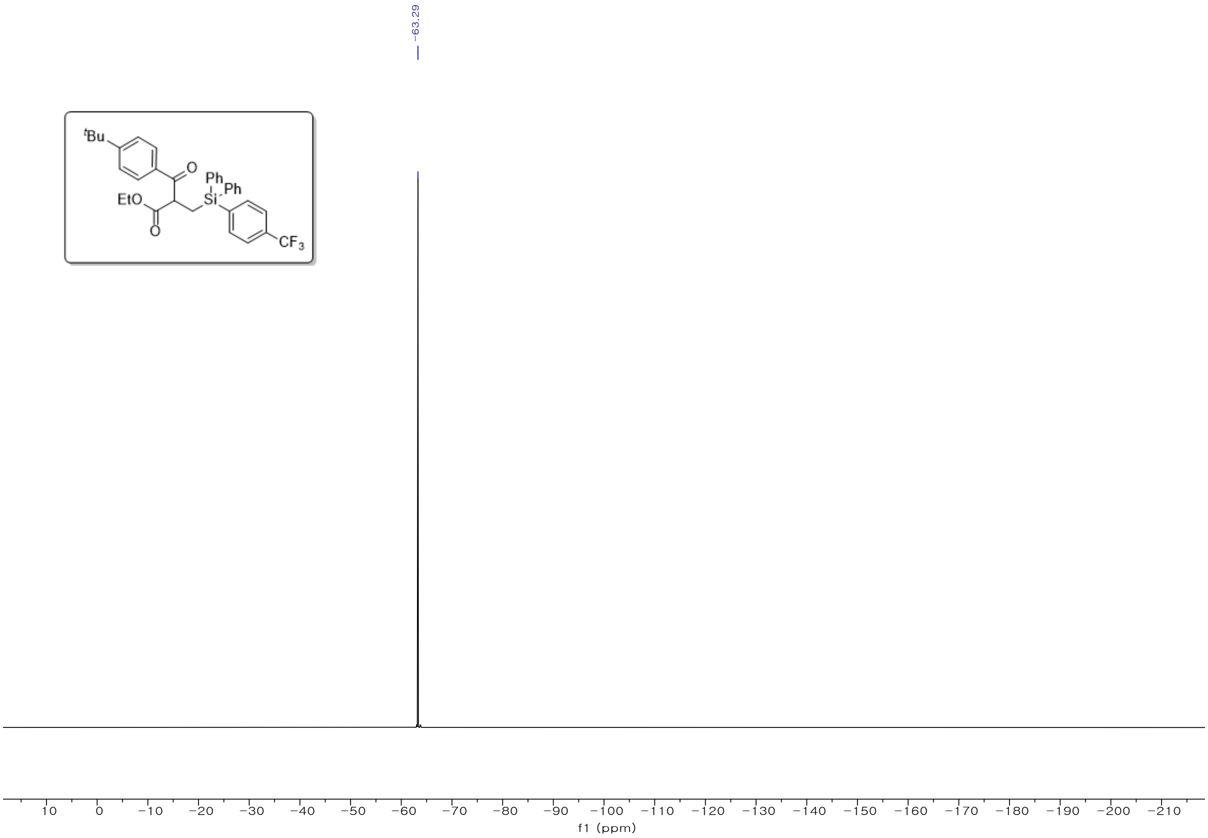

376 MHz, ^{19}F NMR in CD_2Cl_2

ethyl 3-(4-(*tert*-butyl)phenyl)-2-(((4-cyanophenyl)diisopropylsilyl)methyl)-3-oxopropanoate (4o).

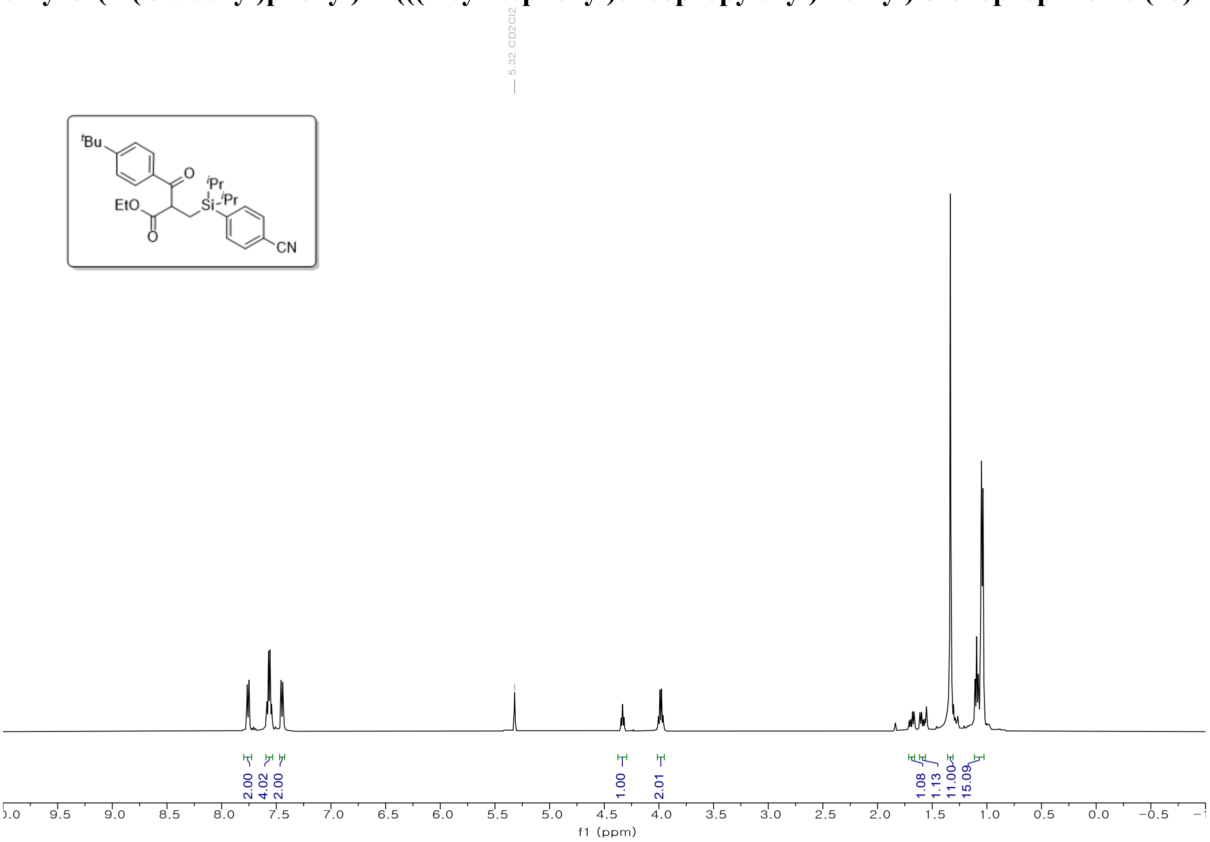

500 MHz, ^{1}H NMR in CD_2Cl_2

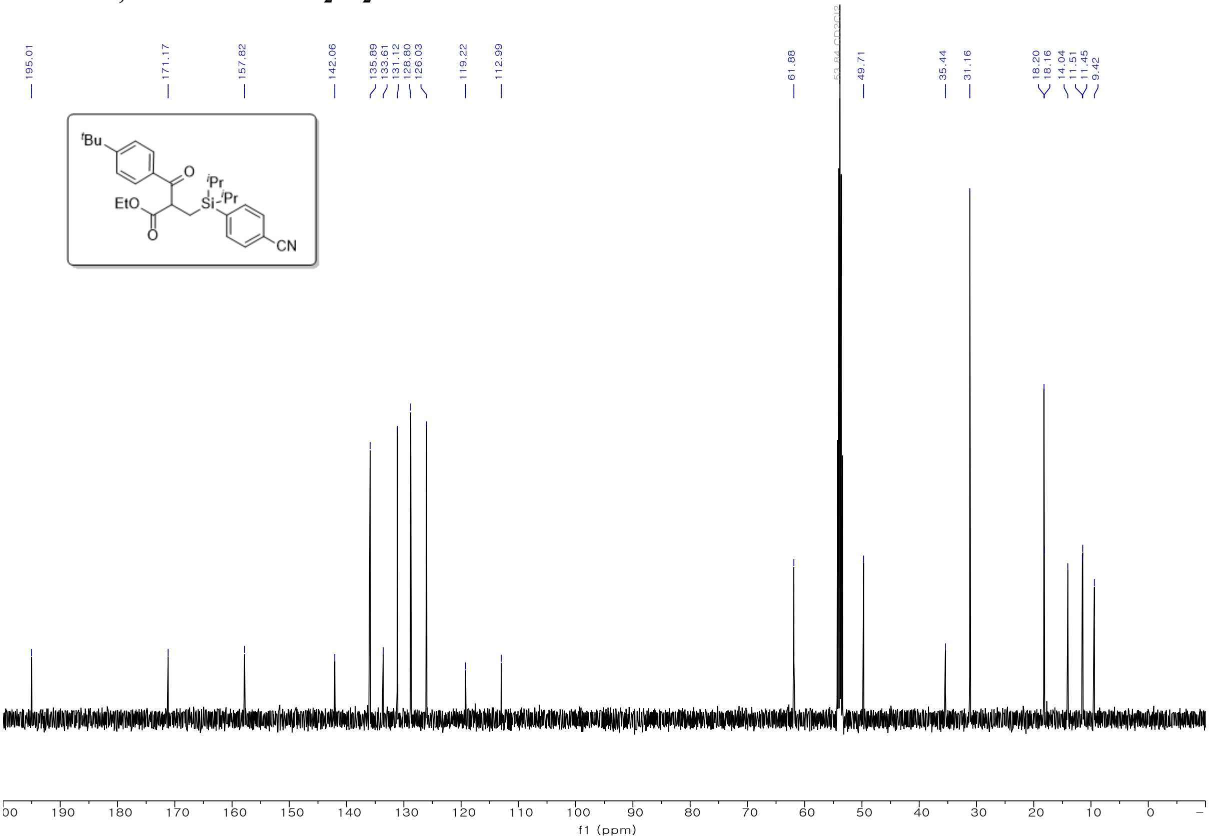

125 MHz, ^{13}C NMR in CD_2Cl_2

ethyl 3-(4-(*tert*-butyl)phenyl)-2-(((4-fluorophenyl)diphenylsilyl)methyl)-3-oxopropanoate (4p).

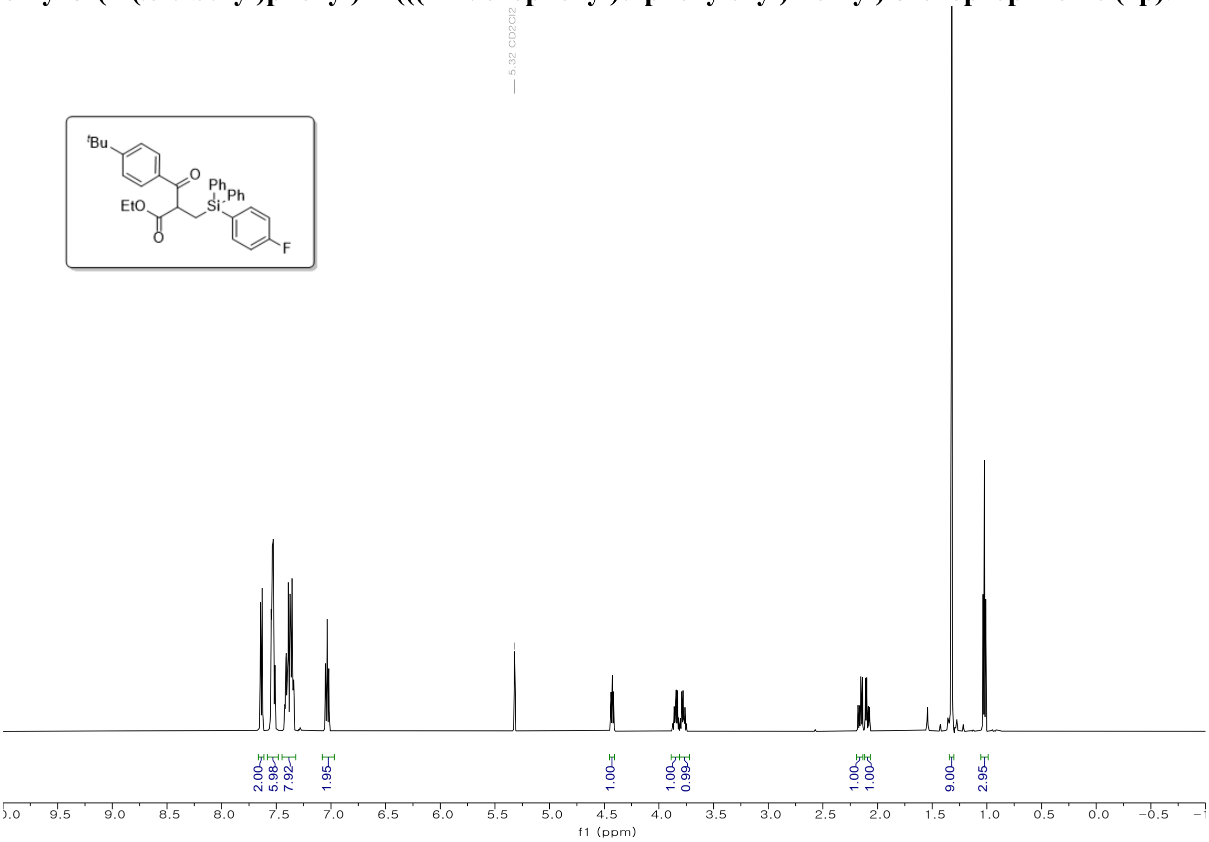

600 MHz, ^{1}H NMR in CD_2Cl_2

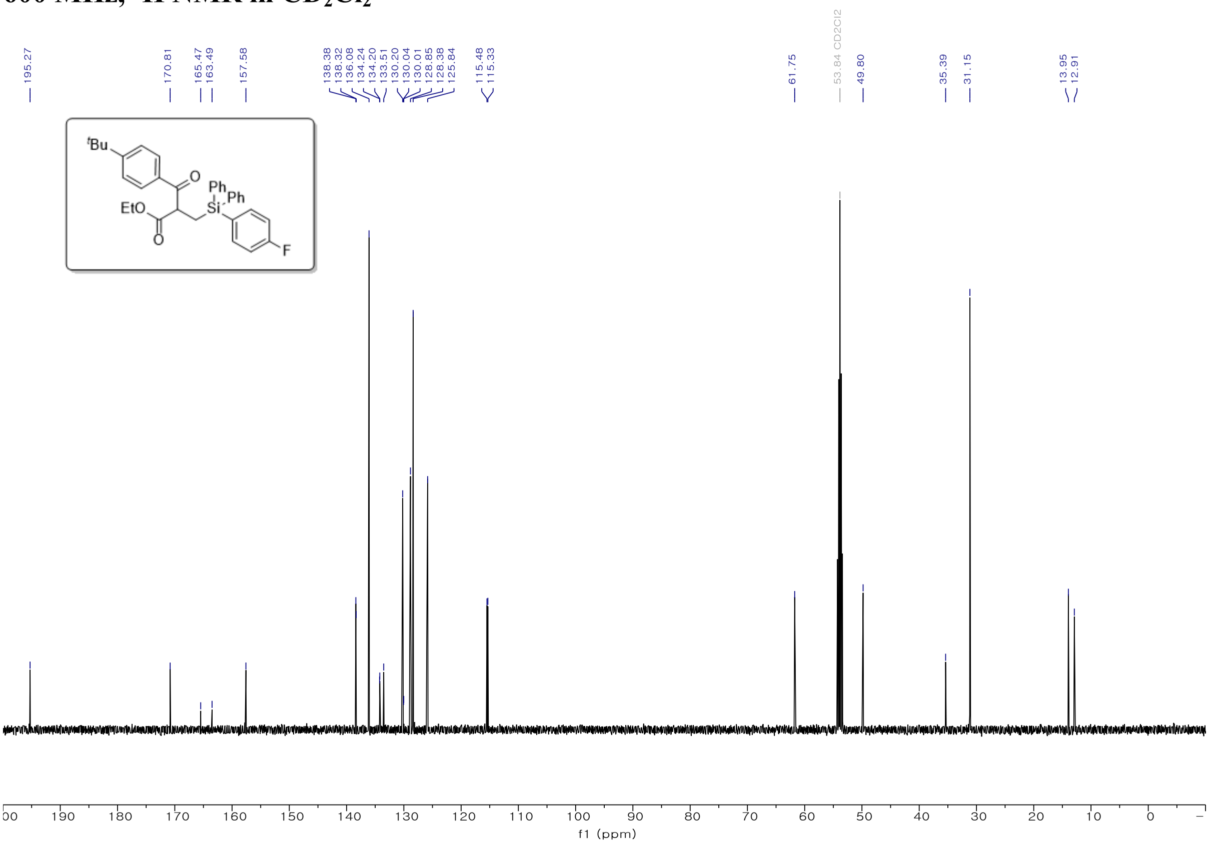

125 MHz, ^{13}C NMR in CD_2Cl_2

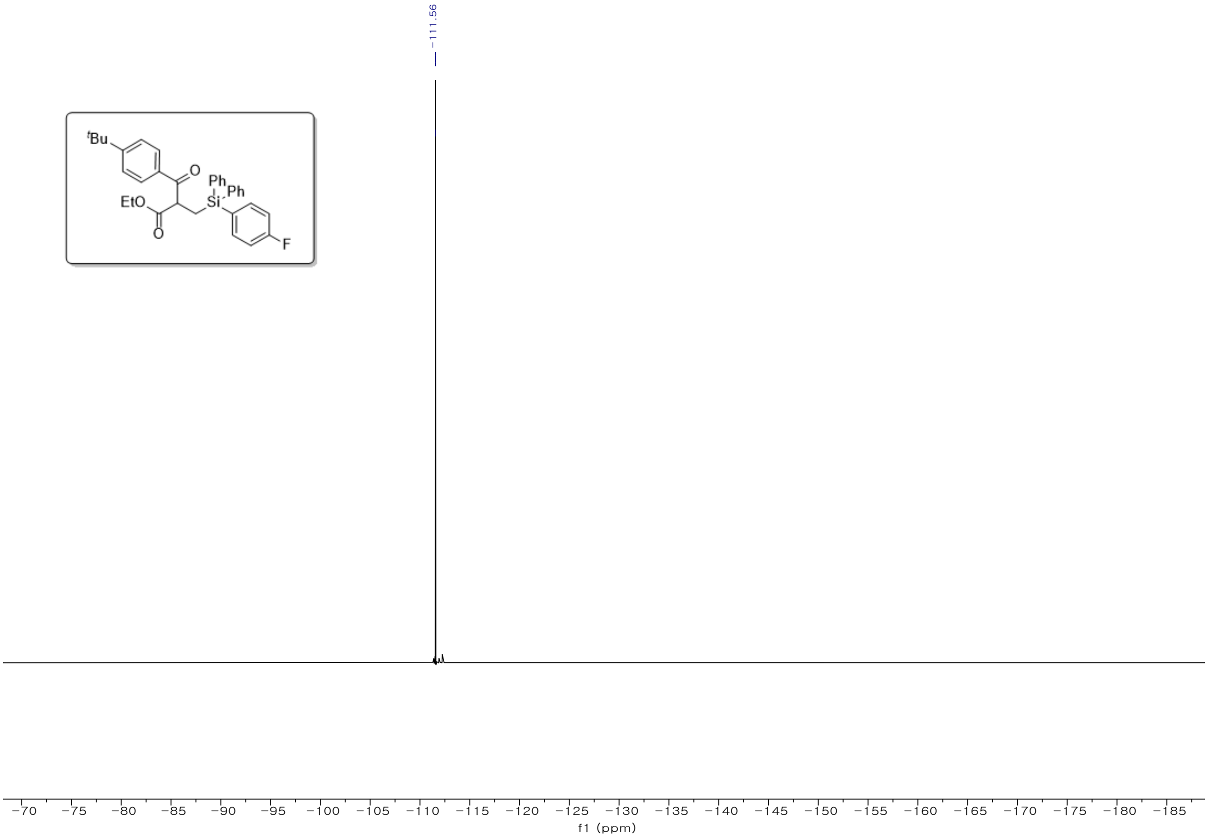

471 MHz, ^{19}F NMR in CD_2Cl_2

ethyl 3-(4-(*tert*-butyl)phenyl)-2-(((3-chlorophenyl)diphenylsilyl)methyl)-3-oxopropanoate (4q).

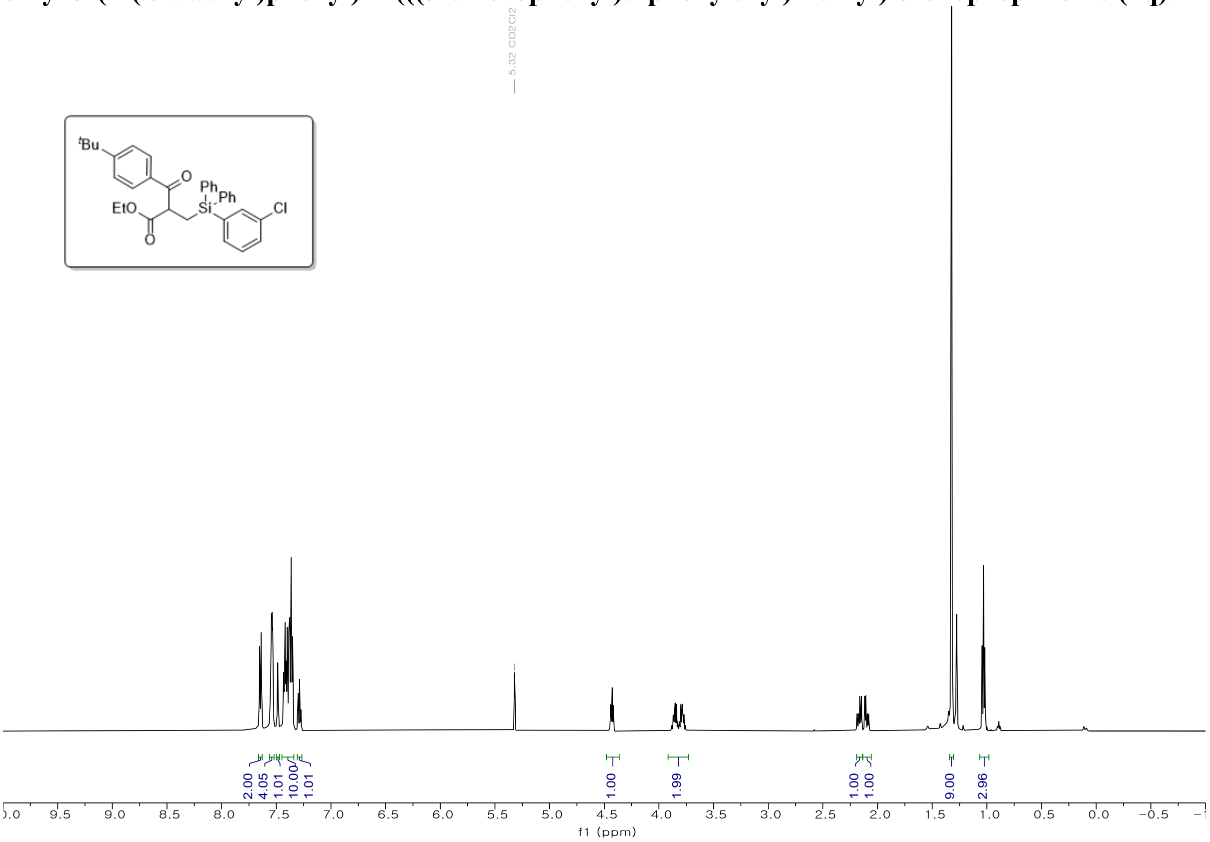

600 MHz, ^{1}H NMR in CD_2Cl_2

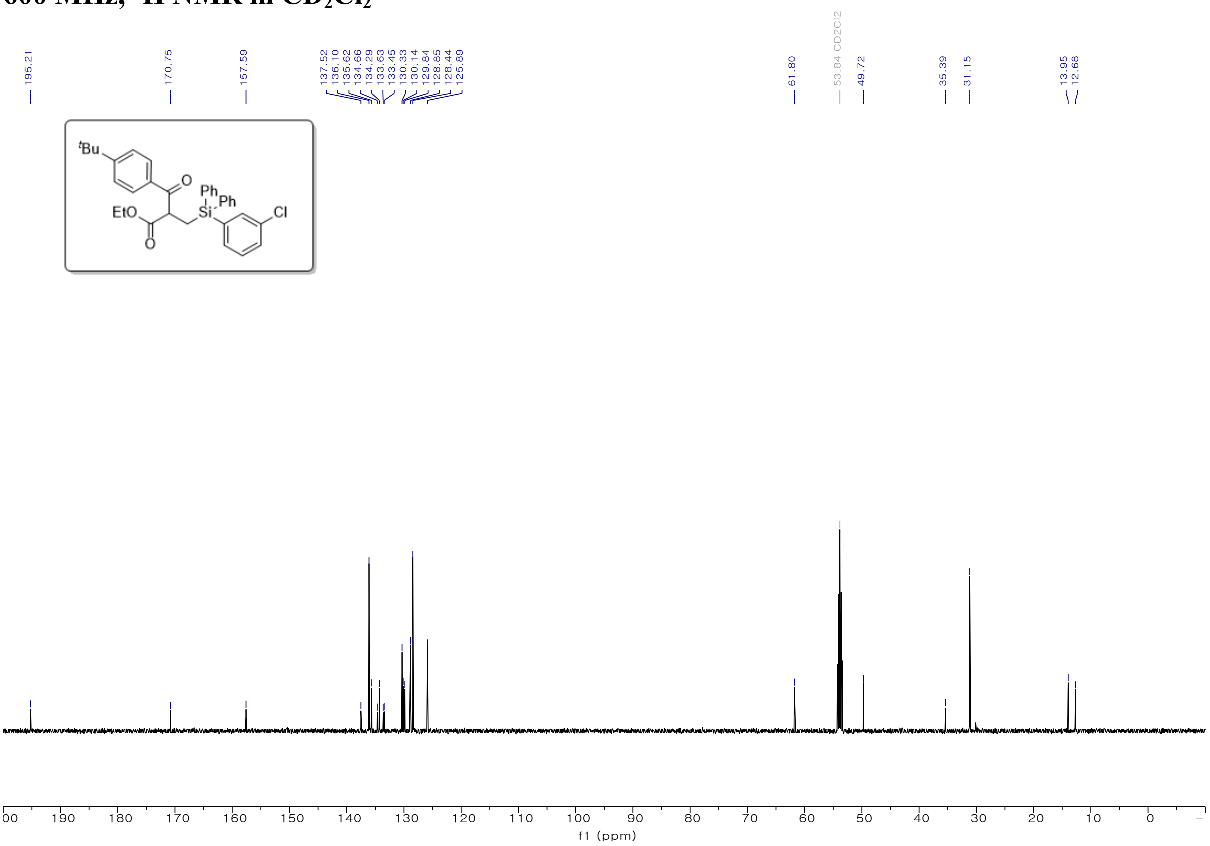

125 MHz, ^{13}C NMR in CD_2Cl_2

ethyl 3-(4-(*tert*-butyl)phenyl)-2-((diisopropyl(phenylethynyl)silyl)methyl)-3-oxopropanoate (4r).

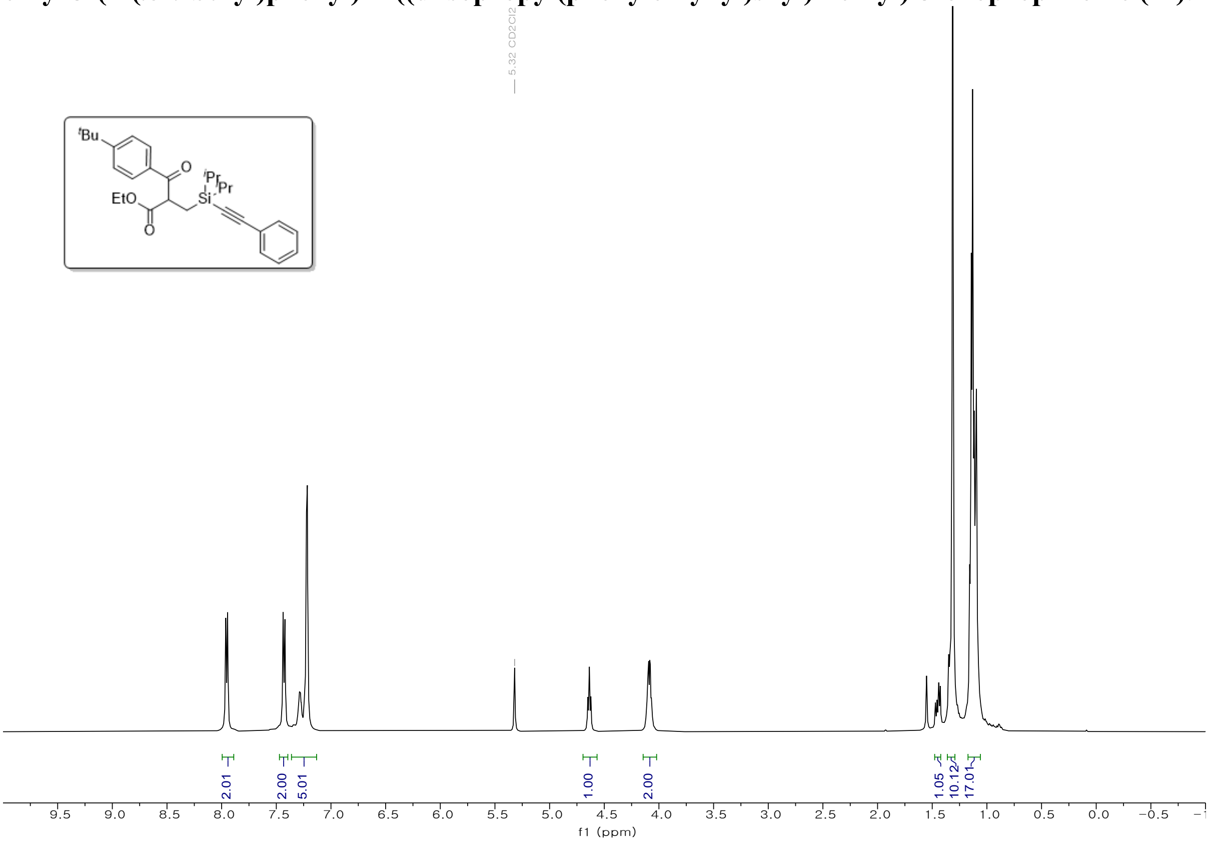

500 MHz, ^{1}H NMR in CD_2Cl_2

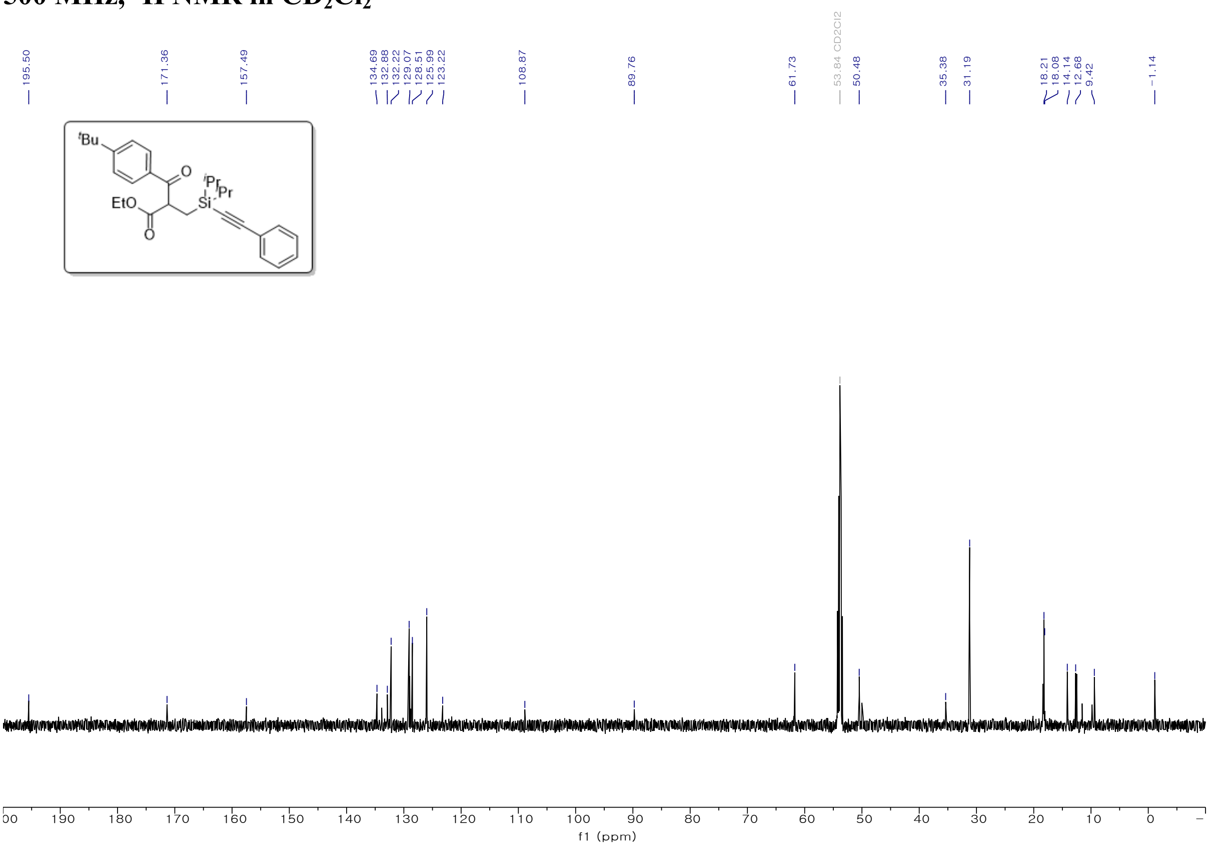

125 MHz, ^{13}C NMR in CD_2Cl_2

ethyl 3-(4-(*tert*-butyl)phenyl)-2-(((4-((2-(4-isobutylphenyl)propanoyl)oxy)phenyl)diphenylsilyl) methyl)-3-oxopropanoate (4s).

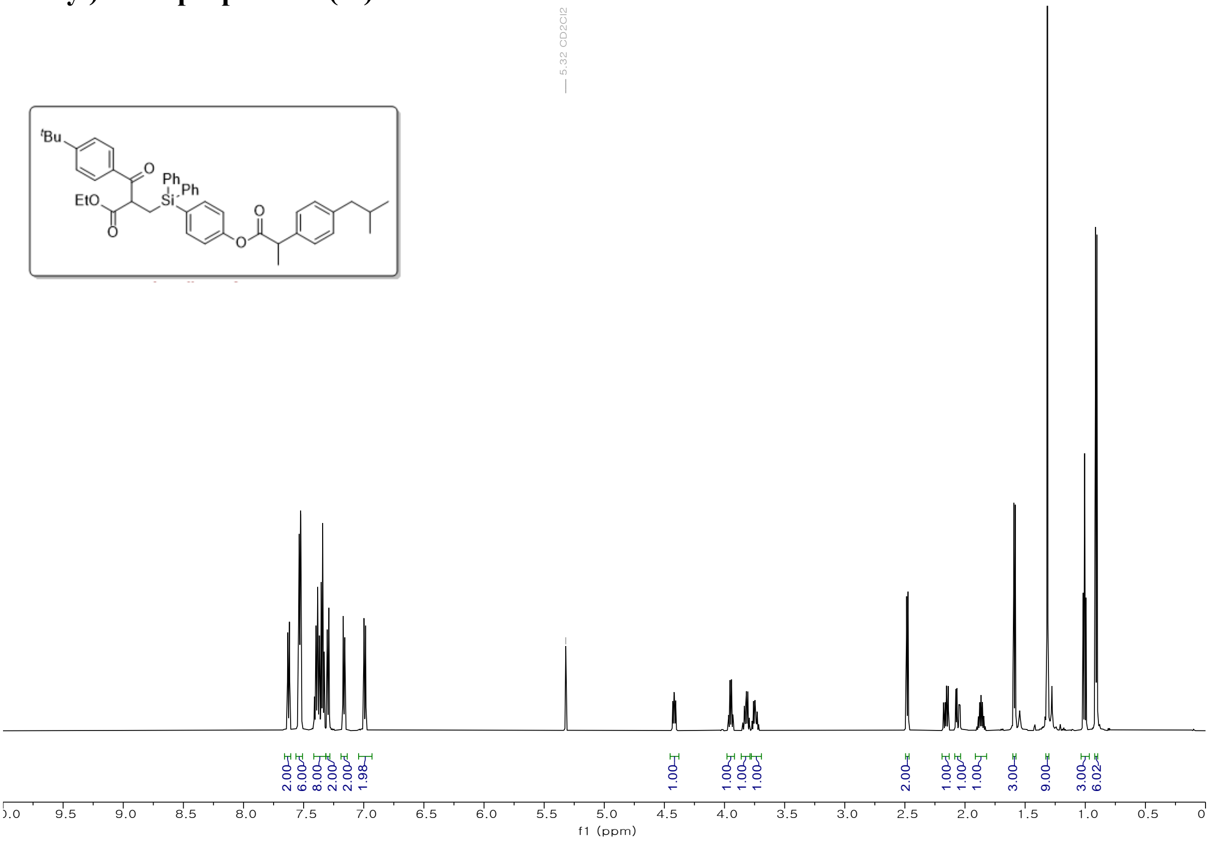

600 MHz, ^{1}H NMR in CD_2Cl_2

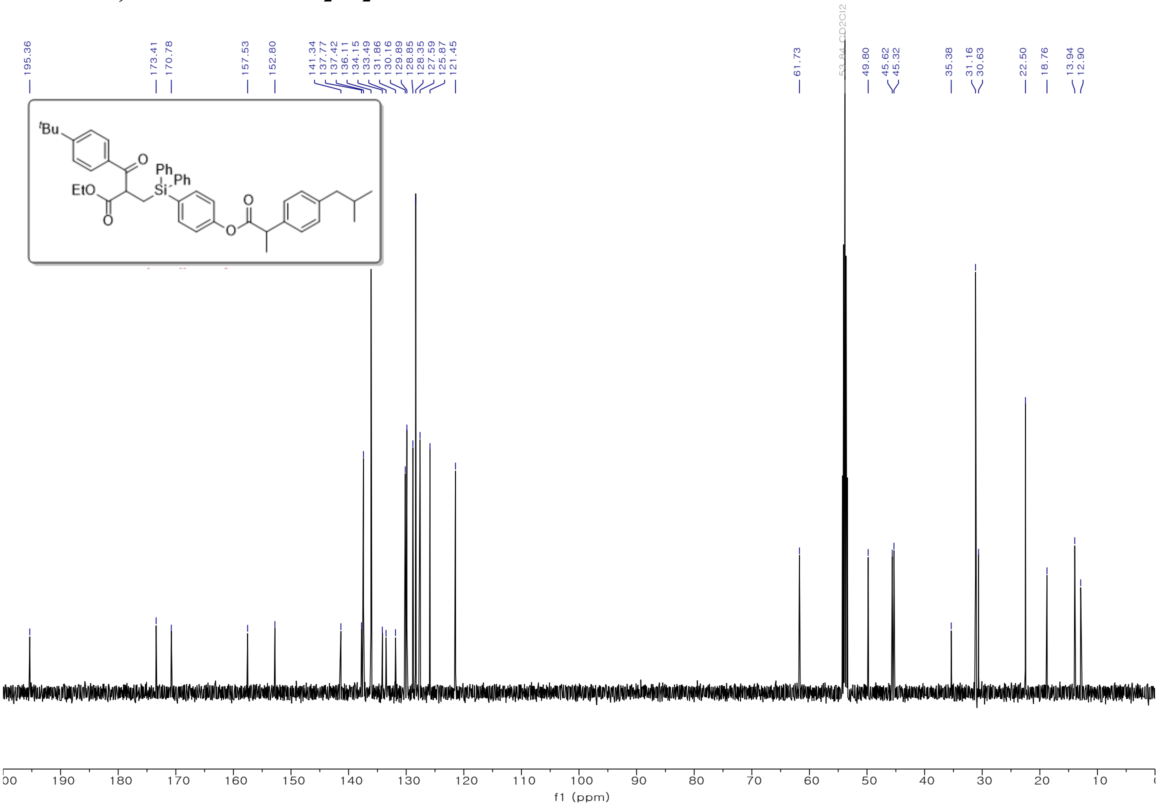

125 MHz, ^{13}C NMR in CD_2Cl_2

2-isopropyl-5-methylcyclohexyl 4-((2-(4-(*tert*-butyl)benzoyl)-3-ethoxy-3-oxopropyl)dimethylsilyl) benzoate (4t).

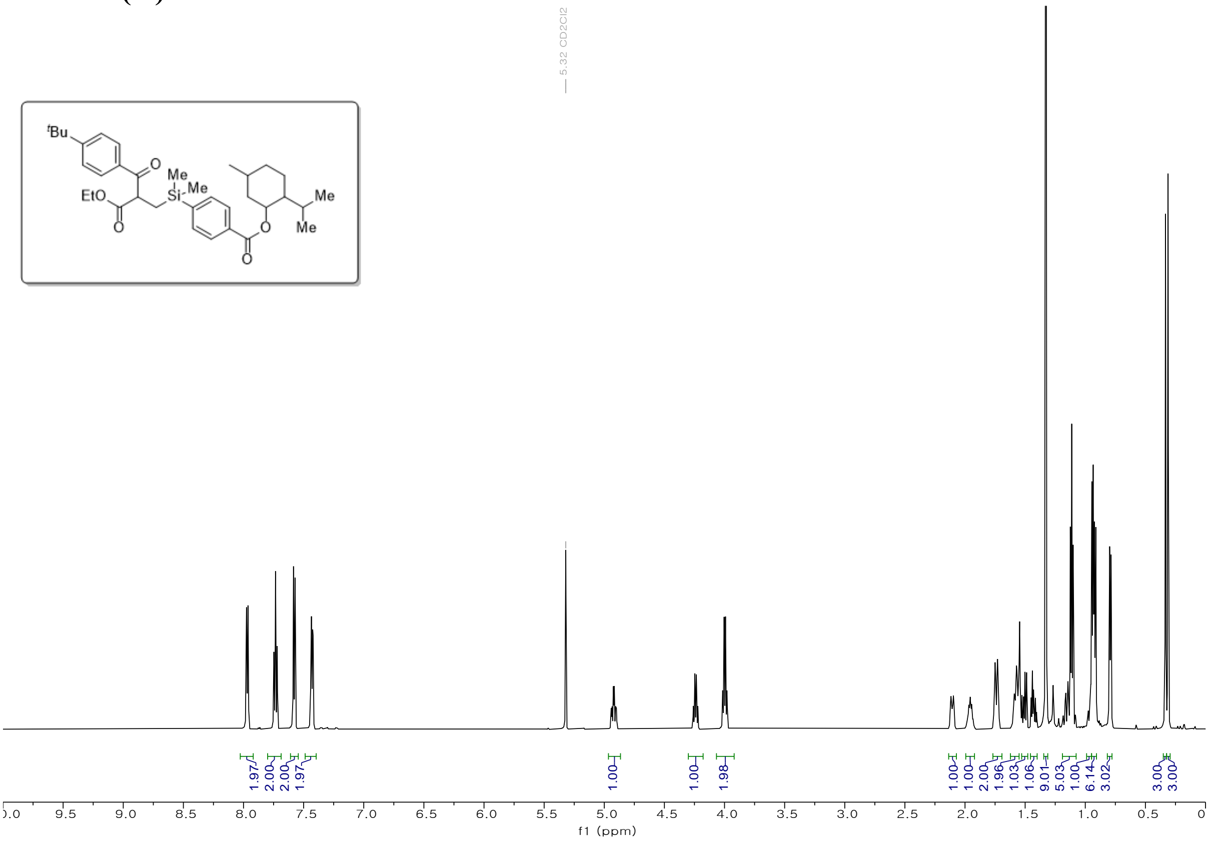

600 MHz, ^{1}H NMR in CD_2Cl_2

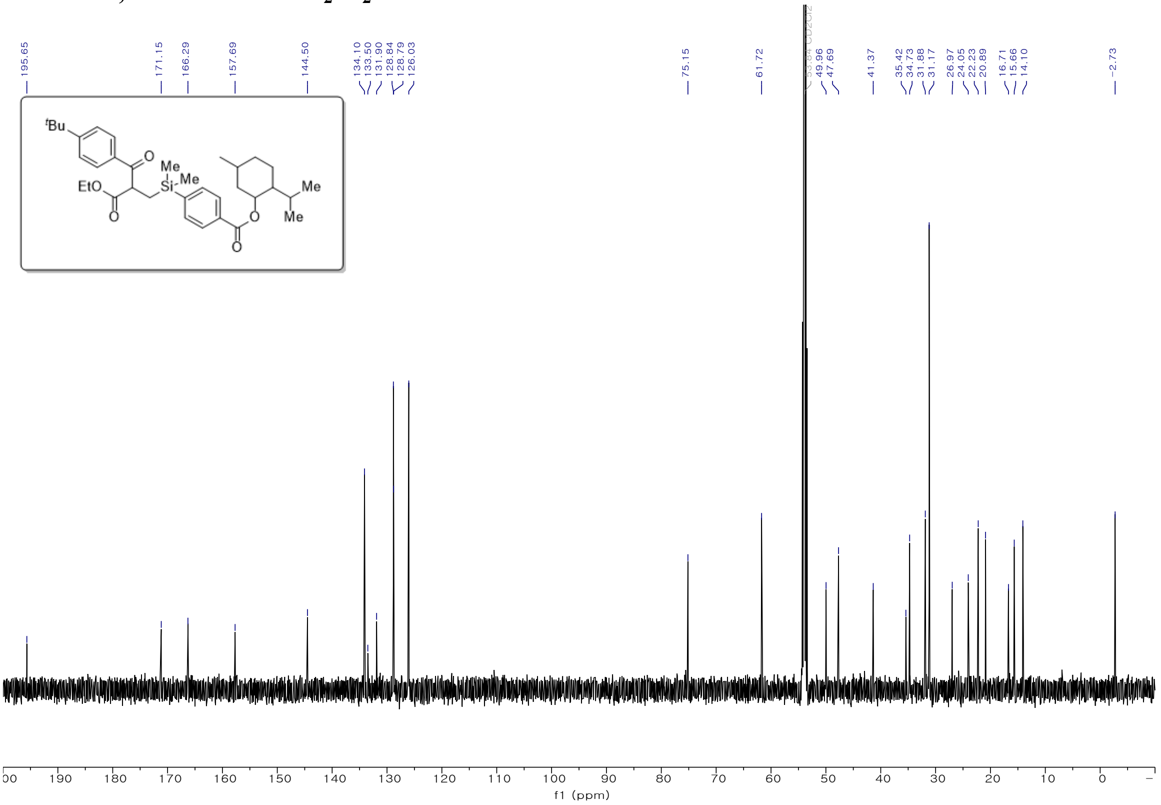

125 MHz, ^{13}C NMR in CD_2Cl_2

(1*S*,2*R*,4*S*)-1,7,7-trimethylbicyclo[2.2.1]heptan-2-yl 4-((2-(4-(*tert*-butyl)benzoyl)-3-ethoxy-3-oxo propyl)dimethylsilyl)benzoate (4u).

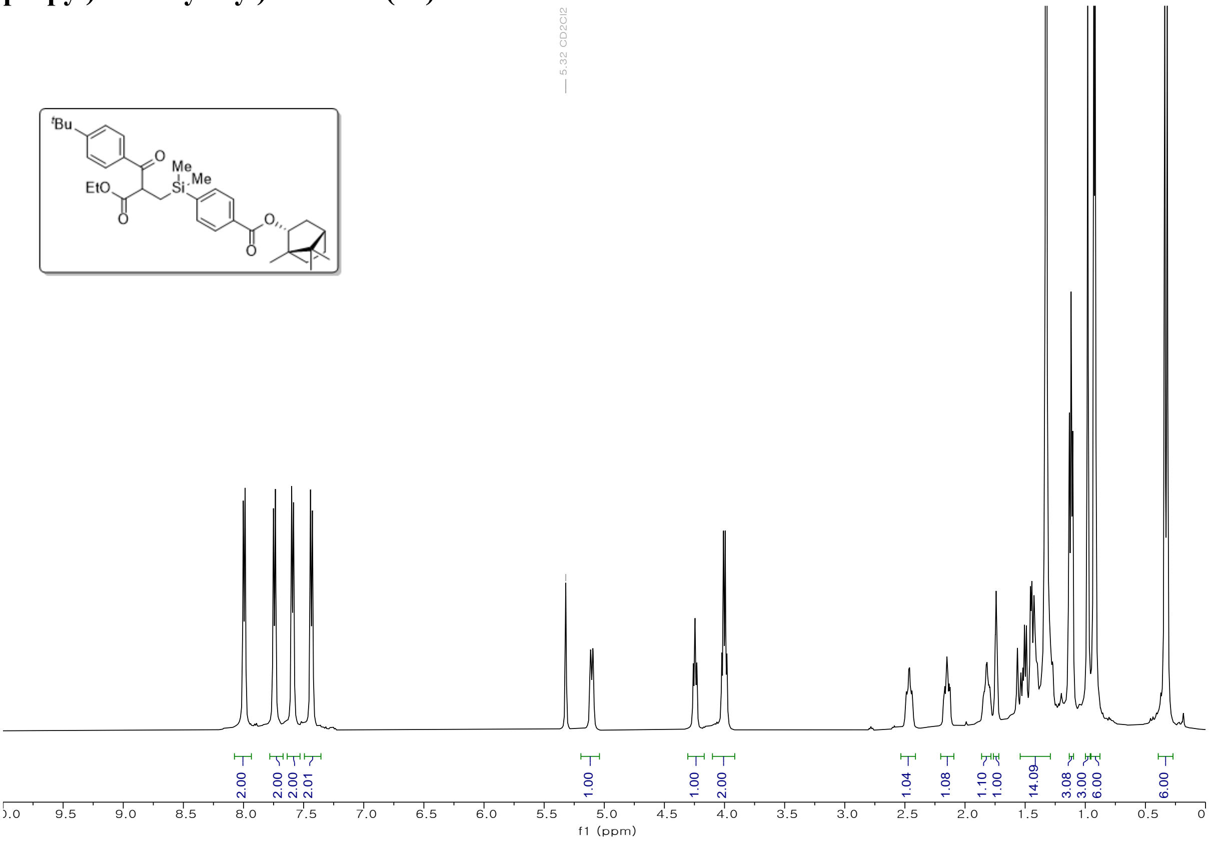

500 MHz, ^{1}H NMR in CD_2Cl_2

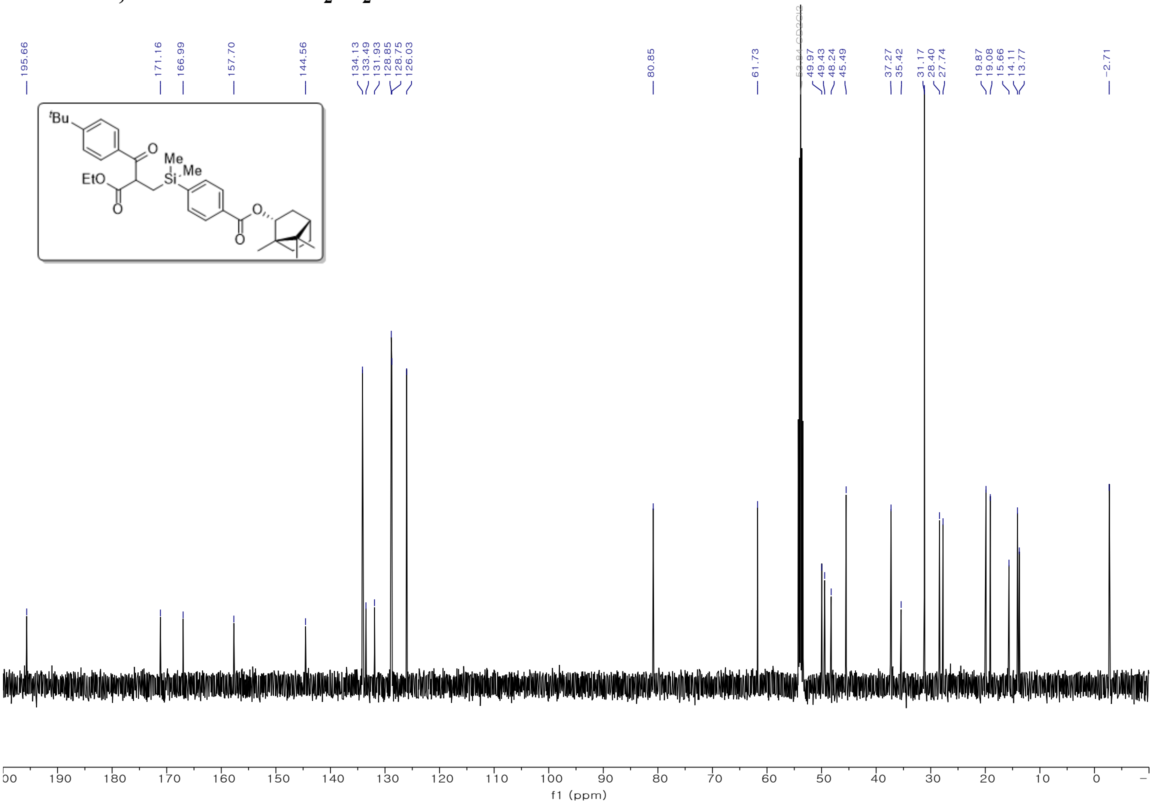

125 MHz, ^{13}C NMR in CD_2Cl_2

methyl 3-(4-(*tert*-butyl)phenyl)-2-((1,1,1,3,3,3-hexamethyl-2-(trimethylsilyl)trisilan-2-yl)methyl)-3-oxopropanoate (4aa).

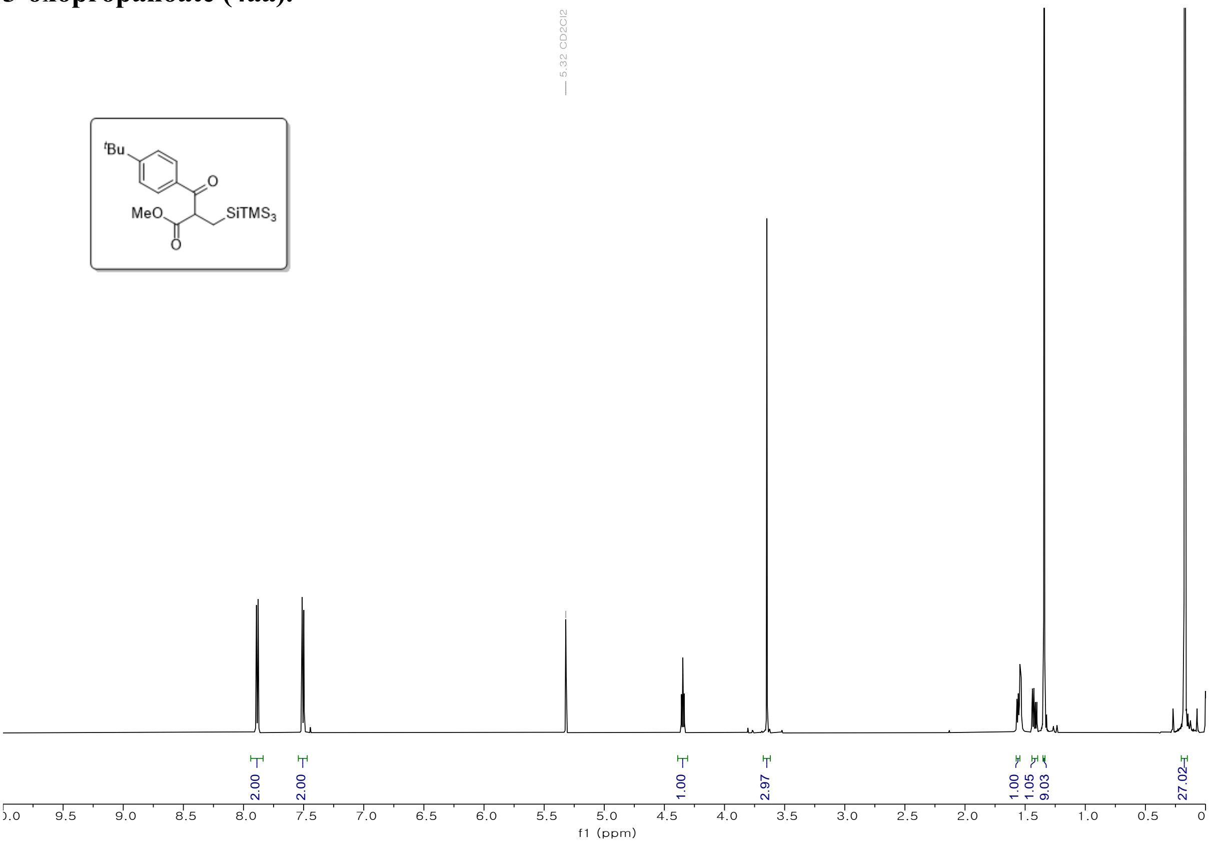

600 MHz, ^{1}H NMR in CD_2Cl_2

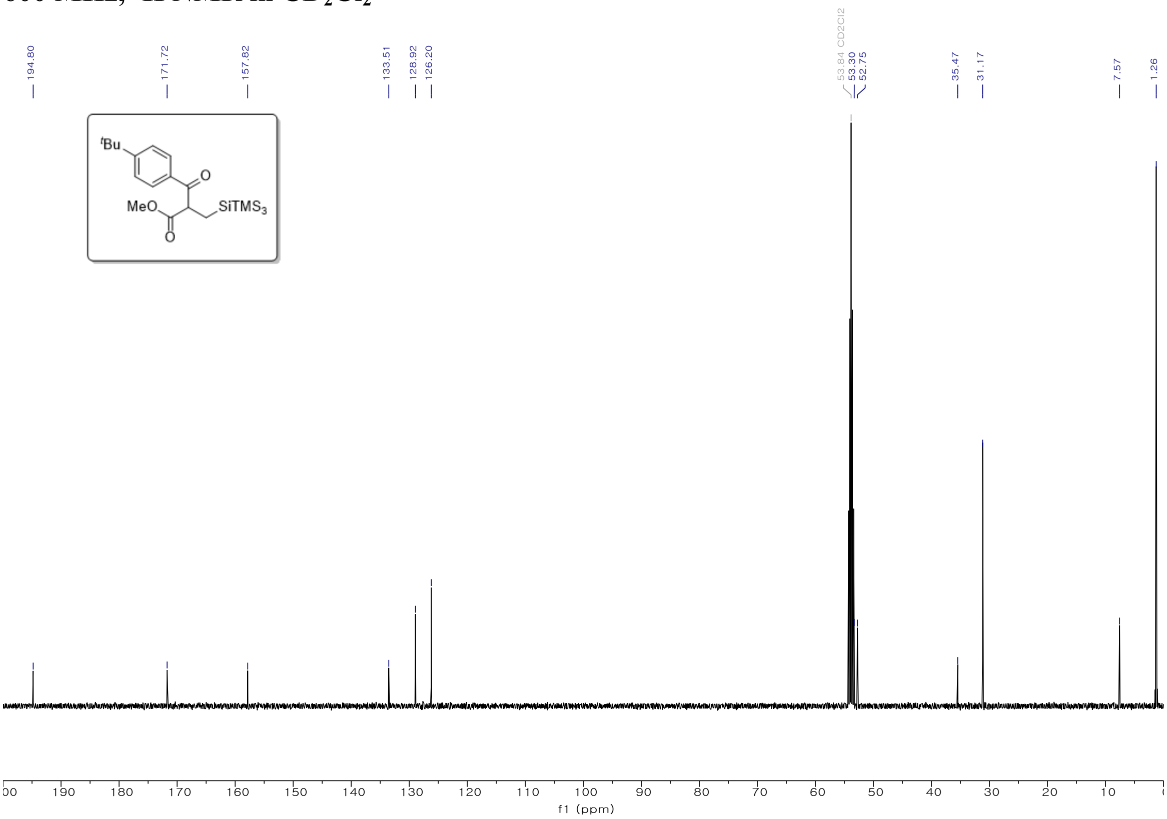

125 MHz, ^{13}C NMR in CD_2Cl_2

***tert*-butyl 3-(4-(*tert*-butyl)phenyl)-2-((1,1,1,3,3,3-hexamethyl-2-(trimethylsilyl)trisilan-2-yl)methyl)-3-oxopropanoate (4ab).**

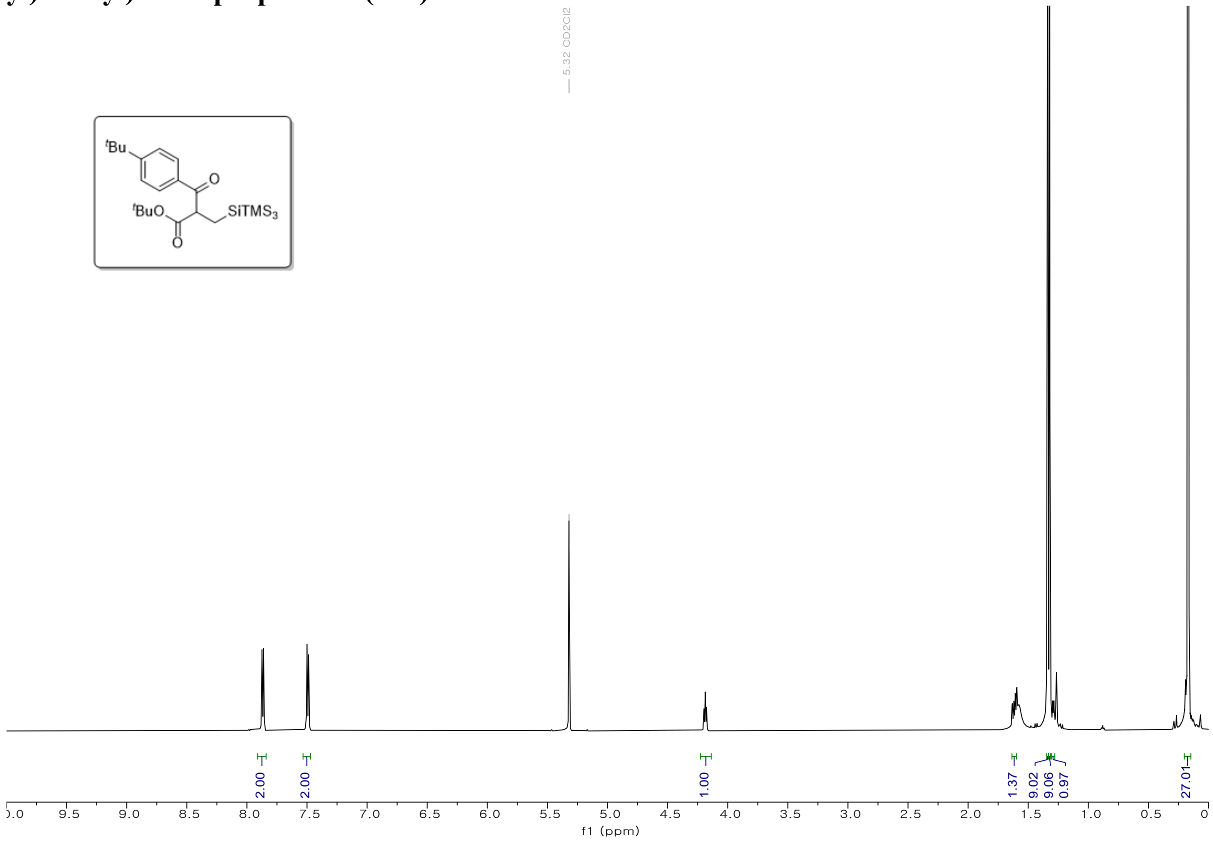

600 MHz, ^{1}H NMR in CD_2Cl_2

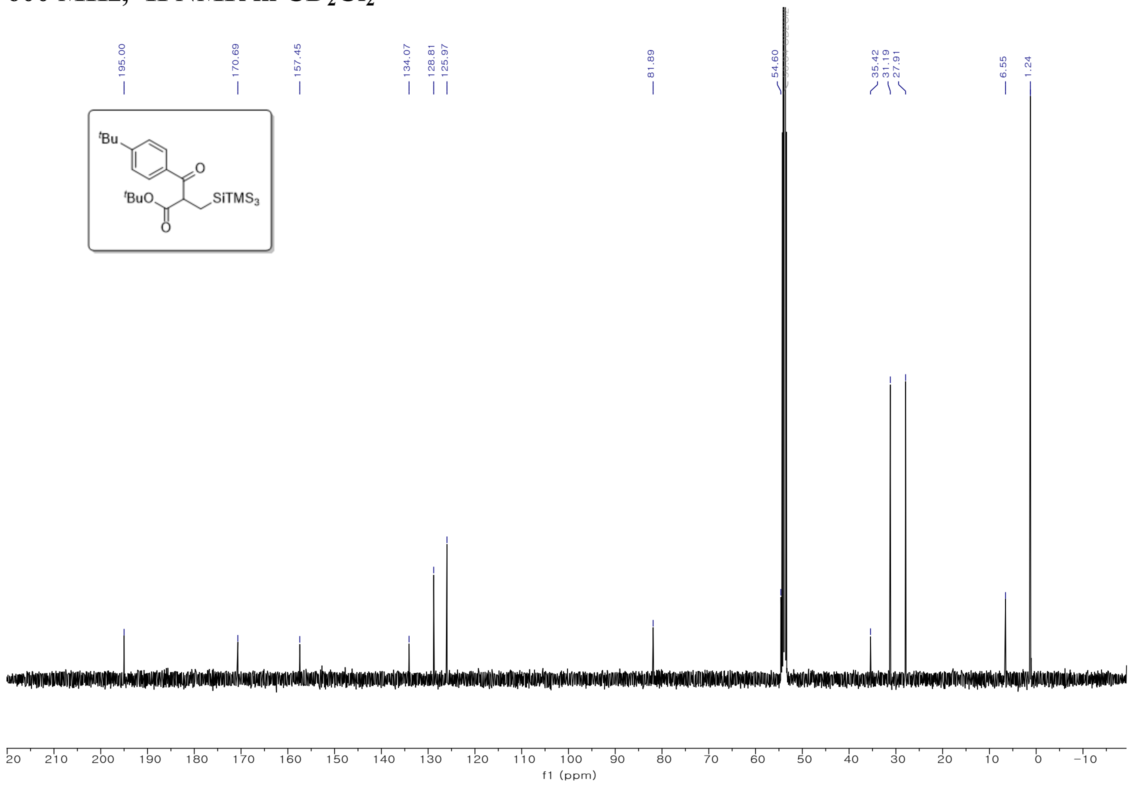

125 MHz, ^{13}C NMR in CD_2Cl_2

butyl 3-(4-(*tert*-butyl)phenyl)-2-((1,1,1,3,3,3-hexamethyl-2-(trimethylsilyl)trisilan-2-yl)methyl)-3-oxopropanoate (4ac).

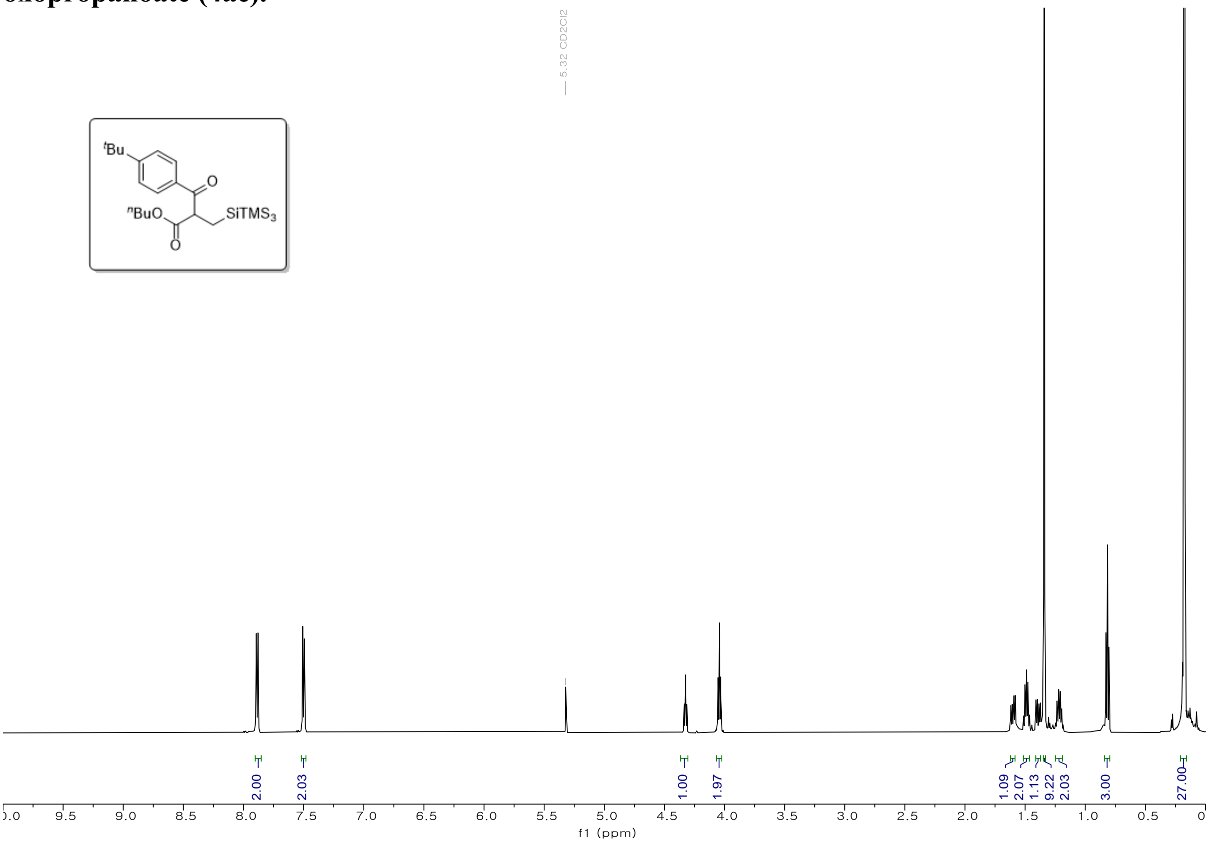

600 MHz, ^{1}H NMR in CD_2Cl_2

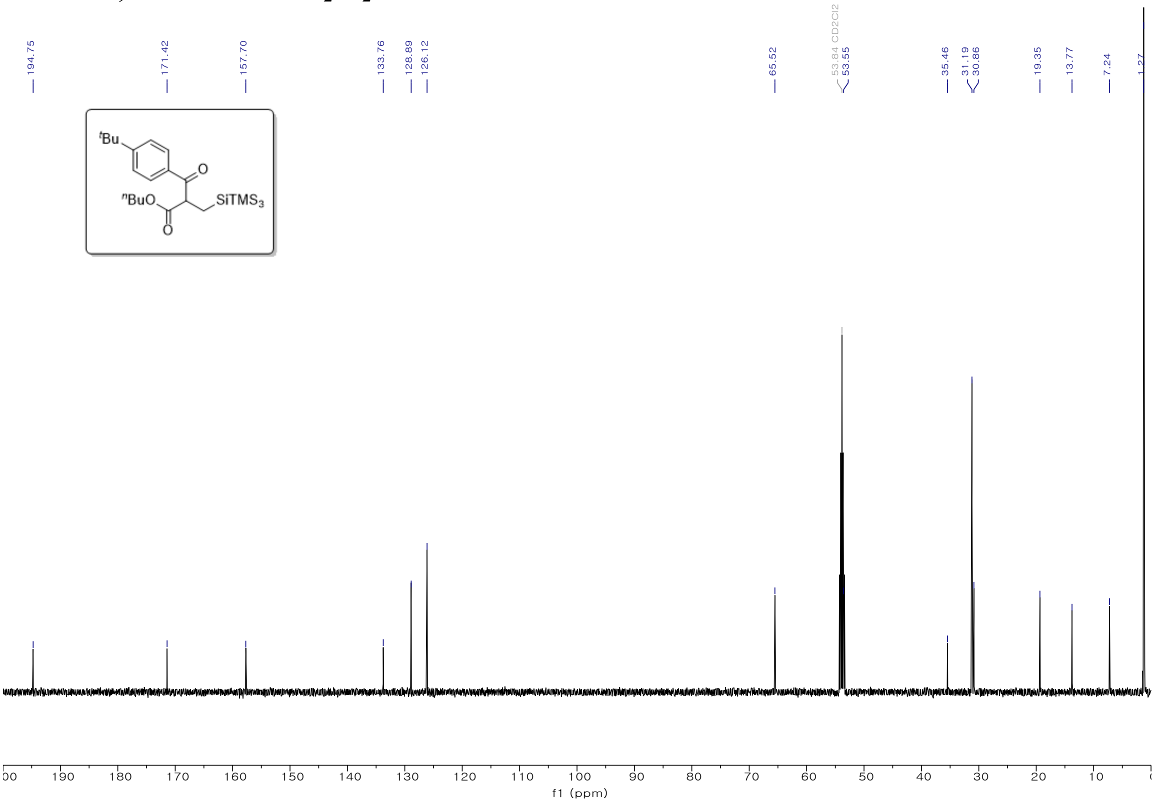

125 MHz, ^{13}C NMR in CD_2Cl_2

phenyl 3-(4-(*tert*-butyl)phenyl)-2-((1,1,1,3,3,3-hexamethyl-2-(trimethylsilyl)trisilan-2-yl)methyl)-3-oxopropanoate (4ad).

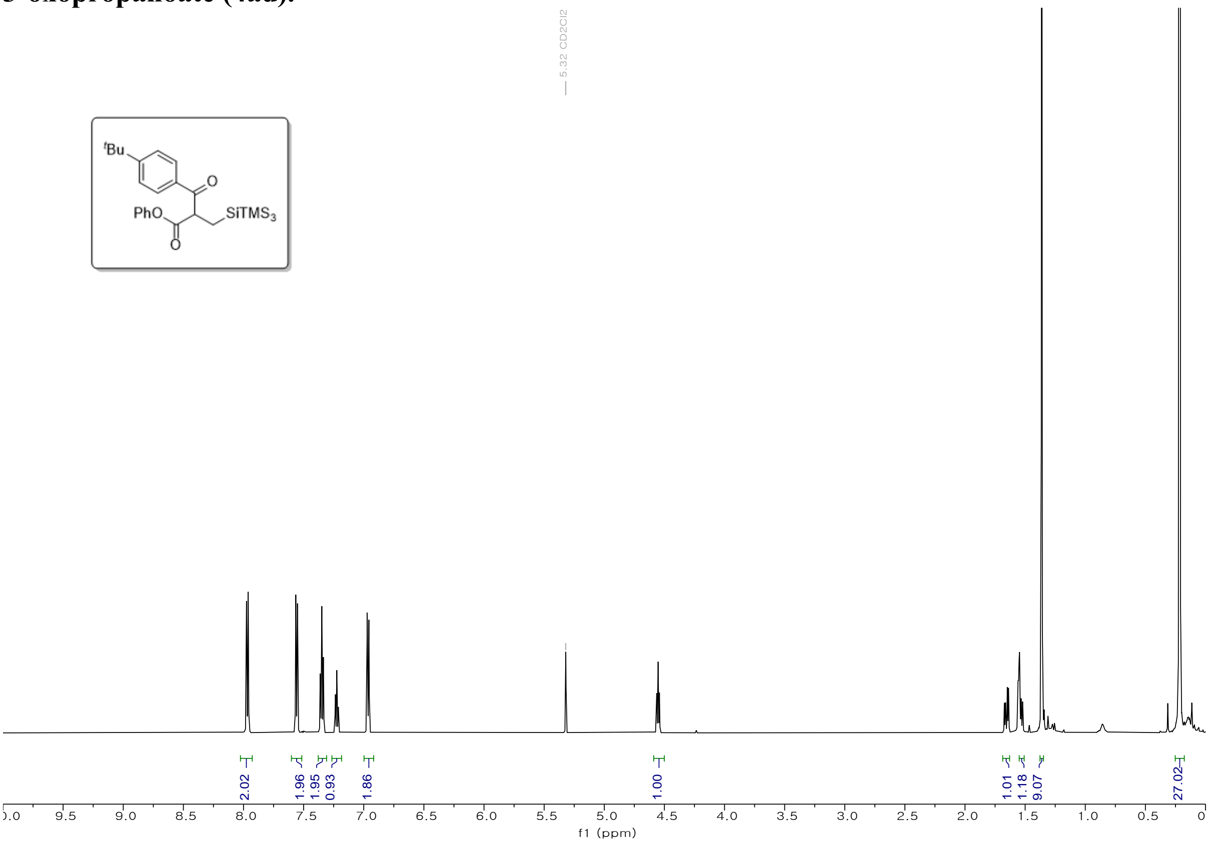

600 MHz, ^{1}H NMR in CD_2Cl_2

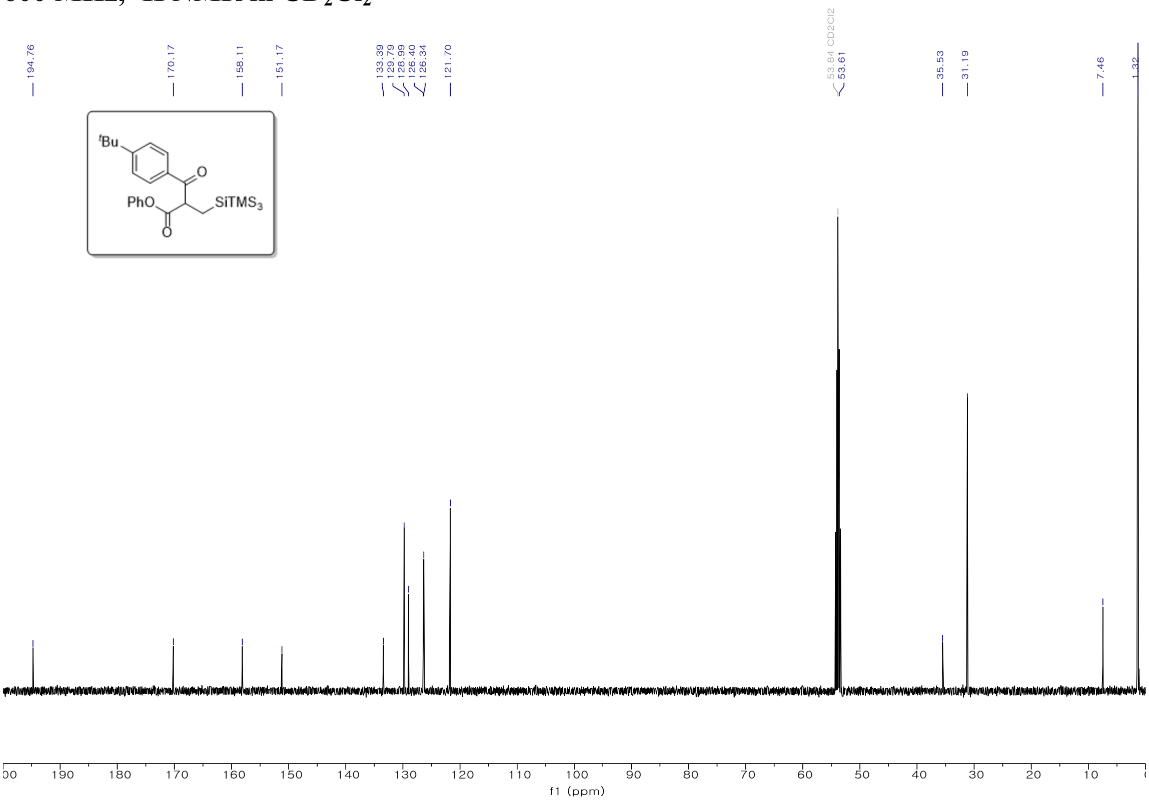

125 MHz, ^{13}C NMR in CD_2Cl_2

benzyl 3-(4-(*tert*-butyl)phenyl)-2-((1,1,1,3,3,3-hexamethyl-2-(trimethylsilyl)trisilan-2-yl)methyl)-3-oxopropanoate (4ae).

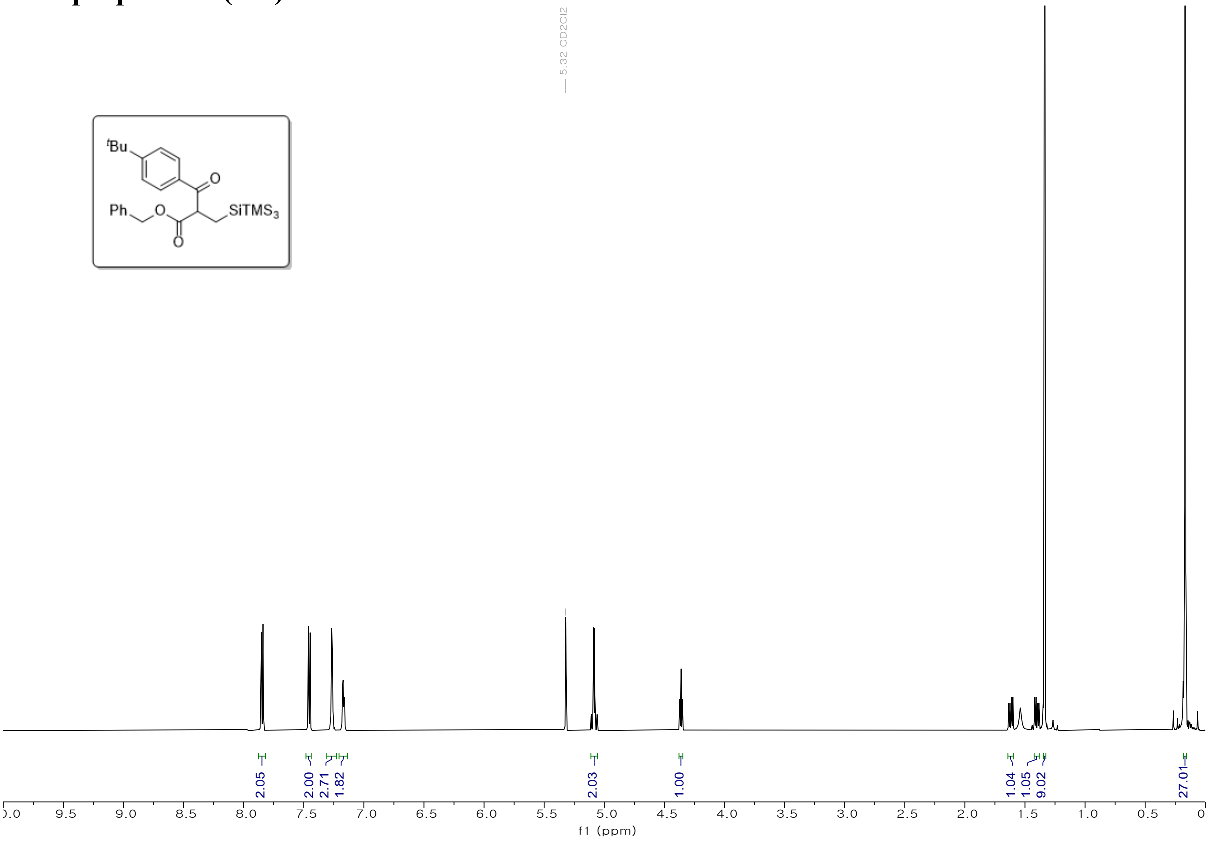

600 MHz, ^{1}H NMR in CD_2Cl_2

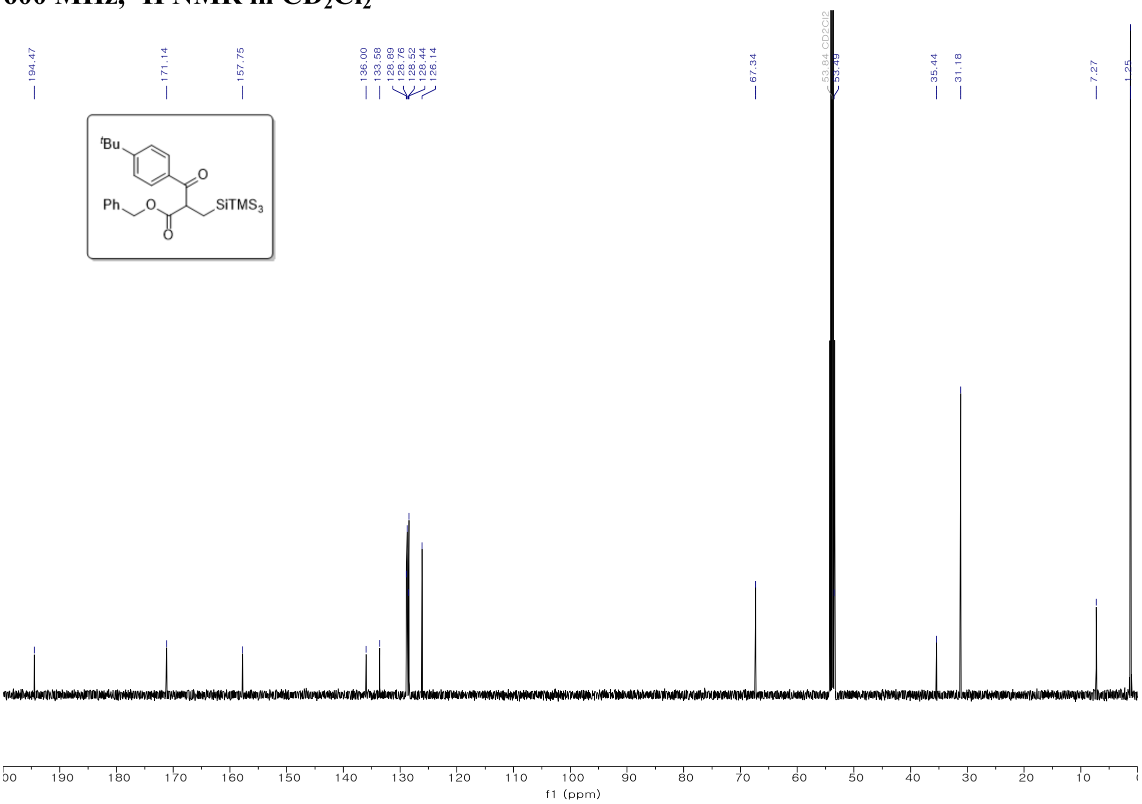

125 MHz, ^{13}C NMR in CD_2Cl_2

2-methoxyethyl 3-(4-(*tert*-butyl)phenyl)-2-((1,1,1,3,3,3-hexamethyl-2-(trimethylsilyl)trisilan-2-yl)methyl)-3-oxopropanoate (4af).

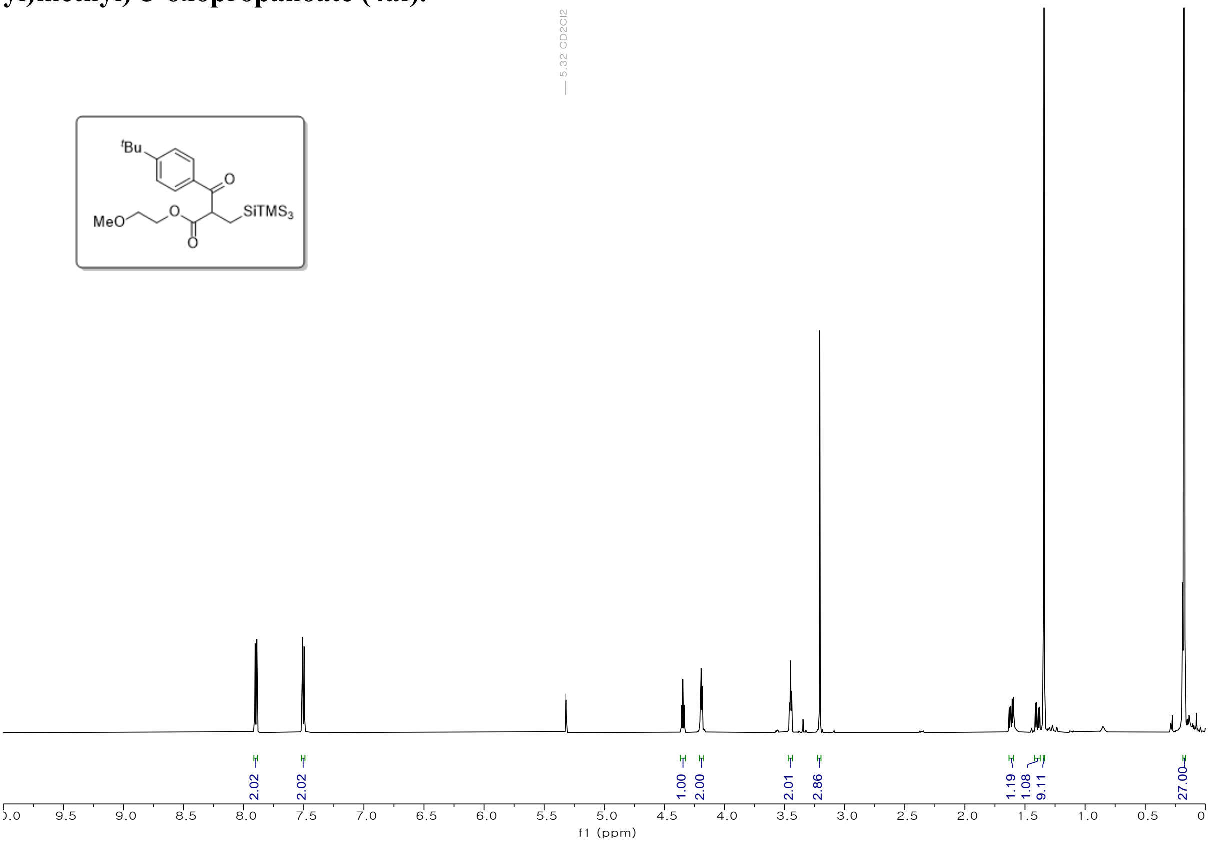

600 MHz, ^{1}H NMR in CD_2Cl_2

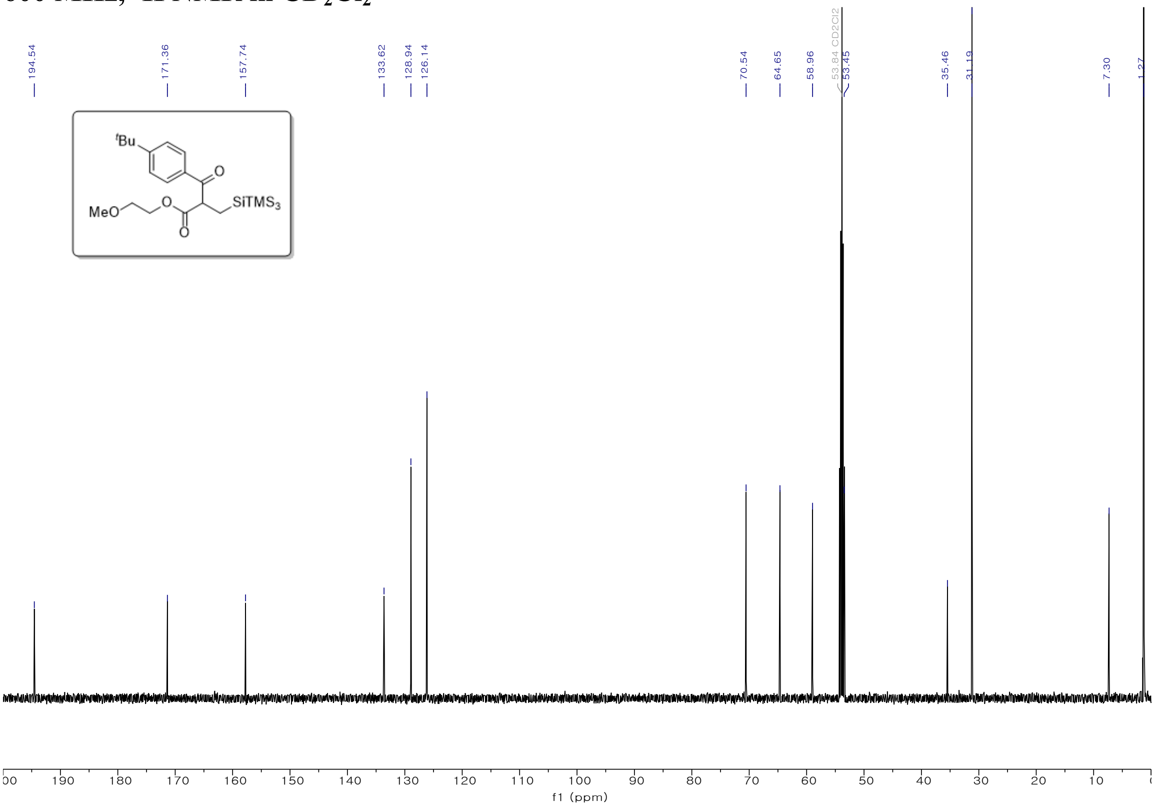

125 MHz, ^{13}C NMR in CD_2Cl_2

***tert*-butyl 4-((3-(4-(*tert*-butyl)phenyl)-2-((1,1,1,3,3,3-hexamethyl-2-(trimethylsilyl)trisilan-2-yl)methyl)-3-oxopropanoyl)oxy)piperidine-1-carboxylate (4ag).**

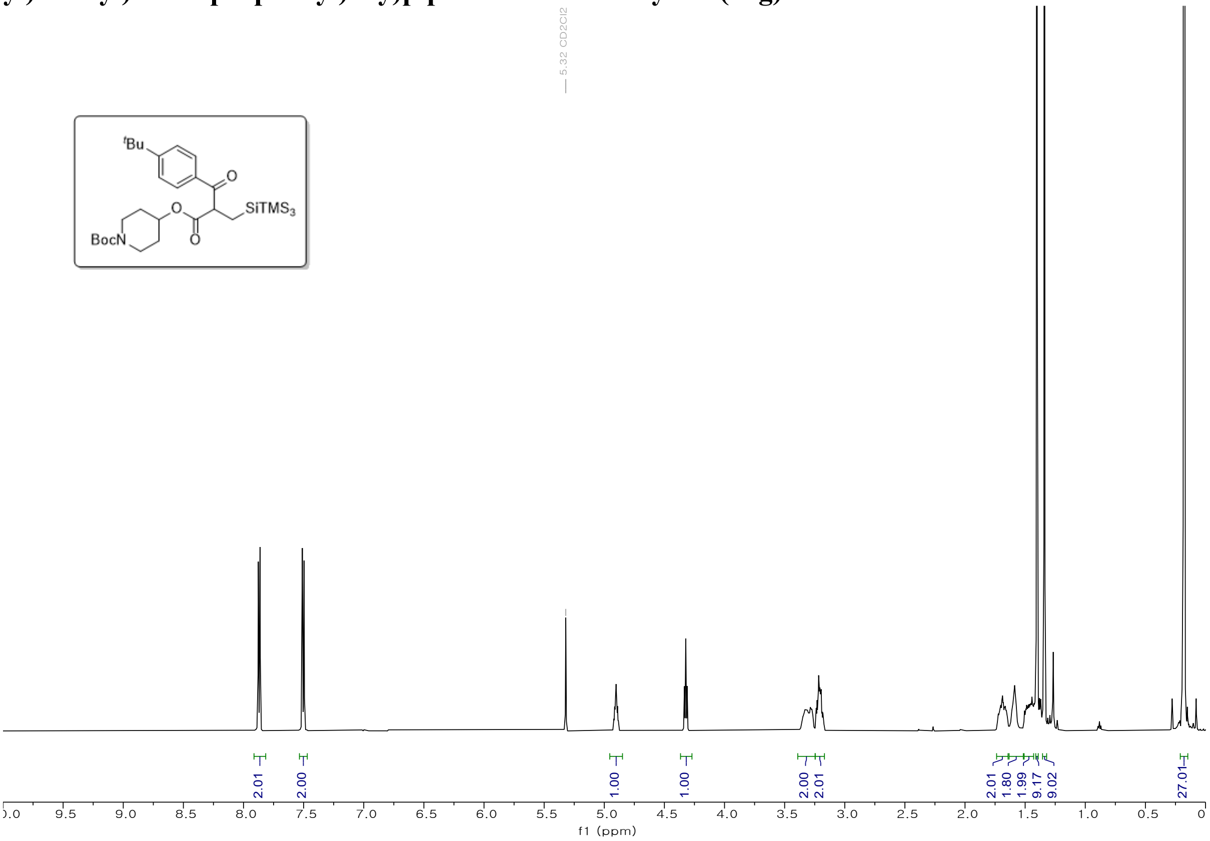

600 MHz, ^{1}H NMR in CD_2Cl_2

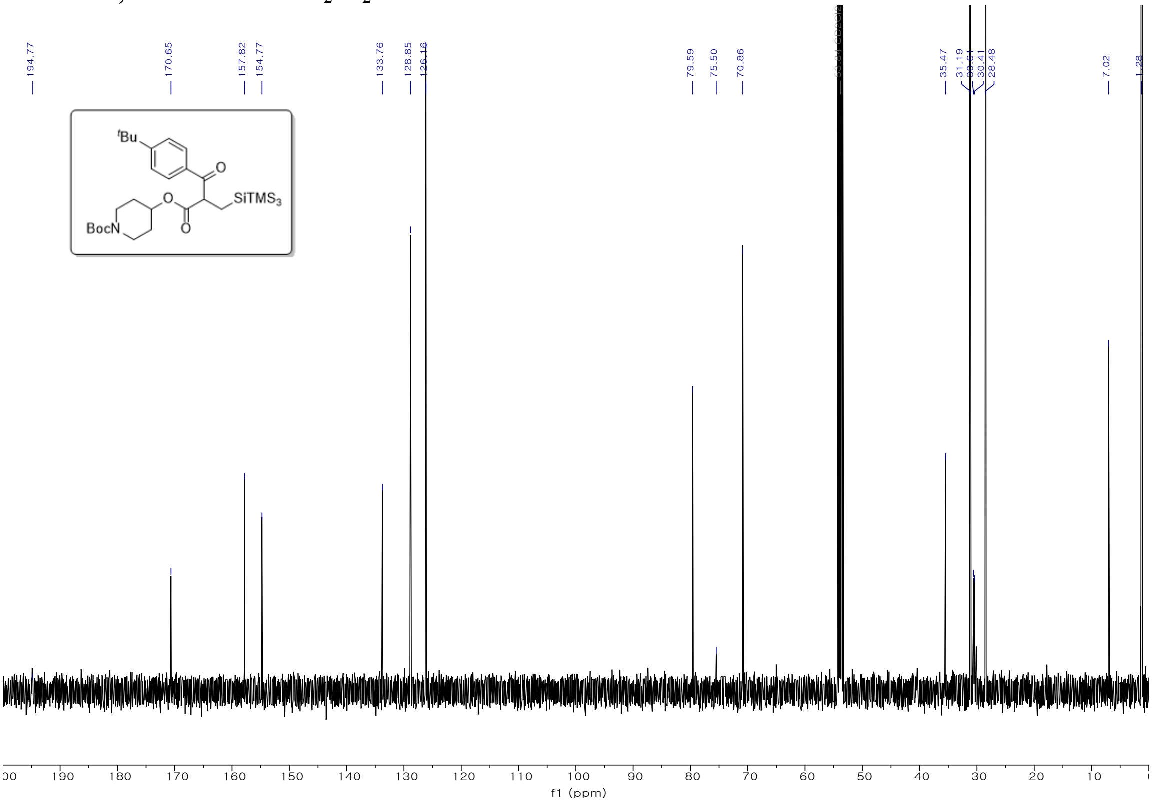

125 MHz, ^{13}C NMR in CD_2Cl_2

ethyl 6-((3-(4-(*tert*-butyl)phenyl)-2-((1,1,1,3,3,3-hexamethyl-2-(trimethylsilyl)trisilan-2-yl)methyl)-3-oxopropanoyl)oxy)hexanoate (4ah).

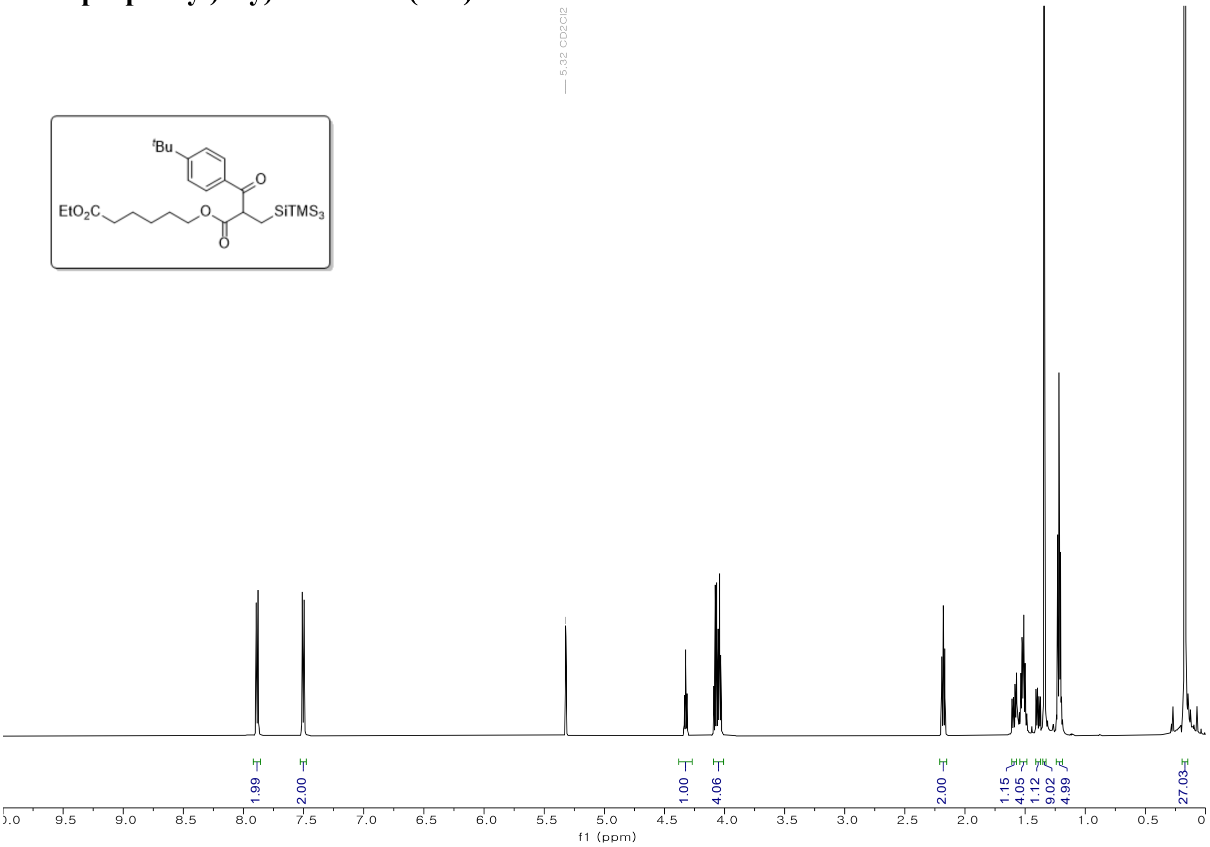

600 MHz, ^{1}H NMR in CD_2Cl_2

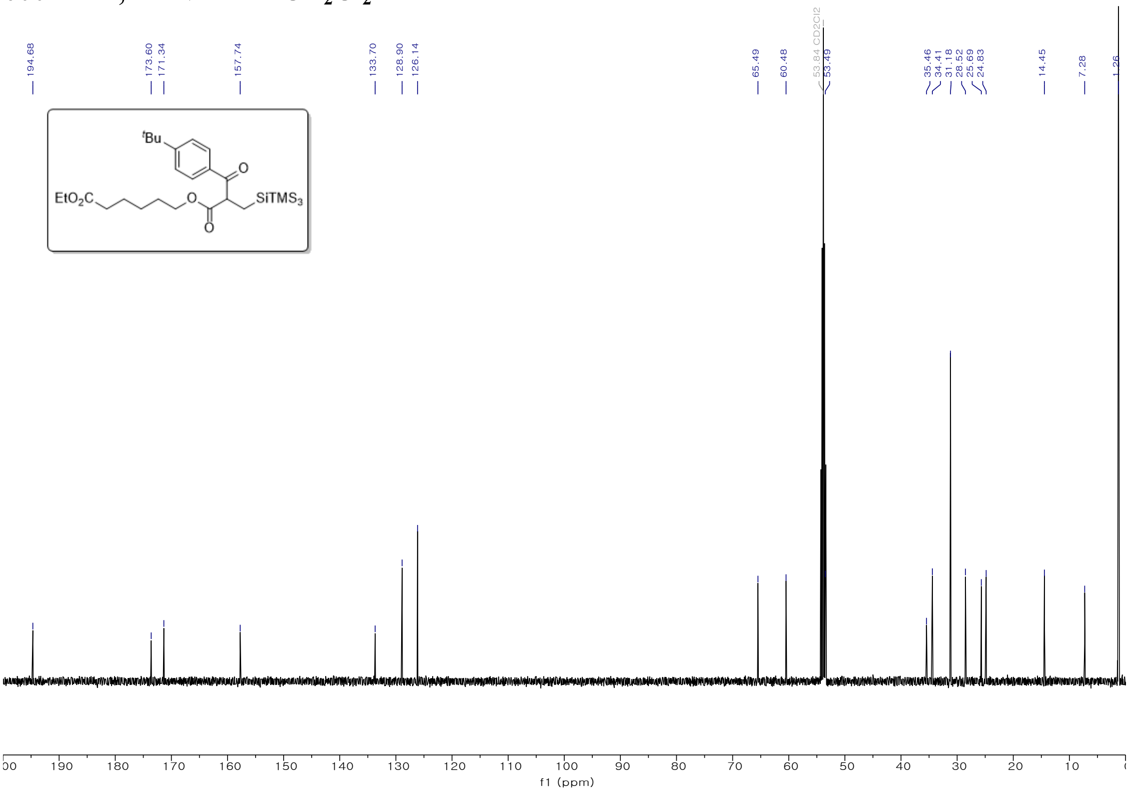

125 MHz, ^{13}C NMR in CD_2Cl_2

2-hydroxyethyl 3-(4-(*tert*-butyl)phenyl)-2-((1,1,1,3,3,3-hexamethyl-2-(trimethylsilyl)trisilan-2-yl)methyl)-3-oxopropanoate (4ai).

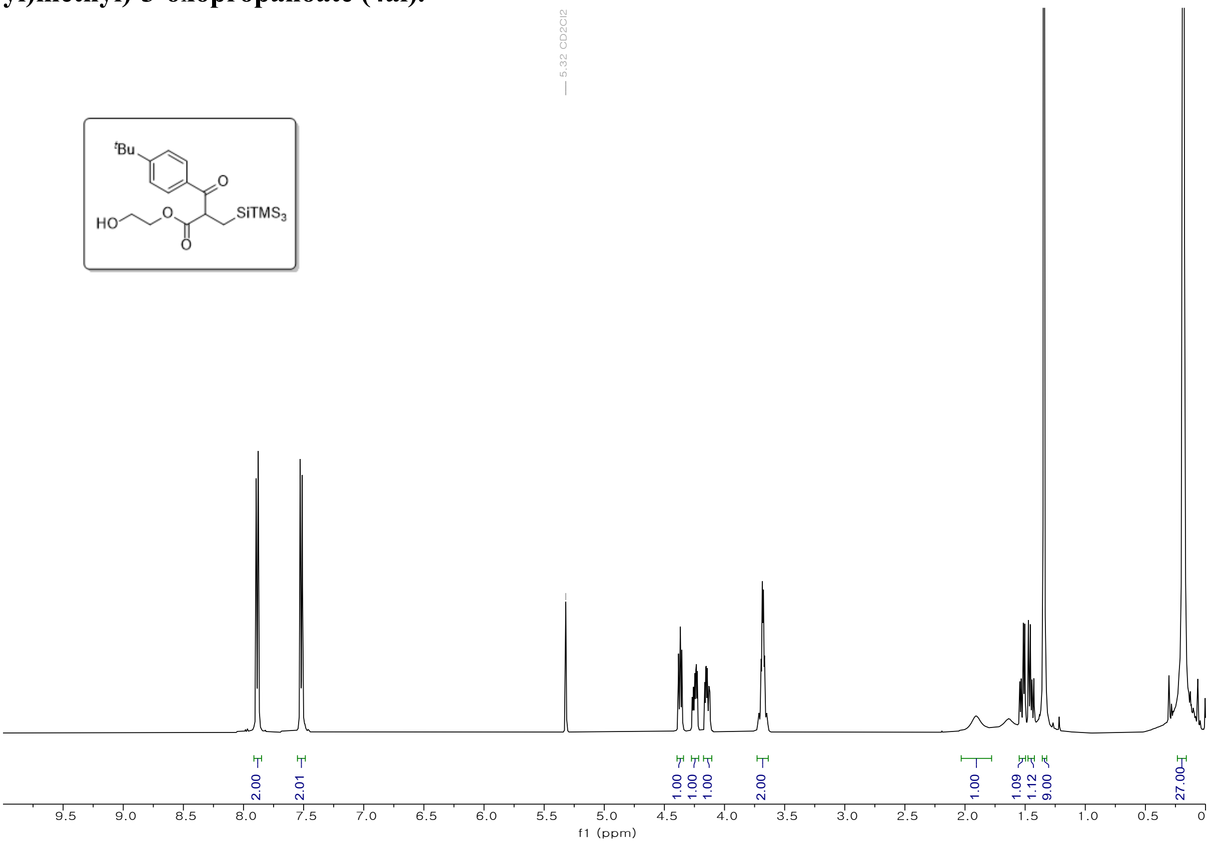

500 MHz, ^{1}H NMR in CD_2Cl_2

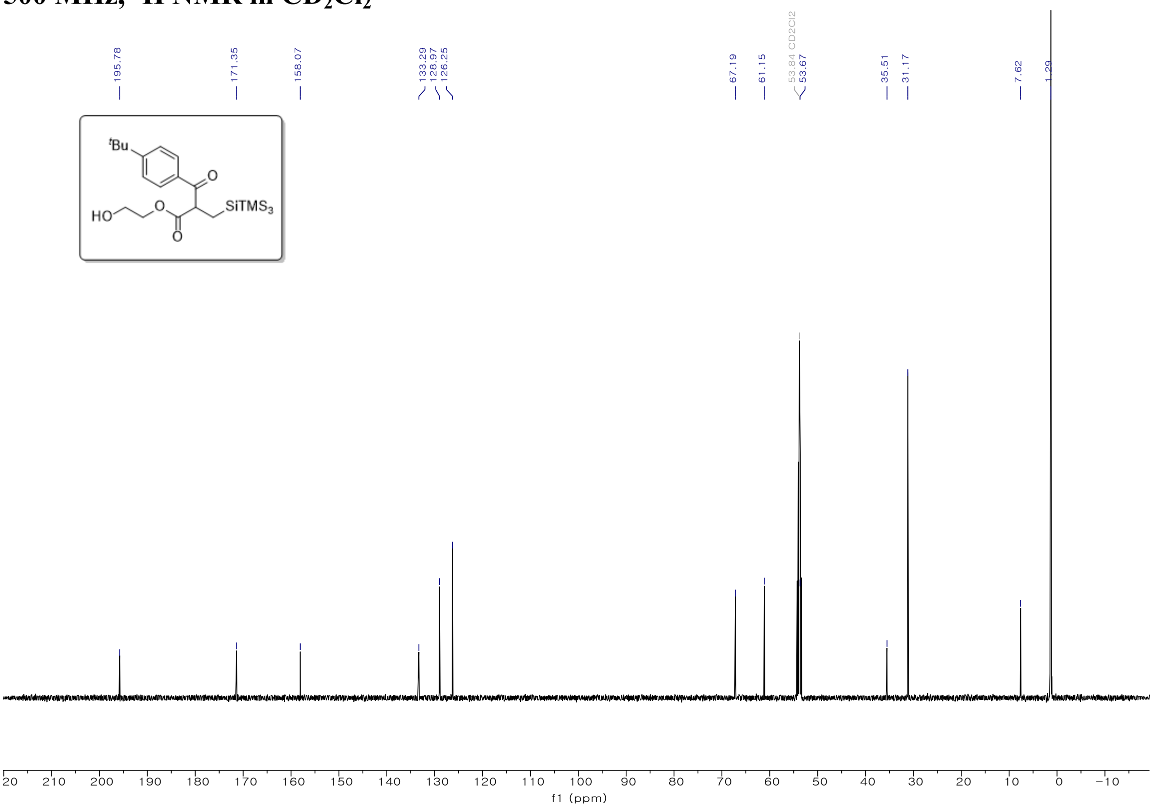

125 MHz, ^{13}C NMR in CD_2Cl_2

tetrahydro-2*H*-pyran-2-yl)methyl 3-(4-(*tert*-butyl)phenyl)-2-((1,1,1,3,3,3-hexamethyl-2-(trimethylsilyl)trisilan-2-yl)methyl)-3-oxopropanoate (4aj).

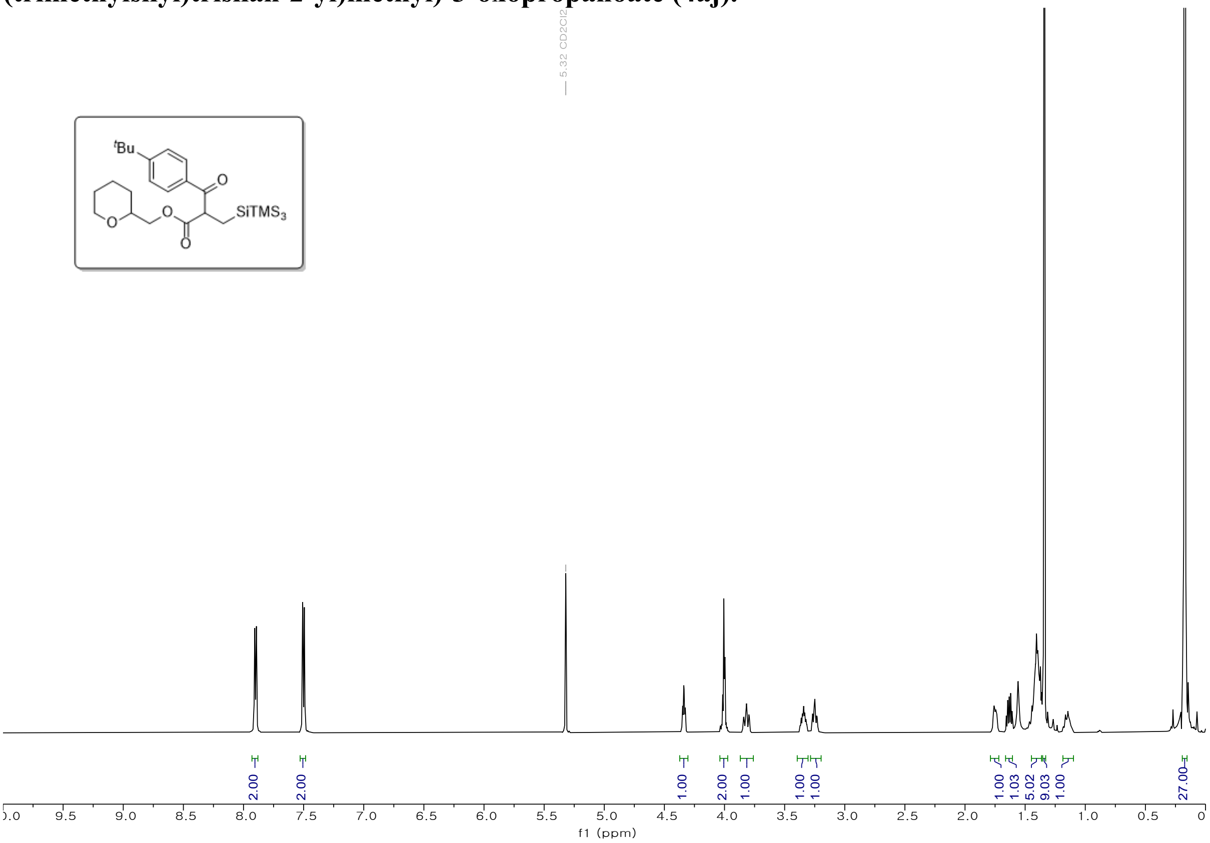

600 MHz, ^{1}H NMR in CD_2Cl_2

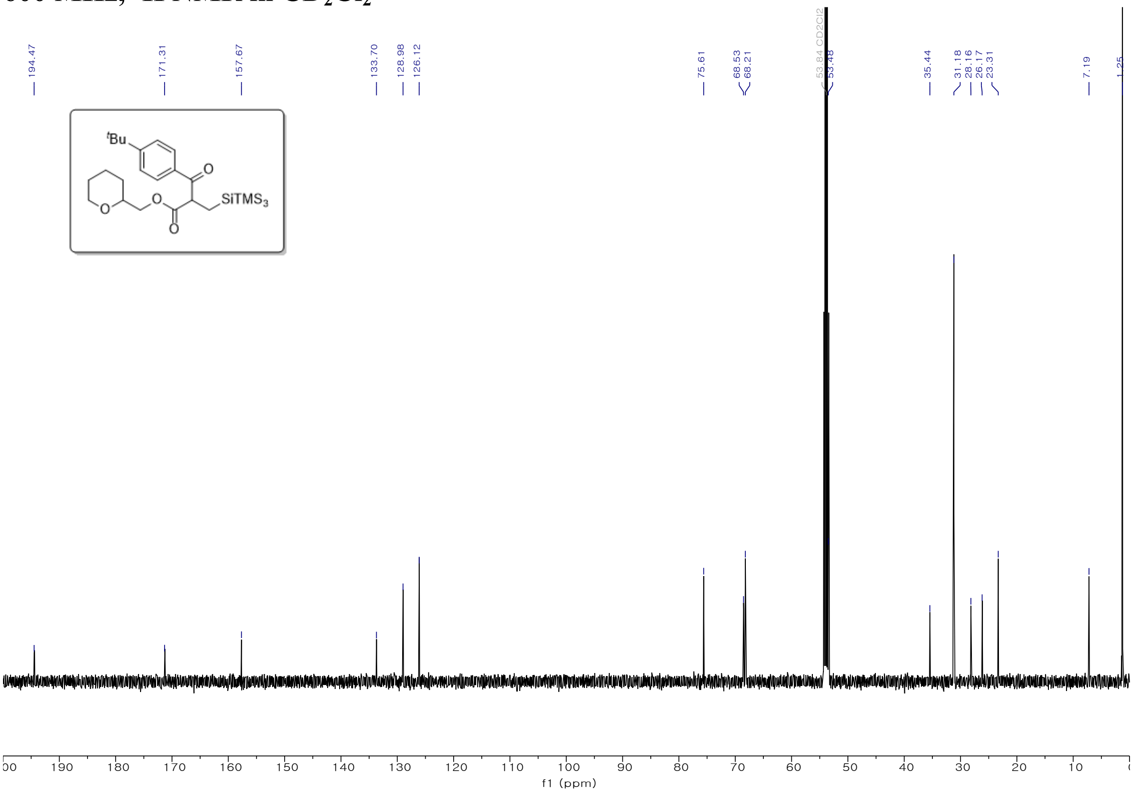

125 MHz, ^{13}C NMR in CD_2Cl_2

***N*-(*tert*-butyl)-3-(4-(*tert*-butyl)phenyl)-2-((1,1,1,3,3,3-hexamethyl-2-(trimethylsilyl)trisilan-2-yl)methyl)-3-oxopropanamide (4ak).**

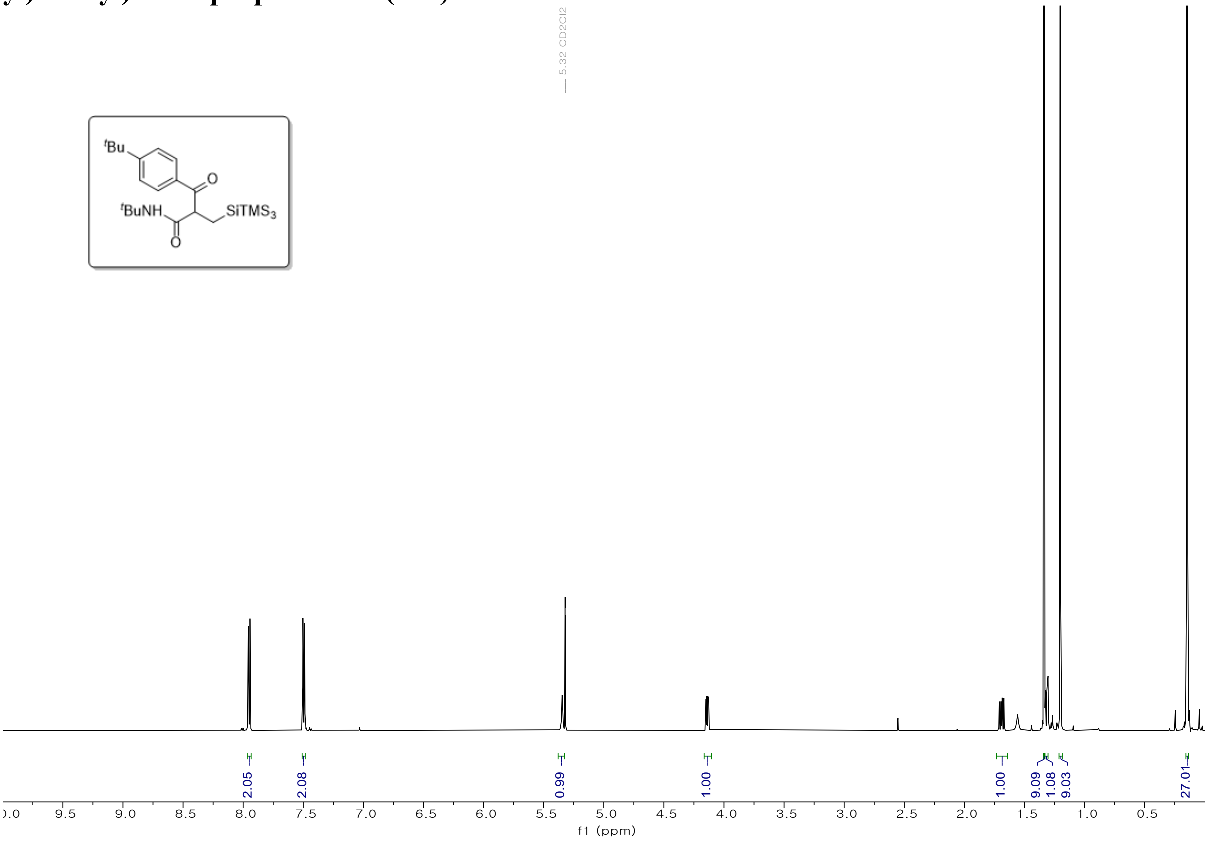

600 MHz, ^{1}H NMR in CD_2Cl_2

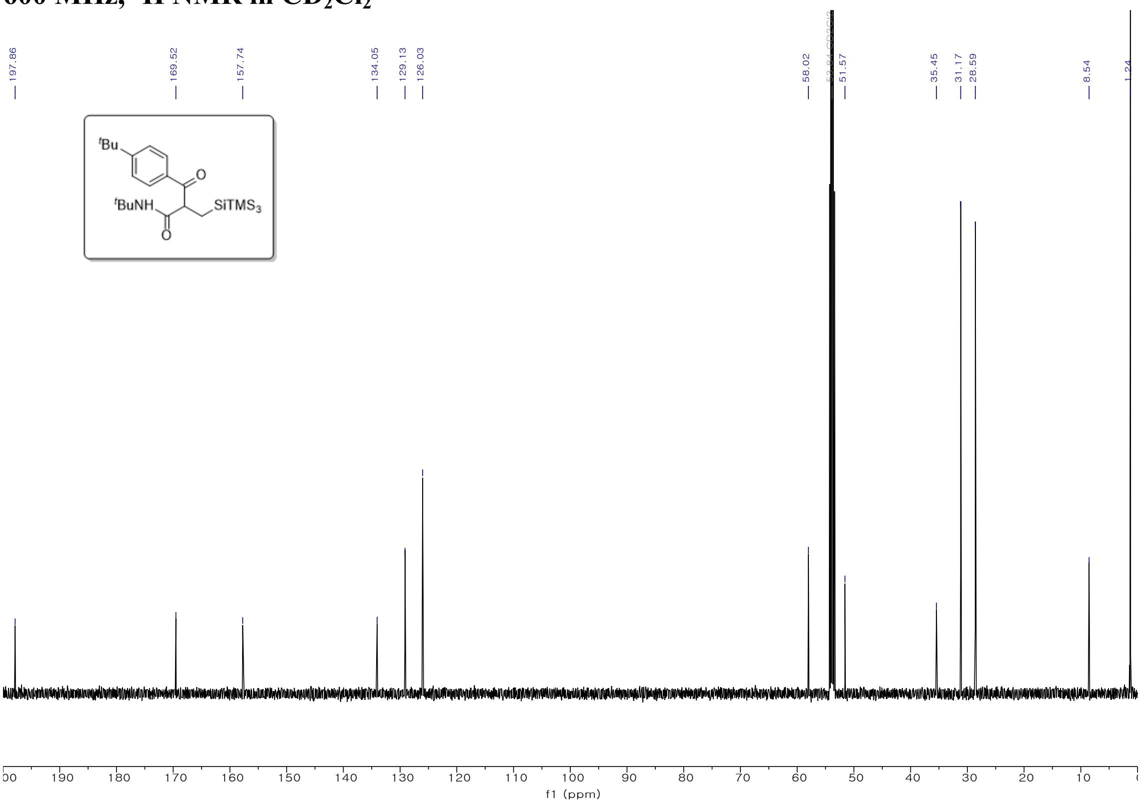

125 MHz, ^{13}C NMR in CD_2Cl_2

2-isopropyl-5-methylcyclohexyl 3-(4-(*tert*-butyl)phenyl)-2-((1,1,1,3,3,3-hexamethyl-2-(trimethylsilyl)trisilan-2-yl)methyl)-3-oxopropanoate (4al).

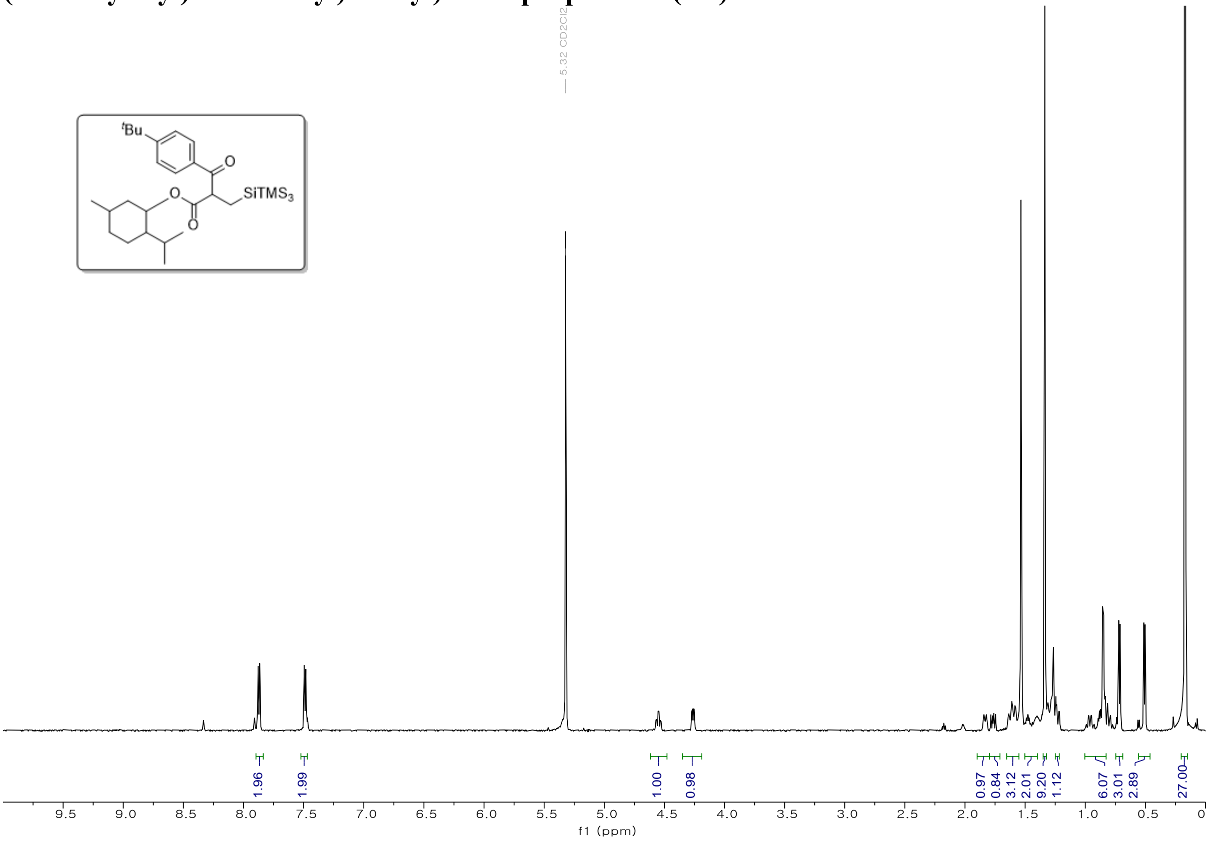

600 MHz, ^{1}H NMR in CD_2Cl_2

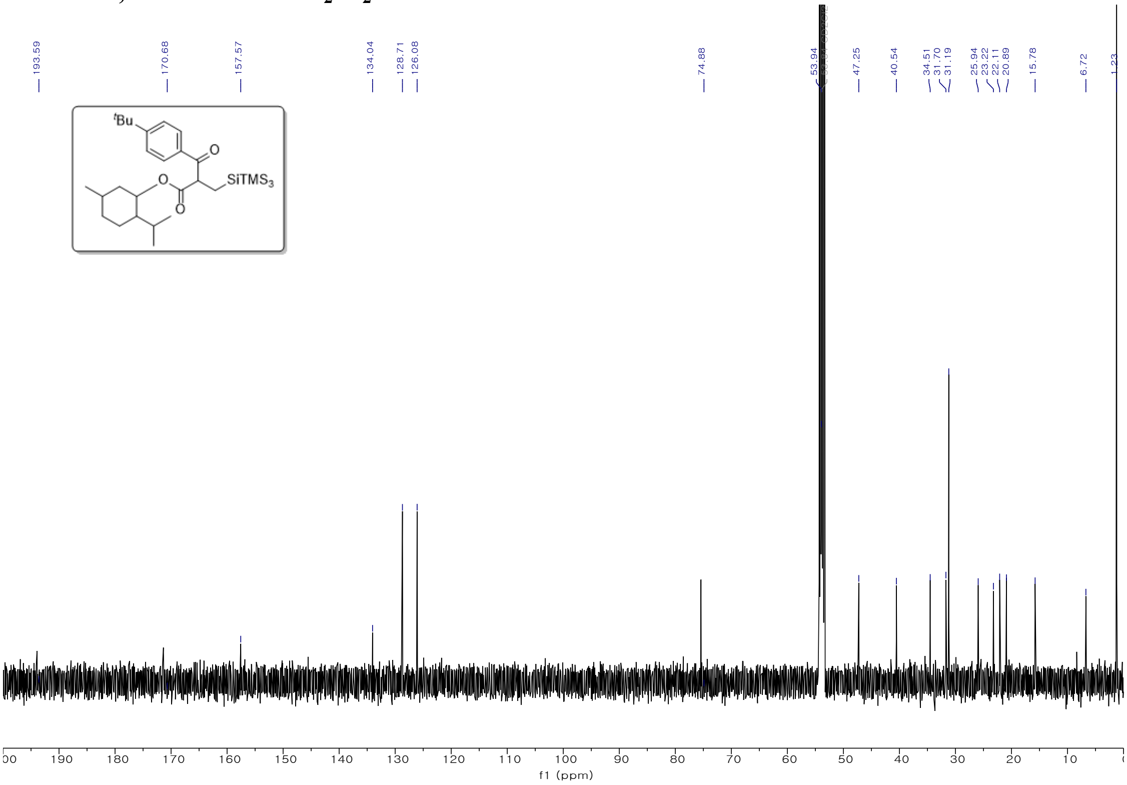

125 MHz, ^{13}C NMR in CD_2Cl_2

10,13-dimethyl-17-(6-methylheptan-2-yl)-2,3,4,7,8,9,10,11,12,13,14,15,16,17-tetradecahydro-1*H*-cyclopenta[a]phenanthren-3-yl 3-(4-(*tert*-butyl)phenyl)-2-((1,1,1,3,3,3-hexamethyl-2-(trimethyl silyl)trisilan-2-yl)methyl)-3-oxopropanoate (4am).

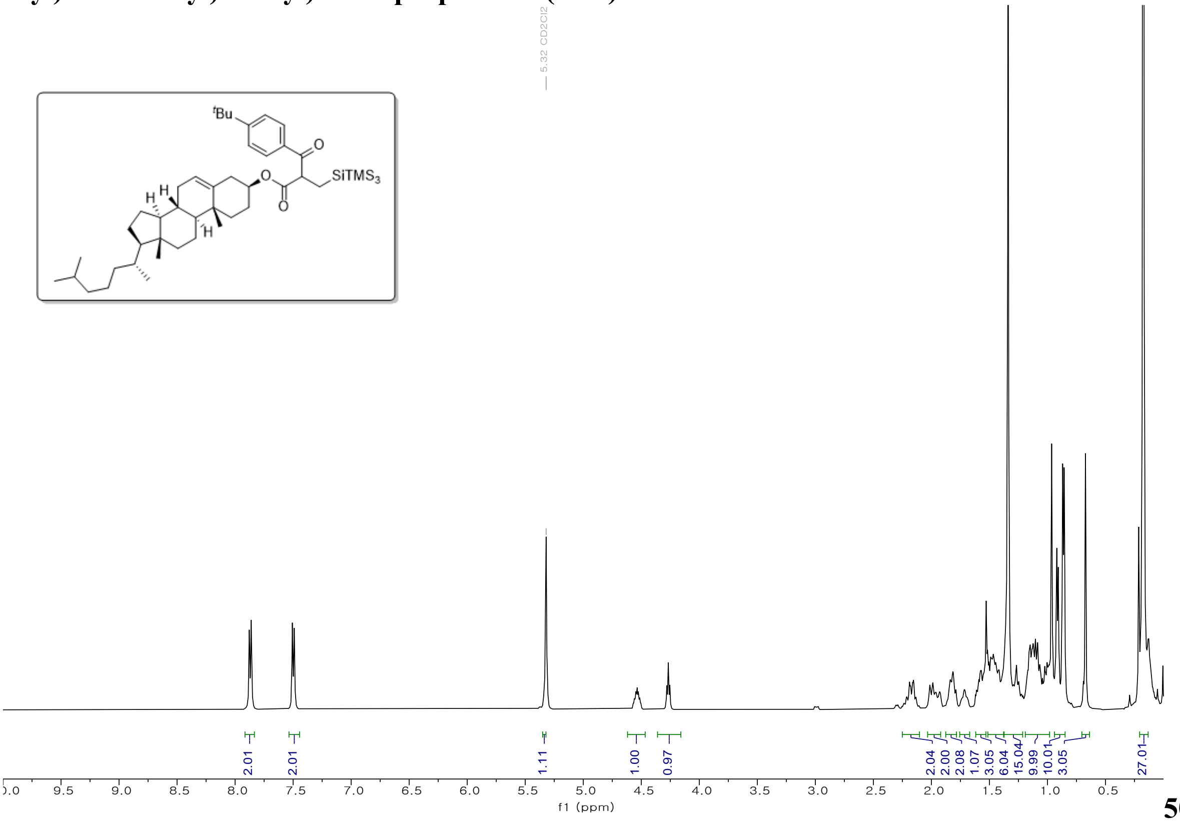

50

0 MHz, ^{1}H NMR in CD_2Cl_2

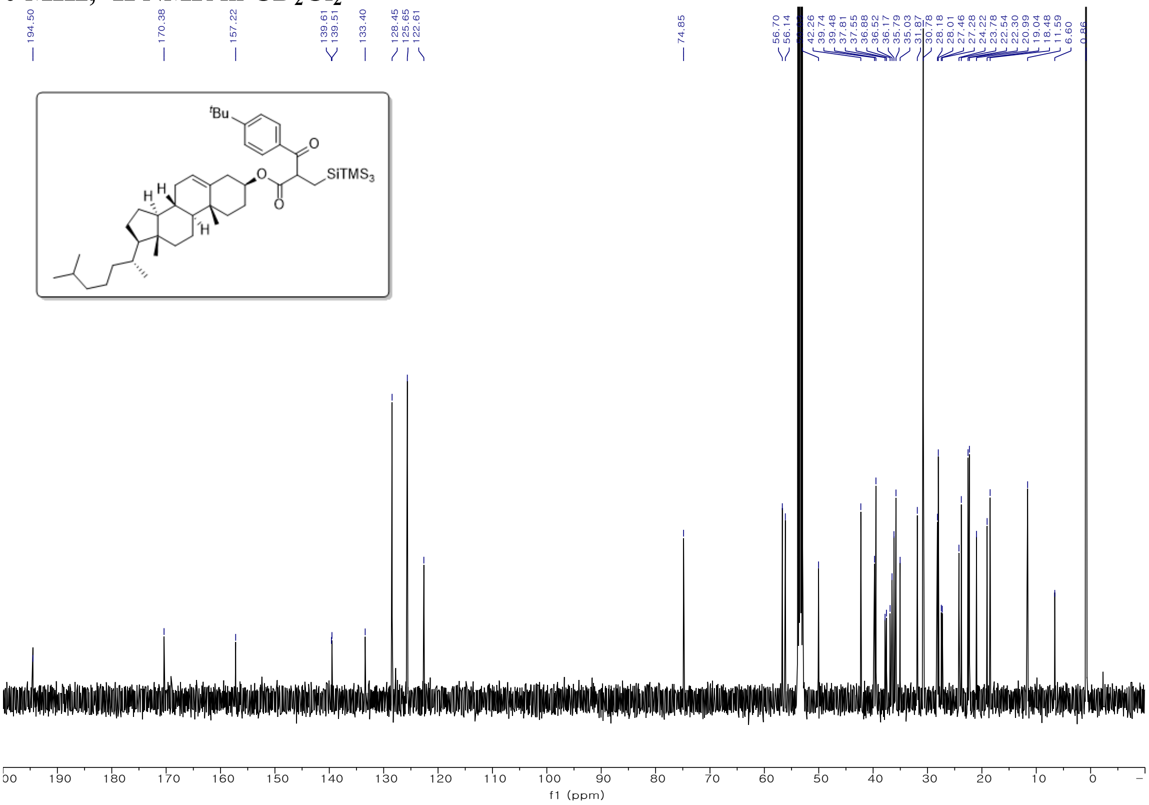

125 MHz, ^{13}C NMR in CD_2Cl_2

13-methyl-17-oxo-7,8,9,11,12,13,14,15,16,17-decahydro-6*H*-cyclopenta[*a*]phenanthren-3-yl 3-(4-(*tert*-butyl)phenyl)-2-((1,1,1,3,3,3-hexamethyl-2-(trimethylsilyl)trisilan-2-yl)methyl)-3-oxopropanoate (4an).

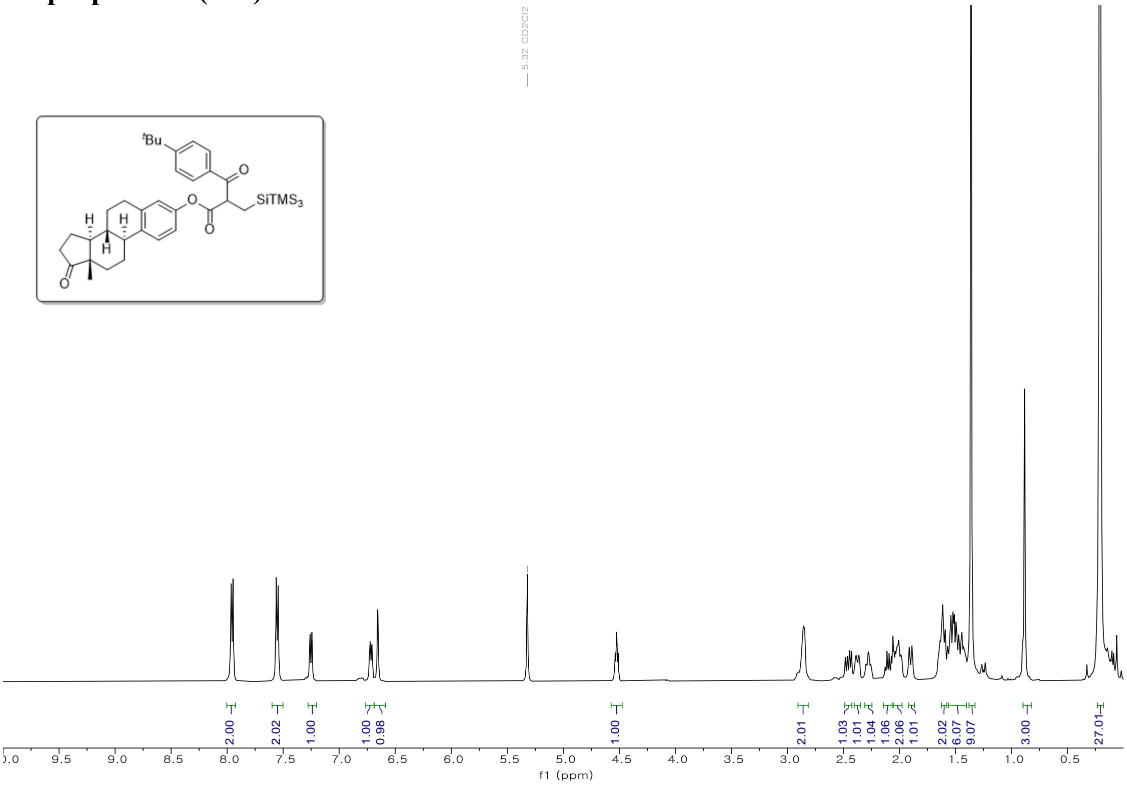

500 MHz, ^{1}H NMR in CD_2Cl_2

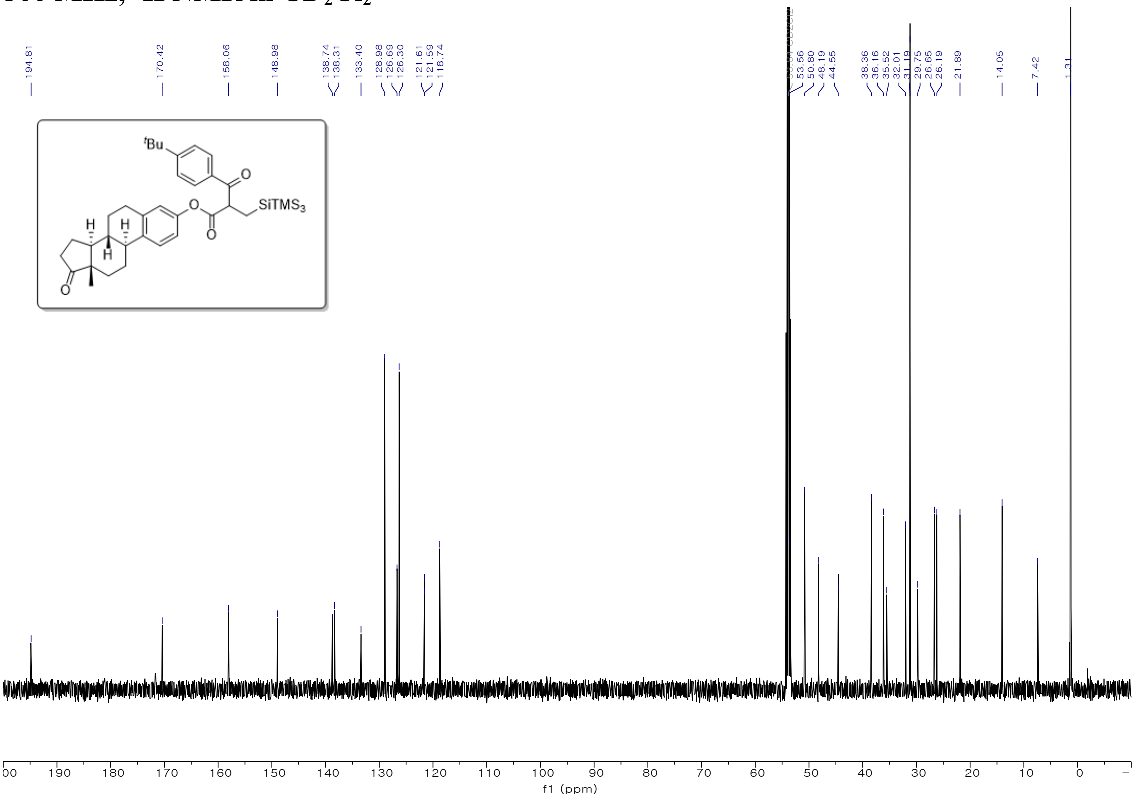

125 MHz, ^{13}C NMR in CD_2Cl_2

4-allyl-2-methoxyphenyl 3-(4-(*tert*-butyl)phenyl)-2-((1,1,1,3,3,3-hexamethyl-2-(trimethylsilyl)trisilan-2-yl)methyl)-3-oxopropanoate (4ao).

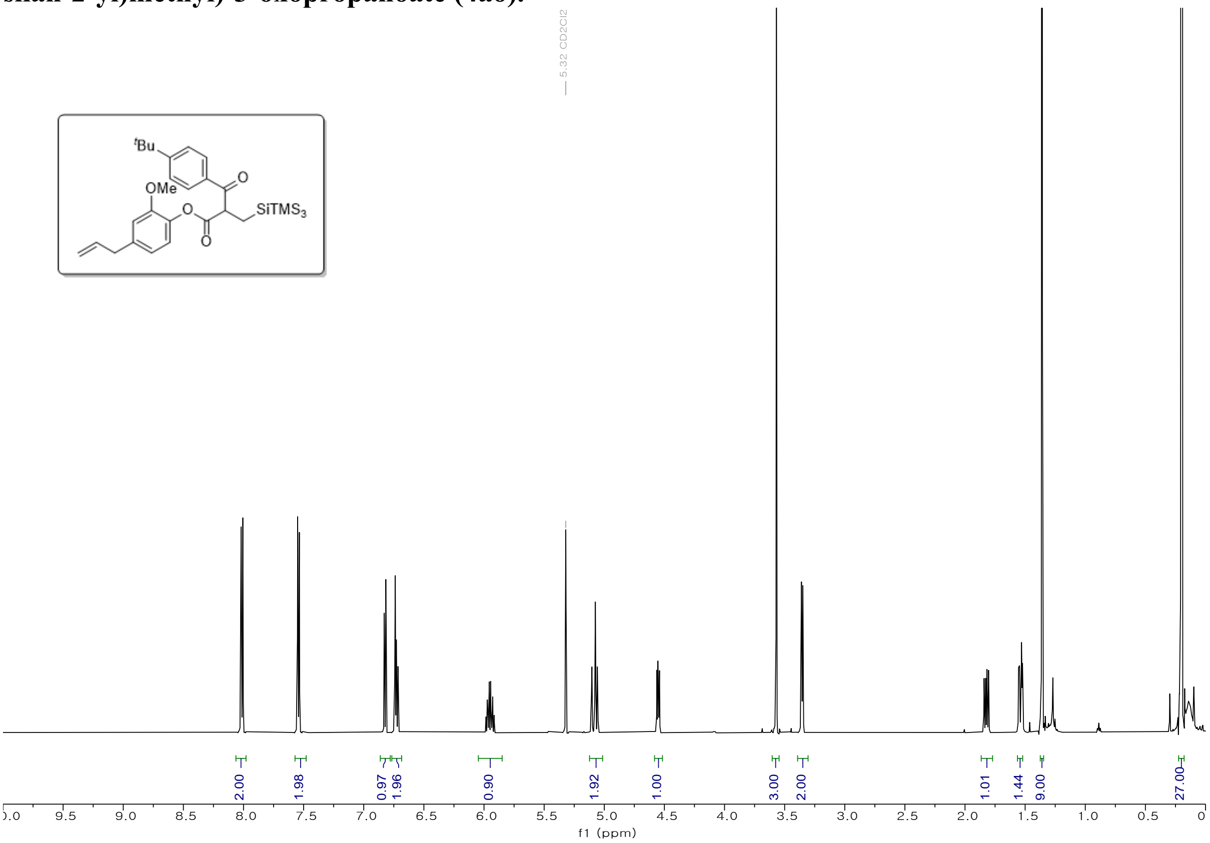

600 MHz, ^{1}H NMR in CD_2Cl_2

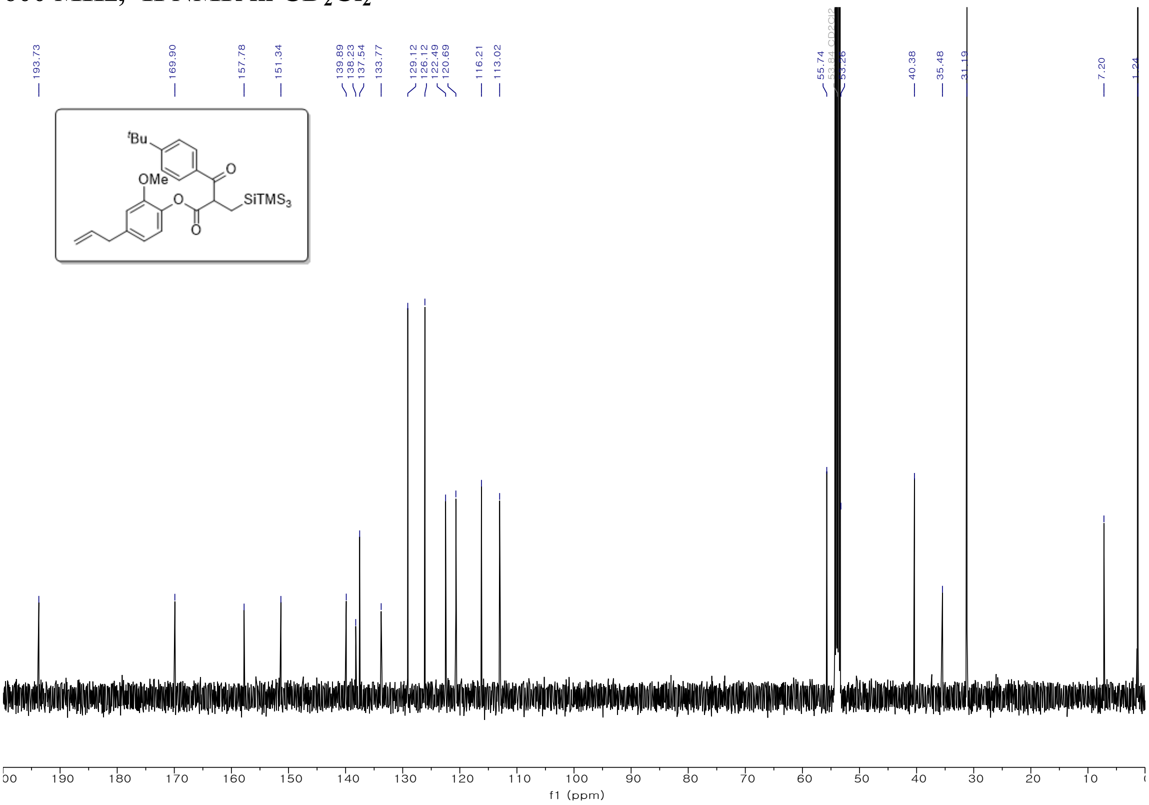

125 MHz, ^{13}C NMR in CD_2Cl_2

ethyl 2-((1,1,1,3,3,3-hexamethyl-2-(trimethylsilyl)trisilan-2-yl)methyl)-3-oxo-3-phenylpropanoate (4ap).

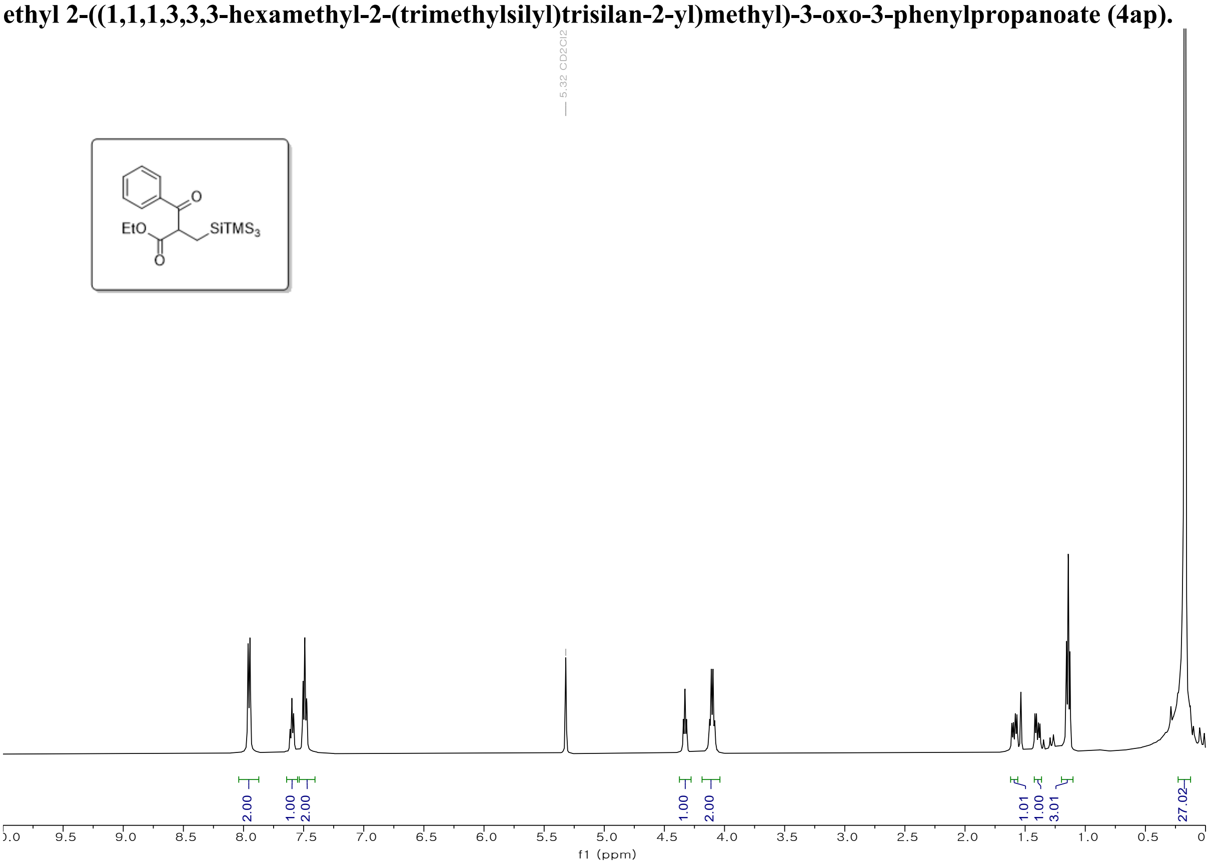

500 MHz, ^{1}H NMR in CD_2Cl_2

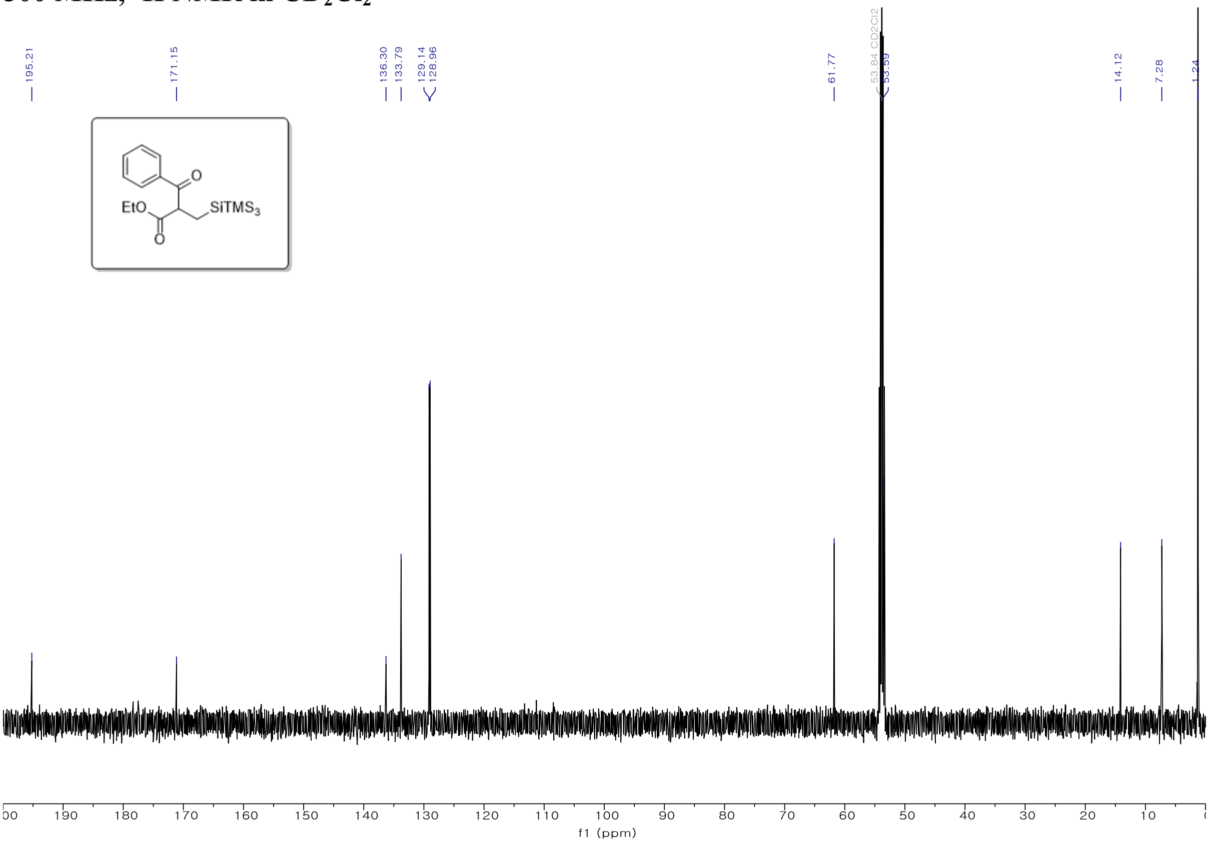

125 MHz, ^{13}C NMR in CD_2Cl_2

ethyl 2-((1,1,1,3,3,3-hexamethyl-2-(trimethylsilyl)trisilan-2-yl)methyl)-3-oxo-3-(p-tolyl)propanoate (4aq).

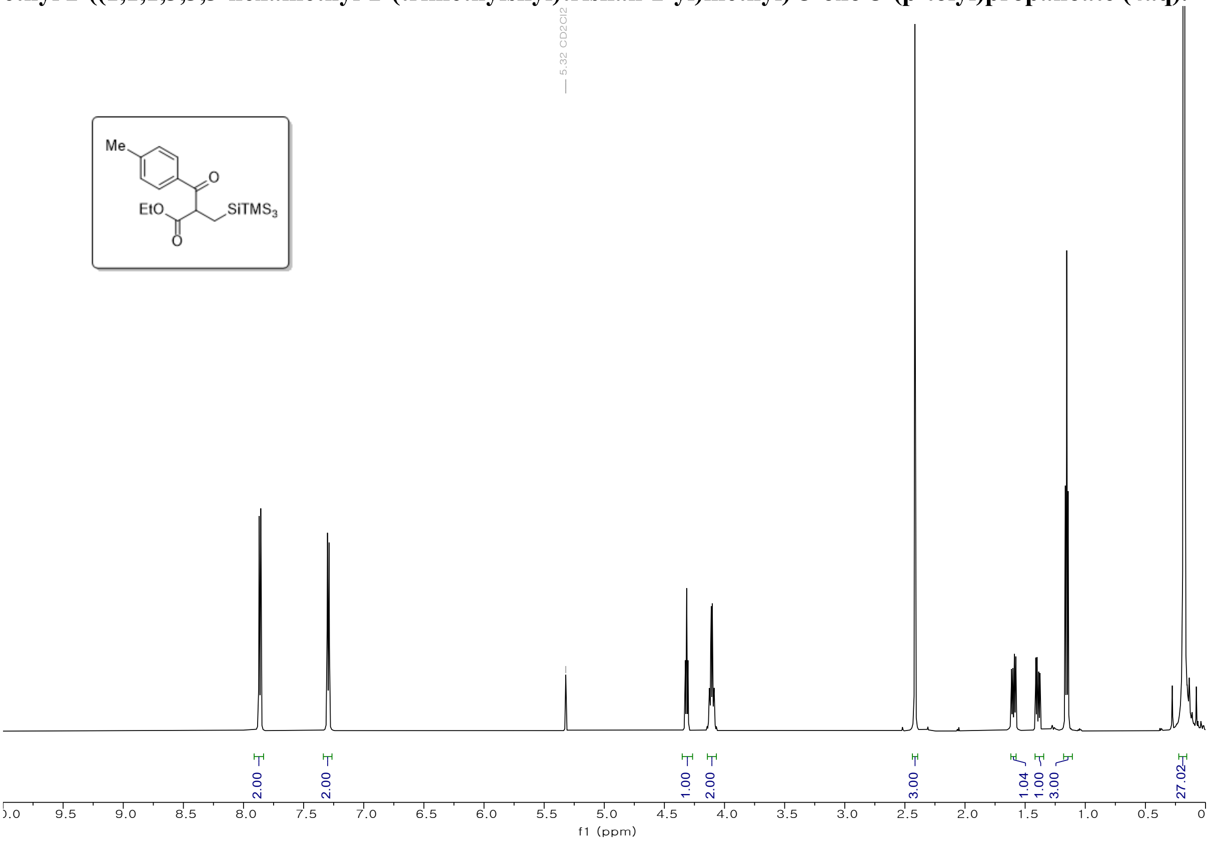

600 MHz, ^{1}H NMR in CD_2Cl_2

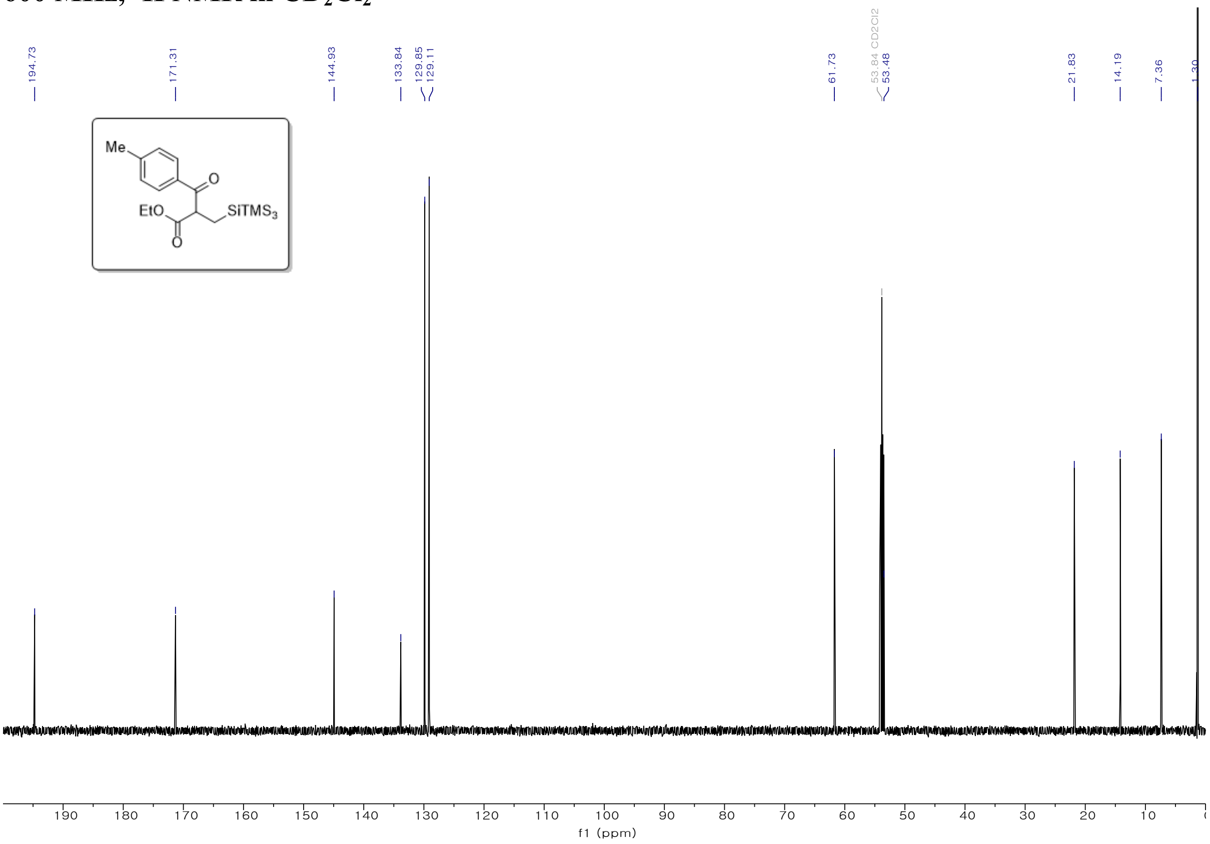

150 MHz, ^{13}C NMR in CD_2Cl_2

ethyl 2-((1,1,1,3,3,3-hexamethyl-2-(trimethylsilyl)trisilan-2-yl)methyl)-3-(4-methoxyphenyl)-3-oxopropanoate (4ar).

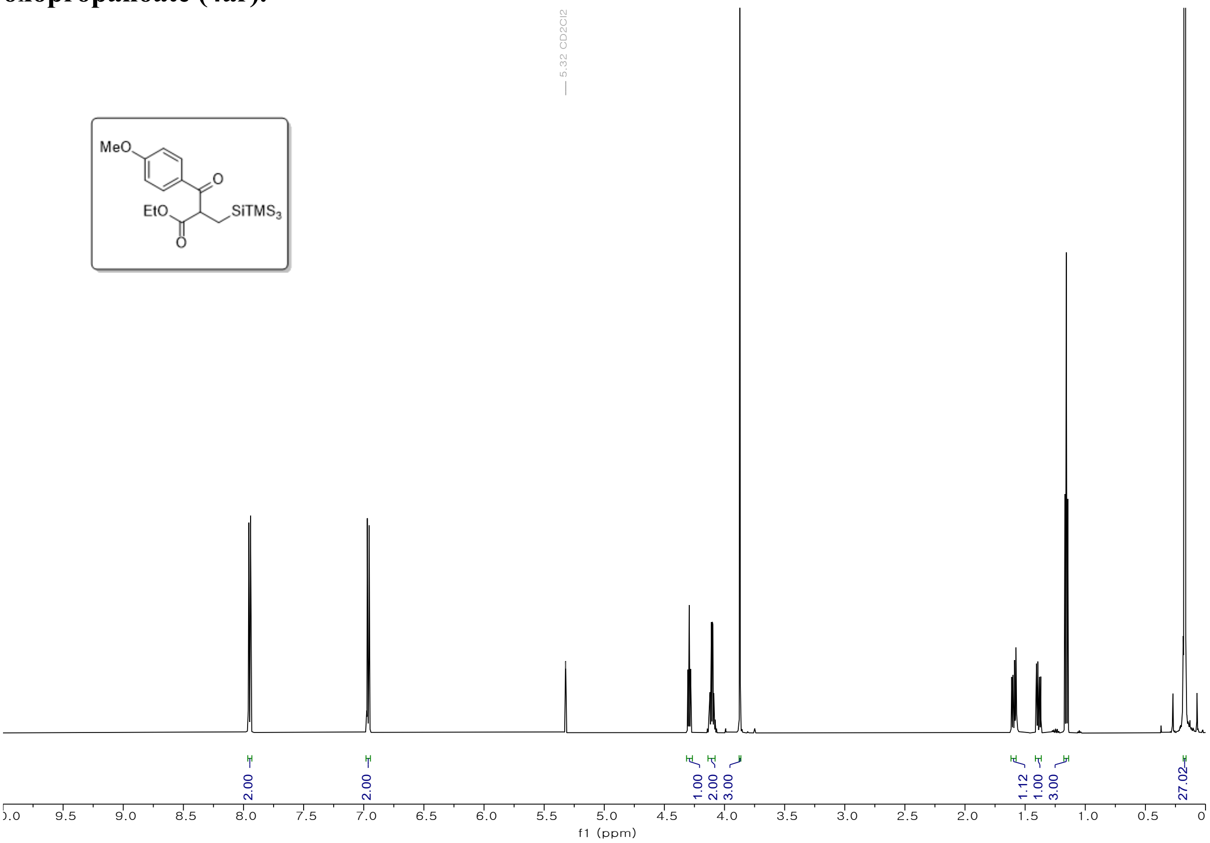

600 MHz, ^{1}H NMR in CD_2Cl_2

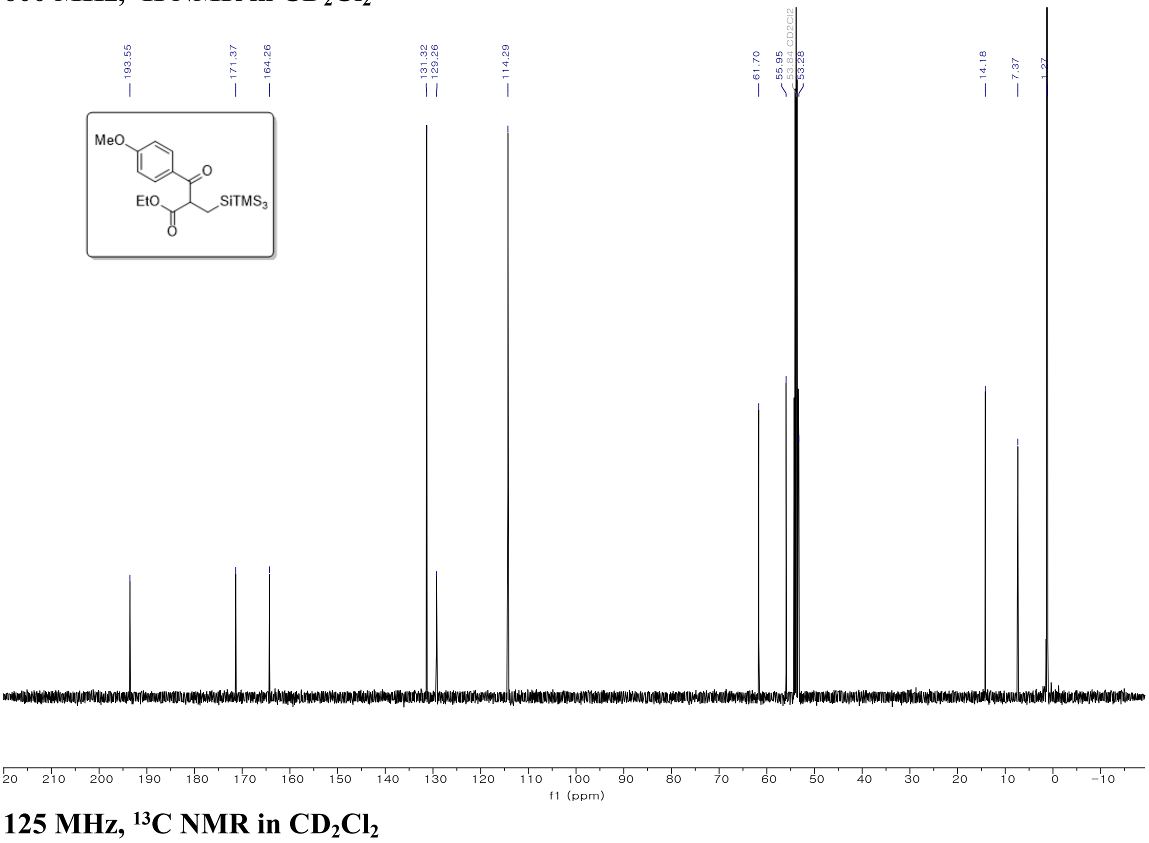

125 MHz, ^{13}C NMR in CD_2Cl_2

ethyl 2-((1,1,1,3,3,3-hexamethyl-2-(trimethylsilyl)trisilan-2-yl)methyl)-3-oxo-3-(4-(trifluoromethoxy)phenyl)propanoate (4as).

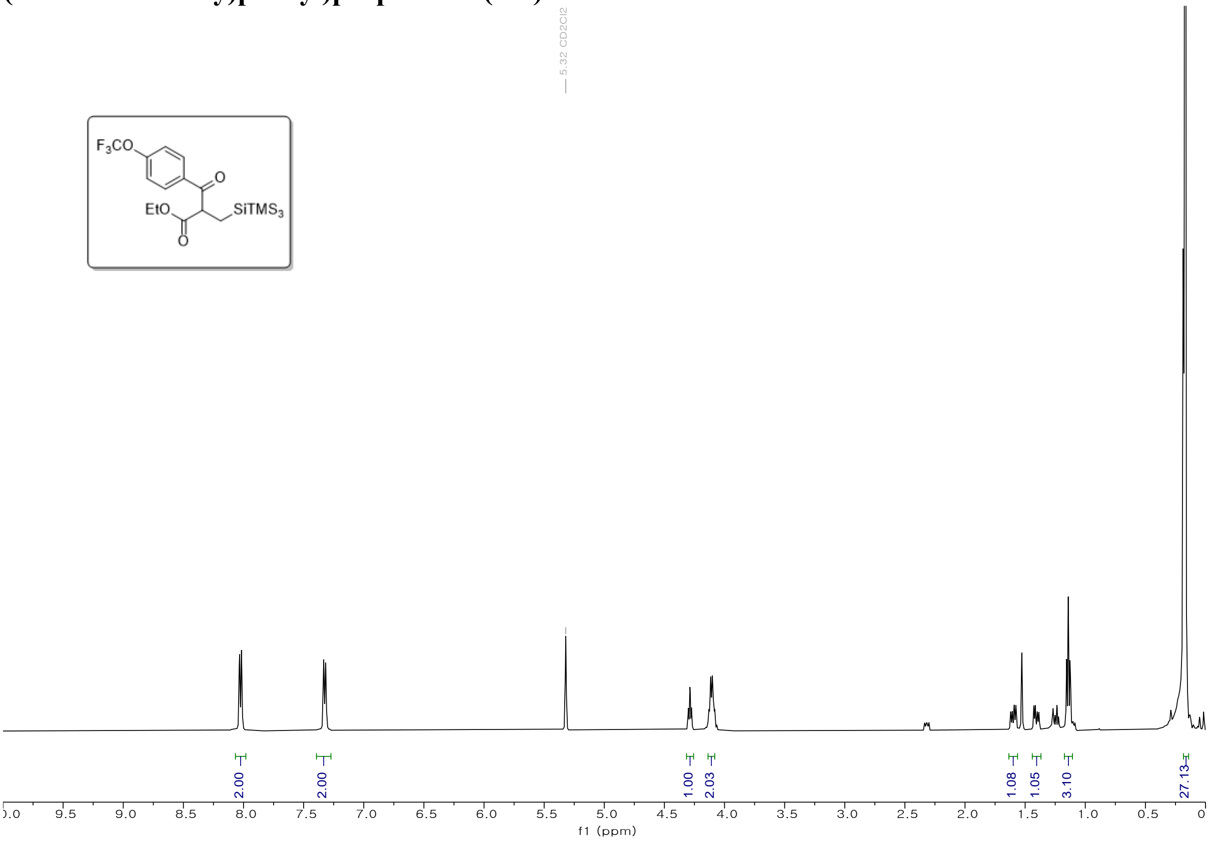

500 MHz, ^{1}H NMR in CD_2Cl_2

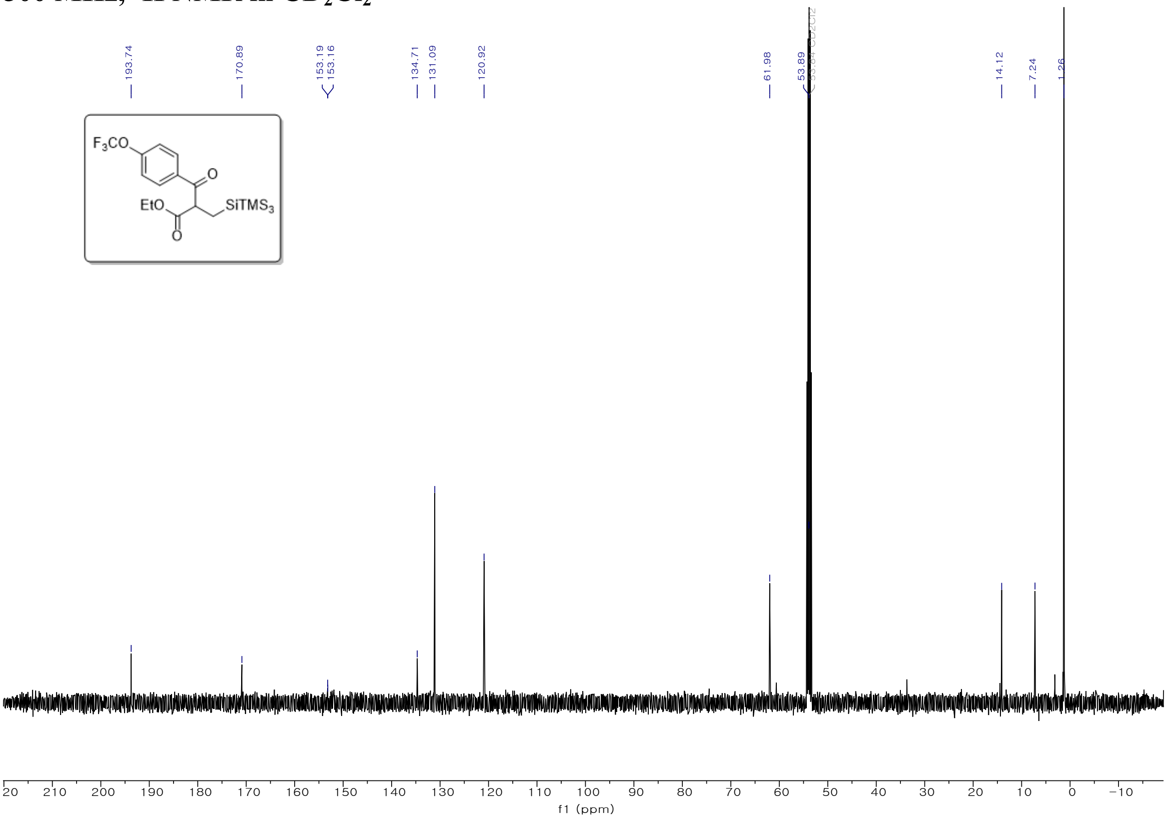

125 MHz, ^{13}C NMR in CD_2Cl_2

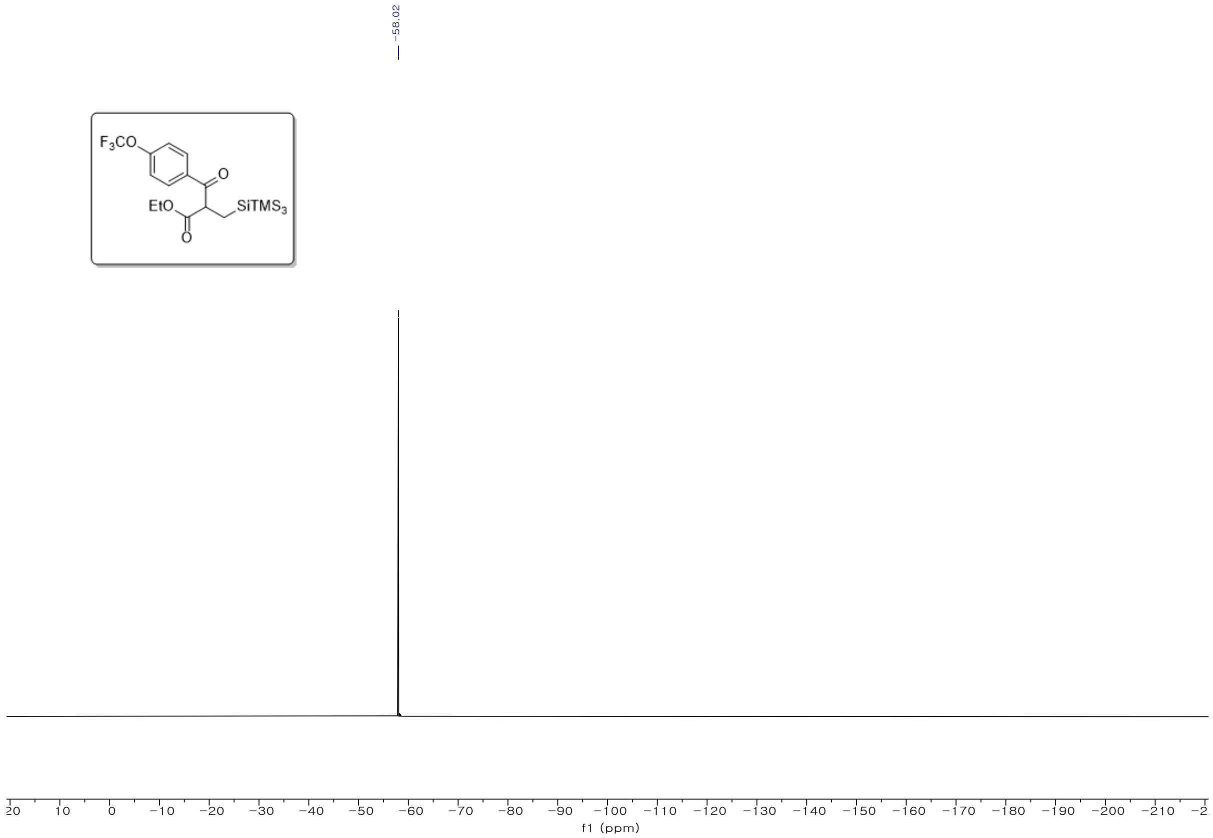

471 MHz, ^{19}F NMR in CD_2Cl_2

ethyl 3-(4-fluorophenyl)-2-((1,1,1,3,3,3-hexamethyl-2-(trimethylsilyl)trisilan-2-yl)methyl)-3-oxo propanoate (4at).

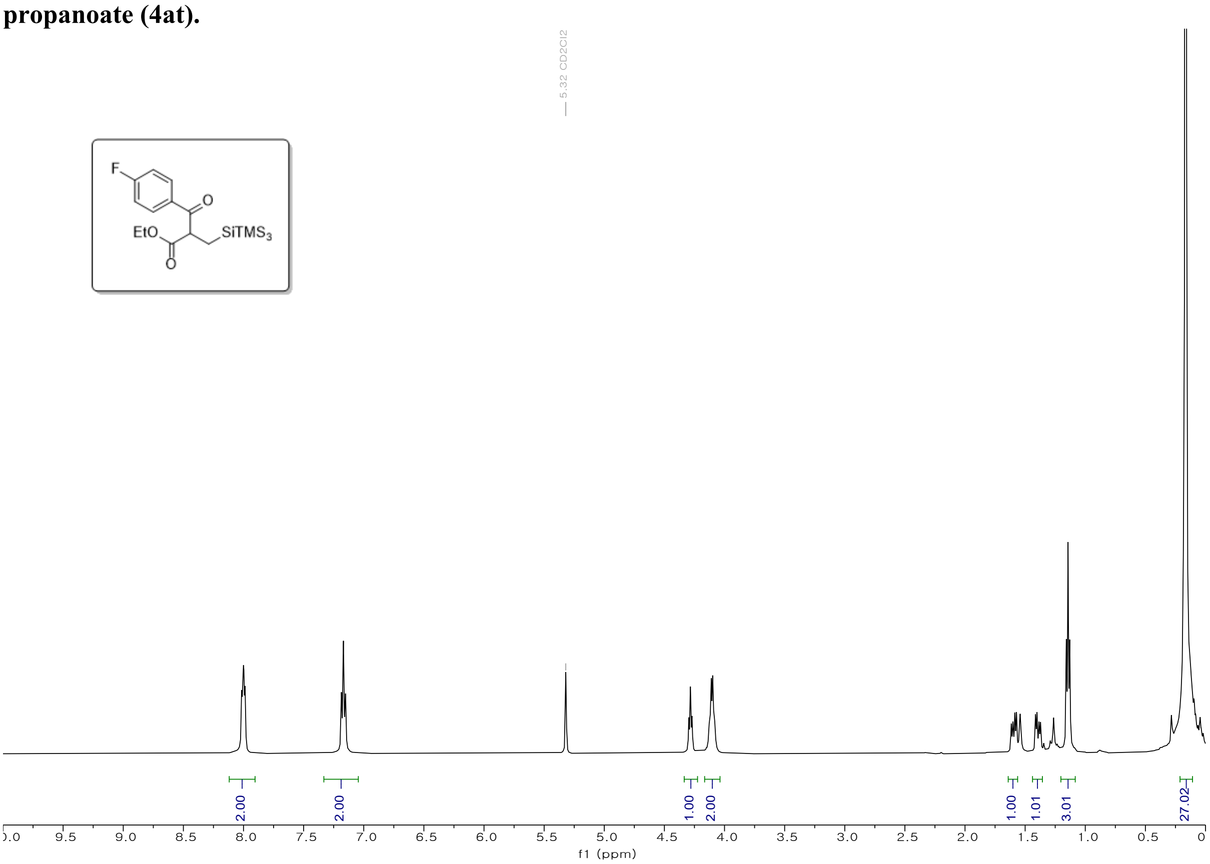

600 MHz, ^{1}H NMR in CD_2Cl_2

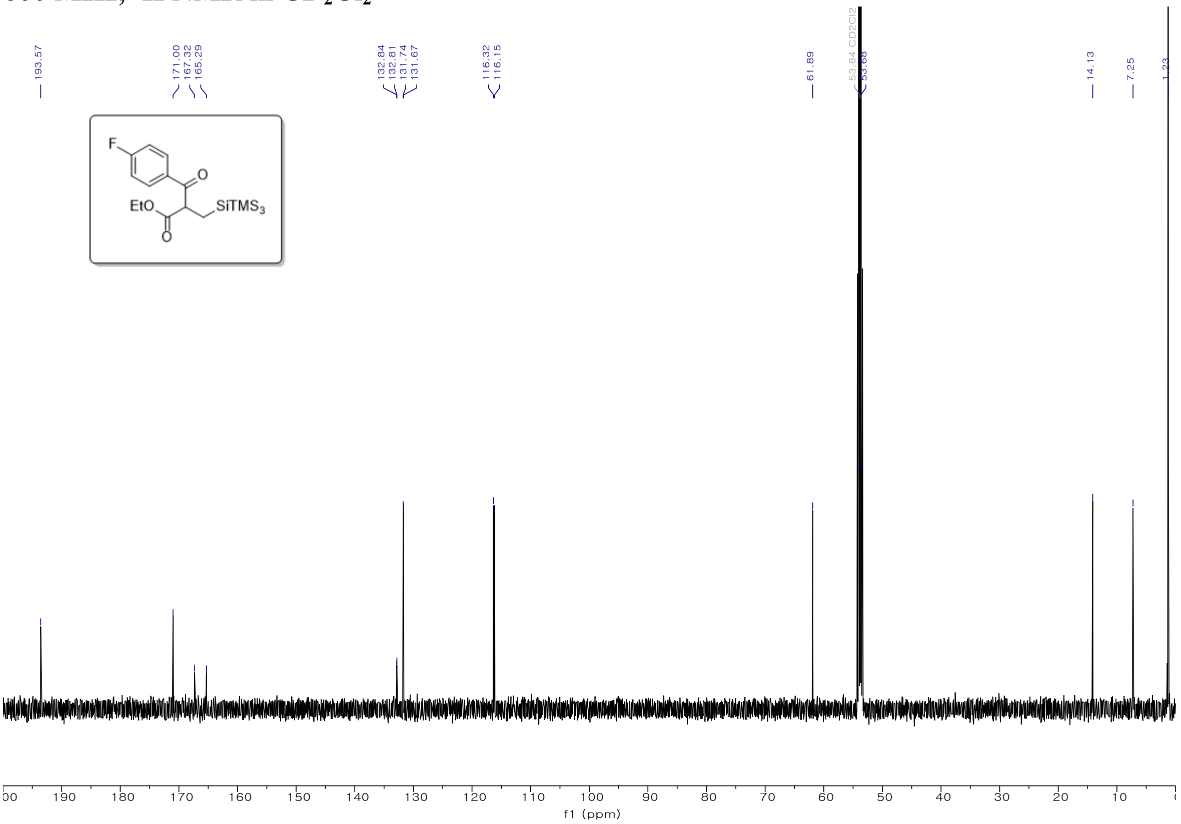

125 MHz, ^{13}C NMR in CD_2Cl_2

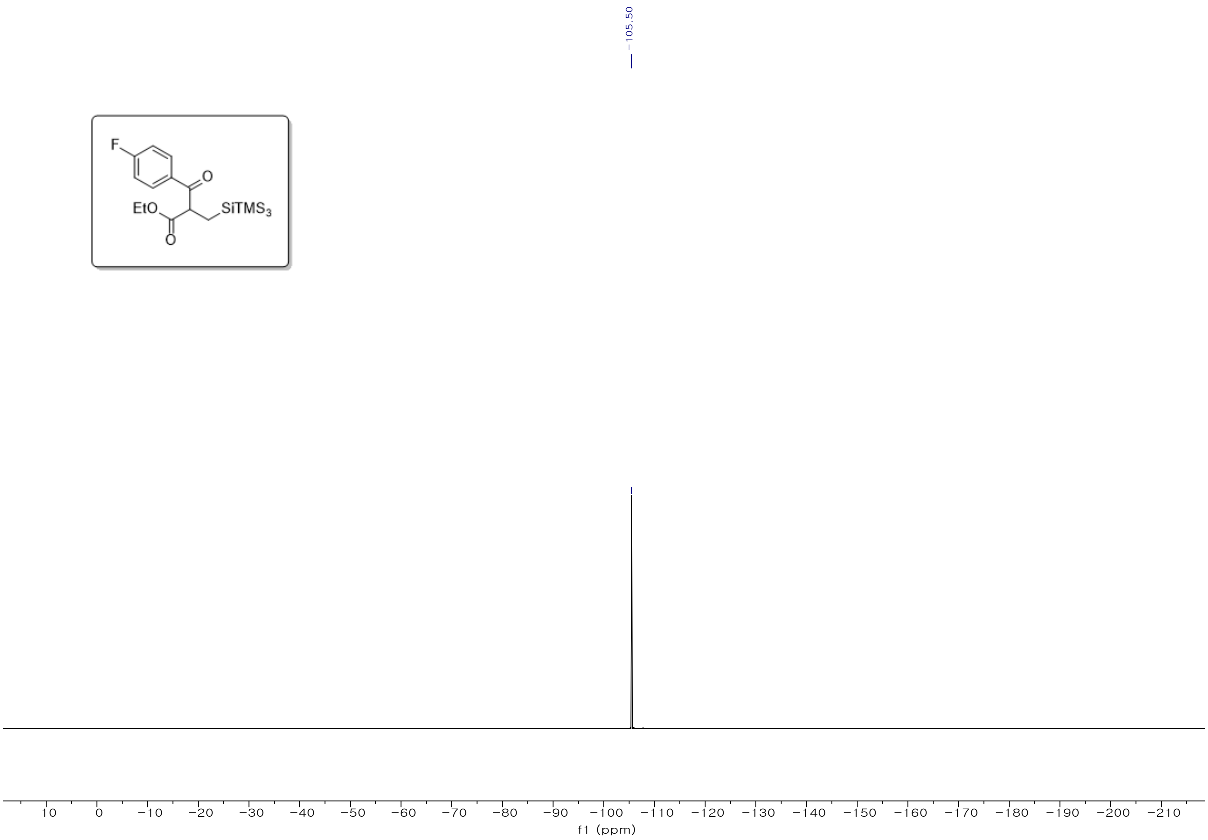

376 MHz, ^{19}F NMR in CD_2Cl_2

ethyl 2-((1,1,1,3,3,3-hexamethyl-2-(trimethylsilyl)trisilan-2-yl)methyl)-3-(3-methoxyphenyl)-3-oxopropanoate (4au).

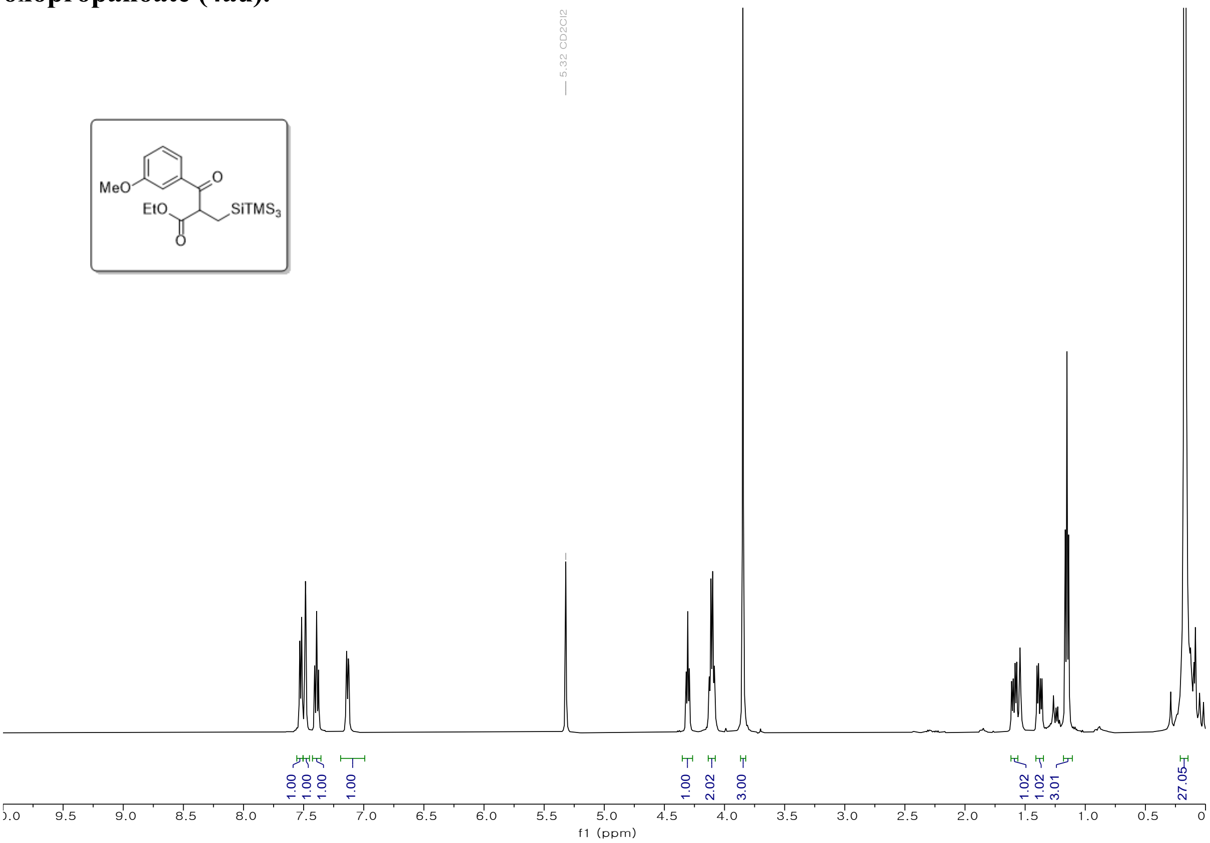

500 MHz, ^{1}H NMR in CD_2Cl_2

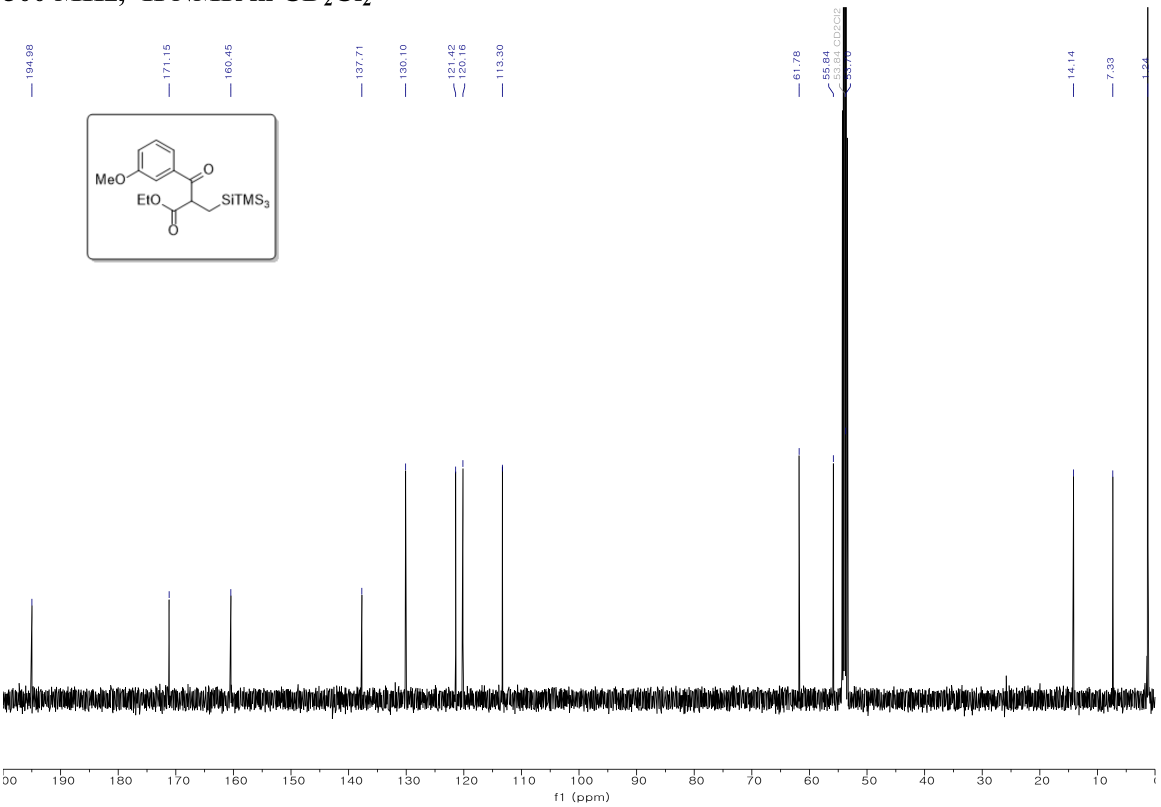

125 MHz, ^{13}C NMR in CD_2Cl_2

ethyl 3-([1,1'-biphenyl]-3-yl)-2-((1,1,1,3,3,3-hexamethyl-2-(trimethylsilyl)trisilan-2-yl)methyl)-3-oxopropanoate (4av).

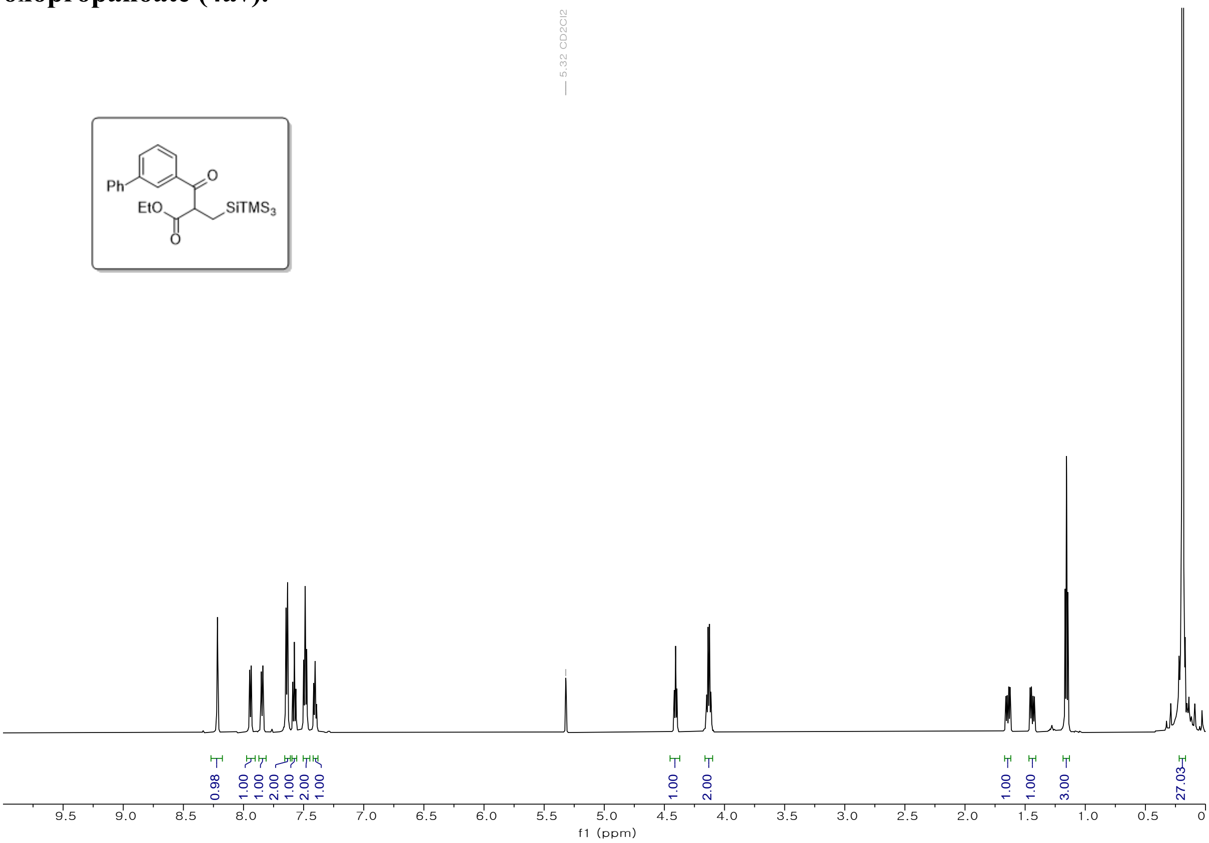

600 MHz, ^{1}H NMR in CD_2Cl_2

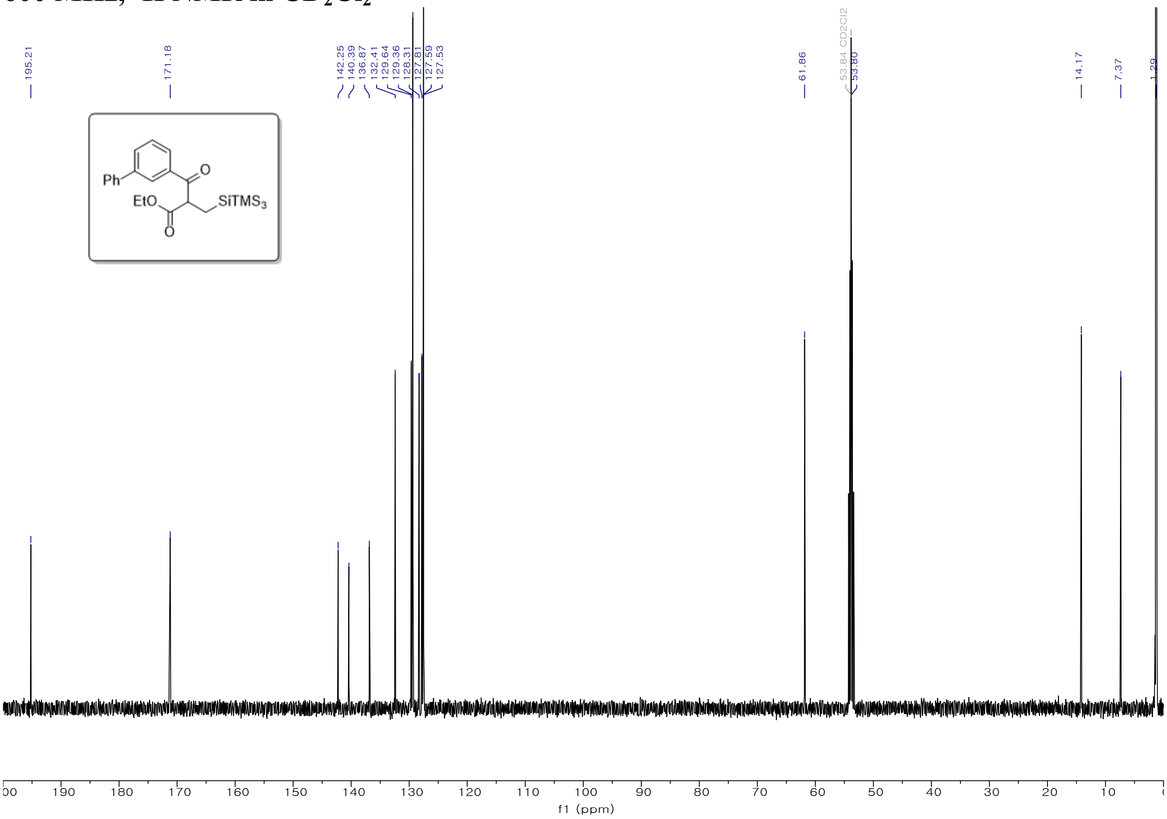

125 MHz, ^{13}C NMR in CD_2Cl_2

ethyl 3-(3-fluorophenyl)-2-((1,1,1,3,3,3-hexamethyl-2-(trimethylsilyl)trisilan-2-yl)methyl)-3-oxopropanoate (4aw).

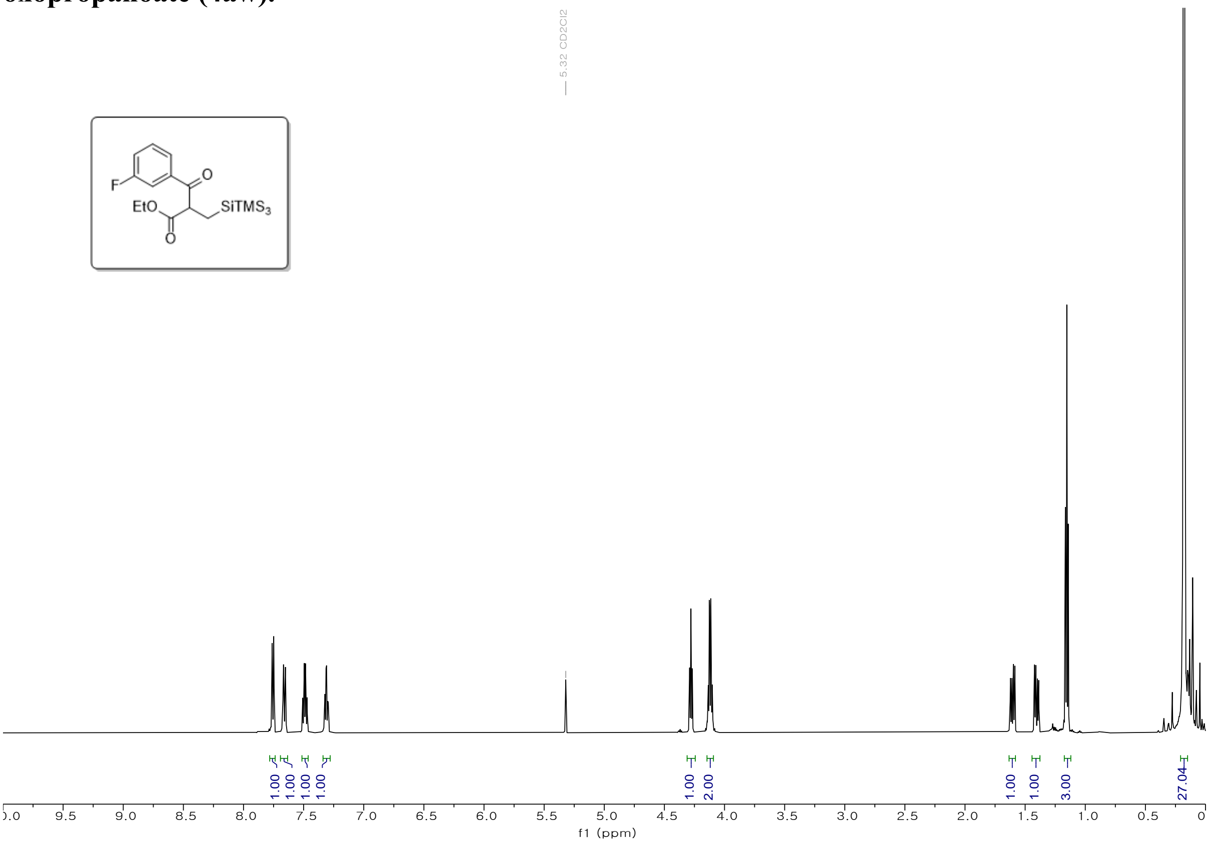

600 MHz, ^{1}H NMR in CD_2Cl_2

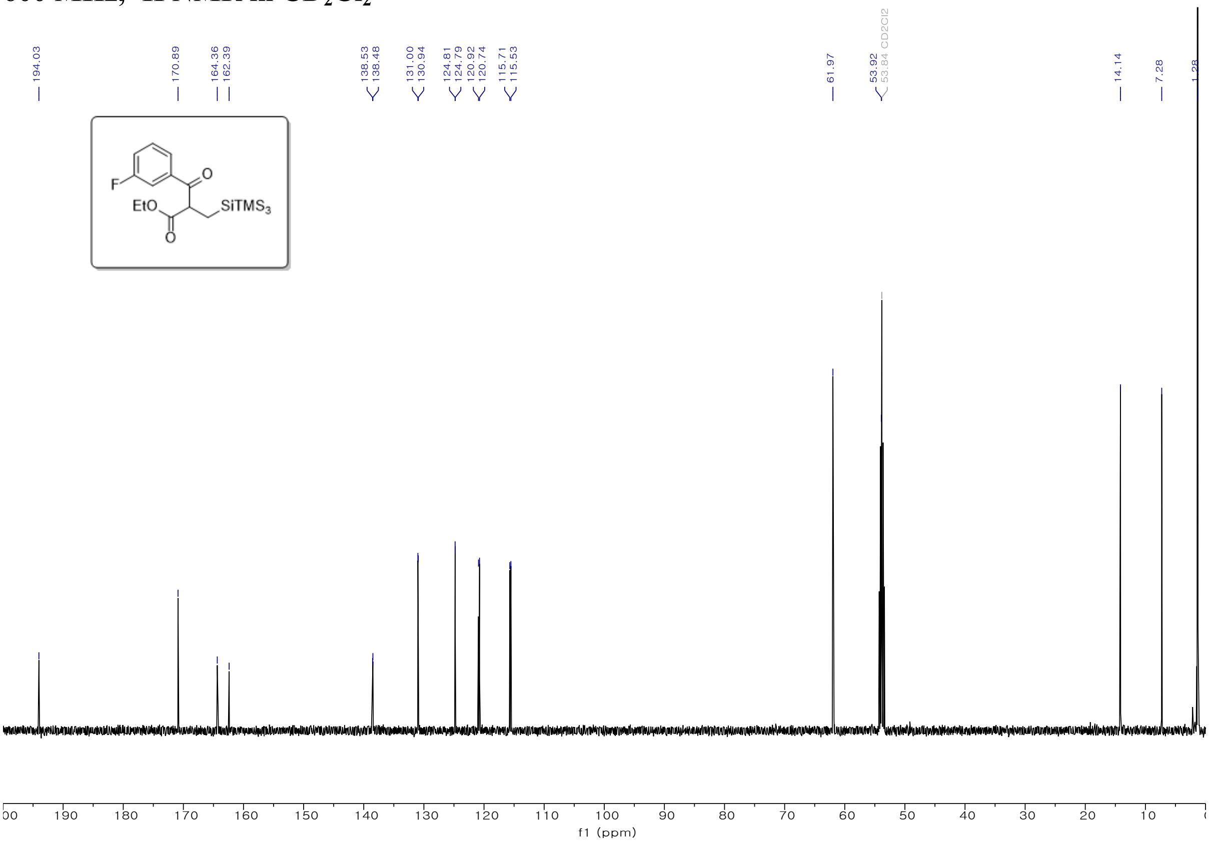

125 MHz, ^{13}C NMR in CD_2Cl_2

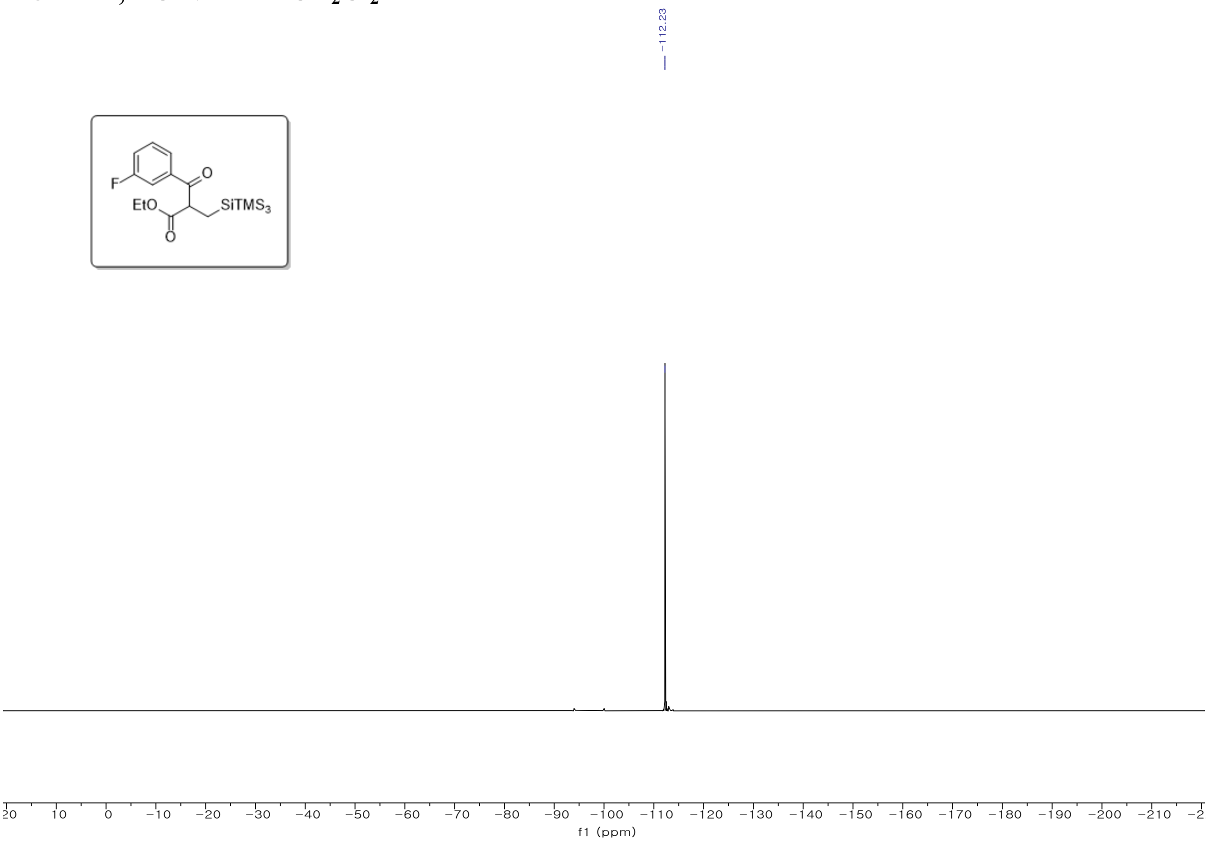

376 MHz, ^{19}F NMR in CD_2Cl_2

ethyl 2-((1,1,1,3,3,3-hexamethyl-2-(trimethylsilyl)trisilan-2-yl)methyl)-3-oxo-3-(3-(trifluoromethyl)phenyl)propanoate (4ax).

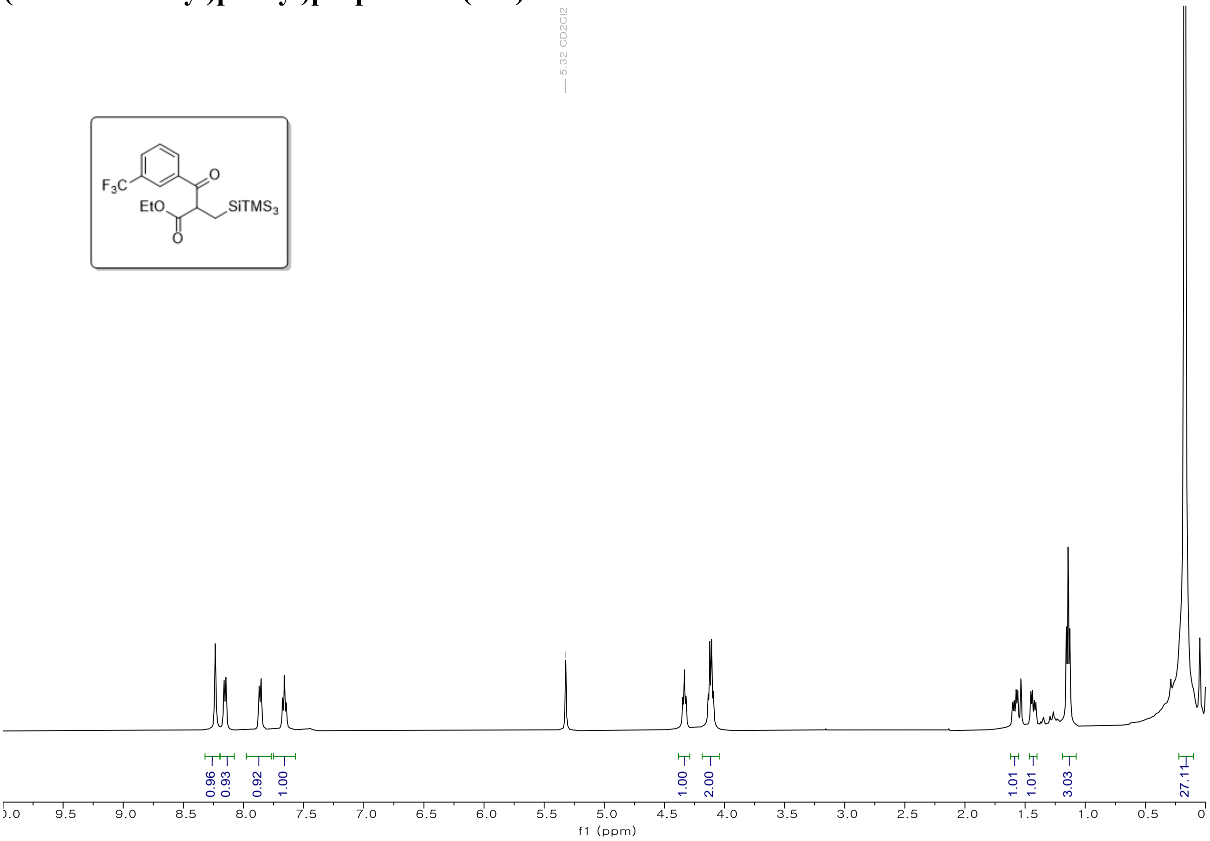

500 MHz, ¹H NMR in CD₂Cl₂

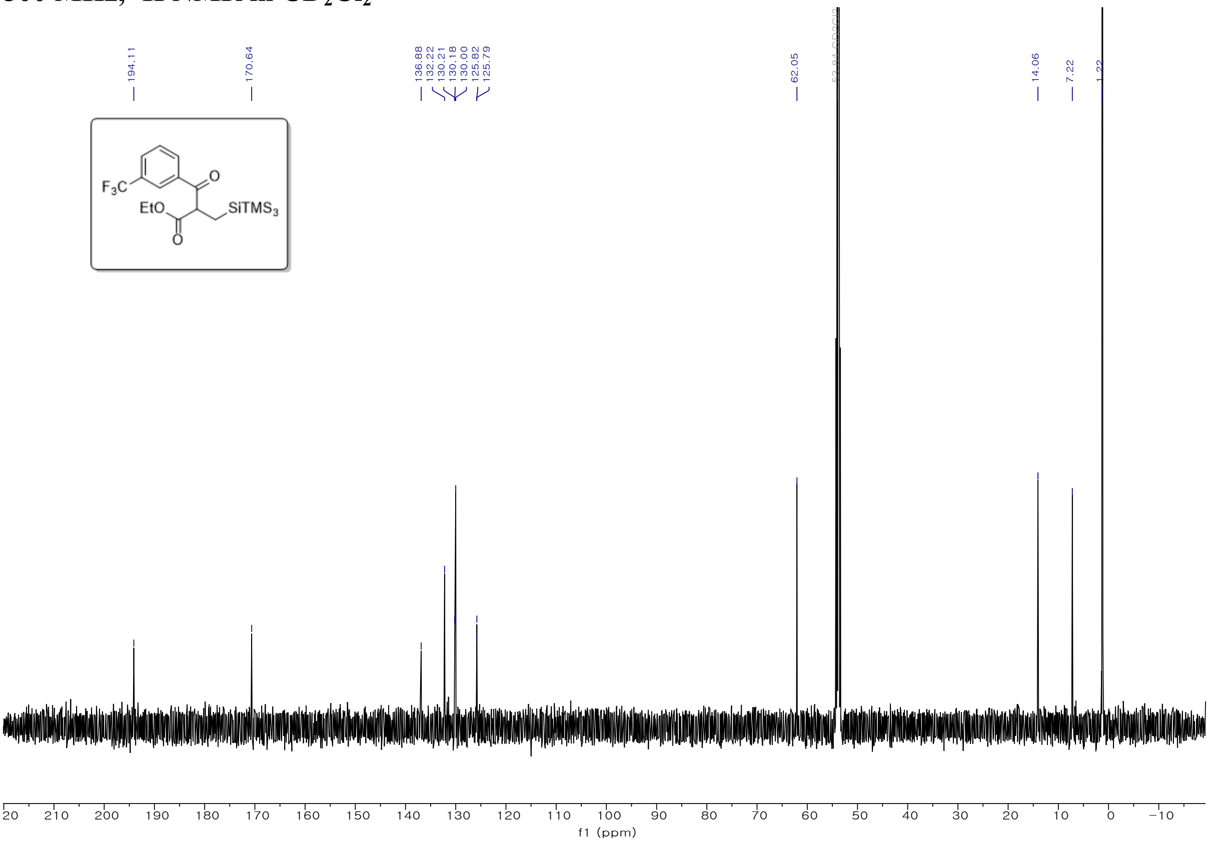

125 MHz, ^{13}C NMR in CD_2Cl_2

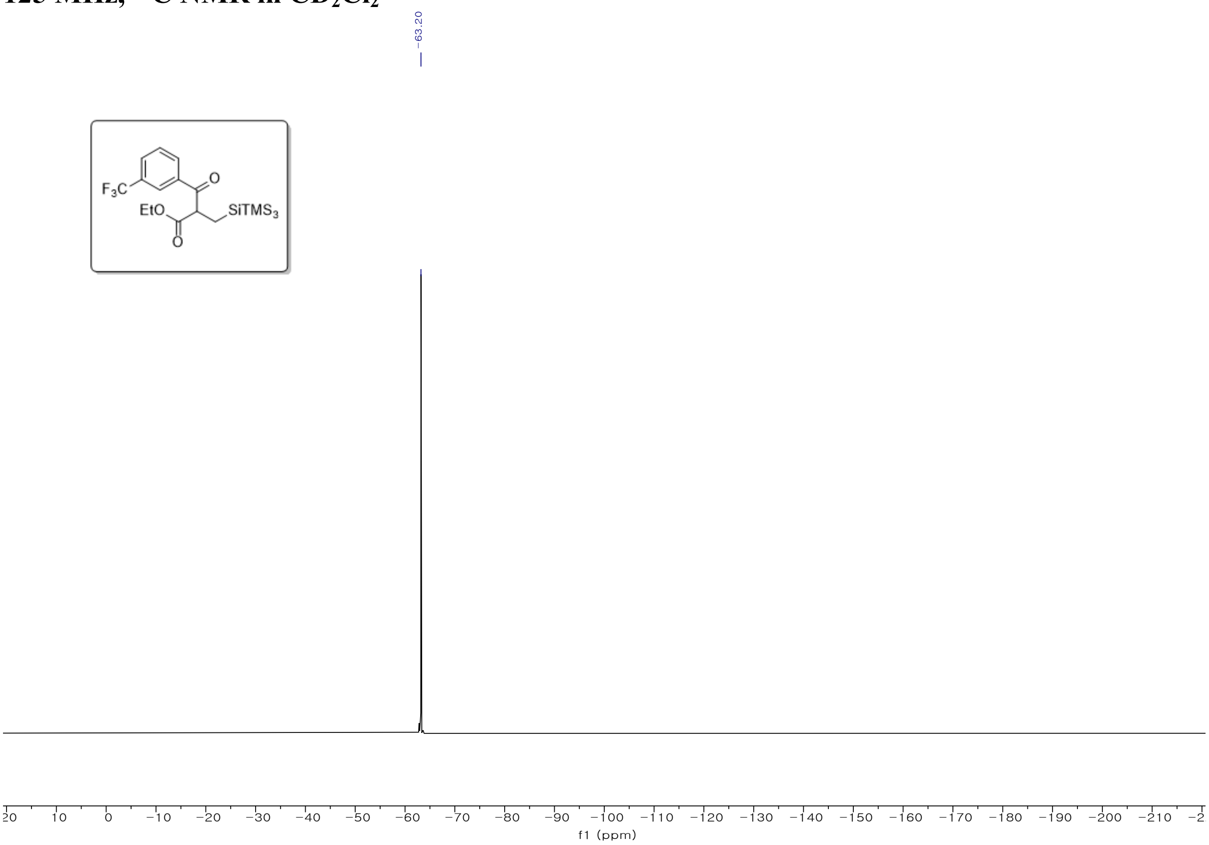

471 MHz, ^{19}F NMR in CD_2Cl_2

methyl 3-(3-ethoxy-2-((1,1,1,3,3,3-hexamethyl-2-(trimethylsilyl)trisilan-2-yl)methyl)-3-oxopropanoyl)benzoate (4ay).

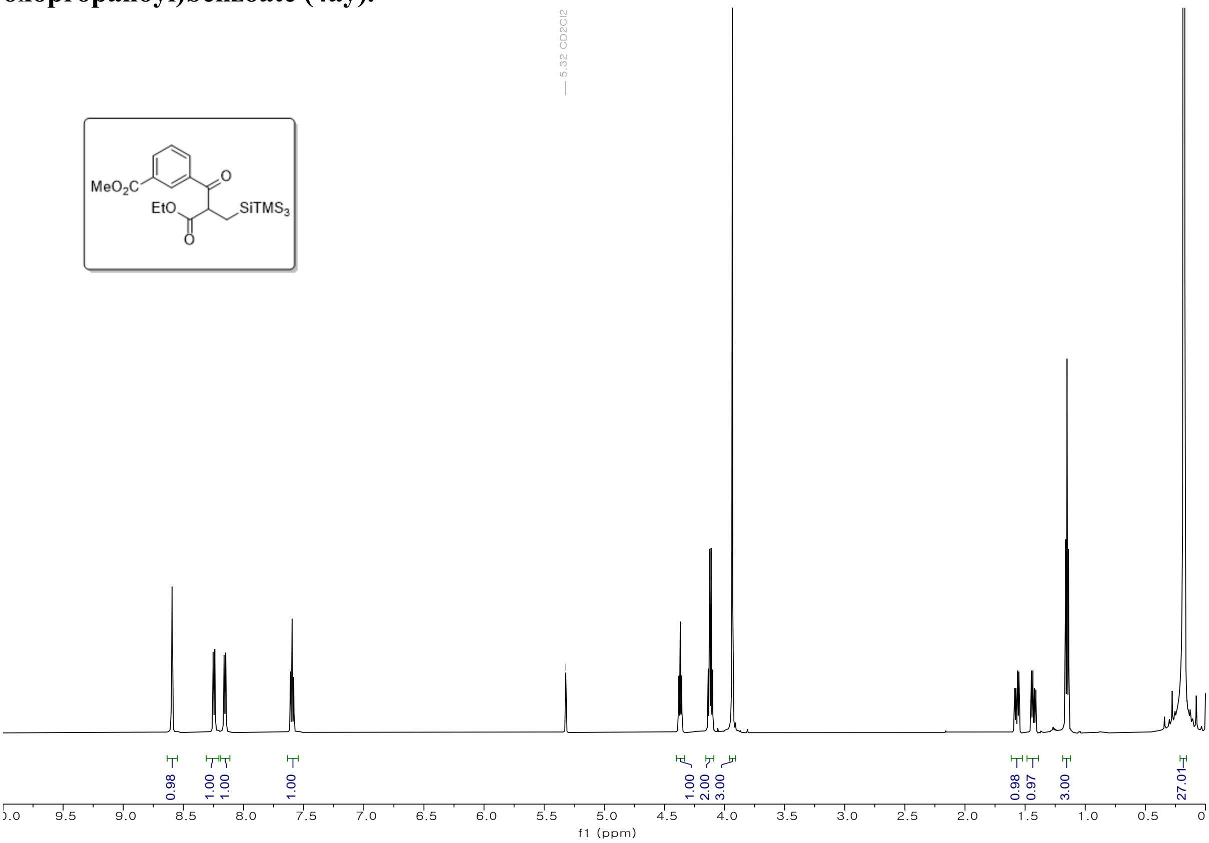

600 MHz, ^{1}H NMR in CD_2Cl_2

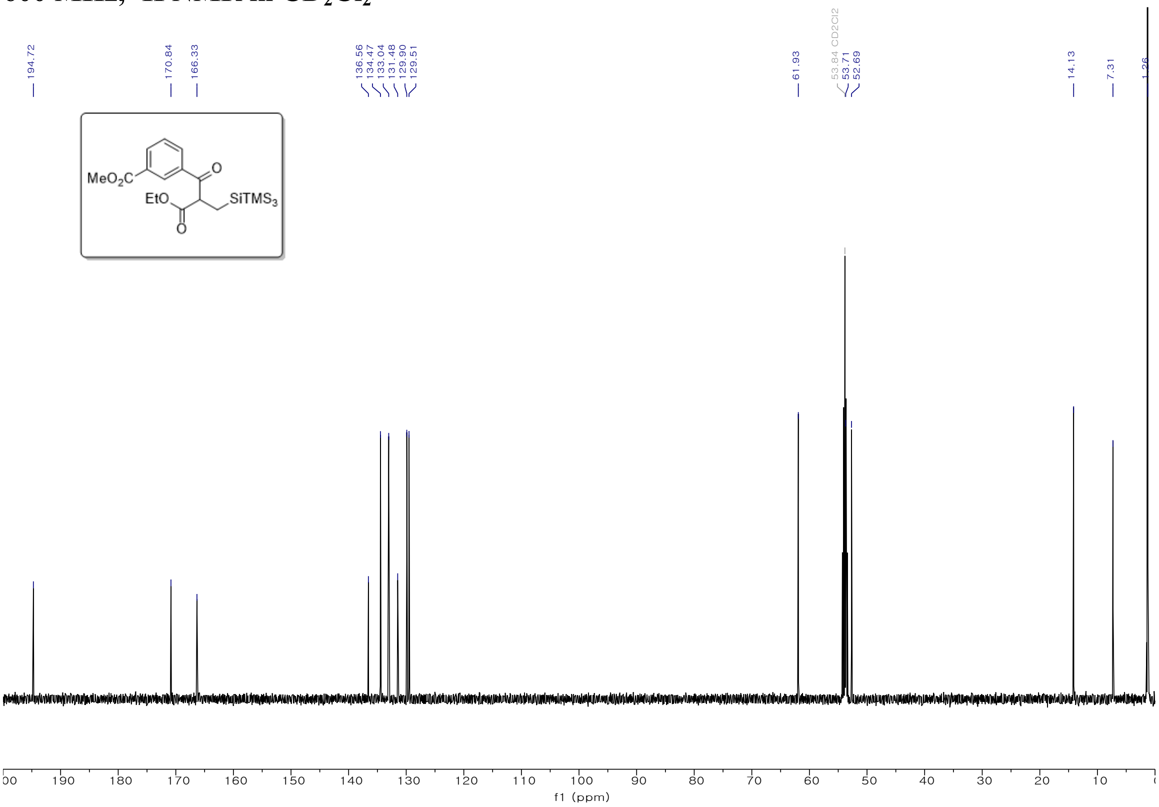

125 MHz, ^{13}C NMR in CD_2Cl_2

ethyl 2-((1,1,1,3,3,3-hexamethyl-2-(trimethylsilyl)trisilan-2-yl)methyl)-3-oxo-3-(o-tolyl)propanoate (4az).

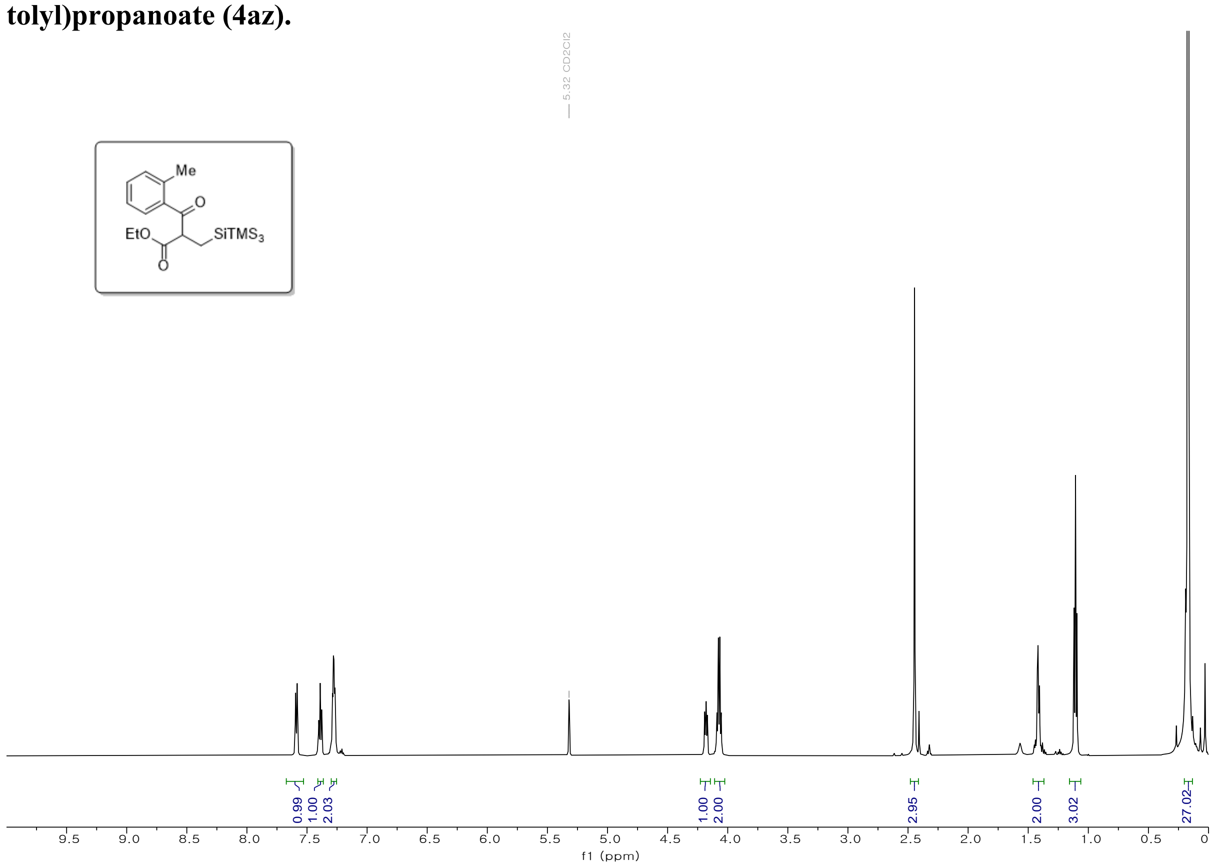

600 MHz, ^{1}H NMR in CD_2Cl_2

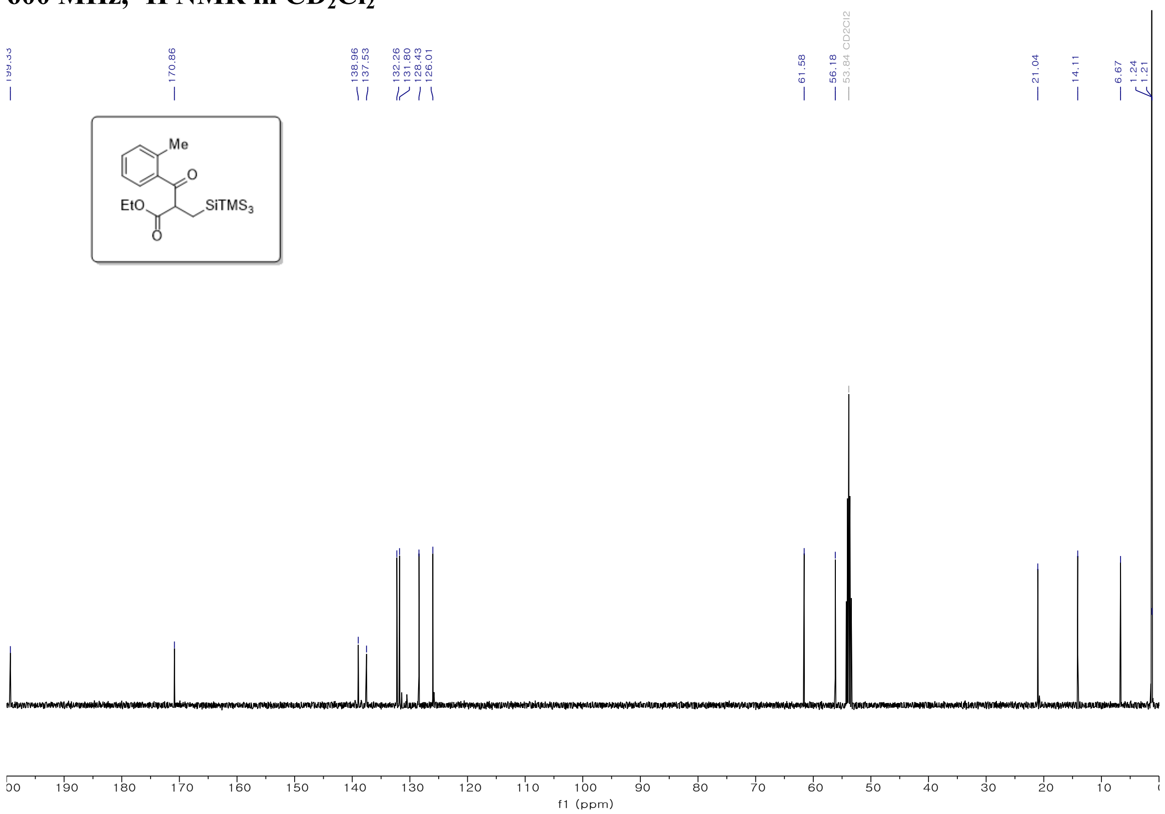

125 MHz, ^{13}C NMR in CD_2Cl_2

ethyl 2-((1,1,1,3,3,3-hexamethyl-2-(trimethylsilyl)trisilan-2-yl)methyl)-3-(2-methoxyphenyl)-3-oxopropanoate (4ba).

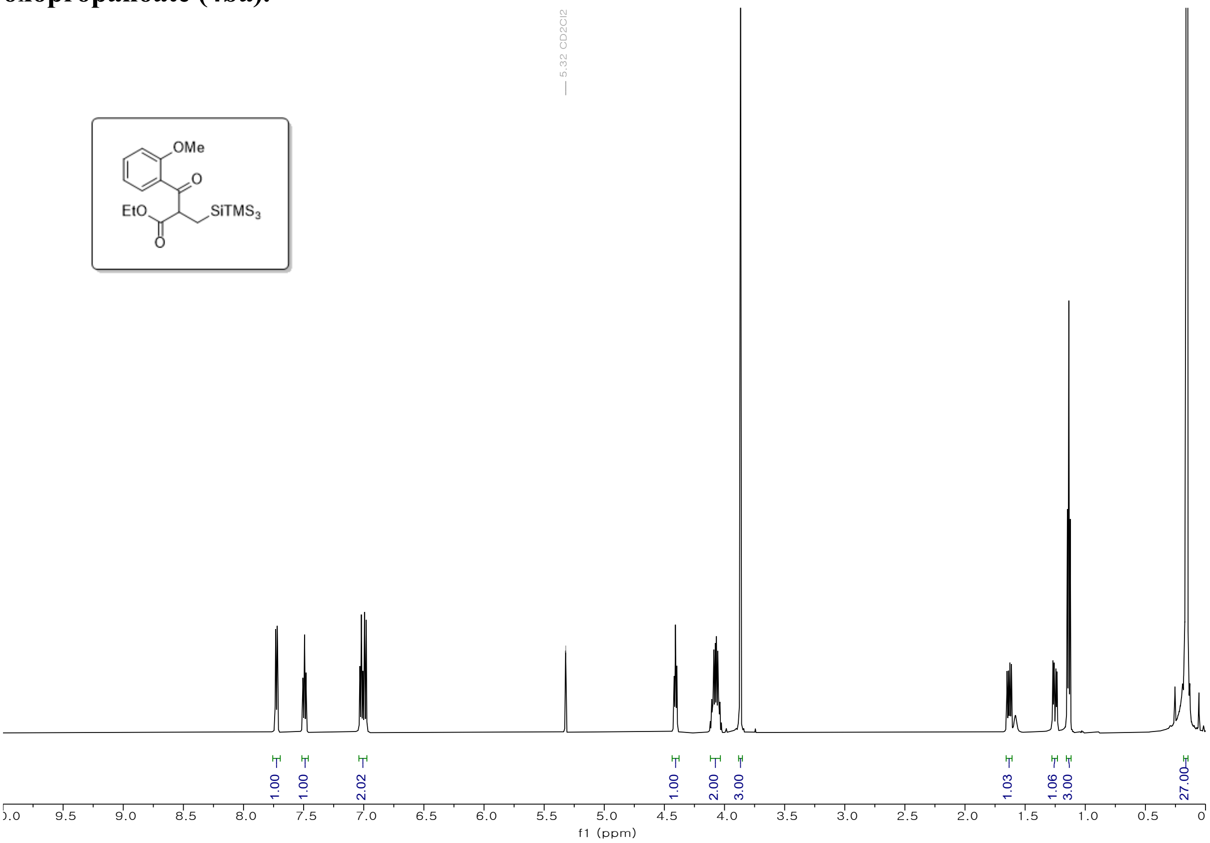

600 MHz, ^{1}H NMR in CD_2Cl_2

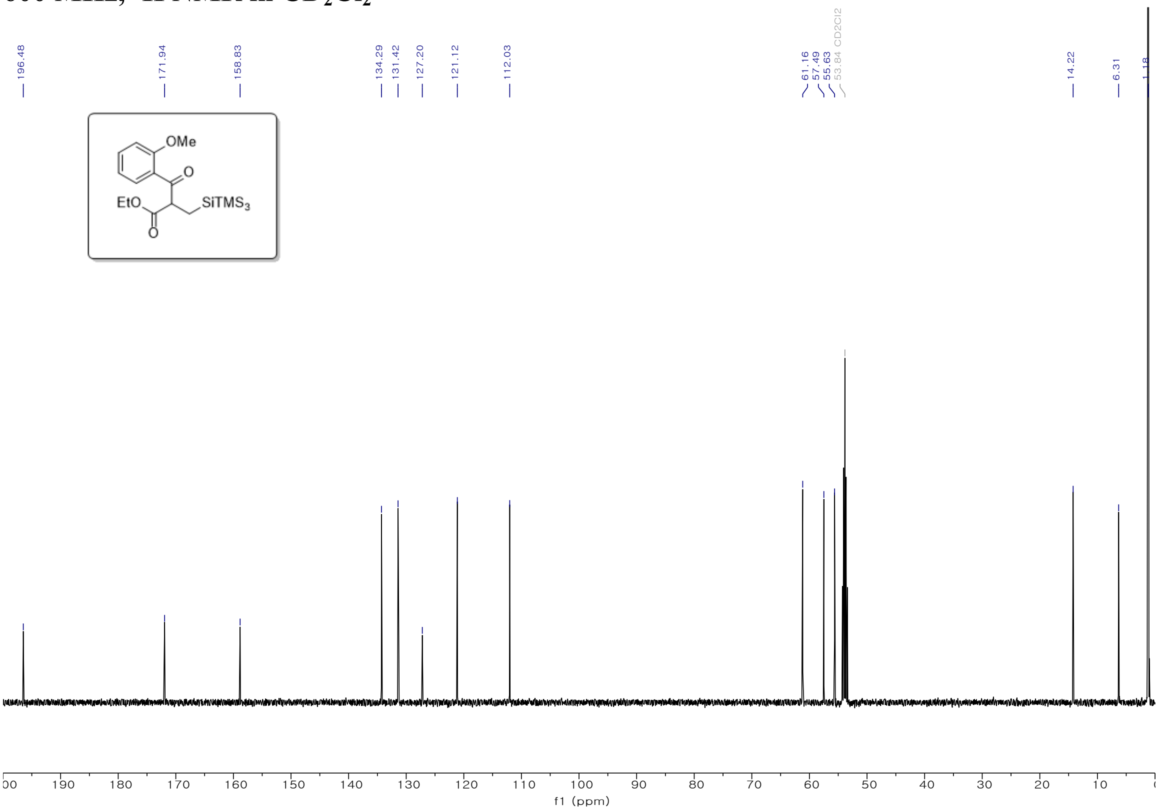

125 MHz, ^{13}C NMR in CD_2Cl_2
